# Enantioselective construction of *C*-chiral allylic sulfilimines *via* the iridium-catalyzed allylic amination with *S*,*S*-diphenylsulfilimine: asymmetric synthesis of primary allylic amines†
†Electronic supplementary information (ESI) available. See DOI: 10.1039/c4sc01317d



**DOI:** 10.1039/c4sc01317d

**Published:** 2014-09-08

**Authors:** Rebecca L. Grange, Elizabeth A. Clizbe, Emma J. Counsell, P. Andrew Evans

**Affiliations:** a Queen's University , Department of Chemistry , 90 Bader Lane , Kingston , ON K7L 3N6 , Canada . Email: andrew.evans@chem.queensu.ca; b The University of Liverpool , Department of Chemistry , Crown Street , Liverpool , L69 7ZD , UK

## Abstract

We have devised a highly regio- and enantioselective iridium-catalyzed allylic amination reaction with the sulfur-stabilized aza-ylide, *S*,*S*-diphenylsulfilimine. This process provides a robust and scalable method for the construction of aryl-, alkyl- and alkenyl-substituted *C*-chiral allylic sulfilimines, which are important functional groups for organic synthesis. Additionally, the combination of the allylic amination with an *in situ* deprotection of the sulfilimine constitutes a convenient one-pot protocol for the construction of chiral nonracemic primary allylic amines.

## Abstract 2

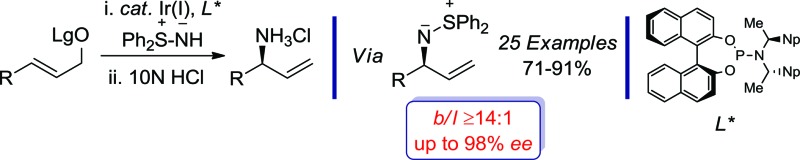
We have devised a highly regio- and enantioselective iridium-catalyzed allylic amination reaction with the sulfur-stabilized aza-ylide, *S*,*S*-diphenylsulfilimine.

## Introduction

Sulfur-stabilized aza-ylides, as exemplified by sulfilimines, provide an interesting class of molecules that display unique and diverse reactivity.^1^ For example, the ylide character of the nitrogen–sulfur group provides an ambidentate species that can either undergo nucleophilic or electrophilic aziridination of electron-deficient and electron-rich olefins, respectively.^2,3^ Sulfilimines also participate in cycloaddition reactions, which provides valuable opportunities for target directed synthesis.^1^ Notwithstanding the distinctive synthetic attributes of the sulfilimine, it represents a rather intriguing functional group in so much that it can be stereogenic at both carbon and sulfur. Although there are a number of convenient methods for the construction of enantioenriched *S*-chiral sulfilimines, the construction of *C*-chiral sulfilimines has not been forthcoming. Furthermore, sulfilimines provide important synthetic targets, as exemplified by their incorporation in pesticides and photographic recording materials.^4^ Additionally, a sulfilimine was recently implicated in the stabilization of collagen IV networks in the form of a critical cross-link between a hydroxylysine and methionine residue, which further underscores the growing significance of this unusual structural motif in organic chemistry.^5^

**Scheme 1:**
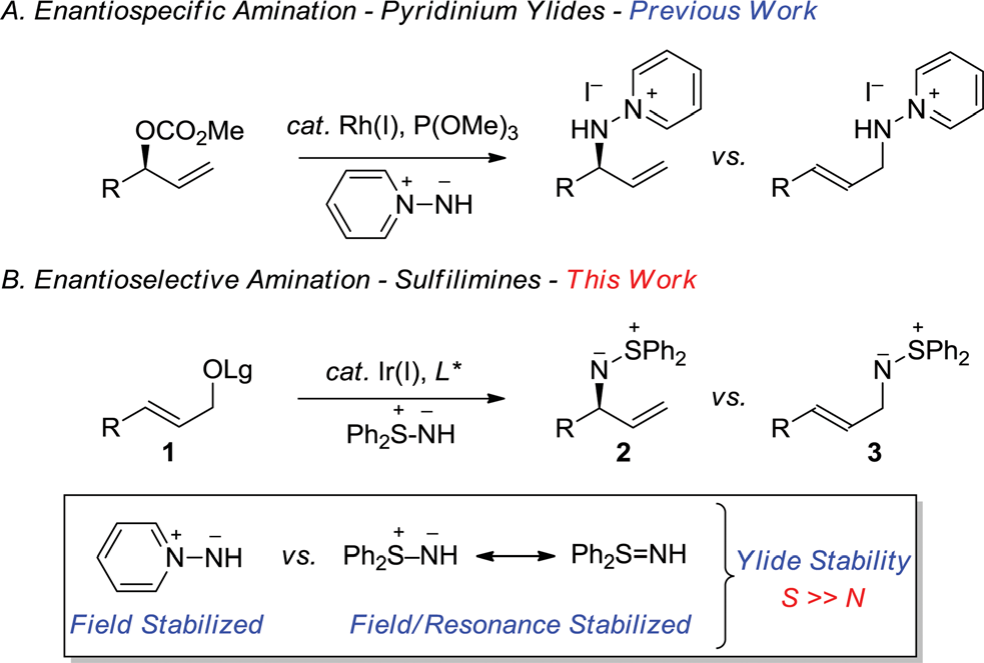
Enantiospecific and enantioselective metal-catalyzed allylic amination reactions with aza-ylides.

In a program directed towards the utilization of charge separated nucleophiles in the metal-catalyzed allylic substitution reaction;^6^ we recently demonstrated the merit of pyridinium ylides, which provide a new class of air-stable and non-basic nitrogen nucleophiles (Scheme 1A).^7^ In contrast, the sulfur-stabilized derivative, which would permit the construction of the aforementioned *C*-chiral allylic sulfilimines, is a particularly poor nucleophile for the rhodium-catalyzed reaction due to the combination of field and resonance stabilization.^8,9^ Hence, we envisioned that the iridium-catalyzed reaction, which typically facilitates the alkylation of several weak nucleophiles,^10^ should overcome the poor reactivity and thereby provide exciting opportunities to illustrate the unique reactivity of the sulfilimine group. Herein, we now describe the first regio- and enantioselective iridium-catalyzed allylic amination^11^ of allylic carbonates and benzoates **1** with the commercially available nitrogen ylide *S*,*S*-diphenylsulfilimine^12,13^ for the construction of chiral non-racemic *N*-allylic sulfilimines **2**, a hitherto unreported motif of considerable synthetic potential (Scheme 1B).

## Results and discussion

**Fig. 1:**
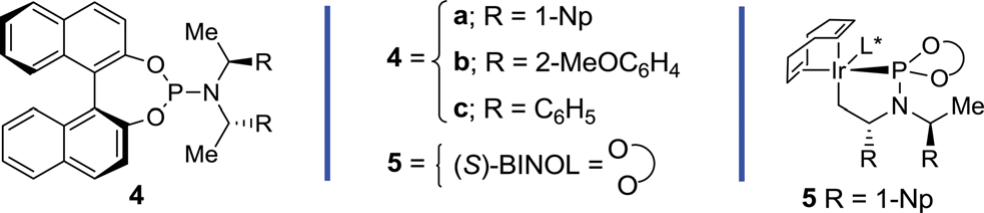
Chiral phosphoramidite ligands and the cyclometallated iridium complex utilized for the enantioselective allylic amination.

Table 1 outlines the preliminary studies on the development of the regio- and enantioselective iridium-catalyzed allylic amination with a sulfilimine nucleophile. Treatment of the cinnamyl carbonate **1a** with *S*,*S*-diphenylsulfilimine and the chiral iridium complex derived from [Ir(cod)Cl]_2_ and the phosphoramidite ligand **4a** ^14^ (Fig. 1) in dichloromethane at 35 °C furnished the sulfilimine **2a** in 79% yield with excellent regio- and enantioselectivity (entry 1). Interestingly, the related phosphoramidite ligands **4b** ^15^ and **4c** ^16^ were significantly inferior to **4a** in terms of both the efficiency and regioselectivity (Fig. 1). Further studies examined the feasibility of utilizing catalytic quantities of cesium carbonate as the exogenous base to improve the overall yield. Gratifyingly, the allylic amination reaction in the presence of catalytic cesium carbonate, furnished the allylic sulfilimine **2a** with improved efficiency and comparable selectivity, making this an attractive synthetic protocol (entry 2).^17^

**Table 1:** Optimization of the regio- and enantioselective iridium-catalyzed allylic amination of aryl and aliphatic allylic electrophiles^*a*^

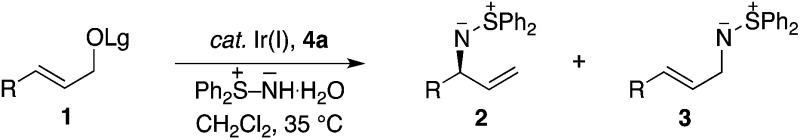							
Entry	Allylic alcohol derivative **1**		p*K*_a_ (LgOH)	Cs_2_CO_3_	Yield (%)^*b*^	**2**/**3** ^*c*^	ee (%)^*d*^ ^,^ ^*e*^
	R =	Lg =					
1	Ph	MeCO_2_	5.61	—	79	≥19 : 1	96
***2***	***Ph***	***MeCO*_*2*_**	***5.61***	***0.25***	***87***	***≥19* : *1***	***97***
3	*n*-Pr	“	“	1.1	43	15 : 1	90
4	“	MeCO	4.76	“	18	14 : 1	99
5	“	PhCO	4.20	“	63	≥19 : 1	93
***6***	***n-Pr***	***3-FC*_*6*_ *H*_*4*_ *CO***	***3.87***	***1.1***	***91***	***≥19* : *1***	***95***
7	“	“	“	0.25	72	≥19 : 1	94

^*a*^All reactions were carried out on a 0.25 mmol reaction scale using 2 mol% of [Ir(cod)Cl]_2_, 4 mol% of **4a** and 1.1 equivalents of *S,S*-diphenylsulfilimine in dichloromethane at 35 °C.

^*b*^Isolated yields.

^*c*^Regioselectivity was determined by 500 MHz ^1^H NMR on the crude reaction mixtures.

^*d*^Enantioselectivity was determined by chiral HPLC analysis of the *N*-trifluoroacetamide or *N-p*-toluenesulfonamide derivative.^19^

^*e*^The absolute configurations of **(*S*)-2a** and **(*R*)-2k** were determined by conversion to either the *N*-trifluoroacetamide or the *N-p*-tosylsulfonamide derivative and comparison of the optical rotations with the reported values.^19,20^

Having established the optimal procedure for the aryl derivatives, we sought to examine aliphatic substituted electrophiles, which are generally more challenging substrates for the iridium-catalyzed allylic substitution reaction.^6f^ In this context, the application of similar reaction conditions to the propyl substituted allylic carbonate **1** led to poor yield with both catalytic and stoichiometric base (entry 3), which prompted the examination of alternative leaving groups. Although the acetate leaving-group was inferior to the carbonate (entry 3 *vs.* 4), the benzoate provided significant improvement in yield and regioselectivity (entry 5). Additional modifications to the stereoelectronics of the benzoate identified the 3-fluorobenzoate as the optimal leaving group (3-FC_6_H_4_CO > PhCO > MeOCO > MeCO) in terms of selectivity and efficiency (entry 6).^18^ Furthermore, this reaction can also be conducted with catalytic base, albeit with slightly diminished yield (entry 7). Hence, the scope of the reaction was examined using stoichiometric cesium carbonate.

Table 2 outlines the application of the optimized reaction conditions (Table 1, entries 2 and 6) to aryl-, alkyl- and alkenyl-substituted allylic carbonates and benzoates.‡ Interestingly, the reaction is tolerant of a wide array of electron-rich and electron-poor cinnamyl alcohol derivatives, in which 2-, 3- and 4-substituted aromatic allylic carbonates afford excellent yields and regioselectivities (entries 1–10), albeit with a slightly diminished selectivity for the chloro- and bromo-derivatives (entries 7 and 8). In addition, excellent enantioselectivities were observed in all cases with the exception of the 2*-*substituted variant (entry 2), which often provides lower selectivity in related iridium-catalyzed allylic substitution reactions.^6*f*^ The 2-naphthyl derivative also provides excellent regio- and enantioselectivity for this process (entry 10), thereby further illustrating the scope with aryl derivatives. Gratifyingly, the application of the optimized reaction conditions to the more challenging alkyl and alkenyl derivatives provided exquisite regio- and enantioselectivity. For instance, the linear and branched alkyl derivatives provide excellent yields and selectivities under these conditions (entries 11–17), albeit the α-branched derivatives required pre-activation of the catalyst (entries 14–16) with *n*-propylamine to provide **5** (Fig. 1).^10*a*^ Additional studies demonstrated that *tert*-butyldimethylsilyl and benzyl protected hydroxymethyl and ethyl derivatives are also well tolerated (entries 18–21). Furthermore, the tethered *N*-Boc and *N*-Cbz derivatives also provide suitable substrates for this process to afford differentially protected diamines (entries 22–23). Finally, the chloro- and alkenyl-substituted allylic benzoates afford excellent selectivity (entry 24–25), in which the latter also requires catalyst pre-activation similar to the α-branched derivatives. Overall, this study highlights the synthetic versatility of the allylic amination reaction with a sulfilimine nucleophile, which encompasses an array of aryl-, alkyl- and alkenyl-substituted allylic alcohol derivatives.

**Table 2:** Scope of the regio- and enantioselective iridium-catalyzed allylic amination of aryl, aliphatic and alkenyl allylic alcohol derivatives (Scheme 1B)^*a*^

Entry	Allylic alcohol derivative **1**			Method	Yield^*b*^ (%)	*b*/*l* **2**/**3** ^*c*^	ee^*d*^ (%)
	Lg =	R =					
1	MeOCO–	Ph	**a**	A	87	≥19 : 1	98^*e*^
2	“	2-MeOC_6_H_4_	**b**	A	87	≥19 : 1	81
3	“	3-MeOC_6_H_4_	**c**	A	85	≥19 : 1	94
4	“	4-MeOC_6_H_4_	**d**	A	80	≥19 : 1	95
5	“	4-MeC_6_H_4_	**e**	A	90	≥19 : 1	93
6	“	4-FC_6_H_4_	**f**	A	88	≥19 : 1	96
7	“	4-ClC_6_H_4_	**g**	A	78	17 : 1	96
8	“	4-BrC_6_H_4_	**h**	A	81	18 : 1	91
9	“	3,5-(MeO)_2_C_6_H_3_	**i**	A	81	≥19 : 1	94
10	“	2-Naphthyl	**j**	A	89	≥19 : 1	96
11	3-FC_6_H_4_CO–	*n*-Pr	**k**	B	91	≥19 : 1	95^*e*^
12	“	Me	**l**	B	91	≥19 : 1	95^*e*^
13	“	Ph(CH_2_)_2_	**m**	B	86	≥19 : 1	91
14	“	*i*-Pr	**n**	C	82	≥19 : 1	95^*e*^
15	“	*c*-Pen	**o**	C	84	≥19 : 1	97
16	“	*c*-Hex	**p**	C	74	≥19 : 1	97
17	“	*i*-Pen	**q**	B	86	≥19 : 1	94
18	“	TBSOCH_2_	**r**	C	78	17 : 1	95
19	“	TBSO(CH_2_)_2_	**s**	C	77	14 : 1	93
20	“	BnOCH_2_	**t**	B	78	16 : 1	92
21	“	BnO(CH_2_)_2_	**u**	B	71	17 : 1	90
22	“	BocNH(CH_2_)_5_	**v**	B	82	≥19 : 1	93
23	“	CbzNH(CH_2_)_5_	**w**	B	82	≥19 : 1	92
24	“	Cl(CH_2_)_5_	**x**	B	82	≥19 : 1	92
25	“	(*E*)-MeCH=CH	**y**	C	72	≥19 : 1	95

^*a*^All reactions were carried out on a 0.25 mmol reaction scale using 2 mol% of [Ir(cod)Cl]_2_, 4 mol% of **4a** and 1.1 equivalents of *S,S*-diphenylsulfilimine in dichloromethane at 35 °C. Method A: 0.25 equiv. Cs_2_CO_3_; Method B: 1.1 equiv. Cs_2_CO_3_; Method C: 1.1 equiv. Cs_2_CO_3_ and catalyst pre-activated with *n*-PrNH_2_.^10*a*^

^*b*^Isolated yields.

^*c*^Regioselectivity was determined by 500 MHz ^1^H NMR on the crude reaction mixtures.

^*d*^Enantioselectivity was determined by chiral HPLC analysis of the *N*-trifluoroacetamides (entries 1–10 and 20–21) and the *N-p*-tosylsulfonamides (entries 11–19 and 22–25).

^*e*^The absolute configurations were determined by conversion to the known *N-p*-tosylsulfonamide and comparison of the optical rotations, which are consistent with the Hartwig model.^19,20^

**Scheme 2:**
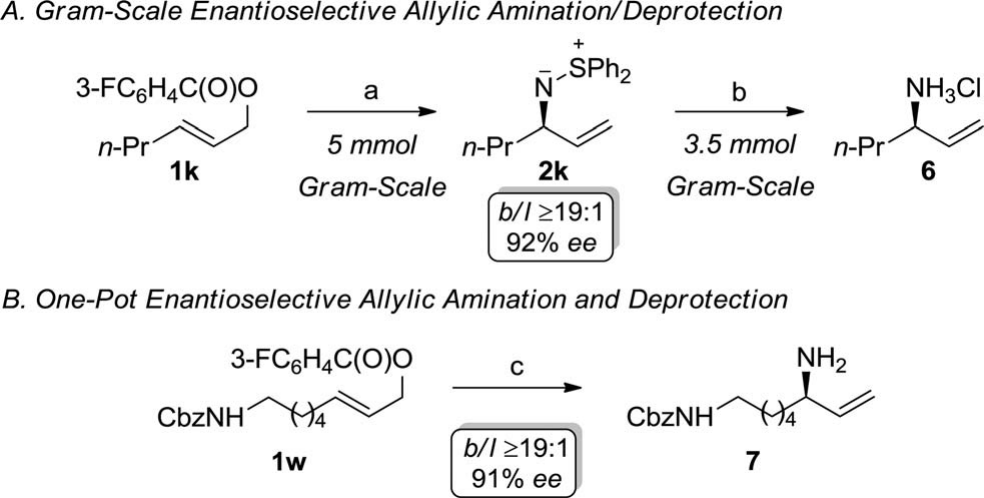
Gram-scale allylic amination/deprotection and one-pot allylic amination/deprotection: (a) *cat*. [Ir(cod)Cl]_2_, **4a**, Ph_2_S^+^NH^–^, Cs_2_CO_3_, CH_2_Cl_2_, 35 °C, 81%; (b) aqueous 10M HCl, CH_3_CN, RT, 94%; c) *cat*. [Ir(cod)Cl]_2_, **4a**, Ph_2_S^+^NH^–^, Cs_2_CO_3_, CH_2_Cl_2_, 35 °C; then aqueous 10M HCl, CH_3_CN, RT, 76%.

Scheme 2 illustrates the synthetic utility of the iridium-catalyzed allylic amination with *S*,*S*-diphenylsulfilimine, which represents a novel ammonia equivalent.^21–23^ The development of ammonia equivalents remains an important area of investigation, since chiral nonracemic primary allylic amines are versatile synthons for target directed synthesis. Recent landmark reports on the ability to utilize ammonia to directly prepare the primary allylic amine represents an important advance in this area. Nevertheless, this approach has limitations, which provides the impetus for further developments. For instance, the allylic amination with ammonia requires a large excess of the nucleophile (100 fold) and a specialized catalyst to reduce dialkylation. Although this process affords exquisite selectivity, the yields of the primary amine are generally modest. Alternatively, the allylic amination with sulfamic acid, which forms ammonia through *in situ* fragmentation, provides modest yields and enantioselectivities with simple alkyl substrates, thereby making this less attractive for synthetic applications. Consequently, we envisioned that the allylic amination with *S*,*S*-diphenylsulfilimine could be combined with a simple deprotection to provide a practical and scalable route to this important functional group.^21^ For example, the allylic amination of **1k** can be accomplished on a gram-scale to afford the enantiomerically enriched sulfilimine **2k** in 81% yield with excellent selectivity. Interestingly, the cleavage of the *N–S* bond could also be accomplished on a similar scale to afford the allylic amine hydrochloride salt **6** in 94% yield (99% *cee*) (Scheme 2A).^24^ Alternatively, enantiomerically enriched primary allylic amines can be accessed directly using the one-pot process outlined in Scheme 2B. Treatment of the primary allylic carbonate **1w** under the optimal reaction conditions, followed by the *in situ* cleavage of the *N–S* bond of the sulfilimine, furnished the mono-protected diamine **7** in 76% overall yield (*b*/*l* ≥19 : 1, 91% *ee*), thereby illustrating the synthetic utility of the sulfilimine.‡Representative procedure: the phosphoramidite ligand **4a** (128 mg, 0.20 mmol) and [Ir(cod)Cl]_2_ (67 mg, 0.10 mmol) were dissolved in anhydrous dichloromethane (7.5 mL) and stirred at room temperature for *ca.* 15 minutes under an atmosphere of argon resulting in a homogeneous orange solution. A suspension of *S*,*S*-diphenylsulfilimine monohydrate (1.21 g, 5.5 mmol) and cesium carbonate (1.72 g, 5.3 mmol) in anhydrous DCM (2.5 mL) was heated in a 35 °C oil bath for *ca.* 15 minutes. The catalyst was added *via* syringe to the nucleophile, followed by the addition of the allylic benzoate **1k** (1.11 g, 5.0 mmol) using a tared 5 mL gas-tight syringe. The resulting reaction mixture was stirred in a 35 °C oil bath for *ca.* 20 hours and then concentrated *in vacuo* to afford the crude product. Purification by flash chromatography (SiO_2_, gradient elution with ethyl acetate/hexanes 70 : 30 followed by ethyl acetate/hexanes/triethylamine 50 : 50 : 0.5) afforded the *allylic sulfilimine* **2k** (1.15 g, 81%, 92% *ee*) as a colorless oil.


## Conclusions

In conclusion, we have devised the first highly regio- and enantioselective iridium-catalyzed allylic amination reaction with the sulfur-stabilized aza-ylide, *S*,*S*-diphenylsulfilimine, which is directly applicable to aryl-, alkyl- and alkenyl-substituted sulfilimines. Additionally, the sulfilimine provides a convenient and commercially available ammonia equivalent, which is readily cleaved with acid to afford the enantiomerically enriched primary amine hydrochloride salt. Furthermore, the ability to conduct this sequence either on a gram-scale or in one-pot, permits the direct formation of the primary allylic amine to illustrate the utility of this process for challenging synthetic applications. Overall, this work demonstrates that aza-ylides are a valuable class of nucleophiles for enantioselective metal-catalyzed allylic substitution reactions.^25^

## References

[cit1] Koval I. V. (1993). Sulfur Rep..

[cit2] Clark D. A., De Riccardis F., Nicolaou K. C. (1994). Tetrahedron.

[cit3] Hayashi Y., Swern D. (1973). J. Am. Chem. Soc..

[cit4] (a) Credner H. H., Ger. Patent, 3215834, 1983.

[cit5] Vanacore R., Ham A.-J. L., Voehler M., Sanders C. R., Conrads T. P., Veenstra T. D., Sharpless K. B., Dawson P. E., Hudson B. G. (2009). Science.

[cit6] (a) Leahy D. K. and Evans P. A., in Modern Rhodium Catalyzed Reactions, ed. P. A. Evans, Wiley-VCH, Weinheim, 2005; ch. 10, p. 191.

[cit7] Evans P. A., Clizbe E. A. (2009). J. Am. Chem. Soc..

[cit8] The rhodium-catalyzed allylic amination with *S*,*S*-diphenylsulfilimine provides the allylic sulfilimine in poor yield (8%)

[cit9] Evans P. A., Robinson J. E., Nelson J. D. (1999). J. Am. Chem. Soc..

[cit10] Shu C., Leitner A., Hartwig J. F. (2004). Angew. Chem., Int. Ed..

[cit11] (i) Ye K.-Y. Dai L.-X. You S.-L., Chem.–Eur. J., 2014, 20 , 3040 , and pertinent references cited therein .24677302

[cit12] Sulfilimines have a coordinate covalent (dative) single bond with strong polarization towards the nitrogen, thereby making them sufficiently nucleophilic to promote amination, see: Pichierri F., Chem. Phys. Lett., 2010, 487 , 315 .

[cit13] *S*,*S*-Diphenylsulfilimine is stable at ambient temperature, whereas many dialkyl sulfilimines decompose above –30 °C, see: Appel R. Büchner W., Chem. Ber., 1962, 95 , 855 .

[cit14] Naasz R., Arnold L. A., Minnaard A. J., Feringa B. L. (2001). Angew. Chem., Int. Ed..

[cit15] Tissot-Croset K., Polet D., Alexakis A. (2004). Angew. Chem., Int. Ed..

[cit16] Sewald N., Wendisch V. (1998). Tetrahedron: Asymmetry.

[cit17] Enhanced reactivity of sulfilimines in the presence of base is well precedented, see: Claridge R. P. Millar R. W. Sandall J. P. B. Thompson C., Tetrahedron, 1999, 55 , 10243 .

[cit18] Interestingly, the 3-fluorobenzoate leaving group provided sub-optimal results with an aryl-substituted electrophile: the reaction of **1** (R = Ph, LG = 3-FC_6_H_4_CO) furnished **2a** in 59% yield and with 97% enantiomeric excess

[cit19] For the conversion of sulfilimines to *N*-trifluoroacetamides or *N*-tosylsulfonamides, see: Drabowicz J. Łyzwa P. Mikołajczyk M., Synthesis, 1981 , 890 .

[cit20] Madrahimov S. T., Markovic D., Hartwig J. F. (2009). J. Am. Chem. Soc..

[cit21] Pouy M. J., Stanley L. M., Hartwig J. F. (2009). J. Am. Chem. Soc..

[cit22] For enantio-convergent iridium-catalyzed allylic substitution using *in-situ* generated ammonia as the pro-nucleophile, see: Lafrance M. Roggen M. Carreira E. M., Angew. Chem., Int. Ed., 2012, 51 , 3470 . 10.1002/anie.201108287 22344968

[cit23] *S,S*-Diphenylsulfilimine has also been utilized as an ammonia equivalent in a 1,6-conjugate addition.2*b*

[cit24] Shine H. J., Kim K. (1974). Tetrahedron Lett..

[cit25] Enantiomerically enriched allylic sulfilimines readily undergo a novel metathesis reaction to afford chiral-nonracemic allylic isocyanates, see: Grange R. L. Evans P. A., J. Am. Chem. Soc., 2014, 136 , 11870 . 25109231 10.1021/ja504631v

