# Supplementary material for: Enantioselective construction of C-chiral allylic sulfilimines via the iridium-catalyzed allylic amination with S,S-diphenylsulfilimine: asymmetric synthesis of primary allylic amines
Source: Chem Sci. 2014 Sep 8;6(1):777–81. doi: 10.1039/c4sc01317d (PMC5592745; doi:10.1039/c4sc01317d)
Supplement: Supplementary file 1 [file SC-006-C4SC01317D-s001.pdf]

Electronic Supplementary Material (ESI) for Chemical Science.
This journal is © The Royal Society of Chemistry 2014

Enantioselective construction of *C*-chiral allylic sulfilimines *via* the iridium-catalyzed allylic amination with *S,S*-diphenylsulfilimine: asymmetric synthesis of primary allylic amines

Rebecca L. Grange,[a] Elizabeth A. Clizbe,[b] Emma J. Counsell[b] and P. Andrew Evans*[a]

[a] *Department of Chemistry, Queen's University, Kingston, Ontario, K7L 3N6, Canada*

[b] *Department of Chemistry, University of Liverpool, Liverpool, L69 7ZD, United Kingdom*

Contents of Supporting Information:

1. General Information S1
2. Representative Experimental Procedures S2
3. Spectral Data for the Allylic Carbonates, Benzoates and Sulfilimines S4
4. Experimental Procedures and Spectral Data for Hydrochloride Salt **6** and Amine **7** S25
5. Copies of NMR Spectra S27

1. General Information

All reactions were carried out in flame-dried glassware under an atmosphere of argon or nitrogen using anhydrous solvent. Dichloromethane was dried by passage through activated alumina columns. *n*-Propylamine was distilled from activated zinc dust and stored over sodium hydroxide. All other reagents were purchased from Acros, Aldrich, Alfa Aesar or Strem and used as received. Thin layer chromatography (TLC) was performed using Whatman F_{254} or EMD Chemicals Inc. 60 F_{254} precoated silica gel plates. Visualisation was accomplished with a UV light, ceric ammonium nitrate solution or ninhydrin. Flash chromatography was performed using Merck Silica Gel 60 (230-400 mesh), Whatman Silica Gel Purasil® 60Å (230-400 mesh) or EMD Silica Gel 60 (0.04 – 0063 mm particle size). The melting points were obtained from a *Buchi Melting Point Apparatus*. Solvents for extraction and flash chromatography were reagent grade.

Infrared spectra were obtained on a Perkin-Elmer spectrum 100 series or an Agilent Technologies Cary 630 FTIR spectrometer. Peaks are reported in cm^{-1} with the following relative intensities: vs (very strong), s (strong), m (medium) and w (weak). Mass spectra were performed at the University of Liverpool Mass Spectrometry Center, EPSRC National Mass Spectrometry Service Centre or Queen's University Mass Spectrometry and Proteomics Services Unit. High-resolution electron-impact (EI, ionization voltages of 70 eV) and chemical ionization mass spectra (CI, reagent gas CH_4 or NH_3) were obtained on either a Micromass 70-250S double focusing mass spectrometer or an Autospec, ZAB 2SE, Kratos MS-80, VG 7070E double focusing magnetic sector mass spectrometer equipped with a solid probe inlet, a Quattro II, MAT 95, or a MAT 900. The electrospray ionization (ESI) mass spectra were obtained on a Waters micromass LCT mass spectrometer. ^{1}H

and ^{13}C NMR were recorded on a Bruker AV 500 MHz NMR spectrometer in $CDCl_3$, which was obtained from Cambridge Isotope Labs or Sigma Aldrich. NMR data were calibrated using the signal of residual undeuterated solvent as an internal reference ($CHCl_3$, δ_H = 7.26 ppm, δ_C = 77.16 ppm; C_6D_6, δ_H = 7.16 ppm, δ_C = 128.39 ppm; CD_3OD, δ_C = 49.15 ppm). ^{1}H NMR data are reported as follows: chemical shift (multiplicity, 1st order spin system if available, coupling constant and integration). Coupling constants (*J*) are reported in Hz and apparent splitting patterns are designated using the following abbreviations: s (singlet), d (doublet), t (triplet), q (quartet), quintet, sextet, m (multiplet), br. (broad), app. (apparent) and combinations thereof. ^{13}C NMR spectra with complete proton decoupling were described with the aid of an APT sequence, separating methylene and quaternary carbons (e, even), from methyl and methine carbons (o, odd).

All liquid chromatographs were obtained on an Agilent 1200 series or an Agilent 1260 Infinity HPLC equipped with a variable wavelength UV detector. The instrument was fitted with a CHIRALPAK™ AD-H column (Diacel, 4.6mm x 25cm), CHIRALCEL™ OD column (Diacel, 4.6mm x 25cm), CHIRALCEL™ OJ column (Diacel, 4.6mm x 25cm) or a CHIRALPAK™ AS-H column (Diacel, 4.6mm x 25cm).

2. Representative Experimental Procedures

Representative Procedure for the Preparation of Allylic Carbonates and Benzoates: The primary allylic alcohol (3.1 mmol) was dissolved in anhydrous dichloromethane (10 mL) and cooled with stirring to 0 °C under an atmosphere of nitrogen. 4-Dimethylaminopyridine (452 mg, 3.7 mmol) was added and the resulting reaction mixture stirred for *ca*. 15 minutes prior to the addition of the acylating agent (methyl chloroformate or 3-fluorobenzoyl chloride) (2.8 mmol) *via* syringe. The reaction mixture was then warmed to ambient temperature and stirred for *ca*. 16 hours, before being cooled to 0 °C, quenched with water and partitioned with dichloromethane. The organic phases were combined, washed with saturated aqueous NaCl solution, dried (anhyd. Na_2SO_4), filtered and concentrated *in vacuo* to afford the crude product. Purification by flash chromatography (SiO_2, eluting with ethyl acetate/hexanes) afforded carbonates **1a-j** and benzoates **1k-y**.

Representative Procedure for the Iridium-Catalyzed Allylic Amination Reaction with Aryl Substrates (Method A): The phosphoramidite ligand **4a** (6.4 mg, 0.01 mmol) and $[Ir(cod)Cl]_2$ (3.4 mg, 0.005 mmol) were dissolved in anhydrous dichloromethane (0.5 mL) and stirred at room temperature for *ca*. 15 minutes under an atmosphere of argon resulting in a homogeneous orange solution. A suspension of *S,S*-diphenylsulfilimine monohydrate[1] (60.4 mg, 0.28 mmol) and cesium carbonate (20.4 mg, 0.06 mmol) in anhydrous dichloromethane (0.25 mL) was heated in a 35 °C oil

[1] Available from Aldrich (catalog no. 232173).

bath for *ca*. 15 minutes. The catalyst was then added *via* syringe to the nucleophile, followed by the addition of the allylic carbonate **1** (0.25 mmol) using a tared 500 μL gas-tight syringe. The resulting reaction mixture was stirred in a 35 °C oil bath for *ca*. 6 hours and then concentrated *in vacuo* to afford the crude product. Purification by flash chromatography (SiO_2, gradient elution with 70:30:0 to 70:30:0.5 ethyl acetate/hexanes/triethylamine) afforded the *allylic sulfilimines* **2a-j**.

Representative Procedure for the Derivatization to the N-Trifluoroacetamide for the Determination of the Enantioselectivity:[2] Sodium iodide (20.0 mg, 132 μmol) and trifluoroacetic anhydride (19.0 μL, 132 μmol) were added to the allylic sulfilimine **2** (66 μmol) in acetone (750 μL) at 0 °C and the reaction mixture stirred for *ca*. 30 minutes. The reaction was then allowed to warm to ambient temperature and stirred for an additional *ca*. 30 minutes, quenched by the addition of 10% aqueous Na_2CO_3 solution and partitioned with dichloromethane. The organic phases were combined, washed with 10% aqueous Na_2CO_3 solution, dried (anhyd. Na_2SO_4), filtered and concentrated *in vacuo* to afford the crude product. Purification by flash chromatography (SiO_2, gradient elution with 99:1 to 95:5 ethyl acetate/hexanes) furnished the *N*-trifluoroacetyl derivatives suitable for chiral HPLC analysis.

Representative Procedure for the Iridium-Catalyzed Allylic Amination Reaction with Alkyl Substrates Using the In-situ Activated Catalyst (Method B): The phosphoramidite ligand **4a** (6.4 mg, 0.01 mmol) and $[Ir(cod)Cl]_2$ (3.4 mg, 0.005 mmol) were dissolved in anhydrous dichloromethane (0.5 mL) and stirred at room temperature for *ca*. 15 minutes under an atmosphere of argon resulting in a homogeneous orange solution. A suspension of *S,S*-diphenylsulfilimine monohydrate (60.4 mg, 0.28 mmol) and cesium carbonate (85.5 mg, 0.263 mmol) in anhydrous dichloromethane (0.25 mL) was heated in a 35 °C oil bath for *ca*. 15 minutes. The catalyst was then added *via* syringe to the nucleophile, followed by the addition of the allylic benzoate **1** (0.25 mmol) using a tared 500 μL gas-tight syringe. The resulting reaction mixture was stirred in a 35 °C oil bath for *ca*. 6 hours and then concentrated *in vacuo* to afford the crude product. Purification by flash chromatography (SiO_2, gradient elution with 70:30:0 to 50:50:0.5 ethyl acetate/hexanes/triethylamine) furnished the *allylic sulfilimines* **2k-m**, **q** and **t-x**.

Representative Procedure for the Iridium-Catalyzed Allylic Amination Reaction Using the Pre-Activated Catalyst (Method C): The phosphoramidite ligand **4a** (6.4 mg, 0.01 mmol) and $[Ir(cod)Cl]_2$ (3.4 mg, 0.005 mmol) were dissolved with stirring in anhydrous THF (0.1 mL) at room temperature under an atmosphere of argon. *n*-Propylamine (0.1 mL) was added and the resulting mixture was stirred in a 50 °C oil bath for *ca*. 30 minutes resulting in a homogeneous yellow

[2] J. Drabowicz, P. Łyzwa, M. Mikołajczyk, *Synthesis* **1981**, 890-891.

solution. The reaction mixture was evaporated *in vacuo* using a high vacuum pump and the residue was redissolved in anhydrous dichloromethane (300 μL). The catalyst was then added to a mixture of the *S,S*-diphenylsulfilimine monohydrate (60.4 mg, 0.28 mmol) and cesium carbonate (86.0 mg, 0.26 mmol), followed by the addition of the allylic benzoate **1** (0.25 mmol) using a tared 500 μL gas-tight syringe. The resulting reaction mixture was stirred in a 35 °C oil bath for *ca*. 6 hours and then concentrated *in vacuo* to afford the crude product. Purification by flash chromatography (SiO_2, gradient elution with 70:30:0 to 50:50:0.5 hexanes/ethyl acetate/triethylamine) furnished the *allylic sulfilimines* **2n** – **p**, **r**, **s** and **y**.

Representative Procedure for the Derivatization to the p-Toluenesulfonamide for the Determination of the Enantioselectivity (unless otherwise indicated):[2] The procedure is analogous to that utilized to prepare the *N*-trifluoroacetamide, albeit with *p*-toluenesulfonyl chloride as the electrophile.

3. Spectral Data for the Allylic Carbonates, Benzoates and Sulfilimines

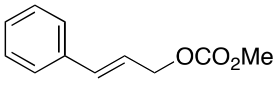

Methyl (*E*)-3-phenylprop-2-en-1-yl carbonate (1a).[3]

Color and State: Colorless oil.

¹H NMR (500 MHz, $CDCl_3$) δ 7.43-7.40 (m, 2H), 7.39-7.33 (m, 2H), 7.31-7.27 (m, 1H), 6.72 (d, *J* = 15.9 Hz, 1H), 6.32 (dt, *J* = 15.9, 6.4 Hz, 1H), 4.82 (dd, *J* = 6.5, 1.3 Hz, 2H), 3.83 (s, 3H).

IR 3028 (w), 2957 (w), 1742 (vs), 1441 (m), 1251 (vs), 943 (s), 790 (s), 746 (s) cm^{-1}.

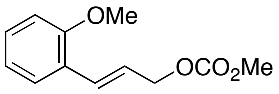

(*E*)-3-(2-Methoxyphenyl)prop-2-en-1-yl methyl carbonate (1b).[4]

Color and State: Colorless oil.

¹H NMR (500 MHz, $CDCl_3$) δ 7.43 (dd, *J* = 7.7, 1.6 Hz, 1H), 7.26-7.23 (m, 1H), 7.02 (d, *J* = 16.0 Hz, 1H), 6.92 (app. td, *J* = 7.5, 0.5 Hz, 1H), 6.87 (d, *J* = 8.2 Hz, 1H), 6.33 (dt, *J* = 16.0, 6.6 Hz, 1H), 4.80 (dd, *J* = 6.6, 1.3 Hz, 2H), 3.84 (s, 3H), 3.80 (s, 3H).

IR (neat) 3004 (w), 2956 (w), 1744 (vs), 1598 (m), 1244 (vs), 1177 (m), 1027 (m), 945 (s), 753 (s) cm^{-1}.

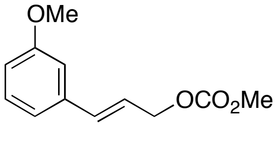

(*E*)-3-(3-Methoxyphenyl)prop-2-en-1-yl methyl carbonate (1c).[5]

Color and State: Light yellow oil.

¹H NMR (500 MHz, $CDCl_3$) δ 7.29-7.25 (m, 1H), 7.01 (d, *J* = 7.6 Hz, 1H), 6.96-6.95 (m, 1H), 6.85 (dd, *J* = 8.1, 2.1 Hz, 1H), 6.68 (d, *J* = 15.9 Hz, 1H), 6.32 (dt, *J* = 15.9, 6.4 Hz, 1H), 4.81 (dd, *J* = 6.4, 1.3 Hz, 2H), 3.83 (s, 6H).

---

[3] J. Lehman, G. C. Lloyd-Jones, *Tetrahedron* **1995**, *51*, 8863–8874.
[4] T. Ohmura, J. F. Hartwig, *J. Am. Chem. Soc.* **2002**, *124*, 15164-15165.
[5] Z. Hu, Y. Li, K. Liu, Q. Shen, *J. Org. Chem.* **2012**, *77*, 7957-7967.

IR (neat) 3007 (w), 2948 (w), 1737 (vs), 1573 (s), 1432 (m), 1243 (vs), 1145 (s), 1032 (s), 931 (vs) cm^{-1}.

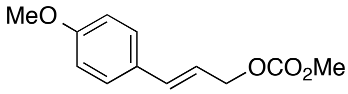

(*E*)-3-(4-Methoxyphenyl)prop-2-en-1-yl methyl carbonate (1d).[6]
Color and State: White solid; **mp** = 88-90 °C (lit.[6] mp = 81-83 °C).
^{1}H NMR (500 MHz, $CDCl_3$) δ 7.35-7.32 (m, 2H), 6.87-6.85 (m, 2H), 6.64 (d, *J* = 15.8 Hz, 1H), 6.16 (dt, *J* = 15.8, 6.6 Hz, 1H), 4.77 (dd, *J* = 6.6, 1.1 Hz, 2H), 3.81 (s, 3H), 3.80 (s, 3H).
IR (neat) 3014 (w), 2958 (w), 1733 (vs), 1601 (m), 1506 (s), 1435 (s), 1262 (vs), 1241 (vs), 1171 (s), 1027 (vs), 951 (vs), 839 (vs) cm^{-1}.

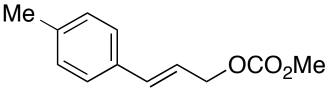

(*E*)-3-(4-Methylphenyl)prop-2-en-1-yl methyl carbonate (1e).[3]
Color and State: White solid; **mp** = 54-56 °C (lit.[3] mp = 47-48 °C).
^{1}H NMR (500 MHz, $CDCl_3$) δ 7.29 (d, *J* = 8.0 Hz, 2H), 7.14 (d, *J* = 7.9 Hz, 2H), 6.66 (d, *J* = 15.9 Hz, 1H), 6.25 (dt, *J* = 15.9, 6.6 Hz, 1H), 4.78 (dd, *J* = 6.6, 1.1 Hz, 2H), 3.81 (s, 3H), 2.34 (s, 3H).
IR (neat) 3001 (w), 2955 (w), 1736 (vs), 1442 (m), 1257 (vs), 948 (vs), 802 (s), 786 (vs) cm^{-1}.

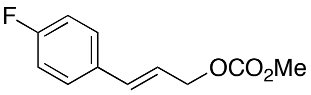

(*E*)-3-(4-Fluorophenyl)prop-2-en-1-yl methyl carbonate (1f).[6]
Color and State: White solid; **mp** = 25-26 °C.
^{1}H NMR (500 MHz, $CDCl_3$) δ 7.38-7.34 (m, 2H), 7.04-6.99 (m, 2H), 6.65 (d, *J* = 15.9 Hz, 1H), 6.21 (dt, *J* = 15.9, 6.5 Hz, 1H), 4.78 (dd, *J* = 6.5, 1.2 Hz, 2H), 3.81 (s, 3H).
IR (neat) 3010 (w), 2959 (w), 1735 (vs), 1265 (vs), 1233 (vs), 948 (vs), 930 (s), 856 (vs) cm^{-1}.

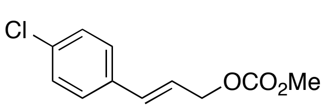

(*E*)-3-(4-Chlorophenyl)prop-2-en-1-yl methyl carbonate (1g).[3]
Color and State: White solid; **mp** = 44-46 °C (lit.[3] mp = 46-47 °C)
^{1}H NMR (500 MHz, $CDCl_3$) δ 7.35-7.29 (m, 4H), 6.67 (d, *J* = 15.9 Hz, 1H), 6.29 (dt, *J* = 15.9, 6.4 Hz, 1H), 4.81 (dd, *J* = 6.3, 1.3 Hz, 2H), 3.84 (s, 3H).
IR (neat) 2958 (w), 1747 (s), 1257 (vs), 1090 (s), 1013 (m), 941 (s), 821 (m) cm^{-1}.

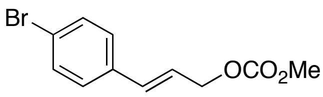

(*E*)-3-(4-Bromophenyl)prop-2-en-1-yl methyl carbonate (1h).[7]
Color and State: White solid; **mp** = 68-69 °C (lit.[7] mp = 74-75 °C).
^{1}H NMR (500 MHz, $CDCl_3$) δ 7.45-7.42 (m, 2H), 7.26-7.23 (m, 2H), 6.61 (d, *J* = 15.9 Hz, 1H), 6.28 (dt, *J* = 15.9, 6.4 Hz, 1H), 4.77 (dd, *J* = 6.4, 1.3 Hz, 2H), 3.80 (s, 3H).

---

[6] M. Ohkoshi, J. Michinishi, S. Hara, H. Senboku *Tetrahedron* **2010**, *66*, 7732-7737.
[7] B. M. Trost, J. R. Miller, C. M. Hoffman Jr, *J. Am. Chem. Soc.* **2011**, *133*, 8165-8167.

IR (neat) 2960 (w), 1741 (vs), 1260 (vs), 1076 (s), 1010 (m), 956 (vs), 834 (s), 789 (vs) cm^{-1}.

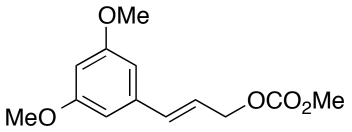

(*E*)-3-(3,5-Dimethoxyphenyl)prop-2-en-1-yl methyl carbonate (1i).

Color and State: White solid; **mp** = 46-47 °C.

^{1}H NMR (500 MHz, $CDCl_3$) δ 6.62 (d, *J* = 15.8 Hz, 1H), 6.54 (d, *J* = 2.2 Hz, 2H), 6.39 (t, *J* = 2.2 Hz, 1H), 6.28 (dt, *J* = 15.8, 6.4 Hz, 1H), 4.78 (d, *J* = 6.4 Hz, 2H), 3.81 (s, 3H), 3.79 (s, 6H).

^{13}C NMR (125 MHz, $CDCl_3$) δ 160.96 (e), 155.71 (e), 138.08 (e), 134.70 (o), 123.04 (o), 104.75 (o), 100.52 (o), 68.32 (e), 55.39 (o), 54.93 (o).

IR (neat) 2961 (w), 1741 (vs), 1588 (m), 1443 (s), 1259 (vs), 956 (vs), 789 (s) cm^{-1}.

HRMS (ESI, $[M+H]^+$) calcd for $C_{13}H_{17}O_5$ 253.1071, found 253.1074.

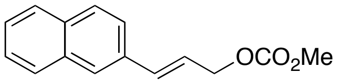

Methyl (*E*)-3-(2-naphthyl)prop-2-en-1-yl carbonate (1j).[8]

Color and State: White solid; **mp** = 102-104 °C.

^{1}H NMR (500 MHz, $CDCl_3$) δ 7.79-7.77 (m, 3H), 7.75 (s, 1H), 7.59 (dd, *J* = 8.6, 1.5 Hz, 1H), 7.46-7.43 (m, 2H), 6.84 (d, *J* = 15.9 Hz, 1H), 6.41 (dt, *J* = 15.9, 6.5 Hz, 1H), 4.84 (d, *J* = 6.5 Hz, 2H), 3.81 (s, 3H).

IR (neat) 2952 (w), 1736 (vs), 1266 (vs), 965 (m), 944 (m), 792 (vs), 746 (s) cm^{-1}.

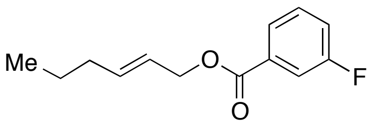

(*E*)-Hex-2-en-1-yl 3-fluorobenzoate (1k).

Color and State: Colorless oil.

^{1}H NMR (500 MHz, $CDCl_3$) δ 7.84 (app. dt, *J* = 7.8, 1.2 Hz, 1H), 7.73 (ddd, *J* = 9.4, 2.6, 1.5 Hz, 1H), 7.41 (app. td, *J* = 8.0, 5.6 Hz, 1H), 7.25 (app. tdd, *J* = 8.3, 2.7, 0.9 Hz, 1H), 5.86 (dt, *J* = 15.2, 6.9 Hz, 1H), 5.68 (dtt, *J* = 15.3, 6.5, 1.4 Hz, 1H), 4.77 (dd, *J* = 6.5, 0.9 Hz, 2H), 2.07 (app. q, *J* = 6.9 Hz, 2H), 1.44 (sextet, *J* = 7.4 Hz, 2H), 0.92 (t, *J* = 7.4 Hz, 3H).

^{13}C NMR (125 MHz, $CDCl_3$) δ 165.29 (e, d, $^4J_{CF}$ = 3.1 Hz), 162.62 (e, d, $^1J_{CF}$ = 246.9 Hz), 136.84 (o), 132.71 (e, d, $^3J_{CF}$ = 7.4 Hz), 129.99 (o, d, $^3J_{CF}$ = 7.9 Hz), 125.40 (o, $^4J_{CF}$ = 2.9 Hz), 123.80 (o), 119.92 (o, d, $^2J_{CF}$ = 21.3 Hz), 116.55 (o, d, $^2J_{CF}$ = 23.0 Hz), 66.15 (e), 34.40 (e) 22.10 (e), 13.68 (o).

IR (neat) 2960 (w), 2933 (w), 2874 (w), 1721 (vs), 1593 (m), 1268 (vs), 1197 (vs), 1093 (m), 951 (s), 894 (m), 807 (m), 753 (vs) cm^{-1}.

HRMS (ESI, $[M+Na]^+$) calcd for $C_{13}H_{15}O_2FNa$ 245.0954, found 245.0951.

[8] J. F. Teichert, B. L. Feringa *Synthesis* **2010**, 1200-1204.

(*E*)-But-2-en-1-yl 3-fluorobenzoate (1l).

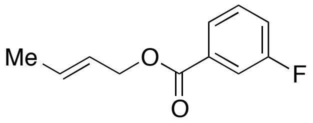

Color and State: Colorless oil.

^{1}H NMR (500 MHz, $CDCl_3$) δ 7.84 (app. dt, J = 7.7, 1.1 Hz, 1H), 7.73 (ddd, J = 9.4, 2.6, 1.5 Hz, 1H), 7.41 (app. td, J = 8.0, 5.6 Hz, 1H), 7.25 (app. tdd, J = 8.3, 2.6, 0.9 Hz, 1H), 5.88 (dqt, J = 15.4, 6.5, 1.1 Hz, 1H), 5.70 (dtq, J = 15.1, 6.5, 1.6 Hz, 1H), 4.75 (dt, J = 6.6, 1.0 Hz, 2H), 1.77-1.75 (m, 3H).

^{13}C NMR (125 MHz, $CDCl_3$) δ 165.37 (e, d, $^4J_{CF}$ = 2.6 Hz), 162.62 (e, d, $^1J_{CF}$ = 246.7 Hz), 132.65 (e, d, $^3J_{CF}$ = 7.4 Hz), 131.91 (o), 130.04 (o, d, $^3J_{CF}$ = 7.9 Hz), 125.43 (o, d, $^4J_{CF}$ = 3.0 Hz), 124.96 (o), 120.02 (o, d, $^2J_{CF}$ = 21.2 Hz), 116.59 (o, d, $^2J_{CF}$ = 23.0 Hz), 66.11 (e), 17.92 (o).

IR (neat) 2945 (w), 2920 (w), 1720 (vs), 1593 (s), 1270 (vs), 1199 (s), 1094 (s), 964 (s), 892 (m), 805 (m), 756 (s) cm^{-1}.

HRMS (CI, $[M+NH_4]^+$) calcd for $C_{11}H_{15}NO_2F$ 212.1081, found 212.1078.

(*E*)-5-Phenylpent-2-en-1-yl 3-fluorobenzoate (1m).

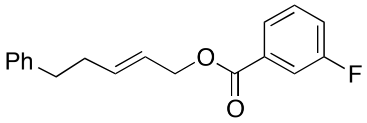

Color and State: Colorless oil.

^{1}H NMR (500 MHz, $CDCl_3$) δ 7.89 (app. dt, J = 7.7, 1.2 Hz, 1H), 7.78 (ddd, J = 9.3, 2.5, 1.5, 1H), 7.43 (app. td, J = 8.0, 5.6 Hz, 1H), 7.33-7.30 (m, 2H), 7.28 (app. tdd, J = 8.4, 2.7, 0.9 Hz, 1H), 7.24-7.22 (m, 3H), 5.95 (dt, J = 15.3, 6.7 Hz, 1H), 5.75 (dtt, J = 15.4, 6.4, 1.3 Hz, 1H), 4.81 (dd, J = 6.4, 0.8 Hz, 2H), 2.78 (t, J = 7.8 Hz, 2H), 2.46 (app. q, J = 7.1 Hz, 2H).

^{13}C NMR (125 MHz, $CDCl_3$) δ 165.22 (e, d, $^4J_{CF}$ = 3.0 Hz), 162.60 (e, d, $^1J_{CF}$ = 247.2 Hz), 141.49 (e), 135.76 (o), 132.64 (e, d, $^3J_{CF}$ = 7.4 Hz), 130.00 (o, d, $^3J_{CF}$ = 8.0 Hz), 128.49 (o), 128.39 (o), 125.99 (o), 125.41 (o, d, $^4J_{CF}$ = 2.9 Hz), 124.38 (o), 119.96 (o, d, $^2J_{CF}$ = 21.2 Hz), 116.56 (o, d, $^2J_{CF}$ = 23.0 Hz), 65.88 (e), 35.34 (e), 34.08 (e).

IR (neat) 2933 (w), 2856 (w), 1720 (vs), 1592 (m), 1269 (vs), 1199 (s), 1093 (m), 955 (s), 892 (w), 806 (w), 755 (s) cm^{-1}.

HRMS (EI, M^+) calcd for $C_{18}H_{17}O_2F$ 284.1213, found 284.1219.

(*E*)-4-Methylpent-2-en-1-yl 3-fluorobenzoate (1n).

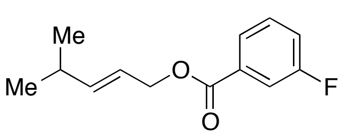

Color and State: Colorless oil.

^{1}H NMR (500 MHz, $CDCl_3$) δ 7.83 (app. dt, J = 7.8, 1.2 Hz, 1H), 7.72 (ddd, J = 9.3, 2.5, 1.5 Hz, 1H), 7.39 (app. td, J = 8.0, 5.6 Hz, 1H), 7.23 (app. tdd, J = 8.3, 2.6, 0.7 Hz, 1H), 5.82 (dd, J = 15.5, 6.4 Hz, 1H), 5.62 (dtd, J = 15.4, 6.5, 1.3 Hz, 1H), 4.76 (d, J = 6.5 Hz, 2H), 2.33 (octet, J = 6.6 Hz, 1H), 1.01 (d, J = 6.8 Hz, 6H).

^{13}C NMR (125 MHz, $CDCl_3$) δ 165.33 (e, d, $^4J_{CF}$ = 2.9 Hz), 162.64 (e, d, $^1J_{CF}$ = 247.1 Hz), 143.69 (o), 132.75 (e, d, $^3J_{CF}$ = 7.4 Hz), 130.01 (o, d, $^3J_{CF}$ = 7.6 Hz), 125.44 (o, d, $^4J_{CF}$ = 3.2 Hz), 120.83 (o), 119.95 (o, d, $^2J_{CF}$ = 21.3 Hz), 116.60 (o, d, $^2J_{CF}$ = 23.0 Hz), 66.29 (e), 30.91 (o), 22.08 (o).

IR (neat) 2961 (m), 2872 (w), 1720 (vs), 1592 (s), 1269 (vs), 1198 (vs), 1093 (s), 970 (s), 893 (m), 805 (m), 755 (s) cm^{-1}.

HRMS (EI, M^+) calcd for $C_{13}H_{15}O_2F$ 221.1056, found 221.1061.

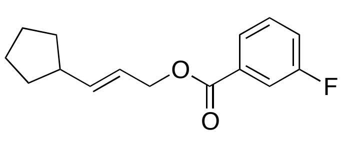

(*E*)-3-Cyclopentylallyl 3-fluorobenzoate (1o).

Color and State: Colorless oil.

^{1}H NMR (500 MHz, $CDCl_3$) δ 7.85 (app. dt, *J* = 7.8, 1.2 Hz, 1H), 7.73 (ddd, *J* = 9.3, 2.6, 1.5 Hz, 1H), 7.41 (app. td, *J* = 8.0, 5.6 Hz, 1H), 7.25 (app. tdd, *J* = 8.3, 2.7, 0.9 Hz, 1H), 5.84 (dd, *J* = 15.3, 7.6 Hz, 1H), 5.65 (dtd, *J* = 15.3, 6.5, 1.0 Hz, 1H), 4.76 (d, *J* = 6.5 Hz, 2H), 2.49 (sextet, *J* = 8.0 Hz, 1H), 1.84-1.78 (m, 2H), 1.71-1.62 (m, 2H), 1.61-1.55 (m, 2H), 1.36-1.29 (m, 2H).

^{13}C NMR (125 MHz, $CDCl_3$) δ 165.39 (e, d, $^4J_{CF}$ = 2.9 Hz), 162.65 (e, d, $^1J_{CF}$ = 246.7 Hz), 141.53 (o), 132.76 (e, d, $^3J_{CF}$ = 7.3 Hz), 130.04 (o, d, $^3J_{CF}$ = 7.7 Hz), 125.46 (o, d, $^4J_{CF}$ = 2.9 Hz), 121.75 (o), 119.99 (o, d, $^2J_{CF}$ = 21.2), 116.63 (o, d, $^2J_{CF}$ = 23.0), 66.31 (e), 43.07 (o), 32.91 (e), 25.26 (e).

IR (neat) 2949 (m), 2867 (w), 1719 (s), 1591 (m), 1267 (vs), 1196 (vs), 1090 (s), 951 (s), 890 (m), 804 (m), 752 (vs) cm^{-1}.

HRMS (EI, M^+) calcd for $C_{15}H_{17}O_2F$ 248.1213, found 248.1222.

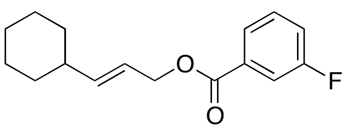

(*E*)-3-Cyclohexylallyl 3-fluorobenzoate (1p).

Color and State: Colorless oil.

^{1}H NMR (500 MHz, $CDCl_3$) δ 7.85 (app. dt, *J* = 7.7, 1.1 Hz, 1H), 7.73 (ddd, *J* = 9.4, 2.6, 1.5 Hz, 1H), 7.41 (app. td, *J* = 8.0, 5.6 Hz, 1H), 7.25 (app. tdd, *J* = 8.3, 2.7, 0.9 Hz, 1H), 5.81 (dd, *J* = 15.5, 6.5 Hz, 1H), 5.62 (dtd, *J* = 15.5, 6.5, 1.2 Hz, 1H), 4.76 (d, *J* = 6.4 Hz, 2H), 2.01 (tdt, *J* = 10.9, 7.2, 3.6 Hz, 1H), 1.77-1.71 (m, 4H), 1.69-1.64 (m, 1H), 1.28 (app. qt, *J* = 12.7, 3.3 Hz, 2H), 1.17 (app. tt, *J* = 12.3, 3.2 Hz, 1H), 1.14-1.06 (m, 2H).

^{13}C NMR (125 MHz, $CDCl_3$) δ 165.39 (e, d, $^4J_{CF}$ = 3.5 Hz), 162.66 (e, d, $^1J_{CF}$ = 246.9 Hz), 142.63 (o), 132.78 (e, d, $^3J_{CF}$ = 7.6 Hz), 130.04 (o, d, $^3J_{CF}$ = 7.7 Hz), 125.46 (o, d, $^4J_{CF}$ = 2.9 Hz), 121.19 (o), 119.99 (o, d, $^2J_{CF}$ = 21.3), 116.63 (o, d, $^2J_{CF}$ = 23.0), 66.46 (e), 40.50 (o), 32.66 (e), 26.23 (e), 26.07 (e).

IR (neat) 2924 (s), 2852 (m), 1720 (vs), 1592 (m), 1269 (vs), 1198 (vs), 1092 (s), 949 (s), 892 (m), 805 (m), 755 (s) cm^{-1}.

HRMS (EI, M^+) calcd for $C_{16}H_{19}O_2F$ 262.1369, found 262.1359.

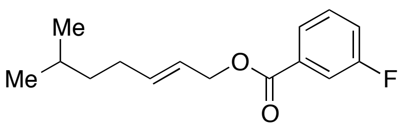

(*E*)-6-Methylhept-2-en-1-yl 3-fluorobenzoate (1q).

Color and State: Colorless oil.

^{1}H NMR (500 MHz, $CDCl_3$) δ 7.84 (d, J = 7.7 Hz, 1H), 7.73 (ddd, J = 9.4, 2.4, 1.6 Hz, 1H), 7.41 (app. td, J = 8.0, 5.6 Hz, 1H), 7.25 (app. tdd, J = 8.3, 2.7, 0.8 Hz, 1H), 5.86 (dt, J = 15.1, 6.9 Hz, 1H), 5.67 (dtt, J = 15.3, 6.4, 1.3 Hz, 1H), 4.76 (d, J = 6.5 Hz, 2H), 2.12 (app. q, J = 7.2 Hz, 2H), 1.59 (septet, J = 6.6 Hz, 1H), 1.34-1.30 (m, 2H), 0.89 (d, J = 6.6 Hz, 6H).

^{13}C NMR (125 MHz, $CDCl_3$) δ 165.37 (e, d, $^4J_{CF}$ = 3.1 Hz), 162.66 (e, d, $^1J_{CF}$ = 247.1 Hz), 137.36 (o), 132.75 (e, d, $^3J_{CF}$ = 7.4 Hz), 130.05 (o, d, $^3J_{CF}$ = 7.8 Hz), 125.46 (o, d, $^4J_{CF}$ = 3.0 Hz), 123.44 (o), 120.01 (o, d, $^2J_{CF}$ = 21.3 Hz), 116.63 (o, d, $^2J_{CF}$ = 23.0 Hz), 66.25 (e), 38.13 (e), 30.28 (e), 27.68 (o), 22.57 (o).

IR (neat) 2956 (m), 2871 (w), 1721 (vs), 1593 (m), 1269 (vs), 1198 (vs), 1092 (m), 953 (s), 892 (m), 805 (m), 755 (s) cm^{-1}.

HRMS (EI, M$^+$) calcd for $C_{15}H_{19}O_2F$ 250.1369, found 250.1372.

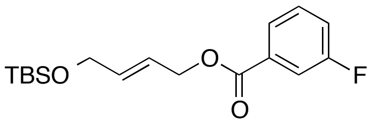

(*E*)-4-((*tert*-Butyldimethylsilyl)oxy)but-2-en-1-yl 3-fluorobenzoate (1r).

Color and State: Colorless oil.

^{1}H NMR (500 MHz, $CDCl_3$) δ 7.84 (dd, J = 7.8, 0.9 Hz, 1H), 7.74-7.71 (m, 1H), 7.41 (app. td, J = 8.0, 5.6 Hz, 1H), 7.25 (app. tdd, J = 8.2, 2.7, 0.9 Hz, 1H), 5.98-5.89 (m, 2H), 4.83 (d, J = 4.6 Hz, 2H), 4.22-4.21 (m, 2H), 0.92 (s, 9H), 0.08 (s, 6H).

^{13}C NMR (125 MHz, $CDCl_3$) δ 165.32 (e, d, $^4J_{CF}$ = 2.7 Hz), 162.68 (e, d, $^1J_{CF}$ = 247.1 Hz), 134.65 (o), 132.60 (e, d, $^3J_{CF}$ = 7.4 Hz), 130.11 (o, d, $^3J_{CF}$ = 7.8 Hz), 125.50 (o, d, $^4J_{CF}$ = 2.9 Hz), 123.39 (o), 120.13 (o, d, $^2J_{CF}$ = 21.3 Hz), 116.67 (o, d, $^2J_{CF}$ = 23.0 Hz), 65.41 (e), 62.99 (e), 26.07 (o), 18.55 (e), –5.12 (o).

IR (neat) 2955 (w), 2930 (m), 2886 (w), 2857 (m), 1724 (s), 1593 (m), 1269 (vs), 1198 (s), 1100 (s), 966 (s), 893 (w), 835 (vs), 807 (m), 755 (s) cm^{-1}.

HRMS (ESI, [M+H]$^+$) calcd for $C_{17}H_{26}O_3FSi$ 325.1630, found 325.1619.

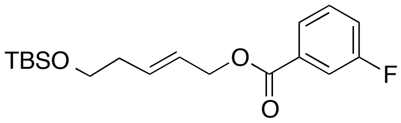

(*E*)-5-((*tert*-Butyldimethylsilyl)oxy)pent-2-en-1-yl 3-fluorobenzoate (1s).

Color and State: Colorless oil.

^{1}H NMR (500 MHz, $CDCl_3$) δ 7.84 (app. dt, J = 7.8, 1.2 Hz, 1H), 7.72 (ddd, J = 9.3, 2.6, 1.5 Hz, 1H), 7.40 (app. td, J = 8.0, 5.5 Hz, 1H), 7.24 (app. tdd, J = 8.3, 2.6, 0.9 Hz, 1H), 5.87 (dtt, J = 15.3,

6.9, 1.2 Hz, 1H), 5.74 (dtt, J = 15.4, 6.3, 1.3 Hz, 1H), 4.77 (dd, J = 6.3, 0.9 Hz, 2H), 3.67 (t, J = 6.6 Hz, 2H), 2.31 (app. qd, J = 6.7, 0.9 Hz, 2H), 0.88 (s, 9H), 0.04 (s, 6H).

^{13}C NMR (125 MHz, $CDCl_3$) δ 165.33 (e, d, $^4J_{CF}$ = 3.0 Hz), 162.67 (e, d, $^1J_{CF}$ = 246.7 Hz), 133.29 (o), 132.68 (e, d, $^3J_{CF}$ = 7.1 Hz), 130.06 (o, d, $^3J_{CF}$ = 7.9 Hz), 125.64 (o), 125.48 (o, d, $^4J_{CF}$ = 2.9 Hz), 120.05 (o, d, $^2J_{CF}$ = 21.2 Hz), 116.64 (o, d, $^2J_{CF}$ = 23.0 Hz), 66.01 (e), 62.55 (e), 36.00 (e), 26.03 (o), 18.44 (e), –5.16 (o).

IR (neat) 2953 (w), 2929 (m), 2887 (w), 2856 (w), 1722 (s), 1592 (m), 1269 (s), 1197 (s), 1091 (vs), 960 (s), 892 (m), 753 (vs) cm^{-1}.

HRMS (ESI, $[M+Na]^+$) calcd for $C_{18}H_{27}O_3FNaSi$ 361.1611, found 361.1625.

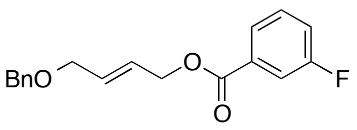

(*E*)-4-(Benzyloxy)but-2-en-1-yl 3-fluorobenzoate (1t).

Color and State: Colorless oil.

^{1}H NMR (500 MHz, $CDCl_3$) δ 7.86 (app. dt, J = 7.8, 1.2 Hz, 1H), 7.75 (ddd, J = 9.3, 2.4, 1.6, 1H), 7.42 (app. td, J = 8.0, 5.6 Hz, 1H), 7.37-7.34 (m, 4H), 7.32-7.29 (m, 1H), 7.27 (app. tdd, J = 8.3, 2.8, 0.9 Hz, 1H), 6.03-5.95 (m, 2H), 4.86-4.85 (m, 2H), 4.55 (s, 2H), 4.09-4.08 (m, 2H).

^{13}C NMR (125 MHz, $CDCl_3$) δ 165.23 (e, d, $^4J_{CF}$ = 2.6 Hz), 162.62 (e, d, $^1J_{CF}$ = 247.1 Hz), 138.12 (e), 132.38 (e, d, $^3J_{CF}$ = 7.4 Hz), 131.35 (o), 130.12 (o, d, $^3J_{CF}$ = 7.6 Hz), 128.53 (o), 127.87 (o), 127.81 (o), 126.35 (o), 125.49 (o, d, $^4J_{CF}$ = 2.8 Hz), 120.18 (o, d, $^2J_{CF}$ = 21.2), 116.64 (o, d, $^2J_{CF}$ = 23.0 Hz), 72.59 (e), 69.85 (e), 64.15 (e).

IR (neat) 2852 (w), 1721 (vs), 1592 (m), 1269 (vs), 1199 (vs), 1100 (s), 964 (s), 893 (m), 806 (m), 755 (s) cm^{-1}.

HRMS (ESI, $[M+Na]^+$) calcd for $C_{18}H_{17}O_3FNa$ 323.1059, found 323.1058.

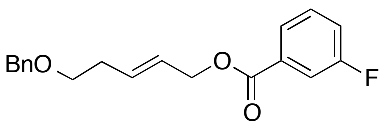

(*E*)-5-(Benzyloxy)pent-2-en-1-yl 3-fluorobenzoate (1u).

Color and State: Colorless oil.

^{1}H NMR (500 MHz, $CDCl_3$) δ 7.84 (d, J = 7.7 Hz, 1H), 7.73 (ddd, J = 9.3, 2.3, 1.4, 1H), 7.41 (app. td, J = 7.9, 5.6 Hz, 1H), 7.34-7.33 (m, 4H), 7.30-7.24 (m, 2H), 5.90 (dt, J = 15.4, 8.7 Hz, 1H), 5.77 (dtt, J = 15.3, 6.3, 1.3 Hz, 1H), 4.78 (d, J = 6.2 Hz, 2H), 4.53 (s, 2H), 3.55 (t, J = 6.6 Hz, 2H), 2.42 (app. q, J = 6.4 Hz, 2H).

^{13}C NMR (125 MHz, $CDCl_3$) δ 165.30 (e, d, $^4J_{CF}$ = 2.9 Hz), 162.63 (e, d, $^1J_{CF}$ = 246.7 Hz), 138.44 (e), 133.02 (o), 132.61 (e, d, $^3J_{CF}$ = 7.6 Hz), 130.06 (o, d, $^3J_{CF}$ = 7.7 Hz), 128.47 (o), 127.74 (o), 127.69 (o), 125.64 (o), 125.45 (o, d, $^4J_{CF}$ = 2.8 Hz), 120.04 (o, d, $^2J_{CF}$ = 21.2 Hz), 116.61 (o, d, $^2J_{CF}$ = 23.0 Hz), 73.03 (e), 69.39 (e), 65.93 (e), 32.85 (e).

IR (neat) 2936 (w), 2856 (w), 1718 (s), 1590 (m), 1267 (vs), 1196 (vs), 1091 (vs), 954 (s), 890 (m), 804 (m), 753 (s), 731 (s), 696 (s) cm^{-1}.

HRMS (ESI, $[M+Na]^+$) calcd for $C_{19}H_{19}O_3FNa$ 337.1215, found 337.1229.

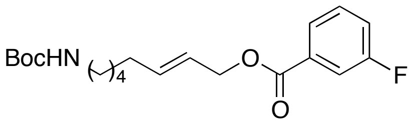

(*E*)-8-[(*tert*Butoxycarbonyl)amino]oct-2-en-1-yl 3-fluorobenzoate (1v).

Color and State: White paste.

^{1}H NMR (500 MHz, $CDCl_3$) δ 7.84 (app. dt, *J* = 7.7, 1.2 Hz, 1H), 7.73 (ddd, *J* = 9.4, 2.5, 1.5 Hz, 1H), 7.41 (app. td, *J* = 8.0, 5.6 Hz, 1H), 7.25 (app. tdd, *J* = 8.3, 2.7, 1.0 Hz, 1H), 5.85 (dt, *J* = 15.3, 6.7 Hz, 1H), 5.70-5.63 (m, 1H), 4.76 (dd, *J* = 6.4 Hz, 2H), 4.50 (br. s, 1H), 3.11 (d, *J* = 5.7 Hz, 2H), 2.09 (app. q, *J* = 7.0 Hz, 2H), 1.49-1.40 (m, 4H), 1.44 (s, 9H), 1.35-1.29 (m, 2H).

^{13}C NMR (125 MHz, $CDCl_3$) δ 165.35 (e, d, $^4J_{CF}$ = 3.0 Hz), 162.58 (e, d, $^1J_{CF}$ = 246.7 Hz), 156.06 (e), 136.73 (o), 132.59 (e, d, $^3J_{CF}$ = 7.4 Hz), 130.04 (o, d, $^3J_{CF}$ = 7.6 Hz), 125.42 (o, d, $^4J_{CF}$ = 2.8 Hz), 123.78 (o), 120.02 (o, d, $^2J_{CF}$ = 21.3 Hz), 116.57 (o, d, $^2J_{CF}$ = 23.0 Hz), 79.06 (e), 66.12 (e), 40.55 (e), 32.25 (e), 29.97 (e), 28.56 (e), 28.49 (o), 26.38 (e).

IR (neat) 3372 (w; br.), 2961 (w), 2931 (w), 2859 (w), 1717 (vs), 1592 (m), 1268 (vs), 1100 (m), 955 (s), 892 (w), 755 (s) cm^{-1}.

HRMS (ESI, $[M+Na]^+$) calcd for $C_{20}H_{28}NO_4FNa$ 388.1900, found 388.1912.

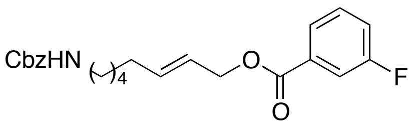

(*E*)-8-[(Benzyloxycarbonyl)amino]oct-2-en-1-yl 3-fluorobenzoate (1w).

Color and State: Colorless oil.

^{1}H NMR (500 MHz, $CDCl_3$) δ 7.84 (d, *J* = 7.6 Hz, 1H), 7.73 (d, *J* = 9.3 Hz, 1H), 7.41 (app. td, *J* = 7.9, 5.6 Hz, 1H), 7.36-7.35 (m, 4H), 7.33-7.29 (m, 1H), 7.25 (app. td, *J* = 8.3, 2.4 Hz, 1H), 5.87-5.81 (m, 1H), 5.69-5.54 (m, 1H), 5.10 (s, 2H), 4.76 (d, *J* = 6.4 Hz, 2H), 4.71 (br. s, 1H), 3.19 (app. q, *J* = 6.8 Hz, 2H), 2.09 (app. q, *J* = 7.1 Hz, 2H), 1.51 (app. quintet , *J* = 7.4 Hz, 2H), 1.43 (app. quintet, *J* = 7.4 Hz, 2H), 1.36-1.31 (m, 2H).

^{13}C NMR (125 MHz, $CDCl_3$) δ 165.26 (e, d, $^4J_{CF}$ = 2.6 Hz), 162.54 (e, d, $^1J_{CF}$ = 247.1 Hz), 156.45 (e), 136.75 (e), 136.53 (o), 132.60 (e, d, $^3J_{CF}$ = 7.3 Hz), 129.99 (o, d, $^3J_{CF}$ = 7.7 Hz), 128.50 (o) 128.08 (o), 128.05 (o), 125.36 (o, d, $^4J_{CF}$ = 3.0 Hz), 123.84 (o), 119.93 (o, d, $^2J_{CF}$ = 21.3 Hz), 116.50 (o, d, $^2J_{CF}$ = 23.0 Hz), 66.54 (e), 66.02 (e), 41.02 (e), 32.15 (e), 29.82 (e), 28.49 (e), 26.26 (e).

IR (neat) 3354 (w; br.), 2931 (m), 2858 (w), 1713 (vs), 1592 (m), 1522 (s), 1269 (vs), 1198 (vs), 1093 (s), 951 (s), 891 (m), 754 (s), 696 (s) cm^{-1}.

HRMS (EI, M^+) calcd for $C_{23}H_{26}NO_4F$ 399.1846, found 399.1829.

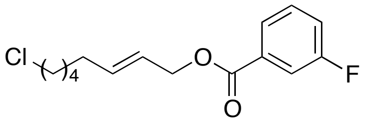

(*E*)-8-Chlorooct-2-en-1-yl 3-fluorobenzoate (1x).

Color and State: Colorless oil.

^{1}H NMR (500 MHz, $CDCl_3$) δ 7.84 (app. dt, *J* = 7.8, 1.0 Hz, 1H), 7.72 (ddd, *J* = 9.3, 2.4, 1.4 Hz, 1H), 7.40 (app. dt, *J* = 8.0, 5.6 Hz, 1H), 7.26-7.22 (m, 1H), 5.85 (dt, *J* = 15.3, 6.7 Hz, 1H), 5.68 (dtt, *J* = 15.3, 6.5, 1.3 Hz, 1H), 4.76 (dd, *J* = 6.5, 0.7 Hz, 2H), 3.52 (app. t, *J* = 6.7 Hz, 2H), 2.10 (app. q, *J* = 6.4 Hz, 2H), 1.77 (app. quintet, *J* = 6.8 Hz, 2H), 1.49-1.40 (m, 4H).

^{13}C NMR (125 MHz, $CDCl_3$) δ 165.35 (e, d, $^{4}J_{CF}$ = 2.9 Hz), 162.64 (e, d, $^{1}J_{CF}$ = 246.7 Hz), 136.50 (o), 132.68 (e, d, $^{3}J_{CF}$ = 7.6 Hz), 130.07 (o, d, $^{3}J_{CF}$ = 7.7 Hz), 125.45 (o, d, $^{4}J_{CF}$ = 3.2 Hz), 124.05 (o), 120.03 (o, d, $^{2}J_{CF}$ = 21.3 Hz), 116.61 (o, d, $^{2}J_{CF}$ = 23.0 Hz), 66.08 (e), 45.06 (e), 32.52 (e), 32.17 (e), 28.20 (e), 26.47 (e).

IR (neat) 2933 (w), 2858 (w), 1718 (s), 1591 (m), 1267 (vs), 1196 (s), 1092 (s), 950 (s), 891 (m), 804 (m), 753 (vs) cm^{-1}.

HRMS (EI, M^+) calcd for $C_{15}H_{18}O_2{}^{35}ClF$ 284.0979, found 284.0973.

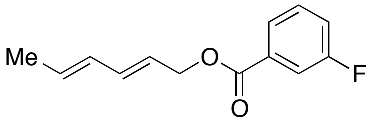

(2*E*,4*E*)-Hexa-2,4-dien-1-yl 3-fluorobenzoate (1y).

Color and State: Pale yellow oil.

^{1}H NMR (500 MHz, $CDCl_3$) δ 7.84 (d, *J* = 7.8 Hz, 1H), 7.73 (ddd, *J* = 9.3, 2.4, 1.5 Hz, 1H), 7.41 (td, *J* = 8.0, 5.6 Hz, 1H), 7.25 (tdd, *J* = 8.3, 2.6, 0.9 Hz, 1H), 6.34 (dd, *J* = 15.2, 10.5 Hz, 1H), 6.09 (ddd, *J* = 15.0, 10.6, 1.1 Hz, 1H), 5.83-5.77 (m, 1H), 5.76-5.70 (m, 1H), 4.83 (d, *J* = 6.7 Hz, 2H), 1.78 (d, *J* = 6.7 Hz, 3H).

^{13}C NMR (125 MHz, $CDCl_3$) δ 165.28 (e, d, $^{4}J_{CF}$ = 2.8), 162.57 (e, d, $^{1}J_{CF}$ = 246.8 Hz), 135.41 (o), 132.53 (e, d, $^{3}J_{CF}$ = 7.4 Hz), 131.69 (o), 130.44 (o), 130.03 (o, d, $^{3}J_{CF}$ = 7.6 Hz), 125.42 (o, d, $^{4}J_{CF}$ = 2.8 Hz), 123.42 (o), 120.03 (o, d, $^{2}J_{CF}$ = 21.3 Hz), 116.57 (o, d, $^{2}J_{CF}$ = 23.0 Hz), 65.88 (e), 18.23 (o).

IR (neat) 3078 (w), 3026 (w), 2937 (w), 1720 (vs), 1662 (w), 1592 (s), 1269 (vs), 1197 (vs), 1091 (s), 952 (s), 892 (m), 806 (m), 754 (vs) cm^{-1}.

HRMS (EI, M^+) calcd for $C_{13}H_{13}O_2F$ 220.0900, found 220.0908.

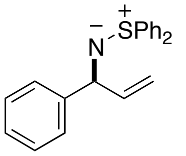

(*S*)-1,1'[*N*-(1-Phenylprop-2-en-1-yl)sulfinimidoyl]dibenzene (2a).

Color and State: Yellow oil; *Selectivity* **2a**/**3a** ≥19:1.

$[\alpha]_D^{20}$ −68.9 (c = 1.0, $CHCl_3$).

Chiral HPLC analysis of the *N*-trifluoroacetamide: 25 cm x 4.6 mm Chiralpak AD-H column, 1% isopropanol-hexane at 1.0 mL/min. flow rate, 215 nm; t_R (*minor*) 11.3 min., t_R (*major*) 12.9 min., 98% *ee*.

^{1}H NMR (500 MHz, $CDCl_3$) δ 7.58-7.56 (m, 2H), 7.52-7.50 (m, 2H), 7.43-7.40 (m, 3H), 7.37-7.33 (m, 5H), 7.21 (app. t, J = 7.6 Hz, 2H), 7.14-7.11 (m, 1H), 5.99 (ddd, J = 17.0, 10.1, 6.8 Hz, 1H), 5.05 (d, J = 17.0 Hz, 1H), 4.91 (d, J = 10.1 Hz, 1H), 4.76 (d, J = 6.8 Hz, 1H).

^{13}C NMR (125 MHz, $CDCl_3$) δ 145.93 (e), 143.83 (o), 141.83 (e), 141.81 (e), 130.50 (o), 130.42 (o), 129.14 (o), 129.07 (o), 128.12 (o), 127.91 (o), 127.79 (o), 127.39 (o), 126.27 (o), 112.87 (e), 67.83 (o).

IR (neat) 3057 (w), 3023 (w), 2925 (w), 1634 (w), 1598 (w), 1580 (w), 1475 (m), 1442 (s), 1057 (m), 1022 (m) cm^{-1}.

HRMS (ESI, $[M+H]^+$) calcd for $C_{21}H_{20}NS$ 318.1316, found 318.1320.

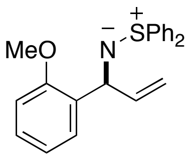

(*S*)-1-{1-[(Diphenyl-λ^4-sulfanylidene)amino]prop-2-en-1-yl}-2-methoxybenzene (2b).

Color and State: Yellow oil; *Selectivity* **2b**/**3b** ≥19:1.

$[\alpha]_D^{20}$ −26.7 (c = 1.1, $CHCl_3$).

Chiral HPLC analysis of the *N*-trifluoroacetamide: 25 cm x 4.6 mm Chiralpak AD-H column, 1% isopropanol-hexane at 1.0 mL/min. flow rate, 210 nm; t_R (*minor*) 7.6 min., t_R (*major*) 8.0 min., 81% *ee*.

^{1}H NMR (500 MHz, $CDCl_3$) δ 7.55-7.53 (m, 3H), 7.49-7.47 (m, 2H), 7.42-7.40 (m, 3H), 7.34-7.26 (m, 3H), 7.08 (app. td, J = 7.8, 1.7 Hz, 1H), 6.85 (app. t, J = 7.3 Hz, 1H), 6.68 (d, J = 8.1 Hz, 1H), 6.05 (ddd, J = 16.8, 10.4, 5.8 Hz, 1H), 5.23 (d, J = 5.7 Hz, 1H), 5.14 (d, J = 17.0 Hz, 1H), 4.94 (app. dt, J = 10.1, 1.5 Hz, 1H), 3.63 (s, 3H).

^{13}C NMR (125 MHz, $CDCl_3$) δ 155.79 (e), 142.97 (o), 141.99 (e), 141.90 (e), 134.14 (e), 130.24 (o), 130.14 (o), 128.95 (o), 128.82 (o), 128.39 (o), 128.01 (o), 127.60 (o), 127.07 (o), 120.57 (o), 112.62 (e), 109.78 (o), 59.81 (o), 55.07 (o).

IR (neat) 3060 (w), 2926 (w), 1635 (m), 1580 (w), 1475 (m), 1443 (s), 1245 (vs), 1067 (m), 1046 (m), 1032 (m) cm^{-1}.

HRMS (ESI, $[M+H]^+$) $C_{22}H_{22}NOS$ 348.1422, found 348.1406.

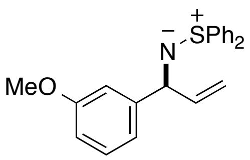

(*S*)-1-{1-[(Diphenyl-λ^4-sulfanylidene)amino]prop-2-en-1-yl}-3-methoxybenzene (2c).

Color and State: Yellow oil; *Selectivity* **2c**/**3c** ≥19:1.

$[\alpha]_D^{20}$ −77.9 (c = 1.1, $CHCl_3$).

Chiral HPLC analysis of the *N*-trifluoroacetamide: 25 cm x 4.6 mm Chiralpak AD-H column, 1% isopropanol-hexane at 1.0 mL/min. flow rate, 210 nm; t_R (*major*) 20.5 min., t_R (*minor*) 23.9 min., 94% *ee*.

^{1}H NMR (500 MHz, $CDCl_3$) δ 7.58-7.56 (m, 2H), 7.53-7.51 (m, 2H), 7.41-7.40 (m, 3H), 7.35-7.33 (m, 3H), 7.12 (t, *J* = 7.8 Hz, 1H), 6.94 (t, *J* = 2.0 Hz, 1H), 6.90 (d, *J* = 7.6 Hz, 1H), 6.67 (dd, *J* = 7.9, 2.2 Hz, 1H), 5.99 (ddd, *J* = 17.1, 10.2, 6.8 Hz, 1H), 5.06 (app. dt, *J* = 17.0, 1.4 Hz, 1H), 4.92 (d, *J* = 10.1 Hz, 1H), 4.75 (d, *J* = 6.7 Hz, 1H), 3.69 (s, 3H).

^{13}C NMR (125 MHz, $CDCl_3$) δ 159.44 (e), 147.65 (e), 143.61 (o), 141.78 (e), 141.69 (e), 130.47 (o), 130.37 (o), 129.10 (o), 129.00 (o), 128.94 (o), 127.87 (o). 127.68 (o), 119.75 (o), 112.91 (e), 112.34 (o), 112.20 (o), 67.79 (o), 55.13 (o).

IR (neat) 3054 (w), 2936 (w), 1634 (w), 1596 (m), 1582 (m), 1475 (m), 1442 (s), 1258 (s), 1147 (m), 1042 (s), 1022 (m) cm^{-1}.

HRMS (ESI, $[M+H]^+$) calcd for $C_{22}H_{22}NOS$ 348.1422, found 348.1407.

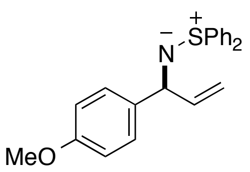

(*S*)-1-{1-[(Diphenyl-λ^4-sulfanylidene)amino]prop-2-en-1-yl}-4-methoxybenzene (2d).

Color and State: Yellow oil; *Selectivity* **2d**/**3d** ≥19:1.

$[\alpha]_D^{20}$ –41.6 (c = 1.1, $CHCl_3$).

Chiral HPLC analysis of the *N*-trifluoroaetamide: 25 cm x 4.6 mm Chiralpak AD-H column, 1% isopropanol-hexane at 1.0 mL/min. flow rate, 210 nm; t_R (*minor*) 18.6 min., t_R (*major*) 21.2 min., 95% *ee*.

^{1}H NMR (500 MHz, $CDCl_3$) δ 7.58-7.55 (m, 2H), 7.52-7.50 (m, 2H), 7.43-7.39 (m, 3H), 7.37-7.32 (m, 3H), 7.27-7.24 (m, 2H), 6.78-6.75 (m, 2H), 5.96 (ddd, *J* = 17.0, 10.1, 6.8 Hz, 1H), 5.01 (dt, *J* = 17.0, 1.4 Hz, 1H), 4.88 (app. dt, *J* = 10.0, 0.8 Hz, 1H), 4.72 (d, *J* = 6.8 Hz, 1H), 3.75 (s, 3H).

^{13}C NMR (125 MHz, $CDCl_3$) δ 158.01 (e), 143.96 (o), 141.78 (e), 141.75 (e), 138.18 (e), 130.45 (o), 130.35 (o), 129.08 (o), 129.02 (o), 128.31 (o), 127.88 (o), 127.69 (o), 113.43 (o), 112.53 (e), 67.09 (o), 55.28 (o).

IR (neat) 3058 (w), 2930 (w), 1673 (w), 1601 (m), 1580 (m), 1508 (s), 1475 (m), 1441 (s), 1243 (vs), 1032 (m), 1023 (m) cm^{-1}.

HRMS (ESI, $[M+H]^+$) calcd $C_{22}H_{22}NOS$ 348.1422, found 348.1412.

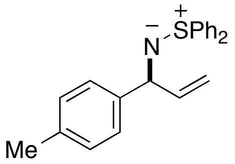

(*S*)-1-{1-[(Diphenyl-λ^4-sulfanylidene)amino]prop-2-en-1-yl}-4-methylbenzene (2e).

Color and State: Yellow oil; *Selectivity* **2e**/**3e** ≥19:1.

$[\alpha]_D^{20}$ –79.8 (c = 1.1, $CHCl_3$).

Chiral HPLC analysis of the *N*-trifluoroacetamide: 25 cm x 4.6 mm Chiralpak AD-H column, 1% isopropanol-hexane at 1.0 mL/min. flow rate, 210 nm; t_R (*major*) 12.0 min., t_R (*minor*) 14.6 min., 93% *ee*.

^{1}H NMR (500 MHz, $CDCl_3$) δ 7.59-7.57 (m, 2H), 7.53-7.51 (m, 2H), 7.42-7.39 (m, 3H), 7.37-7.33 (m, 3H), 7.24 (d, J = 8.0 Hz, 2H), 7.04 (d, J = 7.9 Hz, 2H), 5.97 (ddd, J = 17.1, 10.1, 6.9 Hz, 1H), 5.02 (app. dt, J = 17.0, 1.4 Hz, 1H), 4.88 (d, J = 10.1 Hz, 1H), 4.73 (d, J = 6.8 Hz, 1H), 2.29 (s, 3H).

^{13}C NMR (125 MHz, $CDCl_3$) δ 143.95 (o), 142.99 (e), 141.94 (e), 141.87 (e), 135.67 (e), 130.45 (o), 130.30 (o), 129.07 (o), 129.02 (o), 128.80 (o), 127.94 (o), 127.66 (o), 127.22 (o), 112.59 (e), 67.55 (o), 21.16 (o).

IR (neat) 3053 (w), 2921 (w), 2851 (w), 1633 (w), 1580 (w), 1508 (w), 1475 (m), 1442 (m), 1061 (m), 1020 (m), 907 (s) cm^{-1}.

HRMS (ESI, $[M+H]^+$) calcd for $C_{22}H_{22}NS$ 332.1473, found 332.1487.

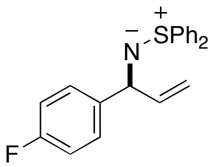

(*S*)-*N*-[3-(4-Fluorophenyl)prop-1-en-3-yl]diphenylsulfilimine (2f).

Color and State: Colorless oil; *Selectivity* **2f**/**3f** ≥19:1.

$[\alpha]_D^{20}$ –63.7 (c = 0.8, $CHCl_3$).

Chiral HPLC analysis of the *N*-trifluoroacetamide: 25 cm x 4.6 mm Chiralpak AD-H column, 1% isopropanol-hexane at 1.0 mL/min. flow rate, 210 nm, 28 °C; t_R(*minor*) 14.0 min., t_R(*major*) 17.7 min., 96% *ee*.

^{1}H NMR (500 MHz, $CDCl_3$) δ 7.56-7.55 (m, 2H), 7.51-7.49 (m, 2H), 7.43-7.41 (m, 3H), 7.38-7.33 (m, 3H), 7.29-7.26 (m, 2H), 6.87 (app. t, J = 8.7 Hz, 2H), 5.96 (ddd, J = 17.0, 10.1, 6.7 Hz, 1H), 5.04 (d, J = 17.0 Hz, 1H), 4.93 (d, J = 10.1 Hz, 1H), 4.73 (d, J = 6.7 Hz, 1H).

^{13}C NMR (125 MHz, $CDCl_3$) δ 161.49 (e, d, $^1J_{CF}$ = 243.5 Hz), 143.76 (o), 141.70 (e, d, $^4J_{CF}$ = 3.4 Hz), 141.68 (e), 141.65 (e), 130.56 (o), 130.55 (o), 129.21 (o), 129.13 (o), 128.80 (o, d, $^3J_{CF}$ = 7.9 Hz), 127.82 (o), 127.77 (o), 114.72 (o, d, $^2J_{CF}$ = 21.0 Hz), 113.05 (e), 67.17 (o).

IR (neat) 3057 (w), 2973 (w), 1633 (w), 1600 (m), 1502 (vs), 1475 (m), 1442 (s), 1216 (v), 1062 (s), 1022 (s), 998 (s), 825 (s) cm^{-1}.

HRMS (ESI, $[M+H]^+$) calcd for $C_{21}H_{19}NSF$ 336.1222, found 336.1234.

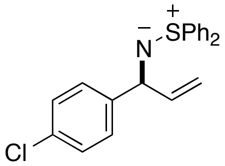

(*S*)-1-Chloro-4-{1-[diphenyl-λ^4-sulfanylidene)amino]prop-2-en-1-yl}benzene (2g).

Color and State: Light orange oil; *Selectivity* **2g**/**3g** =17:1.

$[\alpha]_D^{20}$ –80.1 (c = 1.1, $CHCl_3$).

Chiral HPLC analysis of the *N*-trifluoroacetamide: (25 cm x 4.6 mm Chiralpak AD-H column, 1% isopropanol-hexane at 1.0 mL/min. flow rate, 220 nm; t_R (*minor*) 17.3 min., t_R (*major*) 20.2 min., 96% *ee*.

^{1}H NMR (500 MHz, $CDCl_3$) δ 7.56-7.54 (m, 2H), 7.51-7.48 (m, 2H), 7.43-7.40 (m, 3H), 7.37-7.32 (m, 3H), 7.27-7.24 (m, 2H), 7.17-7.14 (m, 2H), 5.94 (ddd, J = 17.0, 10.1, 6.8 Hz, 1H), 5.04 (app. dt, J = 17.0, 1.4 Hz, 1H), 4.93 (app. dt, J = 10.1, 1.3 Hz, 1H), 4.72 (d, J = 6.8 Hz, 1H).

^{13}C NMR (125 MHz, $CDCl_3$) δ 144.48 (e), 143.42 (o), 141.52 (e), 141.46 (e), 131.70 (e), 130.56 (o), 130.53 (o), 129.18 (o), 129.11 (o), 128.72 (o), 128.07 (o), 127.75 (o), 127.74 (o), 113.22 (e), 67.12 (o).

IR (neat) 3056 (w), 2924 (w), 1633 (w), 1579 (w), 1486 (s), 1475 (s), 1442 (s), 1087 (vs), 1064 (vs), 1014 (s), 810 (m) cm^{-1}.

HRMS (ESI, $[M+H]^+$) calcd for $C_{21}H_{19}NS^{35}Cl$ 352.0927, found 352.0918.

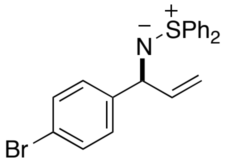

(*S*)-*N*-[3-(4-Bromophenyl)prop-1-en-3yl]diphenylsulfilimine (2h).

Color and State: Colorless oil; *Selectivity* **2h**/**3h** = 18.5:1.

$[\alpha]_D^{20}$ –78.8 (c = 1.9, $CHCl_3$).

Chiral HPLC analysis of the *N*-trifluoroacetamide: 25 cm x 4.6 mm Chiralpak AD-H column, 2% isopropanol-hexane at 1.0 mL/min. flow rate, 210 nm, 28 °C; t_R(*minor*) 12.5 min., t_R(*major*) 17.9 min., 91% *ee*.

^{1}H NMR (500 MHz, $CDCl_3$) δ 7.56-7.54 (m, 2H), 7.50-7.48 (m, 2H), 7.44-7.41 (m, 3H), 7.38-7.33 (m, 3H), 7.32-7.28 (m, 2H), 7.21-7.18 (m, 2H), 5.93 (ddd, J = 17.0, 10.1, 6.8 Hz, 1H), 5.04 (app. dt, J = 16.9, 1.4 Hz, 1H), 4.93 (dd, J = 10.1, 1.3 Hz, 1H), 4.70 (d, J = 6.7 Hz, 1H).

^{13}C NMR (125 MHz, $CDCl_3$) δ 145.09 (e), 143.43 (o), 141.62 (e), 141.57 (e), 131.05 (o), 130.60 (o), 130.57 (o), 129.23 (o), 129.18 (o), 129.16 (o), 127.78 (o), 119.93 (e), 113.28 (e), 67.27 (o).

IR (neat) 3058 (w), 2921 (w), 1633 (w), 1581 (m), 1475 (s), 1442 (s), 1069 (vs), 1023 (s), 1010 (vs) cm^{-1}.

HRMS (ESI, $[M+H]^+$) calcd for $C_{21}H_{19}NS^{79}Br$ 396.0422, found 396.0425.

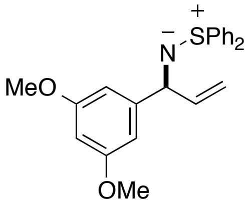

(*S*)-*N*-[3-(3,5-Dimethyoxyphenyl)prop-1-en-3yl] diphenylsulfilimine (2i).

Color and State: Colorless oil; *Selectivity* **2i**/**3i** ≥19:1.

$[\alpha]_D^{20}$ –60.7 (c = 0.7, $CHCl_3$).

Chiral HPLC analysis of the *N*-trifluoroacetamide: 25 cm x 4.6 mm Chiralpak AD-H column, 2% isopropanol-hexane at 1.0 mL/min. flow rate, 210 nm, 28 °C; t_R(*major*) 22.4 min., t_R(*minor*) 30.6 min., 94% *ee*.

^{1}H NMR (500 MHz, $CDCl_3$) δ 7.58-7.56 (m, 2H), 7.53-7.51 (m, 2H), 7.42-7.39 (m, 3H), 7.37-7.33 (m, 3H), 6.50 (d, J = 2.1 Hz, 2H), 6.23 (app. t, J = 2.0 Hz, 1H), 5.98 (ddd, J = 17.0, 10.0, 6.7 Hz, 1H), 5.06 (d, J = 17.0 Hz, 1H), 4.92 (d, J = 10.0 Hz, 1H), 4.70 (d, J = 6.7 Hz, 1H), 3.68 (s, 6H).

^{13}C NMR (125 MHz, $CDCl_3$) δ 160.49 (e), 148.69 (e), 143.51 (o), 141.95 (e), 141.74 (e), 130.55 (o), 130.43 (o), 129.16 (o), 129.05 (o), 127.96 (o), 127.71 (o), 113.07 (e), 105.09 (o), 98.91 (o), 68.13 (o), 55.33 (o).

IR (neat) 3054 (w), 3000 (w), 2937 (w), 2835 (w), 1594 (vs), 1459 (s), 1442 (s), 1426 (s), 1203 (s), 1151 (vs), 1061 (vs), 1022 (m) cm^{-1}.

HRMS (ESI, $[M+H]^+$) calcd for $C_{23}H_{24}NO_2S$ 378.1528, found 378.1525.

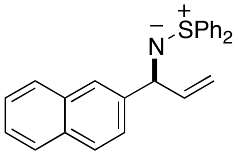

(*S*)-2-{1-[Diphenyl-λ^4-sulfanylidene)amino]prop-2-en-1-yl} naphthalene (2j).

Color and State: Yellow oil; *Selectivity* **2j**/**3j** ≥19:1.

$[\alpha]^{20}_{D}$ −122.4 (c = 1.00, $CHCl_3$).

Chiral HPLC analysis of the *N*-trifluoroacetamide: 25 cm x 4.6 mm Chiralpak AD-H column, 1% isopropanol-hexane at 0.5 mL/min. flow rate, 230 nm; t_R (*minor*) 30.0 min., t_R (*major*) 34.1 min., 96% *ee*.

^{1}H NMR (500 MHz, $CDCl_3$) δ 7.77-7.75 (m, 2H), 7.71 (dd, *J* = 6.5, 2.5 Hz, 1H) 7.70 (d, *J* = 8.4 Hz, 1H), 7.60-7.58 (m, 2H), 7.53-7.52 (m, 2H), 7.48 (dd, *J* = 8.5, 1.6 Hz, 1H), 7.43-7.38 (m, 5H), 7.34-7.29 (m, 3H), 6.05 (ddd, *J* = 16.9, 10.1, 6.7 Hz, 1H), 5.09 (app. dt, *J* = 17.0, 1.3 Hz, 1H), 4.95 (d, *J* = 10.1 Hz, 1H), 4.92 (d, *J* = 6.6 Hz, 1H).

^{13}C NMR (125 MHz, $CDCl_3$) δ 143.62 (o), 143.35 (e), 141.72 (e), 141.58 (e), 133.47 (e), 132.43 (e), 130.49 (o), 130.36 (o), 129.11 (o), 129.01 (o), 127.91 (o), 127.69 (o), 127.63 (o), 127.54 (o), 126.34 (o), 125.59 (o), 125.47 (o), 125.16 (o), 113.13 (e), 67.80 (o).

IR (neat) 3055 (m), 3008 (w), 2972 (w), 1631 (w), 1599 (w), 1580 (w), 1475 (m), 1442 (s), 1065 (m), 1022 (m) 820 (m) cm^{-1}.

HRMS (ESI, $[M+H]^+$) calcd for $C_{25}H_{22}NS$ 368.1473, found 368.1459.

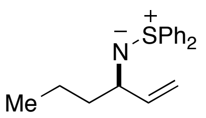

(*R*)-1,1'-[*N*-(Hex-1-en-3-yl)sulfinimidoyl]dibenzene 2k.

Color and State: Colorless oil; *Selectivity* **2k**/**3k** ≥19:1.

$[\alpha]^{20}_{D}$ −52.3 (c = 1.0, $CHCl_3$).

Chiral HPLC analysis of the *N*-tosyl derivative: 25 cm x 4.6 mm Chiralpak AD-H column, 96:3:1 hexane-isopropanol-methanol at 1.0 mL/min. flow rate, 230 nm, 28 °C; t_R(*minor*) 17.7 min., t_R(*major*) 18.7 min., 95% *ee*.

^{1}H NMR(500 MHz, $CDCl_3$) δ 7.60-7.56 (m, 4H), 7.43-7.34 (m, 6H), 5.77 (ddd, *J* = 17.3, 10.0, 7.5 Hz, 1H), 4.93 (dd, *J* = 17.2, 1.0 Hz, 1H), 4.82 (dd, *J* = 10.1, 1.6 Hz, 1H), 3.53 (app. q, *J* = 7.0 Hz, 1H), 1.50 (app. qt, *J* = 13.0, 6.5 Hz, 1H), 1.48 (app. qt, *J* = 12.8, 6.6 Hz, 1H), 1.37-1.23 (m, 2H), 0.82 (t, *J* = 7.4 Hz, 3H),

^{13}C NMR (125 MHz, $CDCl_3$) δ 145.00 (o), 143.63 (e), 142.86 (e), 130.35 (o), 130.21 (o), 129.11 (o), 129.02 (o), 127.61 (o), 127.37 (o), 112.09 (e), 65.53 (o), 41.17 (e), 19.79 (e), 14.17 (o).

IR (neat) 3059 (w), 2956 (m), 2928 (m), 2870 (w), 1635 (w), 1580 (w), 1475 (m), 1442 (s), 1064 (m), 1022 (m) cm^{-1}.

HRMS (ESI, $[M+H]^+$) calcd for $C_{18}H_{22}NS$ 284.1473, found 284.1466.

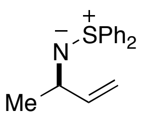

(*R*)-1,1'-[*N*-(But-1-en-3-yl)sulfinimidoyl]dibenzene (2l).

Color and State: Pale yellow oil; *Selectivity* **2l**/**3l** ≥19:1.

$[\alpha]_D^{20}$ –9.8 (c = 0.8, $CHCl_3$).

Chiral HPLC analysis of the *N*-tosyl derivative: 25 cm x 4.6 mm Chiralpak AD-H column, 96:3:1 hexane-isopropanol-methanol at 1.0 mL/min. flow rate, 230 nm, 28 °C; t_R(*major*) 34.2 min., t_R(*major*) 42.2 min., 95% *ee*.

^{1}H NMR (500 MHz, $CDCl_3$) δ 7.61-7.56 (m, 4H), 7.44-7.40 (m, 6H), 5.83 (ddd, *J* = 17.0, 10.3 6.6 Hz, 1H), 4.98 (app. dt, *J* = 17.1, 1.5 Hz, 1H), 4.81 (ddd, *J* = 10.2, 1.7, 1.1 Hz, 1H), 3.76 (app. quintet of t, *J* = 6.6, 1.1 Hz, 1H), 1.18 (d, *J* = 6.5 Hz, 3H).

^{13}C NMR (125 MHz, $CDCl_3$) δ 145.59 (o), 142.77 (e), 142.44 (e), 130.44 (o), 130.27 (o), 129.17 (o), 129.03 (o), 127.75 (o), 127.47 (o), 111.49 (e), 59.37 (o), 24.93 (o).

IR (neat) 3056 (w), 2963 (w), 2920 (w), 1635 (w), 1580 (w), 1474 (m), 1442 (s), 1065 (s), 1021 (m) cm^{-1}.

HRMS (ESI, $[M+H]^+$) calcd for $C_{16}H_{18}NS$ 256.1160, found 256.1161.

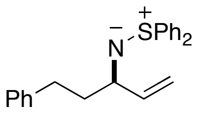

(*R*)-1,1'-[*N*-(5-phenylpent-1-en-3yl)sulfinimidoyl]dibenzene (2m).

Color and State: Colorless oil; *Selectivity* **2m**/**3m** ≥19:1.

$[\alpha]_D^{20}$ –48.3 (c = 1.0, $CHCl_3$).

Chiral HPLC analysis of the *N*-tosyl derivative: 25 cm x 4.6 mm Chiralcel OD column, 96:3:1 hexane-isopropanol-methanol at 1.0 mL/min. flow rate, 230 nm, 28 °C; t_R(*major*) 20.2 min., t_R(*minor*) 23.1 min., 91% *ee*.

^{1}H NMR (500 MHz, $CDCl_3$) δ 7.65-7.61 (m, 2H), 7.60-7.57 (m, 2H), 7.45-7.40 (m, 6H), 7.22 (app. t, *J* = 7.5 Hz, 2H), 7.13 (app. t, *J* = 7.3 Hz, 1H), 7.09 (d, *J* = 7.2 Hz, 2H), 5.85 (ddd, *J* = 17.2, 10.0, 7.5 Hz, 1H), 5.00 (ddd, *J* = 17.3, 1.7, 0.9 Hz, 1H), 4.90 (ddd, *J* = 10.1, 1.9, 0.7 Hz, 1H), 3.61 (app. q, *J* = 6.9 Hz, 1H), 2.66 (ddd, A of ABMN, J_{AB} = 13.6 Hz, J_{AM} = 9.4 Hz, J_{AN} = 6.5 Hz, 1H), 2.61 (ddd, B of ABMN, J_{AB} = 13.8 Hz, J_{BM} = 9.3 Hz, J_{BN} = 6.4 Hz, 1H), 1.89 (app. ddt, *J* = 13.4, 9.6, 6.7 Hz, 1H), 1.82 (app. ddt, *J*= 13.3, 9.7, 6.6 Hz, 1H).

^{13}C NMR (125 MHz, $CDCl_3$) δ 144.56 (o), 143.48 (e), 143.03 (e), 142.62 (e), 130.43 (o), 130.35 (o), 129.18 (o), 129.10 (o), 128.57 (o), 128.23 (o), 127.56 (o), 127.35 (o), 125.48 (o), 112.68 (e), 65.31 (o), 40.40 (e), 32.92 (e).

IR (neat) 3060 (w), 3025 (w), 2919 (w), 1635 (w), 1602 (w), 1580 (w), 1496 (m), 1475 (m), 1442 (s), 1065 (s), 1022 (m) cm^{-1}.

HRMS (EI, M^+) calcd for $C_{23}H_{23}NS$ 345.1551, found 345.1556.

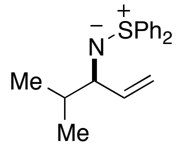

(*R*)-1,1'-[*N*-(4-Methylpent-1-en-3-yl)sulfinimidoyl]dibenzene (2n).

Color and State: Yellow oil; *Selectivity* **2n**/**3n** ≥19:1.

$[\alpha]_D^{20}$ –57.0 (c = 0.9, $CHCl_3$).

Chiral HPLC analysis of the *N*-tosyl derivative: 25 cm x 4.6 mm Chiralpak AS-H column, 96:3:1 hexane-isopropanol-methanol at 1.0 mL/min. flow rate, 230 nm, 28 °C; t_R(*major*) 15.9 min., t_R(*minor*) 24.2 min., 95% *ee*.

^{1}H NMR (500 MHz, $CDCl_3$) δ 7.62-7.60 (m, 2H), 7.57-7.55 (m, 2H), 7.44-7.38 (m, 6H), 5.79 (ddd, *J* = 17.4, 9.8, 8.1 Hz, 1H), 4.92 (dd, *J* = 17.1, 1.9 Hz, 1H), 4.87 (dd, *J* = 10.2, 2.1 Hz, 1H), 3.23 (app. t, *J* = 7.3 Hz, 1H), 1.68 (app. octet, *J* = 6.7 Hz, 1H), 0.90 (d, *J* = 6.7 Hz, 3H), 0.86 (d, *J* = 6.7 Hz, 3H).

^{13}C NMR (125 MHz, $CDCl_3$) δ 143.93 (e), 143.29 (o), 142.97 (e), 130.20 (o), 130.17 (o), 128.99 (o), 127.37 (o), 127.35 (o), 112.98 (e), 73.09 (o), 35.19 (o), 20.17 (o), 19.34 (o).

IR (neat) 3059 (w), 2952 (m), 2867 (w), 1634 (w), 1580 (w), 1474 (m), 1442 (s), 1057 (vs), 1022 (s) cm^{-1}.

HRMS (ESI, $[M+H]^+$) calcd for $C_{18}H_{22}NS$ 284.1473, found 284.1479.

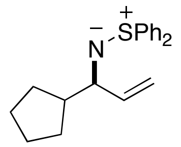

(*S*)-1,1'-[*N*-(3-Cyclopentylprop-1-en-3-yl)sulfinimidoyl]dibenzene (2o).

Color and State: White paste; *Selectivity* **2o**/**3o** ≥19:1.

$[\alpha]_D^{23}$ –90.5 (c = 1.0, $CHCl_3$).

Chiral HPLC analysis of the *N*-tosyl derivative: 25 cm x 4.6 mm Chiralpak AS-H column, 96:3:1 hexane-isopropanol-methanol at 1.0 mL/min. flow rate, 230 nm, 25 °C; t_R(*minor*) 22.0 min., t_R(*major*) 37.4 min., 97% *ee*.

^{1}H NMR (500 MHz, benzene-d_6) δ 7.71 (d, *J* = 7.4 Hz, 2H), 7.68, (d, *J* = 7.3 Hz, 2H), 6.99 (app. t, *J* = 7.7 Hz, 2H), 6.97 (app. t, *J* = 7.7 Hz, 2H), 6.91 (app. t, *J* = 7.2 Hz, 1H), 6.89 (app. t, *J* = 7.5 Hz, 1H), 6.07 (ddd, *J* = 17.3, 9.8, 7.9 Hz, 1H), 5.18 (dd, *J* = 17.1, 2.4 Hz, 1H), 4.97 (dd, *J* = 10.1, 2.4 Hz, 1H), 3.63 (app. t, *J* = 7.6 Hz, 1H), 2.39 (sextet, *J* = 7.9 Hz, 1H), 2.11-2.05 (m, 2H), 1.86-1.76 (m, 2H), 1.74-1.65 (m, 2H), 1.64-1.52 (m, 2H).

^{13}C NMR (125 MHz, benzene-d_6) δ 146.68 (e), 145.74 (o), 145.71 (e), 130.25 (o), 130.22 (o), 129.36 (o), 129.31 (o), 127.01 (o), 126.94 (o), 112.51 (e), 72.25 (o), 48.77 (o), 30.84 (e), 30.70 (e), 26.71 (e), 26.55 (e).

IR (neat) 3054 (w), 2944 (m), 2861 (m), 1632 (w), 1579 (w), 1472 (m), 1440 (m), 1054 (s), 1017 (s) cm^{-1}.

HRMS (EI, M^+) calcd for $C_{20}H_{23}NS$ 309.1551, found 309.1562.

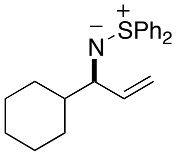

(*S*)-1,1'-[*N*-(3-cyclohexylprop-1-en-3-yl)sulfinimidoyl]dibenzene (2p).

Color and State: White solid; **mp** 41-42 °C; *Selectivity* **2p**/**3p** ≥19:1.

$[\alpha]_D^{20}$ −70.1 (c = 0.9, $CHCl_3$).

Chiral HPLC analysis of the *N*-tosyl derivative: 25 cm x 4.6 mm Chiralcel AD-H column, 96:3:1 hexane-isopropanol-methanol at 0.5 mL/min. flow rate, 230 nm, 25 °C; t_R(*minor*) 63.0 min., t_R(*major*) 68.9 min., 97% *ee*.

^{1}H NMR (500 MHz, $CDCl_3$) δ 7.61-7.59 (m, 2H), 7.56-7.54 (m, 2H), 7.43-7.38 (m, 6H), 5.75 (ddd, *J* = 17.4, 9.6, 8.4 Hz, 1H), 4.88 (dd, *J* = 17.1, 2.0 Hz, 1H), 4.83 (dd, *J* = 10.1, 1.9 Hz, 1H), 3.22 (app. t, *J* = 7.8, 1H), 1.97-1.94 (m, 1H), 1.78-1.75 (m, 1H), 1.71-1.64 (m, 2H), 1.60-1.58 (m, 1H), 1.36 (app. tdt, *J* = 11.2, 7.7, 3.5 Hz 1H), 1.21-1.11 (m, 2H), 1.08 (app. qt, *J* = 12.3, 3.3 Hz, 1H), 0.87 (app. qdd, *J* = 12.1, 7.0, 3.5 Hz, 2H).

^{13}C NMR (125 MHz, $CDCl_3$) δ 143.91 (e), 143.74 (o), 142.87 (e), 130.20 (o), 130.17 (o), 128.99 (o), 128.97 (o), 127.36 (o), 127.35 (o), 112.67 (e), 72.47 (o), 44.78 (o), 30.62 (e), 30.11 (e), 26.92 (e), 26.53 (e), 26.51 (e).

IR (neat) 2922 (s), 2847 (s), 2803 (m), 1627 (w), 1574 (w), 1469 (m), 1434 (s), 1034 (vs), 740 (s) cm^{-1}.

HRMS (ESI, $[M+H]^+$) calcd for $C_{21}H_{26}NS$ 324.1786, found 324.1783.

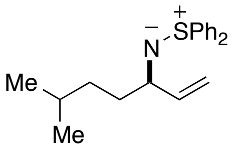

(*R*)-1,1'-[*N*-(6-Methylhept-1-en-3-yl)sulfinimidoyl] dibenzene (2q).

Color and State: Yellow oil; *Selectivity* **2q**/**3q** ≥19:1.

$[\alpha]_D^{20}$ −37.2 (c = 0.8, $CHCl_3$).

Chiral HPLC analysis of the *N*-tosyl derivative: 25 cm x 4.6 mm Chiralpak AD-H column, 96:3:1 hexane-isopropanol-methanol at 1.0 mL/min. flow rate, 230 nm, 28 °C; t_R(*major*) 17.2 min., t_R(*minor*) 20.8 min., 94% *ee.*

^{1}H NMR (500 MHz, $CDCl_3$) δ 7.60-7.57 (m, 4H), 7.43-7.40 (m, 6H), 5.77 (ddd, *J* = 17.3, 10.0, 7.5 Hz, 1H), 5.77 (ddd, *J* = 17.2, 1.9, 0.9 Hz, 1H), 4.94 (ddd, *J* = 17.2, 1.9, 0.9 Hz, 1H), 4.85 (ddd, *J* = 10.1, 2.1, 0.5 Hz, 1H), 3.48 (app. q, *J* = 7.0 Hz, 1H), 1.53-1.43 (m, 2H), 1.43 (septet, *J* = 6.7, 1H), 1.15-1.10 (m, 2H), 0.78 (d, *J* = 6.6 Hz, 6H).

^{13}C NMR (125 MHz, $CDCl_3$) δ 144.97 (o), 143.53 (e), 142.80 (e), 130.39 (o), 130.22 (o), 129.13 (o), 129.01 (o), 127.70 (o), 127.42 (o), 112.24 (e), 65.99 (o), 36.75 (e), 35.89 (e), 28.13 (o), 22.79 (o), 22.77 (o).

IR (neat) 3060 (w), 2952 (m), 2926 (m), 2867 (m), 1635 (w), 1580 (w), 1474 (m), 1442 (s), 1064 (s), 1022 (s) cm^{-1}.

HRMS (EI, M^+) calcd for $C_{20}H_{25}NS$ 311.1708, found 312.1719.

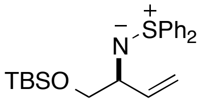

(*S*)-1,1'-{*N*-[4-((*tert*-Butyldimethylsilyl)oxy)but-1-en-3yl]sulfinimidoyl}dibenzene (2r).

Color and State: Clear oil; *Selectivity* **2r**/**3r** = 17:1.

$[\alpha]_D^{20}$ –17.4 (c = 0.8, $CHCl_3$).

Chiral HPLC analysis of the *N*-tosyl derivative: 25 cm x 4.6 mm Chiralcel OJ-H column, 98:1:1 hexane-isopropanol-methanol at 1.0 mL/min. flow rate, 230 nm, 28 °C; t_R(*major*) 8.0 min., t_R(*minor*) 8.5 min., 95% *ee*.

^{1}H NMR (500 MHz, $CDCl_3$) δ 7.63-7.58 (m, 4H), 7.43-7.38 (m, 6H), 5.87 (ddd, J = 17.1, 10.4, 6.4 Hz, 1H), 5.12 (app. dt, J = 17.2, 1.6 Hz, 1H), 4.96 (ddd, J = 10.2, 1.8, 1.1 Hz, 1H), 3.72 (app. q, J = 6.7 Hz, 1H), 3.51 (dd, A of ABM, J_{AB} = 9.7 Hz, J_{AM} = 7.0 Hz, 1H), 3.47 (dd, B of ABM, J_{AB} = 10.2 Hz, J_{BM} = 6.7 Hz, 1H), 0.82 (s, 9H), –0.03 (s, 3H), –0.06 (s, 3H).

^{13}C NMR (125 MHz, $CDCl_3$) δ 143.31 (e), 142.94 (e), 141.67 (o), 130.38 (o), 130.24 (o), 129.17 (o), 128.97 (o), 127.60 (o), 127.50 (o), 114.28 (e), 69.08 (e), 66.43 (o), 26.08 (o), 18.50 (e), –5.18 (o).

IR (neat) 3057 (w), 2953 (m), 2927 (m), 2855 (m), 1637 (w), 1581 (w), 1473 (m), 1443 (m), 1101 (s), 1065 (s), 1023 (m), 835 (vs) cm^{-1}.

HRMS (ESI, $[M+H]^+$) calcd for $C_{22}H_{32}NOSSi$ 386.1968, found 386.1956.

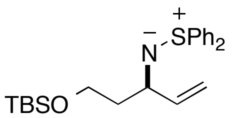

(*R*)-1,1'-{*N*-[4-((*tert*-Butyldimethylsilyl)oxy)pent-1-en-4yl]sulfinimidoyl} dibenzene (2s).

Color and State: Light yellow oil; *Selectivity* **2s**/**3s** = 14:1.

$[\alpha]_D^{23}$ –41.0 (c = 1.0, $CHCl_3$).

Chiral HPLC analysis of the *N*-tosyl derivative: 25 cm x 4.6 mm Chiralcel OJ-H column, 98:1:1 hexane-isopropanol-methanol at 0.5 mL/min. flow rate, 230 nm, 25 °C; t_R(*major*) 14.9 min., t_R(*minor*) 15.7 min., 93% *ee.*

^{1}H NMR (500 MHz, benzene-d_6) δ 7.67-7.63 (m, 4H), 7.00-6.95 (m, 4H), 6.93-6.88 (m, 2H), 6.06 (ddd, J = 17.1, 10.0, 7.2 Hz, 1H), 5.23 (ddd, J = 17.1, 2.1, 1.0 Hz, 1H), 4.95 (dd, J = 10.1, 2.0 Hz, 1H), 4.11 (app. q, J = 7.2 Hz, 1H), 3.98 (dt, A of ABX_2, J_{AB} = 9.9 Hz, J_{AX} = 6.7 Hz, 1H), 3.88 (dt,

B of ABX_2, J_{AB} = 9.9 Hz, J_B = 6.2 Hz, 1H), 2.23-2.17 (m, 1H), 2.14-2.07 (m, 1H), 0.99 (s, 9H), 0.06 (s, 3H), 0.05 (s, 3H).

^{13}C NMR (125 MHz, benzene-d_6) δ 146.20 (e), 146.14 (o), 145.37 (e), 130.31 (o), 129.37 (o), 127.19 (o), 127.14 (o), 112.43 (e), 63.42 (o), 61.45 (e), 43.18 (e), 26.62 (o), 18.90 (e), –4.67 (o), –4.71 (o).

IR (neat) 3059 (w), 2950 (m), 2927 (m), 2854 (m), 1635 (w), 1580 (w), 1471 (m), 1441 (m), 1076 (vs), 1021 (m), 831 (vs) cm^{-1}.

HRMS (EI, M^+) calcd for $C_{23}H_{33}NOSSi$ 399.2052, found 399.2064.

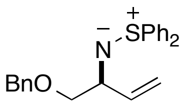

(*S*)-1,1'-{*N*-[4-(Benzyloxy)but-1-en-3yl]sulfinimidoyl}dibenzene (2t).

Color and State: Yellow oil; *Selectivity* **2t**/**3t** = 16:1.

$[\alpha]_D^{20}$ –21.5 (c = 0.8, $CHCl_3$).

Chiral HPLC analysis of the *N*-trifluoroacetamide: 25 cm x 4.6 mm Chiralpak AS-H column, 98:1:1 hexane-isopropanol-methanol at 1.0 mL/min. flow rate, 210 nm, 28 °C; t_R(*minor*) 8.2 min., t_R(*major*) 8.8 min., 92% *ee*.

^{1}H NMR (500 MHz, $CDCl_3$) δ 7.62-7.59 (m, 4H), 7.40-7.37 (m, 6H), 7.30-7.21 (m, 5H), 5.90 (ddd, *J* = 17.0, 10.4, 6.4 Hz, 1H), 5.16 (app. dt, *J* = 17.1, 1.6 Hz, 1H), 4.99 (ddd, *J* = 10.3, 1.7, 1.3 Hz, 1H), 4.41 (s, 2H), 3.94-3.90 (m, 1H), 3.49 (dd, A of ABM, J_{AB} = 9.3 Hz, J_{AM} = 6.1 Hz, 1H), 3.38 (dd, B of ABM, J_{AB} = 9.3 Hz, J_{BM} = 7.1 Hz, 1H).

^{13}C NMR (125 MHz, $CDCl_3$) δ 143.04 (e), 141.32 (o), 138.72 (e), 130.31 (o), 130.27 (o), 129.09 (o), 128.97 (o), 128.23 (o), 127.67 (o), 127.47 (o), 127.42 (o), 127.33 (o), 114.38 (e), 76.42 (e), 73.10 (e), 64.43 (o).

IR (neat) 3059 (w), 2848 (m), 1722 (w), 1637 (w), 1580 (w), 1475 (m), 1442 (s), 1075 (vs), 1023 (s) cm^{-1}.

HRMS (ESI, $[M+H]^+$) calcd for $C_{23}H_{24}NOS$ 362.1579, found 362.1586.

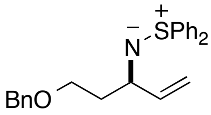

(*R*)-1,1'-{*N*-[4-(Benzyloxy)pent-1-en-4yl]sulfinimidoyl}dibenzene (2u).

Color and State: Light yellow oil; *Selectivity* **2u**/**3u** = 17:1.

$[\alpha]_D^{23}$ –37.7 (c = 1.0, $CHCl_3$).

Chiral HPLC analysis of the *N*-trifluoroacetamide: 25 cm x 4.6 mm Chiralpak AS-H column, 98:1:1 hexane-isopropanol-methanol at 0.5 mL/min. flow rate, 210 nm, 25 °C; t_R(*minor*) 15.6 min., t_R(*major*) 29.8 min., 90% *ee*.

^{1}H NMR (500 MHz, benzene-d_6[9]) δ 7.67-7.61 (m, 4H), 7.32-7.30 (m, 2H), 7.16 (app. t, *J* = 7.5 Hz, 2H), 7.10-7.07 (m, 1H), 6.97-6.94 (m, 4H), 6.92-6.88 (m, 2H), 6.05 (ddd, *J* = 17.2, 10.0, 7.2 Hz, 1H), 5.21 (ddd, *J* = 17.1, 2.2, 1.1 Hz, 1H), 4.94 (ddd, *J* = 10.1, 2.2, 0.7 Hz, 1H), 4.34 (d, A of AB, J_{AB} = 12.1 Hz, 1H), 4.31 (d, B of AB, J_{AB} = 12.2 Hz, 1H), 4.16 (app. q, *J* = 7.2 Hz, 1H), 3.78 (dt, A of ABX_2, J_{AB} = 9.1 Hz, J_{AX} = 6.7 Hz, 1H), 3.66 (dt, B of ABX_2, J_{AB} = 9.1 Hz, J_{BX} = 6.0 Hz, 1H), 2.27-2.16 (m, 2H).

^{13}C NMR (125 MHz, benzene-d_6) δ 146.05 (o), 145.96 (e), 145.42 (e), 140.24 (e), 130.30 (o), 129.37 (o), 128.77 (o), 128.01 (o), 127.69 (o), 127.22 (o), 112.52 (e), 73.26 (e), 68.69 (e), 63.58 (o), 40.17 (e).

IR (neat) 3058 (w), 2909 (w), 2851 (w), 1634 (w), 1579 (w), 1473 (m), 1441 (m), 1072 (vs), 1021 (s) cm^{-1}.

HRMS (EI, M^+) calcd for $C_{24}H_{25}NOS$ 375.1657, found 375.1645.

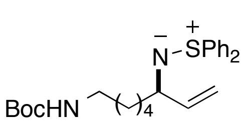

(*R*)-1,1'-{*N*-[8-(*tert*-Butoxycarbonyl)amino-oct-1-en-3yl]sulfinimidoyl}dibenzene (2v).

Color and State: Colorless oil; *Selectivity* **2v**/**3v** ≥19:1.

$[\alpha]^{20}_{D}$ –22.0 (c = 0.9, $CHCl_3$).

Chiral HPLC analysis of the *N*-tosyl derivative: 25 cm x 4.6 mm Chiralcel OD column, 94:5:1 hexane-isopropanol-methanol at 1.0 mL/min. flow rate, 230 nm, 28 °C; t_R(*major*) 17.5 min., t_R(*minor*) 19.6 min., 93% *ee.*

^{1}H NMR (500 MHz, $CDCl_3$) δ 7.60-7.56 (m, 4H), 7.42-7.41 (m, 6H), 5.76 (ddd, *J* = 17.3, 9.9, 7.6 Hz, 1H), 4.94 (d, *J* = 17.1 Hz, 1H), 4.84 (dd, *J* = 10.0, 1.8 Hz, 1H), 4.47-4.41 (br. m, 1H), 3.50 (app. q, *J* = 7.0 Hz, 1H), 3.06-3.02 (m, 2H), 1.51-1.46 (m, 2H), 1.43 (s, 9H), 1.39-1.34 (m, 2H), 1.30-1.19 (m, 4H).

^{13}C NMR (125 MHz, $CDCl_3$) δ 156.01 (e), 144.77 (o), 143.37 (e), 142.69 (e), 130.37 (o), 130.25 (o), 129.10 (o), 129.02 (o), 127.60 (o), 127.33 (o), 112.27 (e), 78.95 (e), 65.57 (o), 40.60 (e), 38.71 (e), 30.03 (e), 28.51 (o), 26.81 (e), 26.20 (e).

IR (neat) 3331 (w; br.), 3060 (w), 2975 (m), 2929 (m), 2855 (m), 1691 (vs), 1637 (w), 1580 (w), 1512 (s), 1475 (m), 1442 (s), 1390 (w), 1170 (vs), 1063 (s), 1022 (s) cm^{-1}.

HRMS (ESI, $[M+H]^+$) calcd for $C_{25}H_{35}N_2O_2S$ 427.2419, found 427.2420.

---

[9] Several spectra were obtained in C_6D_6 to avoid partial decomposition in $CDCl_3$, which includes $CDCl_3$ stored over potassium carbonate.

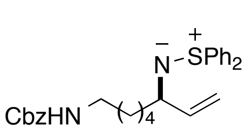

(*R*)-1,1'-{*N*-[8-(Benzyloxycarbonyl)amino-oct-1-en-3yl]sulfinimidoyl}dibenzene (2w).

Color and State: Light yellow oil. *Selectivity* **2w**/**3w** ≥19:1.

$[\alpha]_D^{23}$ –32.2 (c = 1.1, $CHCl_3$).

Chiral HPLC analysis of the *N*-tosyl derivative: 25 cm x 4.6 mm Chiralcel AD-H column, 91:8:1 hexane-isopropanol-methanol at 1.0 mL/min. flow rate, 230 nm, 28 °C; t_R(*minor*) 76.3 min., t_R(*major*) 92.1 min., 92% *ee*.

^{1}H NMR (500 MHz, benzene-d_6) δ 7.67-7.64 (m, 4H), 7.28-7.26 (m, 2H), 7.14-7.11 (m, 2H), 7.08-7.05 (m, 1H), 7.03-6.96 (m, 4H), 6.95-6.90 (m, 2H), 6.05 (ddd, *J* = 17.2, 10.1, 7.3 Hz, 1H), 5.22 (ddd, *J* = 17.1, 2.1, 0.8 Hz, 1H), 5.12 (s, 2H), 4.99 (dd, *J* = 10.1, 2.1 Hz, 1H), 4.26 (br. s, 1H), 3.78 (app. q, *J* = 6.9 Hz, 1H), 2.96 (app. q, *J* = 6.5 Hz, 2H), 1.82 (ddt, *J* = 13.0, 9.7, 6.4 Hz, 1H), 1.71 (ddt, *J* = 12.8, 9.4, 6.3 Hz, 1H), 1.49-1.40 (m, 2H), 1.20-1.07 (m, 4H).

^{13}C NMR (125 MHz, methanol-d_4) δ 158.91 (e), 145.32 (o), 143.05 (e), 142.39 (e), 138.62 (e), 132.30 (o), 132.15 (o), 130.62 (o), 130.55 (o), 129.56 (o), 129.03 (o), 128.97 (o), 128.86 (o), 128.67 (o), 113.59 (e), 67.35 (e), 67.08 (o), 41.89 (e), 39.37 (e), 30.99 (e), 27.79 (e), 27.38 (e).

IR (neat) 3332 (w; br.), 3175 (w; br.), 2927 (m), 2853 (w), 1707 (s), 1524 (m), 1473 (m), 1440 (m), 1247 (s), 1059 (m), 1020 (s) cm^{-1}.

HRMS (EI, M^+) calcd for $C_{28}H_{32}N_2O_2S$ 460.2185, found 460.2199.

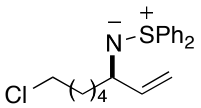

(*R*)-1,1'-[*N*-(8-chloro-oct-1-en-3yl)sulfinimidoyl]dibenzene (2x).

Color and State: Light yellow oil; *Selectivity* **2x**/**3x** ≥19:1.

$[\alpha]_D^{23}$ –38.0 (c = 1.0, $CHCl_3$).

Chiral HPLC analysis of the *N*-tosyl derivative: 25 cm x 4.6 mm Chiralcel AD-H column, 94:5:1 hexane-isopropanol-methanol at 1.0 mL/min. flow rate, 230 nm, 25 °C; t_R(*major*) 17.2 min., t_R(*minor*) 28.2 min., 92% *ee.*

^{1}H NMR (500 MHz, benzene-d_6) δ 7.67-7.65 (m, 4H), 7.01-6.96 (m, 4H), 6.94-6.89 (m, 2H), 6.04 (ddd, *J* = 17.1, 10.0, 7.3 Hz, 1H), 5.23 (ddd, *J* = 17.1, 2.2, 1.0 Hz, 1H), 4.99 (ddd, *J* = 10.0, 2.3, 0.8 Hz, 1H), 3.78 (app. q, *J* = 6.8 Hz, 1H), 3.09 (t, *J* = 6.8 Hz, 2H), 1.82 (ddt, *J* = 13.0, 9.4, 6.5 Hz, 1H), 1.70 (ddt, *J* = 12.9, 9.2, 6.4 Hz, 1H), 1.49-1.36 (m, 4H), 1.30-1.20 (m, 2H).

^{13}C NMR (125 MHz, benzene-d_6) δ 146.26 (o), 146.01 (e), 145.52 (e), 130.36 (o), 130.35 (o), 129.41 (o), 129.39 (o), 127.29 (o), 127.12 (o), 112.42 (e), 66.54 (o), 45.33 (e), 39.88 (e), 33.37 (e), 27.58 (e), 26.38 (e).

IR (neat) 3056 (w), 2929 (m), 2853 (w), 1634 (w), 1579 (w), 1473 (m), 1440 (m), 1061 (s), 1020 (s) cm^{-1}.

HRMS (EI, M^+) calcd for $C_{20}H_{24}NS^{35}Cl$ 345.1318, found 345.1332.

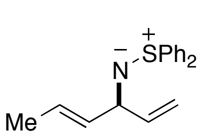

(*S*)-1,1'-[*N*-(hexa-1,4-dien-3-yl)sulfinimidoyl]dibenzene (2y).

Color and State: Yellow oil; *Selectivity* **2y**/**3y** ≥19:1.

$[\alpha]_D^{20}$ –32.5 (c = 1.0, $CHCl_3$).

Chiral HPLC analysis of the *N*-tosyl derivative: 25 cm x 4.6 mm Chiralcel AD-H column, 96:3:1 hexane-isopropanol-methanol at 1.0 mL/min. flow rate, 230 nm, 28 °C; t_R(*minor*) 34.5 min., t_R(*major*) 36.4 min., 95% *ee*.

^{1}H NMR (500 MHz, $CDCl_3$) δ 7.58-7.56 (m, 4H), 7.41-7.40 (m, 6H), 5.83 (ddd, *J* = 17.0, 10.2, 6.6 Hz, 1H), 5.48-5.38 (m, 2H), 5.01 (d, *J* = 17.1 Hz, 1H), 4.88 (d, *J* = 10.1 Hz, 1H), 4.12 (app. t, *J* = 5.9 Hz, 1H), 1.57 (d, *J* = 5.2 Hz, 3H).

^{13}C NMR (125 MHz, $CDCl_3$) δ 143.22 (o), 142.37 (e), 142.22 (e), 135.50 (o), 130.41 (o), 130.38 (o), 129.09 (o), 127.84 (o), 127.75 (o), 124.23 (o), 112.64 (e), 65.68 (o), 17.94 (o).

IR (neat) 3055 (w), 3005 (w), 2963 (w), 2915 (m), 2853 (w), 1634 (w), 1580 (w), 1475 (m), 1442 (s) 1022 (s) cm^{-1}.

HRMS (ESI, $[M+H]^+$) calcd for $C_{18}H_{20}NS$ 282.1316, found 282.1328.

4. Experimental Procedures and Spectral Data for Hydrochloride Salt 6 and Amine 7

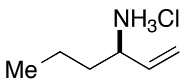

(*R*)-Hex-1-en-3-amine hydrochloride (6).[10]

Hydrochloric acid (1.23 mL, 14.9 mmol; 37% aqueous solution) was added to the allylic sulfilimine **2k** (1.01 g, 3.55 mmol) in acetonitrile (24.5 mL) at ambient temperature and stirred for *ca*. 3.5 hours (t.l.c. control). The reaction mixture was then concentrated *in vacuo* to afford the crude hydrochloride salt, which was redissolved in water and washed with dichloromethane. The pH of the aqueous phase was adjusted to pH 14 using aqueous 6N NaOH solution and partitioned with dichloromethane. The organic phases were combined, dried (anhyd. Na_2SO_4), filtered and the solution cooled with stirring to 0 °C. Hydrochloric acid (4.0 mL, 4.0 mmol; 1.0 M solution in diethyl ether) was then added dropwise and the solution concentrated *in vacuo* to afford the *hydrochloride salt* **6** (0.465 g, 94%) as a white solid: **mp** = 186-187 °C (lit.[10] mp = 189-190 °C); $[\alpha]_D^{23}$ –72.7 (c = 1.0, $CHCl_3$) {lit.[10] $[\alpha]_D$ –20.1 (c = 1.0)}.

Chiral HPLC analysis of the *N*-tosyl derivative:[11] 25 cm x 4.6 mm Chiralpak AD-H column, 96:3:1 hexane-isopropanol-methanol at 1.0 mL/min. flow rate, 230 nm, 28 °C; t_R(*minor*) 19.5 min., t_R(*major*) 21.6 min., 91% *ee*.

^{1}H NMR (500 MHz, $CDCl_3$) δ 8.54 (br. s, 3H), 5.87 (ddd, *J* = 17.3, 10.2, 7.6 Hz, 1H), 5.46 (d, *J* = 17.2 Hz, 1H), 5.37 (d, *J* = 10.5 Hz, 1H), 3.73-3.69 (m, 1H), 1.86 (ddt, *J* = 13.3, 9.1, 6.6 Hz, 1H), 1.73 (dtd, *J* = 14.3, 8.6, 6.3 Hz, 1H), 1.47-1.37 (m, 2H), 0.94 (t, *J* = 7.3 Hz, 3H).

---

[10] Ringdahl, B.; Dahlbom, R. *Chem. Scripta* **1977**, *12*, 47.

^{13}C NMR (125 MHz, $CDCl_3$) δ 133.62 (o), 120.74 (e), 54.60 (o), 35.26 (e), 18.79 (e), 13.66 (o).

IR (neat) 3300-2500 (vs, br.), 2859 (vs), 2549 (m), 1615 (m), 1511 (s), 1423 (s), 993 (s), 929 (vs) cm^{-1}.

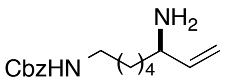

(*R*)-Benzyl (6-aminooct-7-en-1-yl)carbamate (7).

The sulfilimine **2w** was prepared from the allylic benzoate **1w** as described in the representative procedure for the iridium-catalyzed allylic amination reaction using *in-situ* catalyst activation (Method B). Hydrochloric acid (0.3 mL, 3.05 mmol; 37% aqueous solution) was added to the reaction mixture diluted with acetonitrile (1.75 mL) at ambient temperature. The reaction was stirred at this temperature for *ca*. 7 hours (t.l.c. control) and concentrated *in vacuo* to afford the crude hydrochloride salt, which was redissolved in water and washed with dichloromethane. The pH of the aqueous phase was then adjusted to pH 14 using aqueous 6N NaOH solution and partitioned with dichloromethane. The organic phases were combined, dried (anhyd. Na_2SO_4), filtered and concentrated *in vacuo* to afford the *allylic amine* **7** (53.0 mg, 76%,) as a colorless oil: *Selectivity b/l* ≥19:1; $[\alpha]_D^{23}$ –56.0 (c = 1.1, $CHCl_3$).

Chiral HPLC analysis of the *N*-tosyl derivative[11]: 25 cm x 4.6 mm Chiralcel AD-H column, 91:8:1 hexane-isopropanol-methanol at 1.0 mL/min. flow rate, 230 nm, 28 °C; t_R(*minor*) 77.5 min., t_R(*major*) 96.6 min., 91% *ee.*

^{1}H NMR (500 MHz, $CDCl_3$) δ 7.34-7.33 (m, 4H), 7.32-7.27 (m, 1H), 5.75 (ddd, *J* = 17.1, 10.2, 6.8 Hz, 1H), 5.08 (s, 2H), 5.07 (d, *J* = 17.0 Hz, 1H), 4.99 (d, *J* = 10.3 Hz, 1H), 4.86 (br. s, 1H), 3.24 (app. q, *J* = 6.2 Hz, 1H), 3.17 (app. q, *J* = 6.3 Hz, 2H), 1.52-1.49 (m, 2H), 1.41-1.33 (m, 6H), 1.18 (s, 2H).

^{13}C NMR (125 MHz, $CDCl_3$) δ 156.49 (e), 143.63 (o), 136.80 (e), 128.58 (o), 128.18 (o), 128.14 (o), 113.37 (e), 66.63 (e), 54.51 (o), 41.08 (e), 37.55 (e), 30.00 (e), 26.79 (e), 25.76 (e).

IR (neat) 3339 (w; br.), 2925 (m), 2857 (m), 1701 (vs), 1451 (m), 1246 (vs), 1131 (s), 998 (s), 691 (vs) cm^{-1}.

HRMS (EI, M^+) calcd for $C_{16}H_{24}N_2O_2$ 276.1838, found 276.1832.

[11] P. G. M. Wuts, T. W. Greene *Protective Groups in Organic Synthesis*, Wiley, Hoboken, 4th edn, 2007.

5. Copies of NMR Spectra

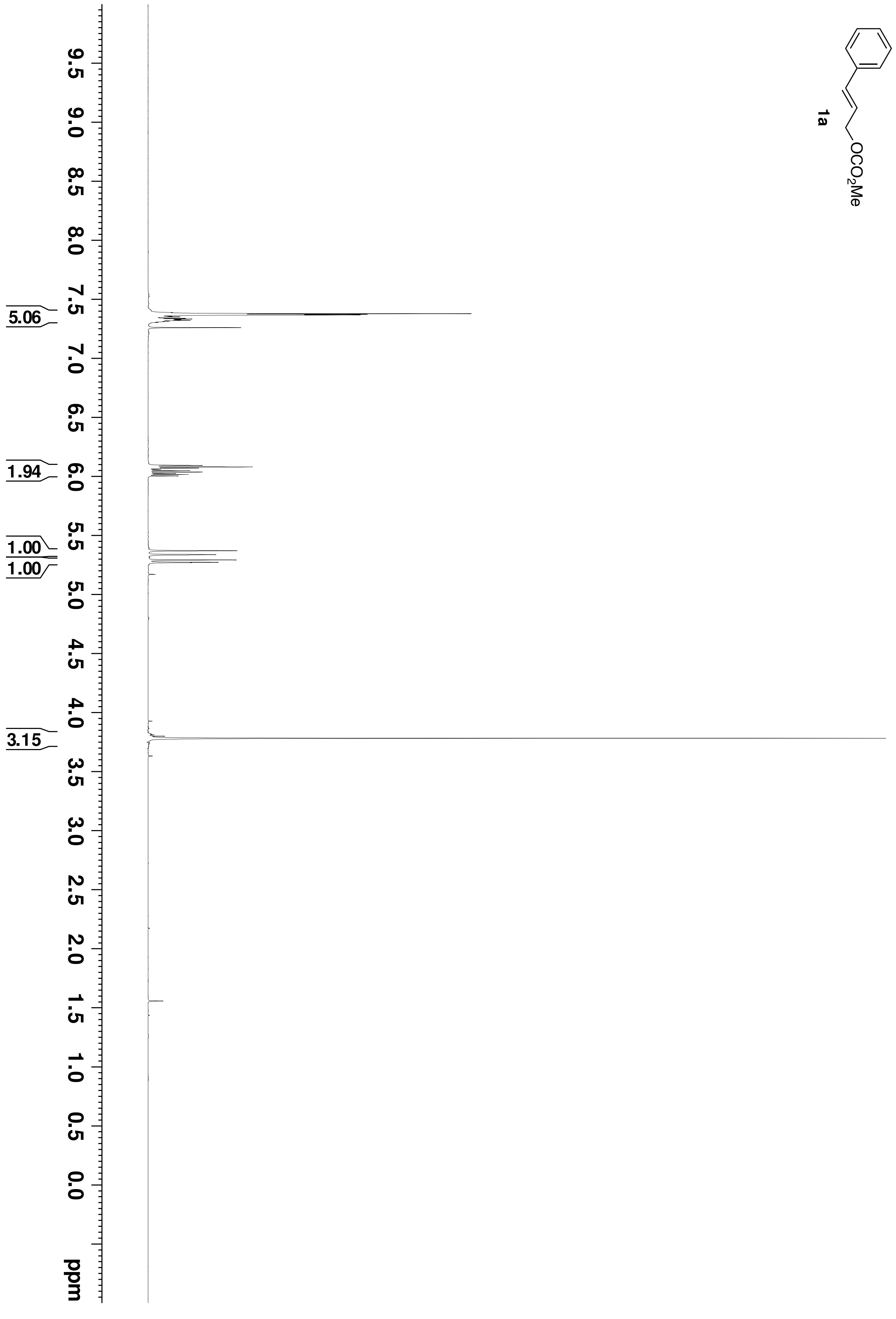

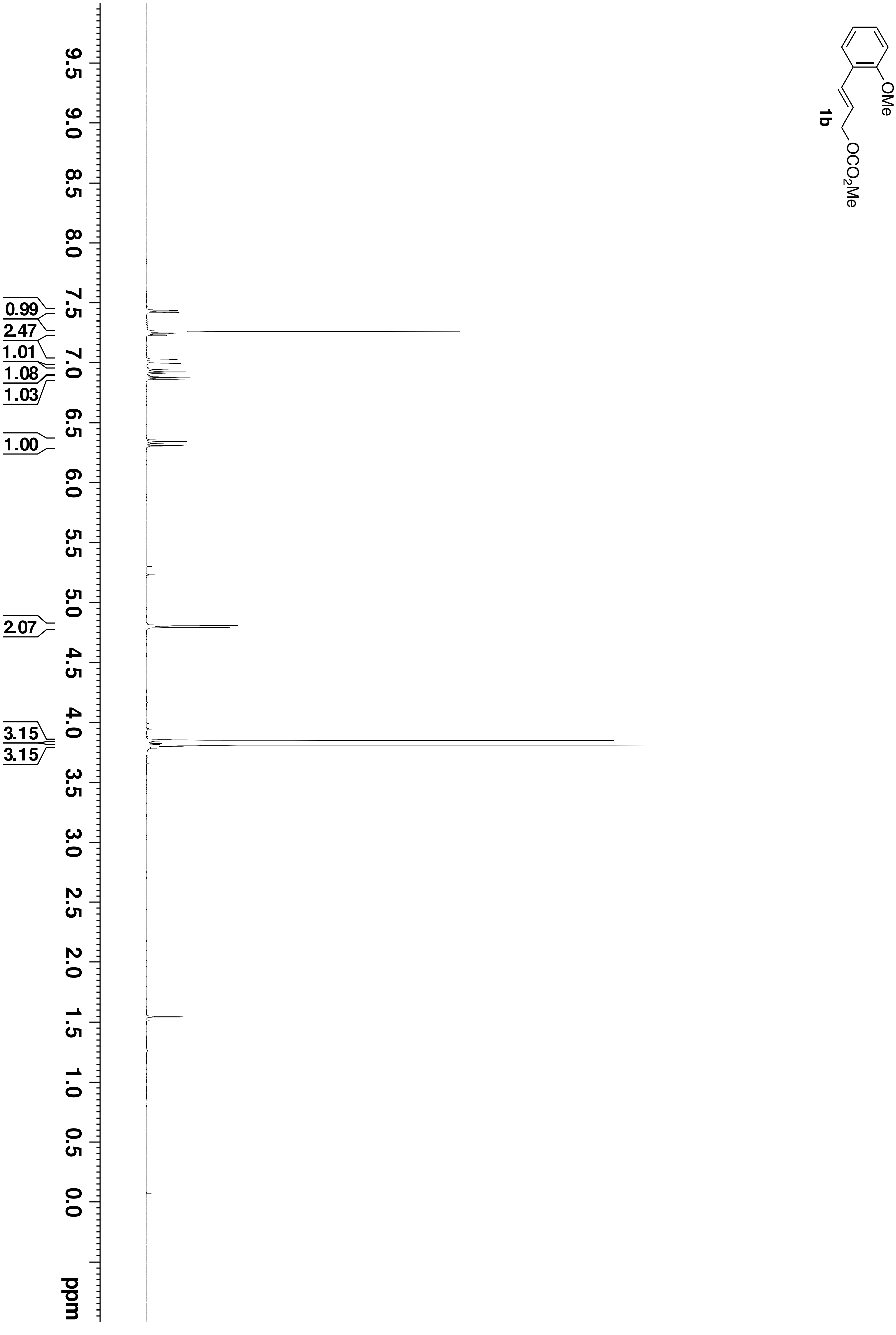
OMe
OCO$_2$Me
1b
9.5
9.0
8.5
8.0
7.5
7.0
6.5
6.0
5.5
5.0
4.5
4.0
3.5
3.0
2.5
2.0
1.5
1.0
0.5
0.0
ppm
0.99
2.47
1.01
1.08
1.03
1.00
2.07
3.15
3.15

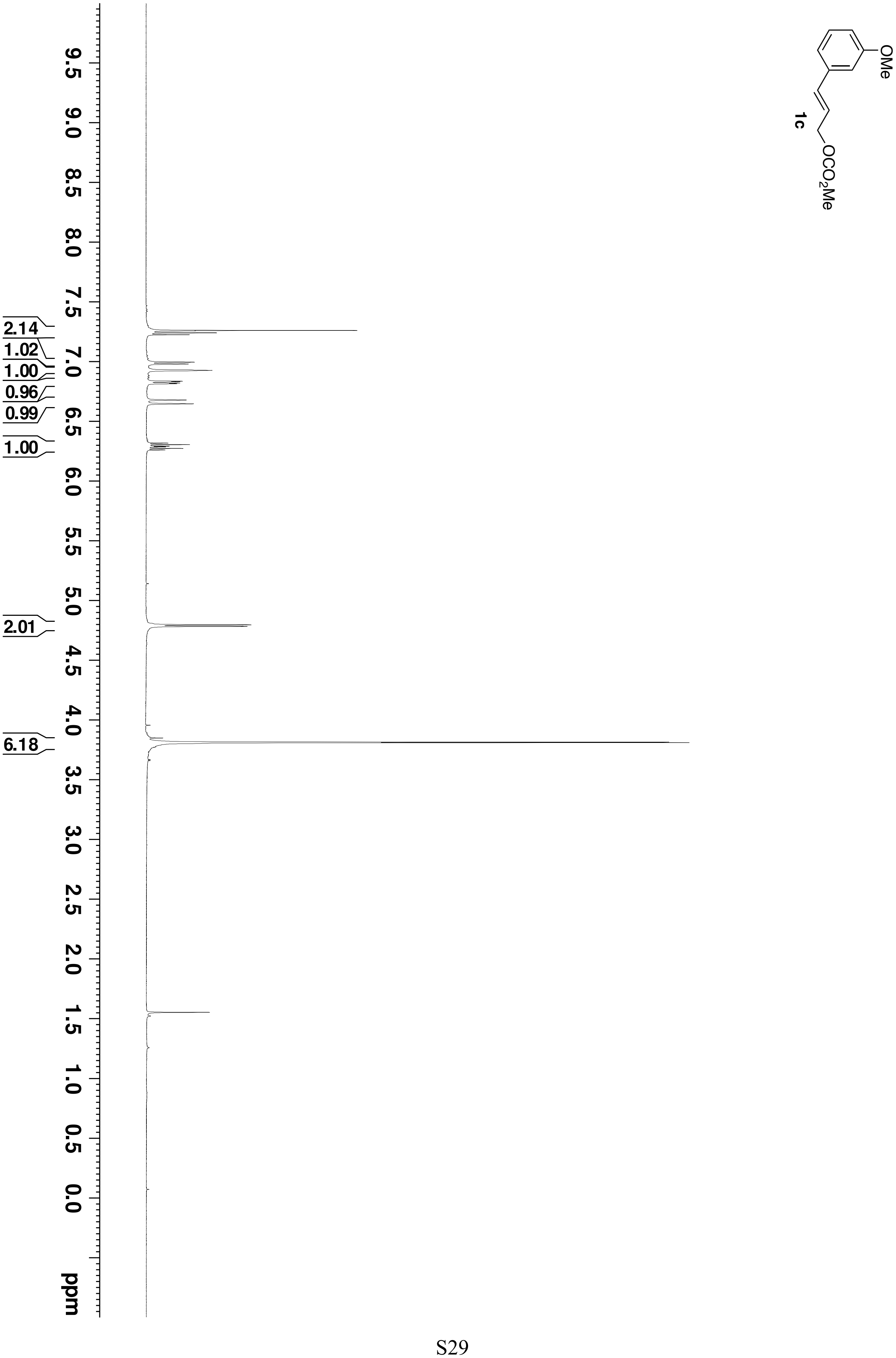
OMe
OCO2Me
1c
9.5
9.0
8.5
8.0
7.5
7.0
6.5
6.0
5.5
5.0
4.5
4.0
3.5
3.0
2.5
2.0
1.5
1.0
0.5
0.0
ppm
2.14
1.02
1.00
0.96
0.99
1.00
2.01
6.18

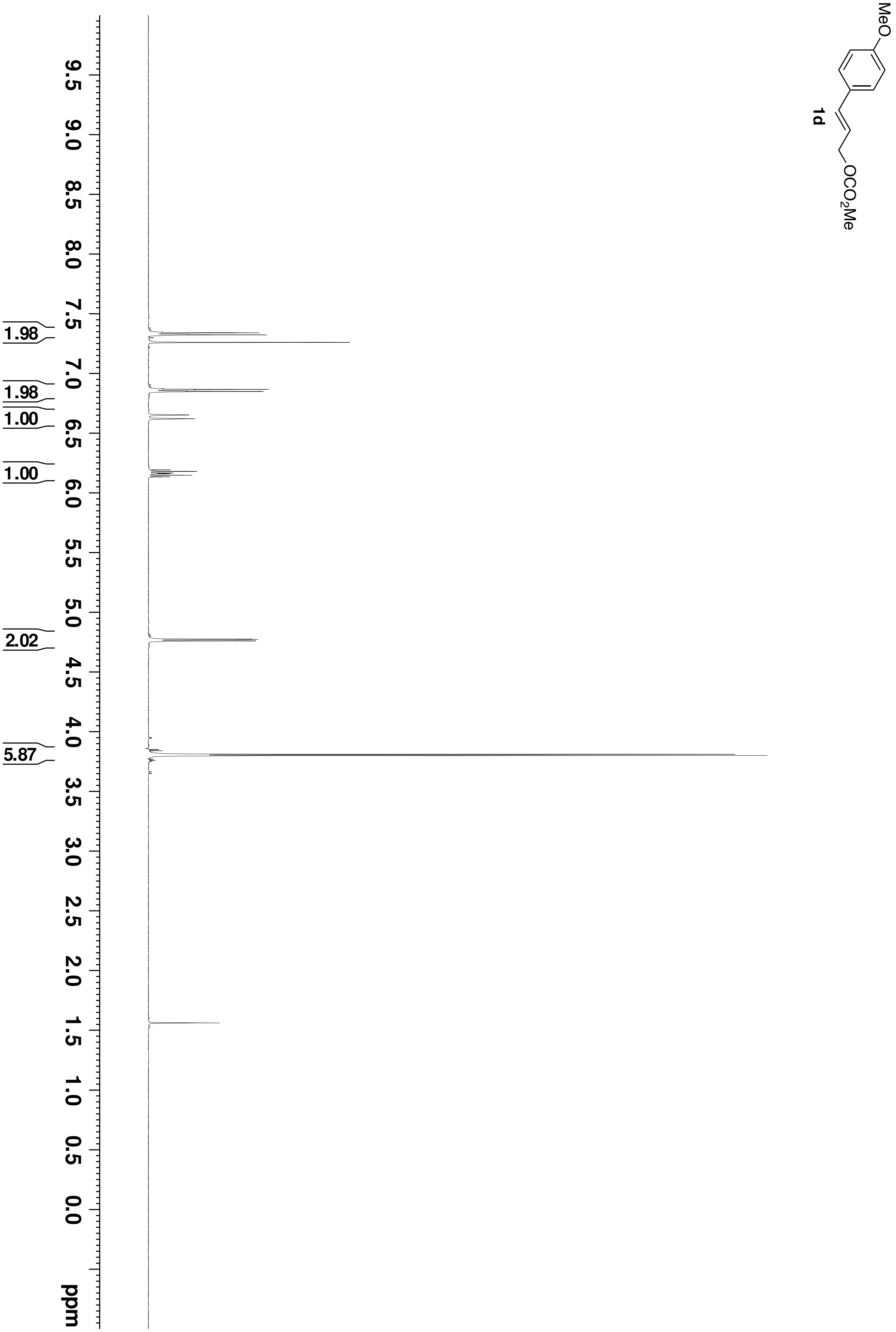
MeO
OCO2Me
1d
1.98
1.98
1.00
1.00
2.02
5.87
9.5 9.0 8.5 8.0 7.5 7.0 6.5 6.0 5.5 5.0 4.5 4.0 3.5 3.0 2.5 2.0 1.5 1.0 0.5 0.0 ppm

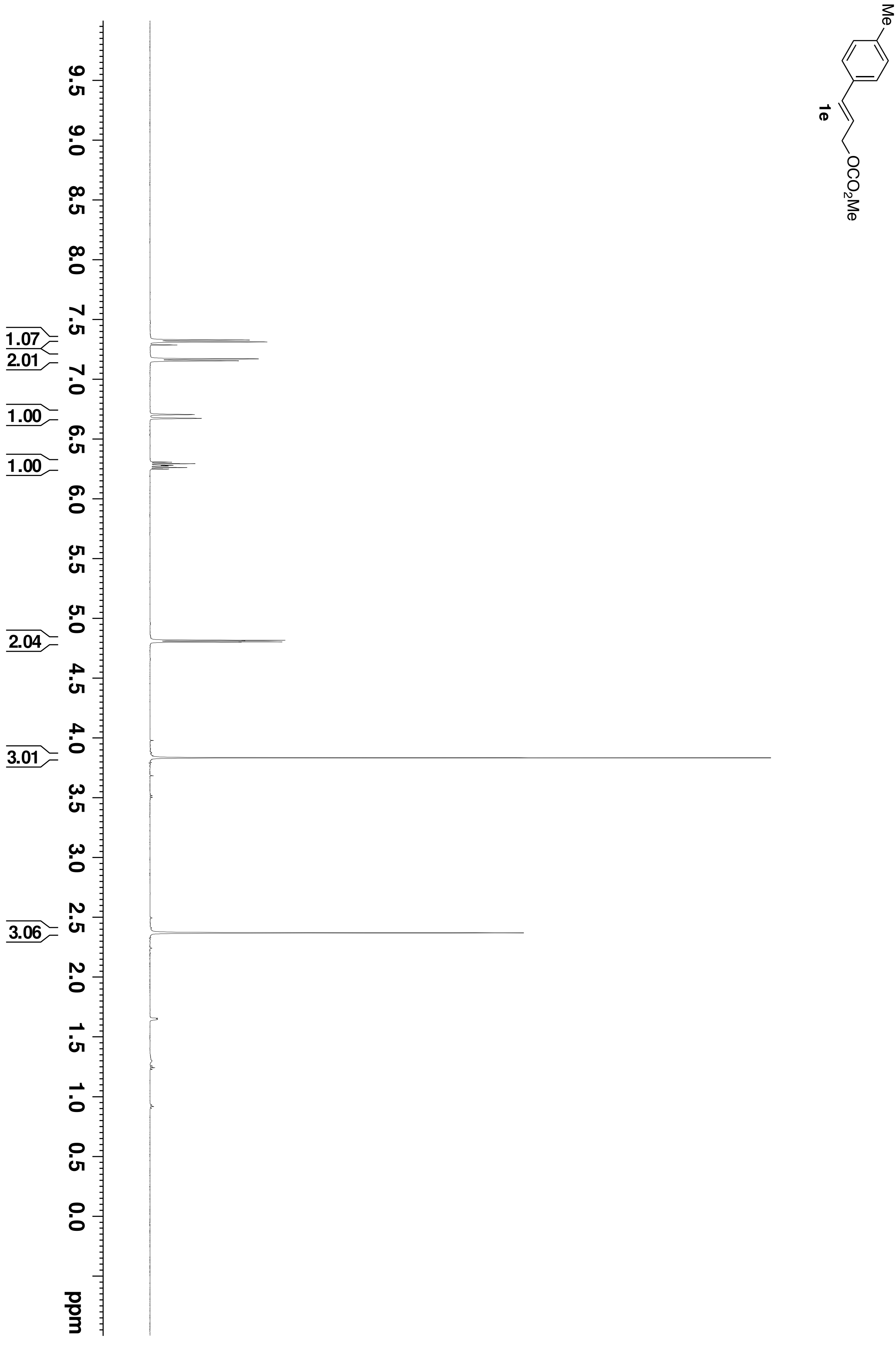
Me
1e
OCO2Me
1.07
2.01
1.00
1.00
2.04
3.01
3.06
9.5
9.0
8.5
8.0
7.5
7.0
6.5
6.0
5.5
5.0
4.5
4.0
3.5
3.0
2.5
2.0
1.5
1.0
0.5
0.0
ppm

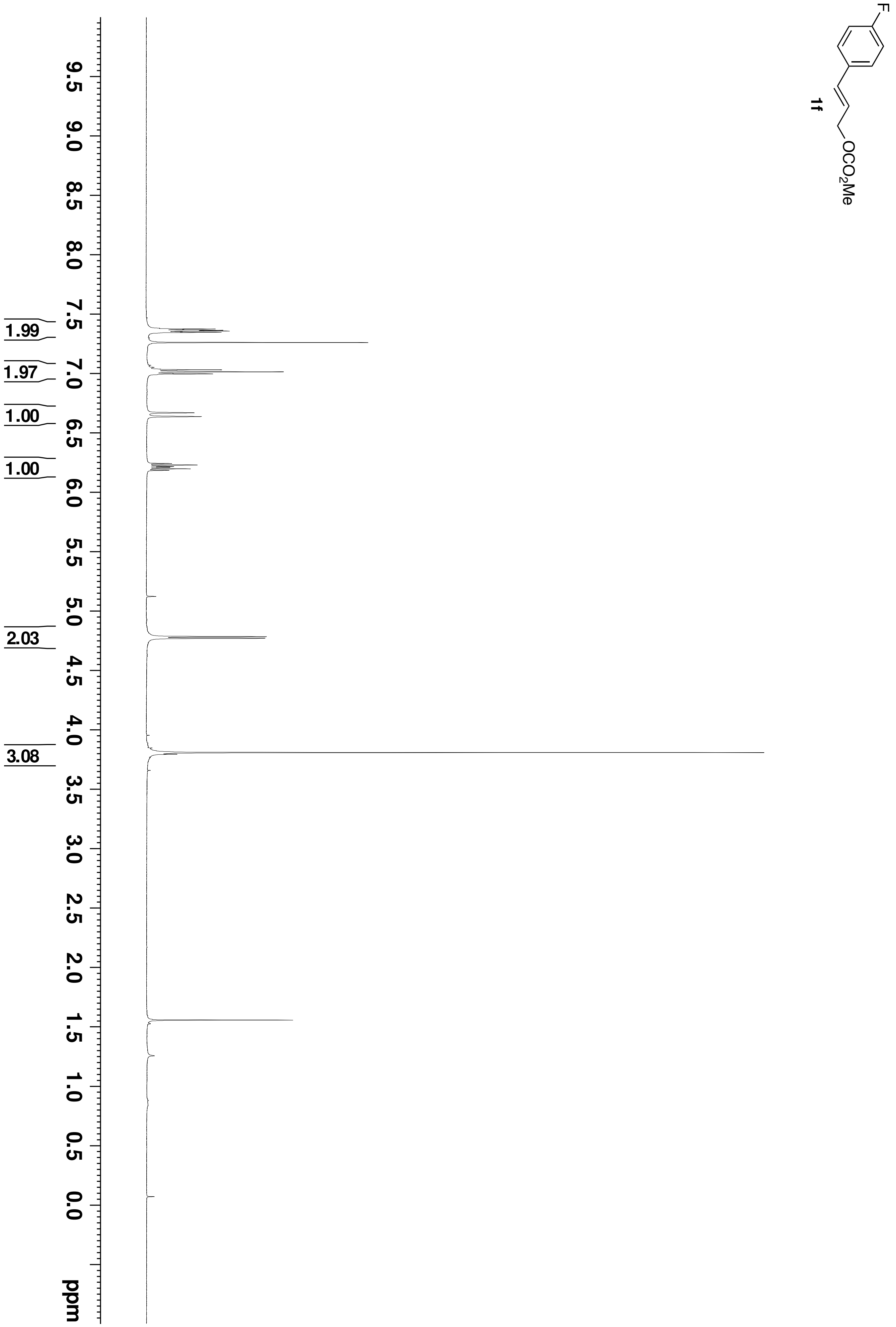
F
OCO2Me
1f
1.99
1.97
1.00
1.00
2.03
3.08
9.5
9.0
8.5
8.0
7.5
7.0
6.5
6.0
5.5
5.0
4.5
4.0
3.5
3.0
2.5
2.0
1.5
1.0
0.5
0.0
ppm

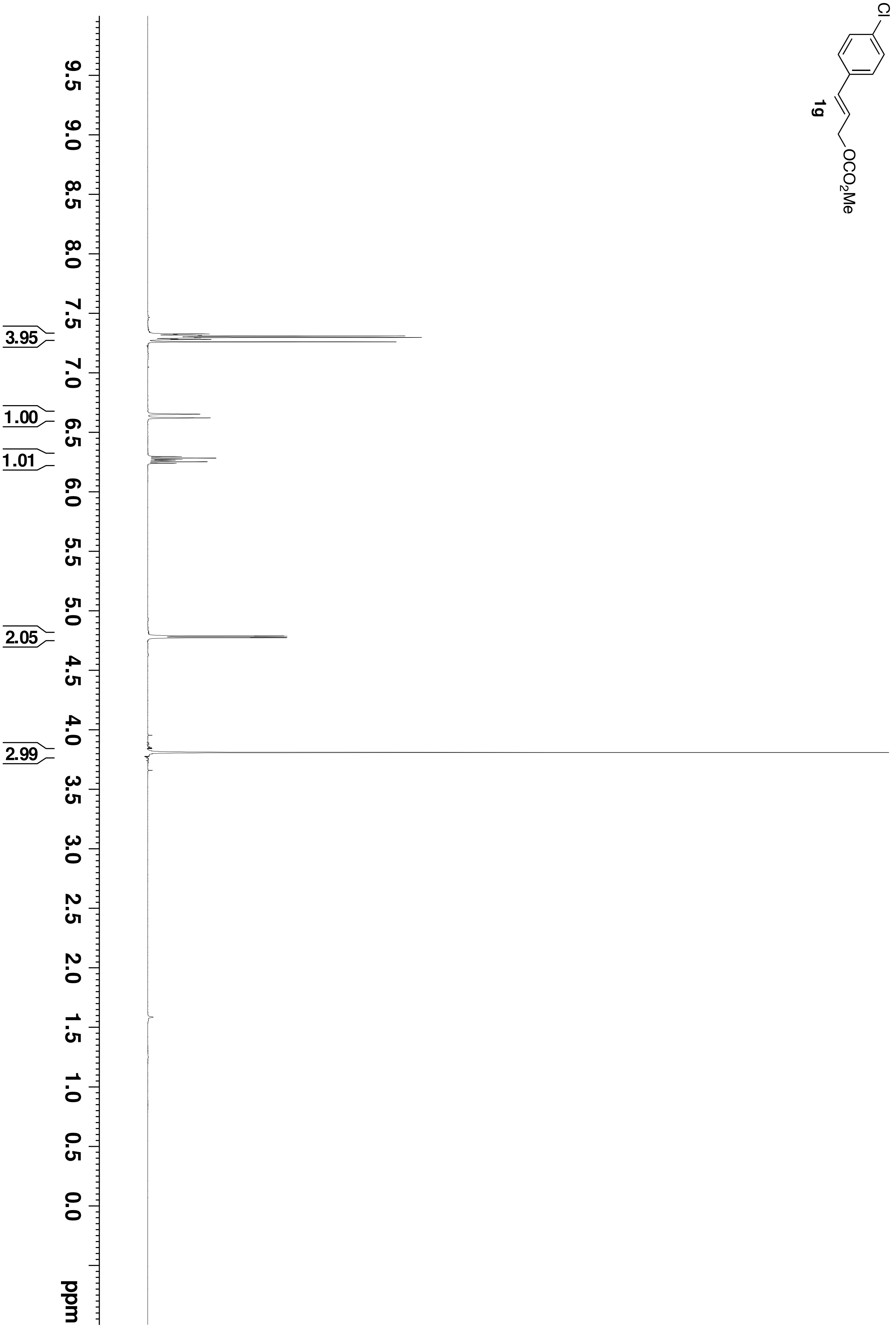
Cl
OCO2Me
1g
9.5
9.0
8.5
8.0
7.5
7.0
6.5
6.0
5.5
5.0
4.5
4.0
3.5
3.0
2.5
2.0
1.5
1.0
0.5
0.0
ppm
3.95
1.00
1.01
2.05
2.99

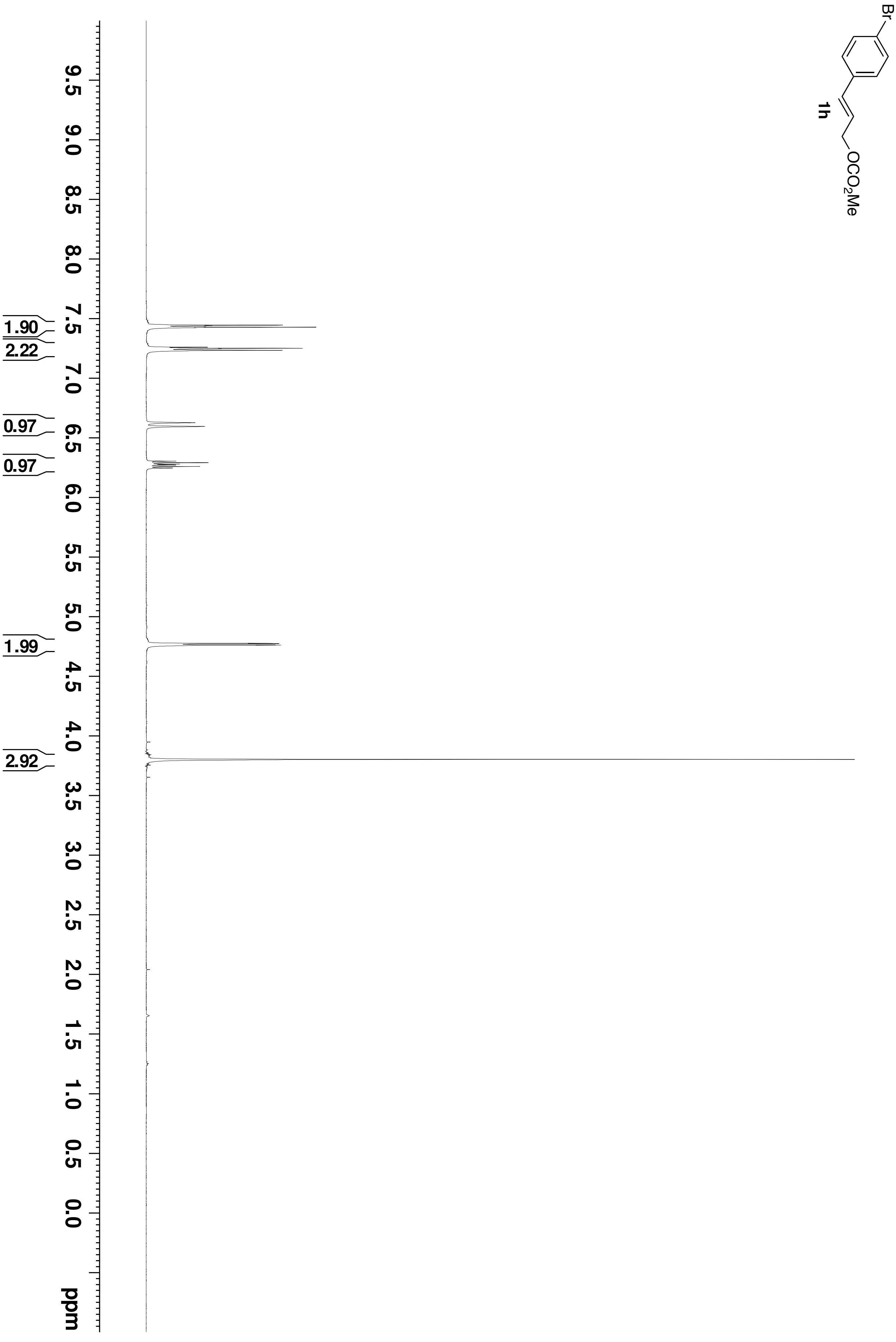

Br
OCO2Me
1h
1.90
2.22
0.97
0.97
1.99
2.92
9.5
9.0
8.5
8.0
7.5
7.0
6.5
6.0
5.5
5.0
4.5
4.0
3.5
3.0
2.5
2.0
1.5
1.0
0.5
0.0
ppm

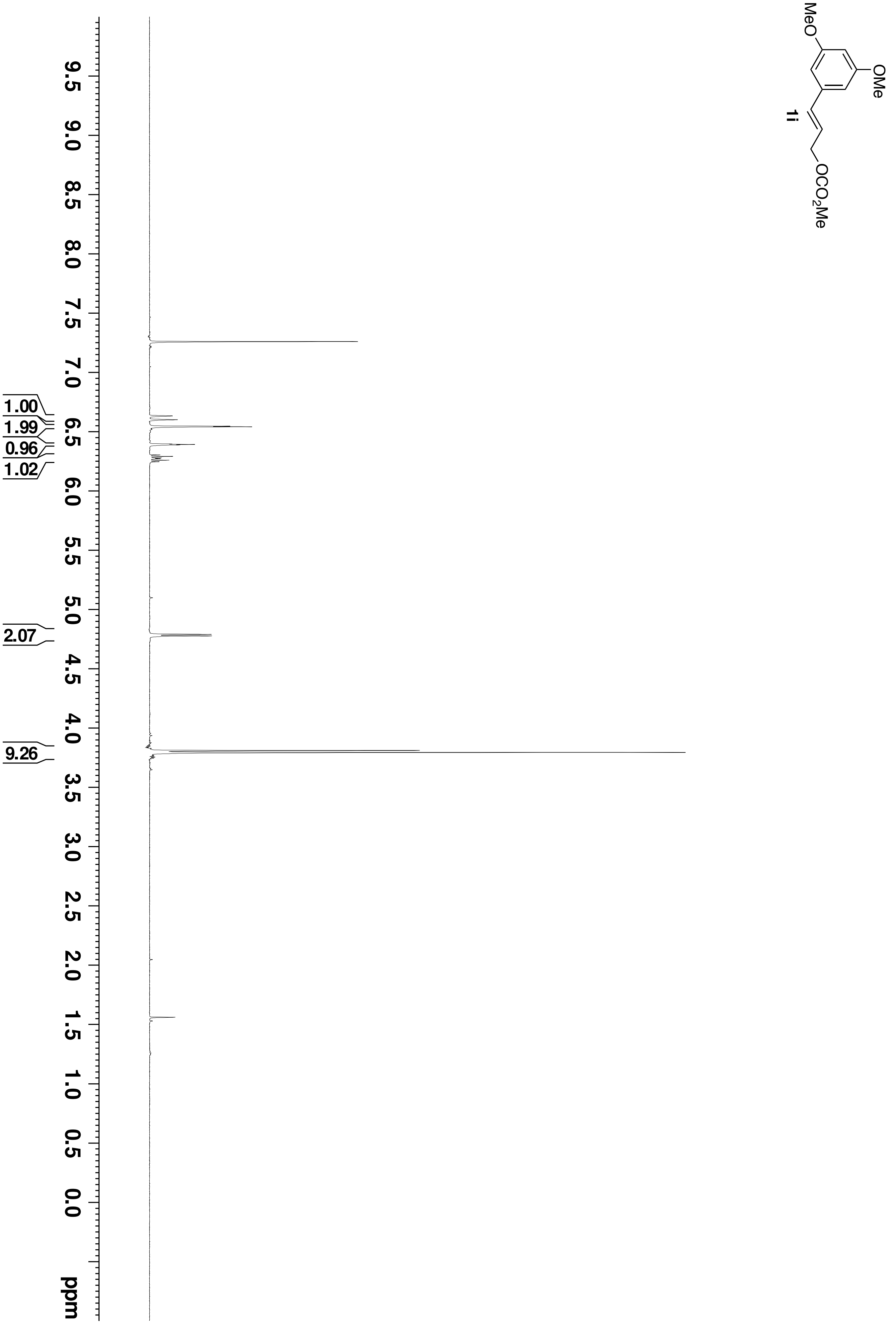
MeO
OMe
OCO2Me
1i
1.00
1.99
0.96
1.02
2.07
9.26
9.5 9.0 8.5 8.0 7.5 7.0 6.5 6.0 5.5 5.0 4.5 4.0 3.5 3.0 2.5 2.0 1.5 1.0 0.5 0.0 ppm

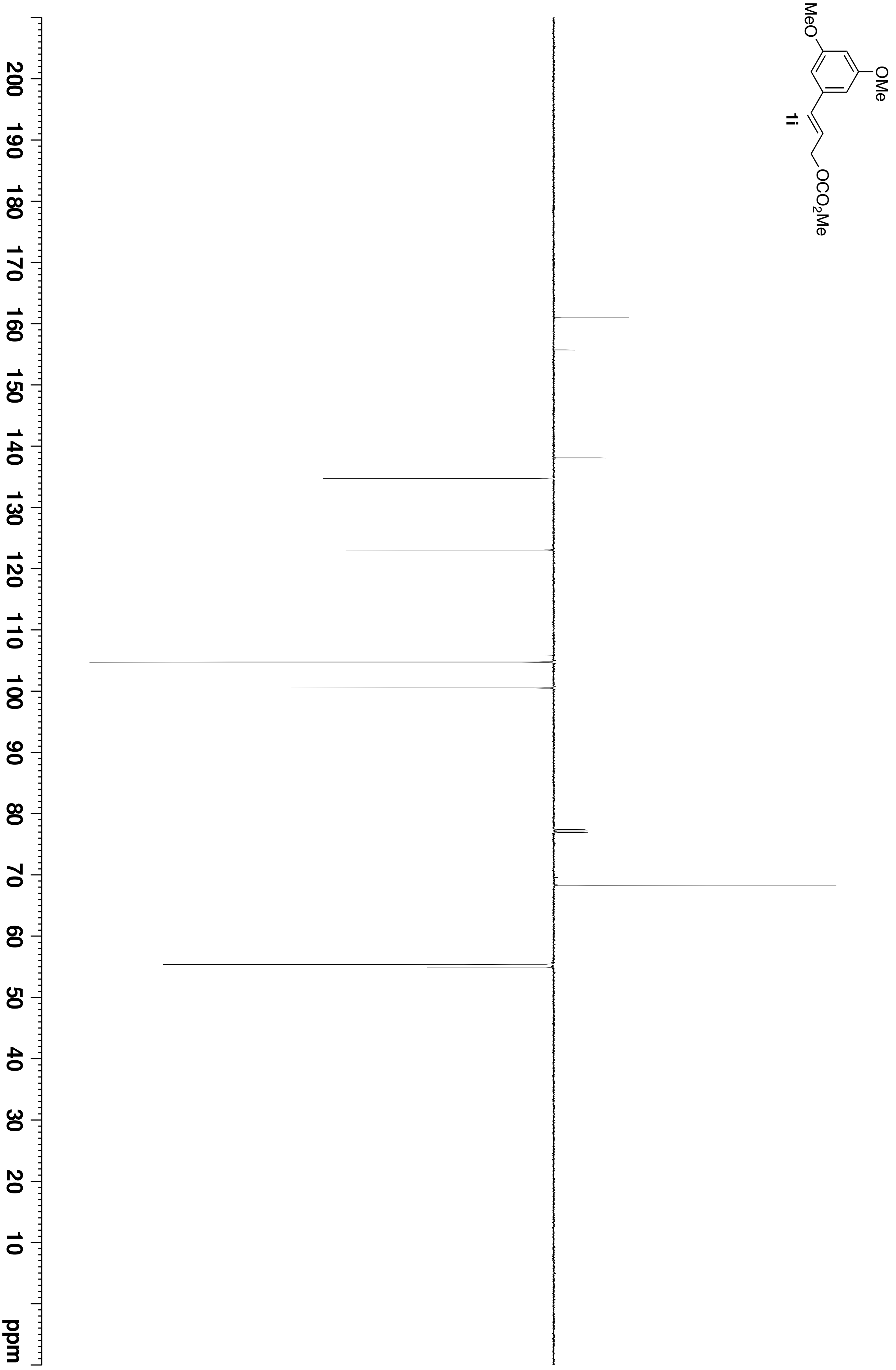
MeO
OMe
OCO2Me
1i
200
190
180
170
160
150
140
130
120
110
100
90
80
70
60
50
40
30
20
10
ppm

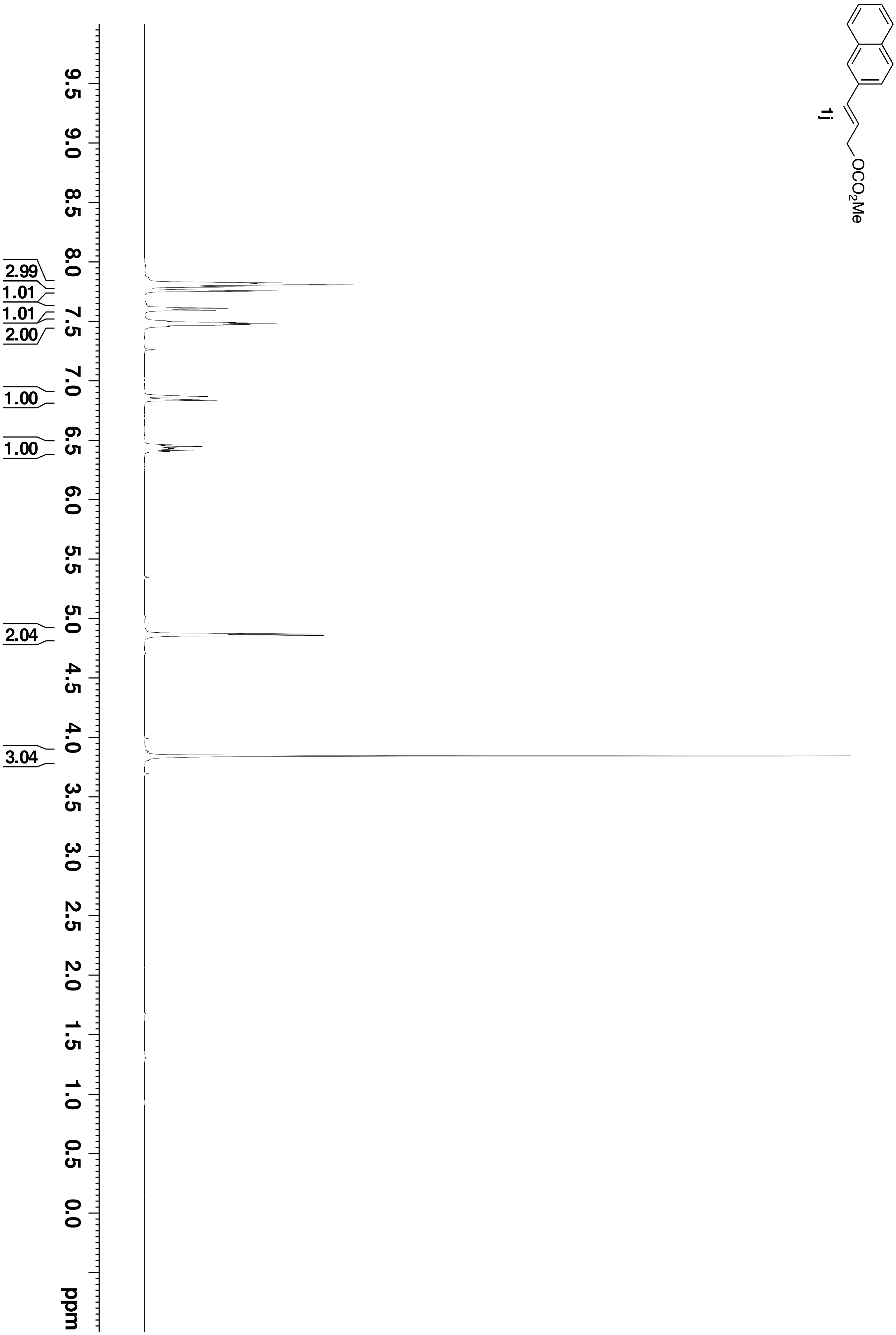

1j
OCO2Me
2.99
1.01
1.01
2.00
1.00
1.00
2.04
3.04
9.5
9.0
8.5
8.0
7.5
7.0
6.5
6.0
5.5
5.0
4.5
4.0
3.5
3.0
2.5
2.0
1.5
1.0
0.5
0.0
ppm

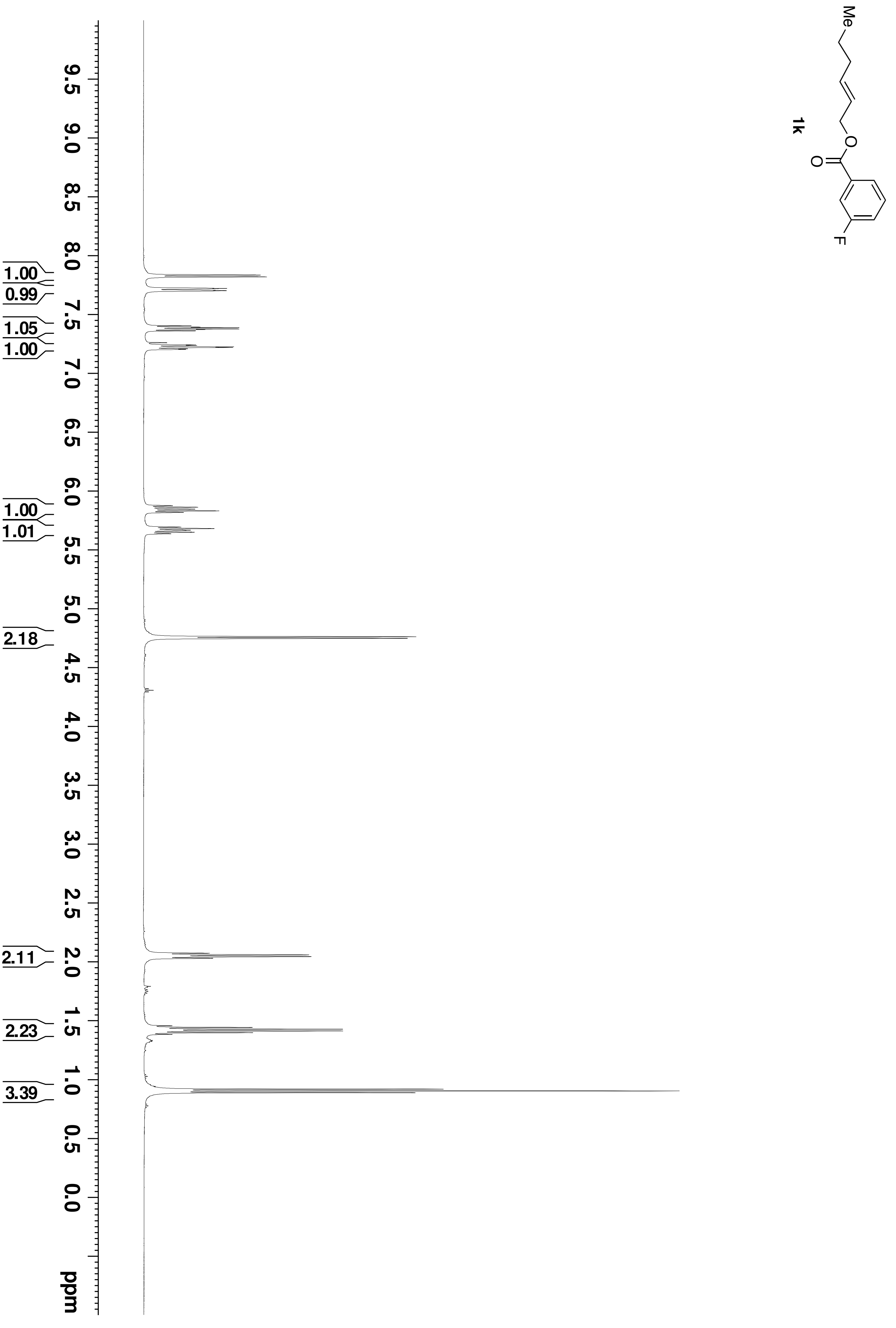

1k
Me
O
O
F
9.5
9.0
8.5
8.0
7.5
7.0
6.5
6.0
5.5
5.0
4.5
4.0
3.5
3.0
2.5
2.0
1.5
1.0
0.5
0.0
ppm
1.00
0.99
1.05
1.00
1.00
1.01
2.18
2.11
2.23
3.39

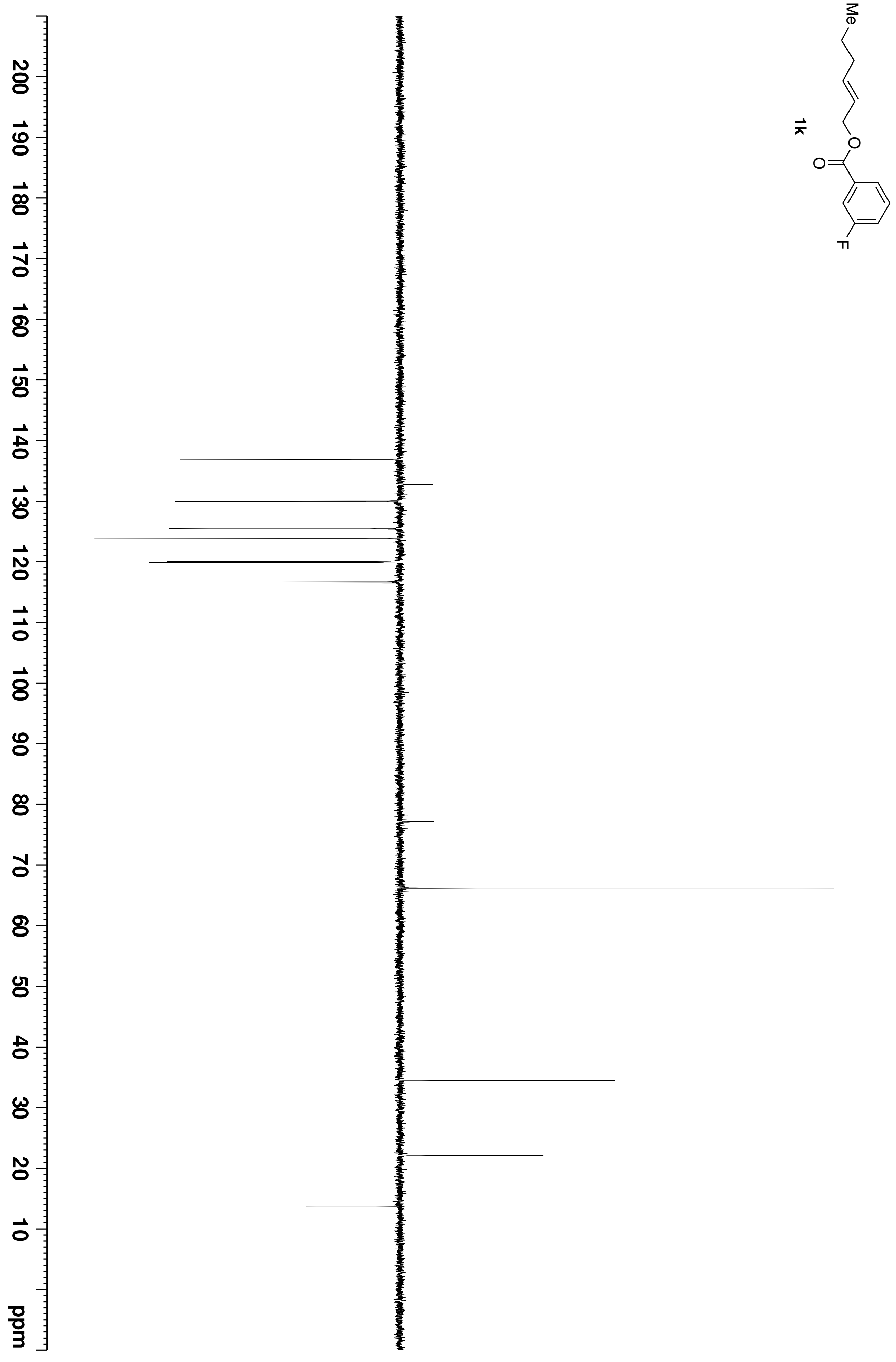

Me
O
O
F
1k
200
190
180
170
160
150
140
130
120
110
100
90
80
70
60
50
40
30
20
10
ppm

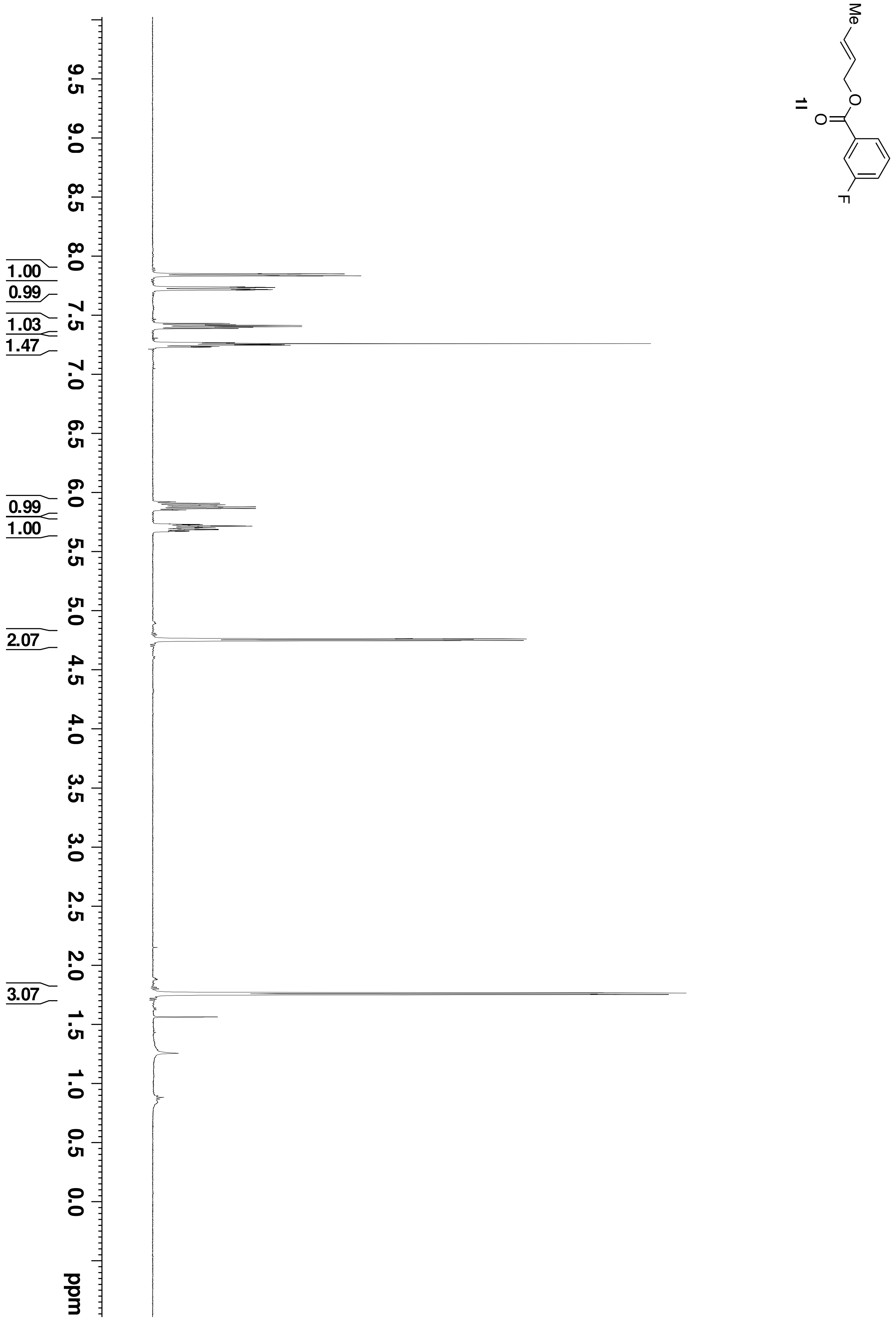
Me
O
O
F
1l
9.5
9.0
8.5
8.0
7.5
7.0
6.5
6.0
5.5
5.0
4.5
4.0
3.5
3.0
2.5
2.0
1.5
1.0
0.5
0.0
ppm
1.00
0.99
1.03
1.47
0.99
1.00
2.07
3.07

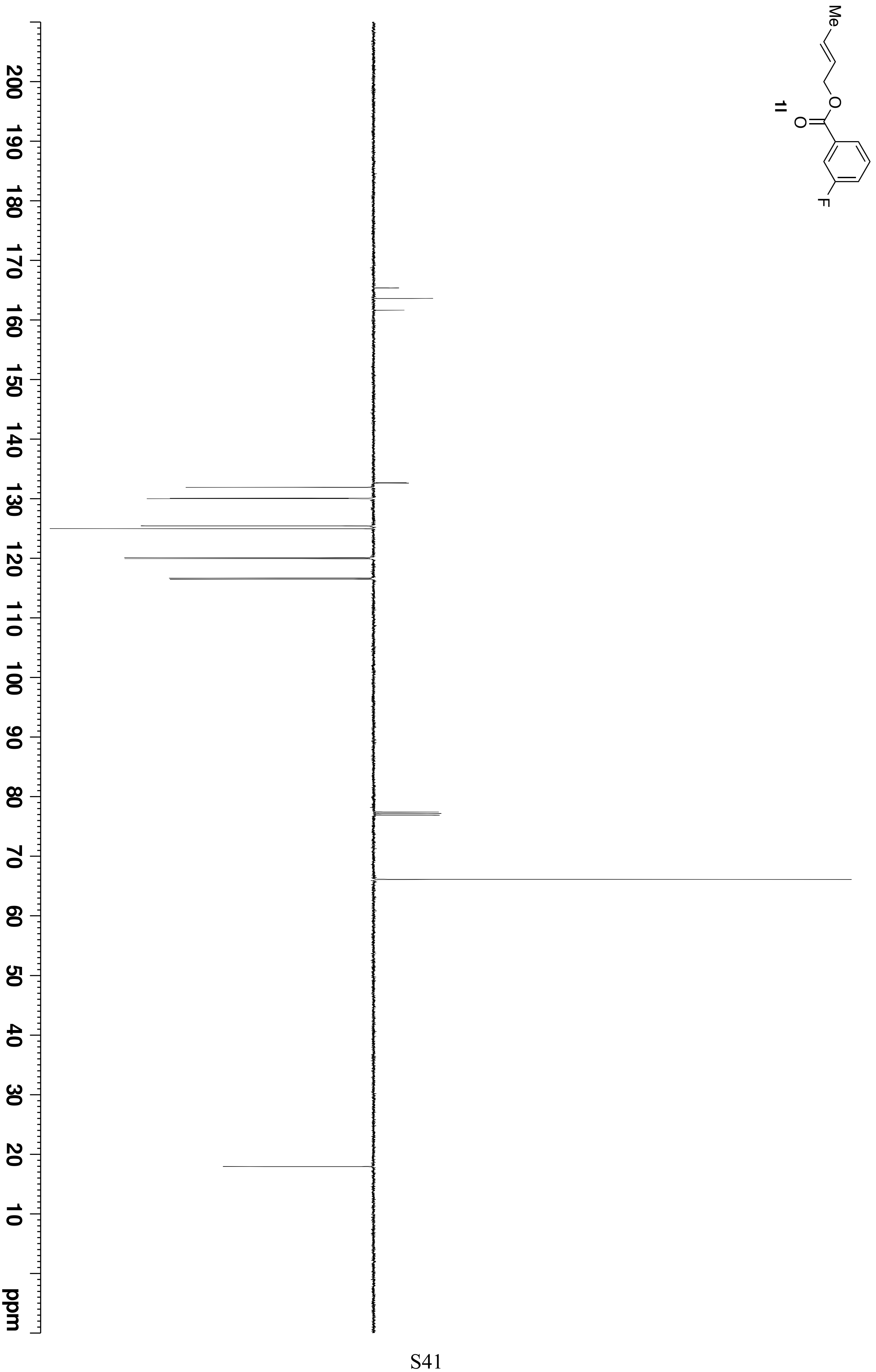

Me
O
O
F
1l
200
190
180
170
160
150
140
130
120
110
100
90
80
70
60
50
40
30
20
10
ppm

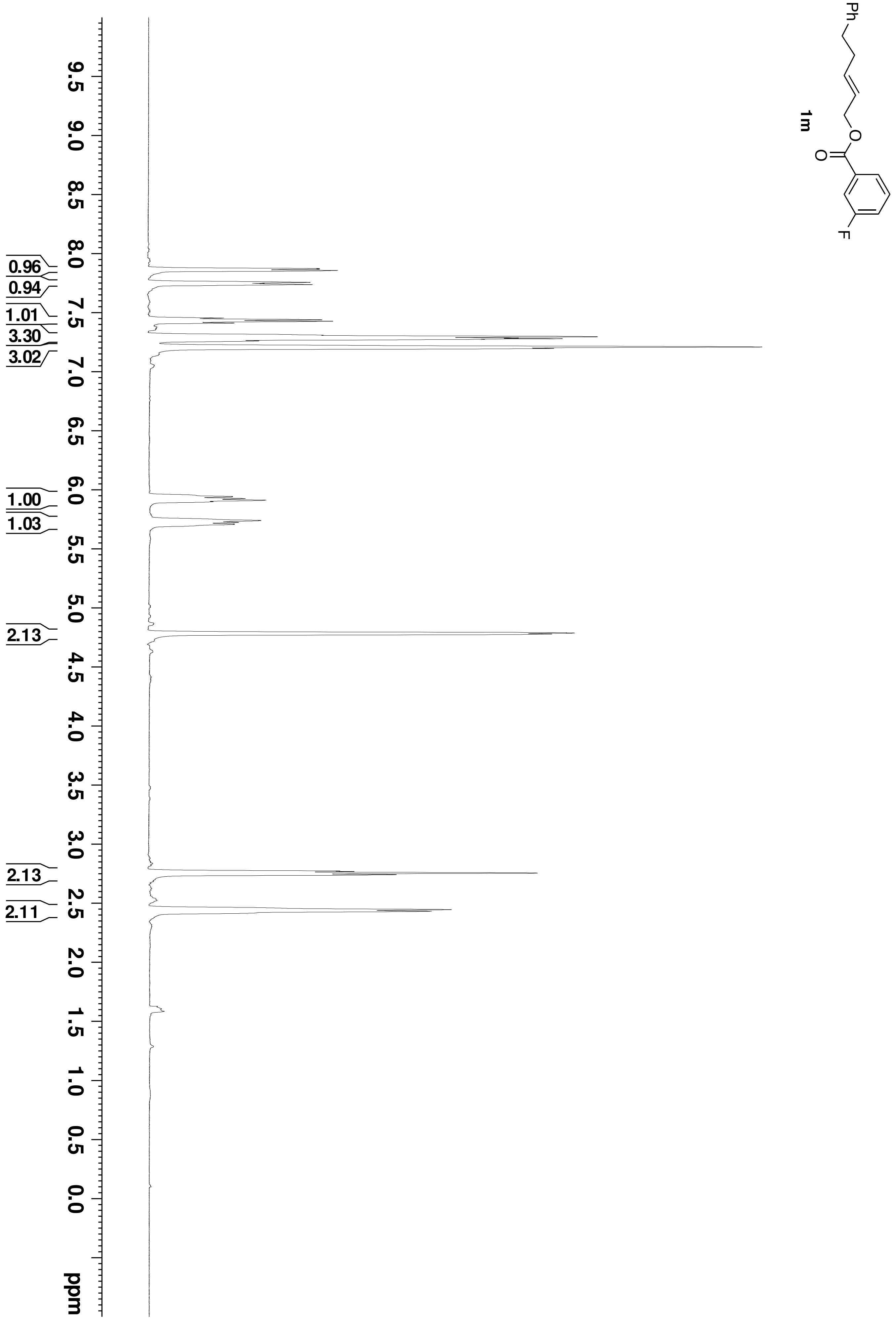

1m
Ph
O
F
0.96
0.94
1.01
3.30
3.02
1.00
1.03
2.13
2.13
2.11
9.5
9.0
8.5
8.0
7.5
7.0
6.5
6.0
5.5
5.0
4.5
4.0
3.5
3.0
2.5
2.0
1.5
1.0
0.5
0.0
ppm

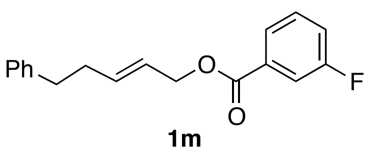
Ph
O
O
F

1m

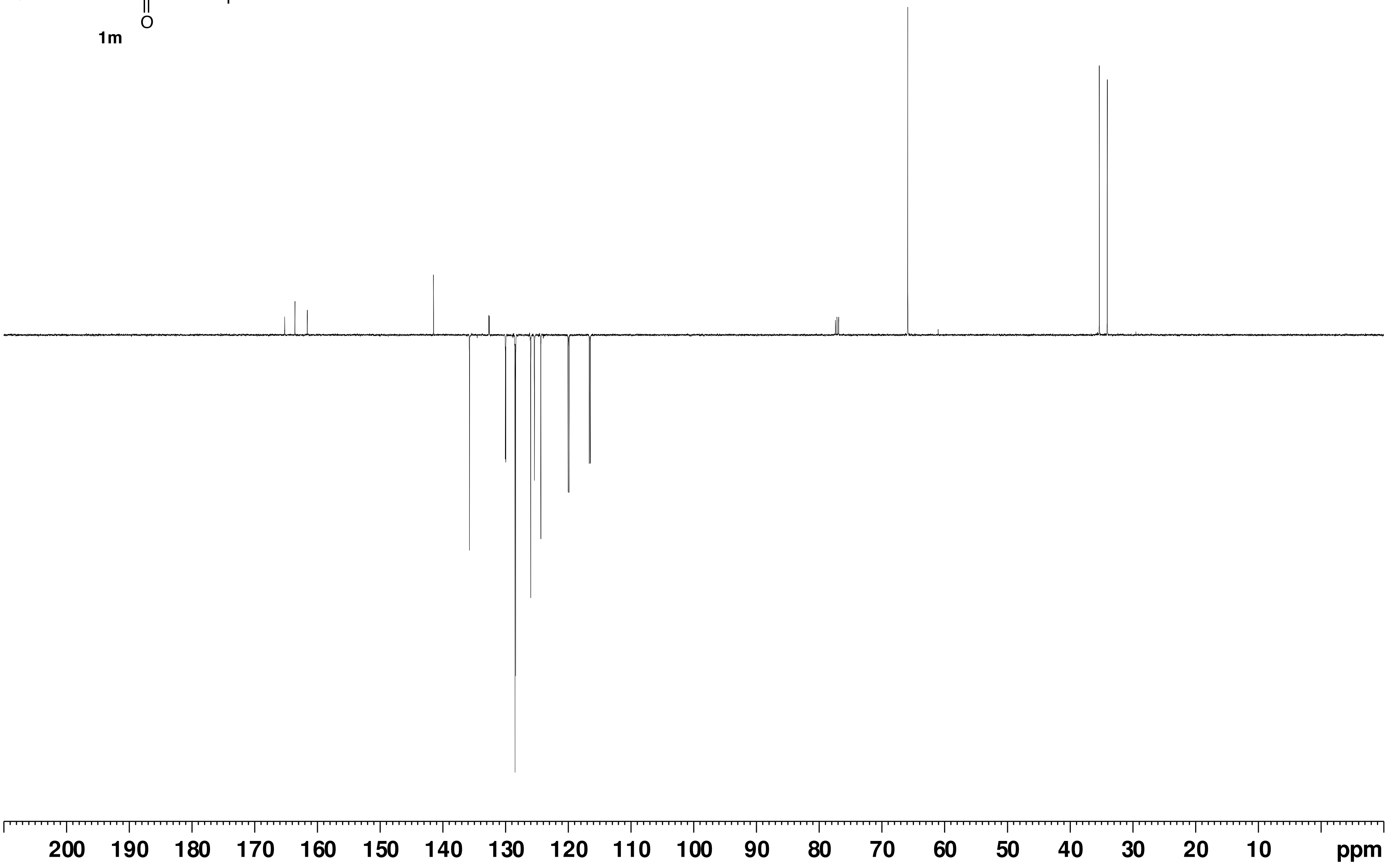
200 190 180 170 160 150 140 130 120 110 100 90 80 70 60 50 40 30 20 10 ppm

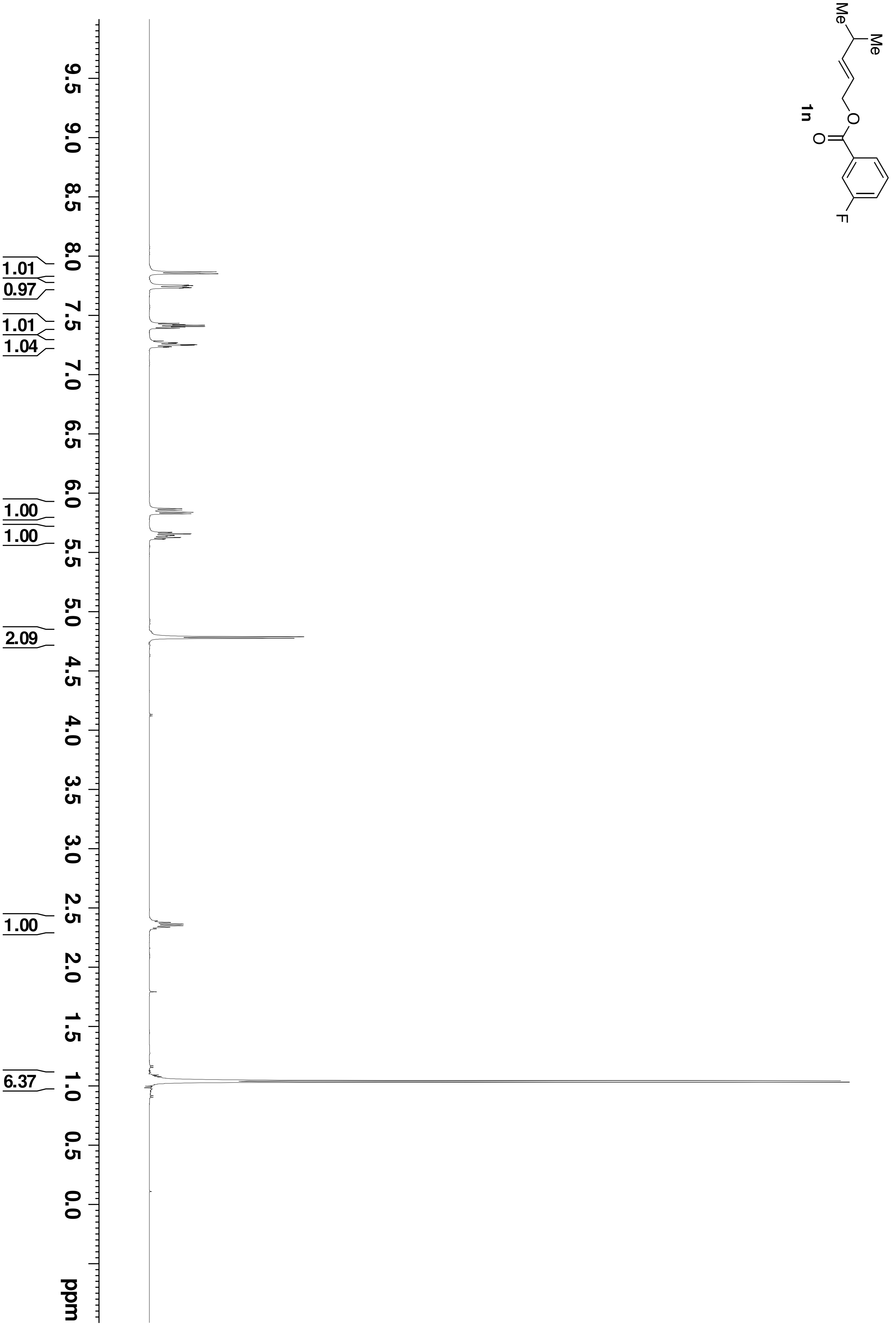

Me
Me
O
O
F
1n
9.5
9.0
8.5
8.0
7.5
7.0
6.5
6.0
5.5
5.0
4.5
4.0
3.5
3.0
2.5
2.0
1.5
1.0
0.5
0.0
ppm
1.01
0.97
1.01
1.04
1.00
1.00
2.09
1.00
6.37

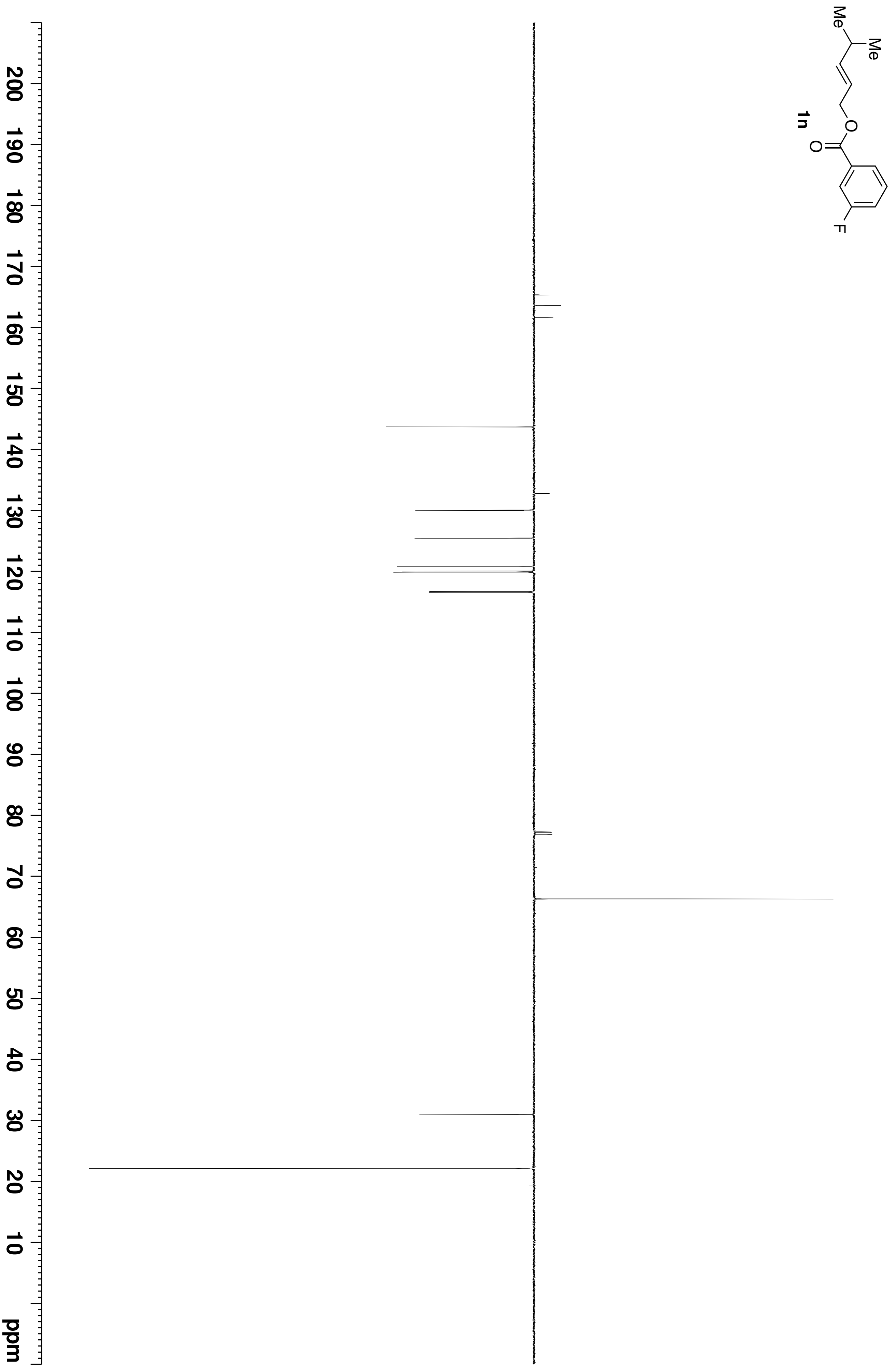

Me
Me
O
O
F
1n
200
190
180
170
160
150
140
130
120
110
100
90
80
70
60
50
40
30
20
10
ppm

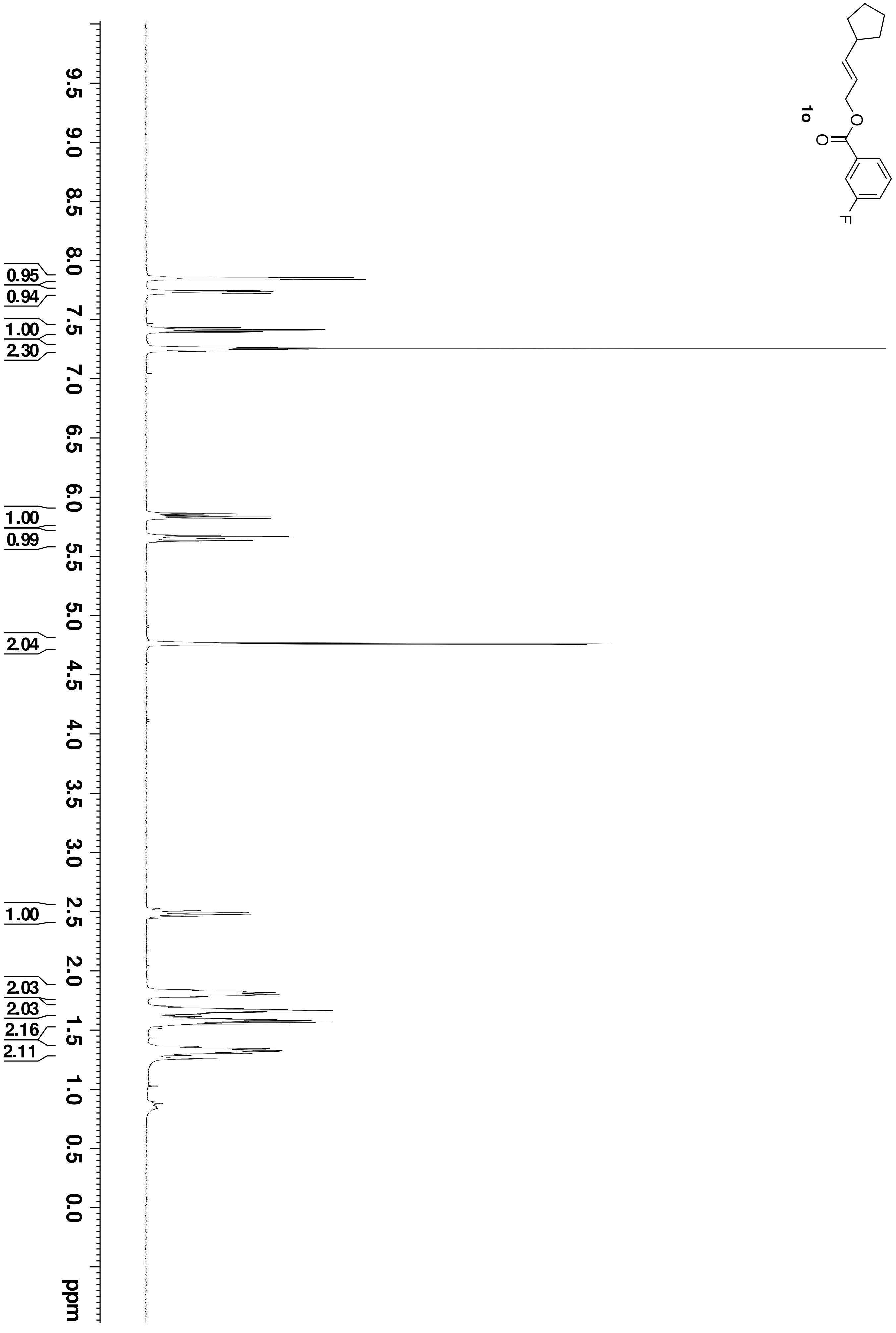
1o
F
O
O
0.95
0.94
1.00
2.30
1.00
0.99
2.04
1.00
2.03
2.03
2.16
2.11
9.5
9.0
8.5
8.0
7.5
7.0
6.5
6.0
5.5
5.0
4.5
4.0
3.5
3.0
2.5
2.0
1.5
1.0
0.5
0.0
ppm

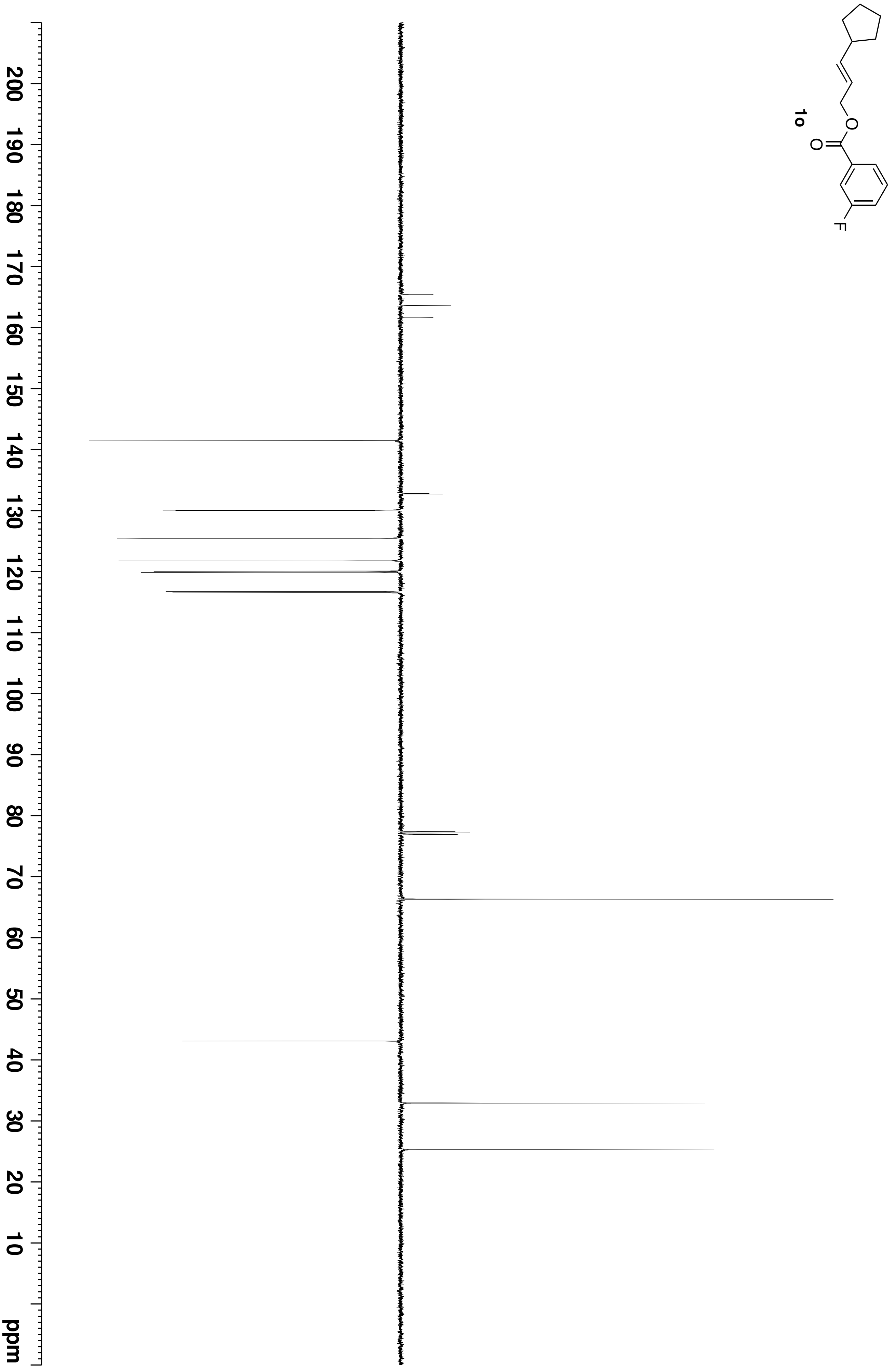

1o
F
O
O
200 190 180 170 160 150 140 130 120 110 100 90 80 70 60 50 40 30 20 10
ppm

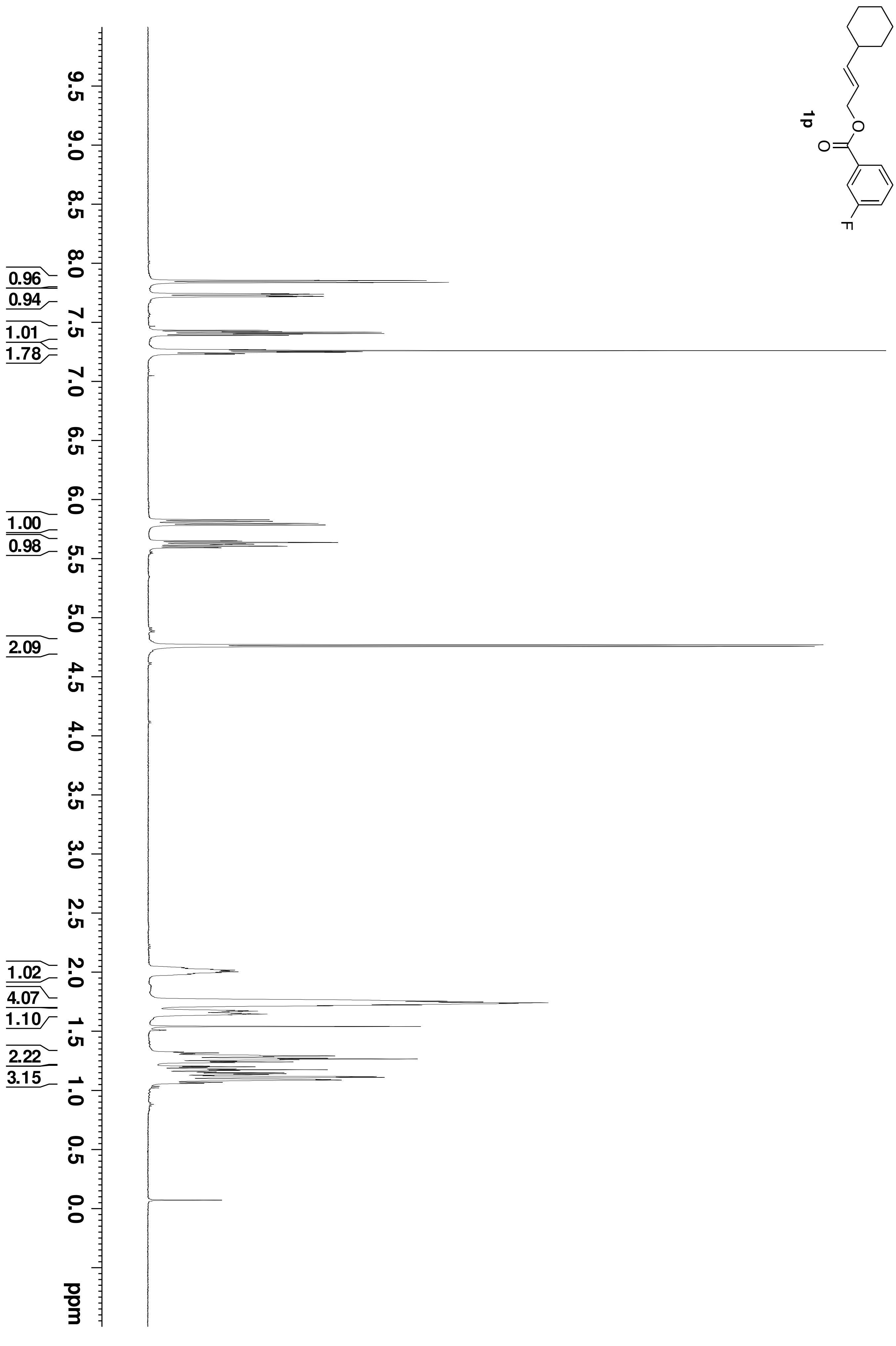
1p
O
O
F
9.5
9.0
8.5
8.0
7.5
7.0
6.5
6.0
5.5
5.0
4.5
4.0
3.5
3.0
2.5
2.0
1.5
1.0
0.5
0.0
ppm
0.96
0.94
1.01
1.78
1.00
0.98
2.09
1.02
4.07
1.10
2.22
3.15

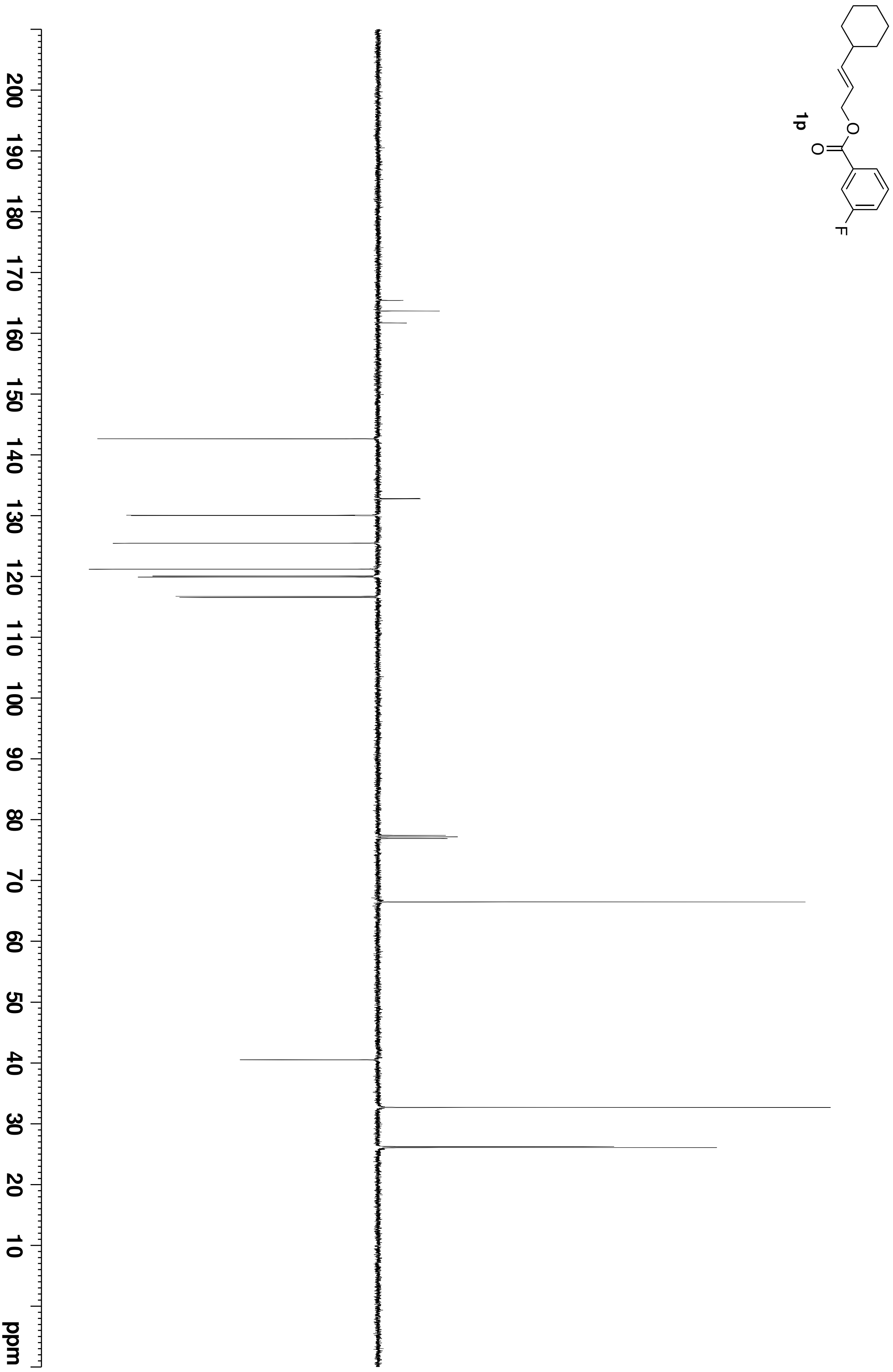
F
O
O
1p
ppm
200
190
180
170
160
150
140
130
120
110
100
90
80
70
60
50
40
30
20
10

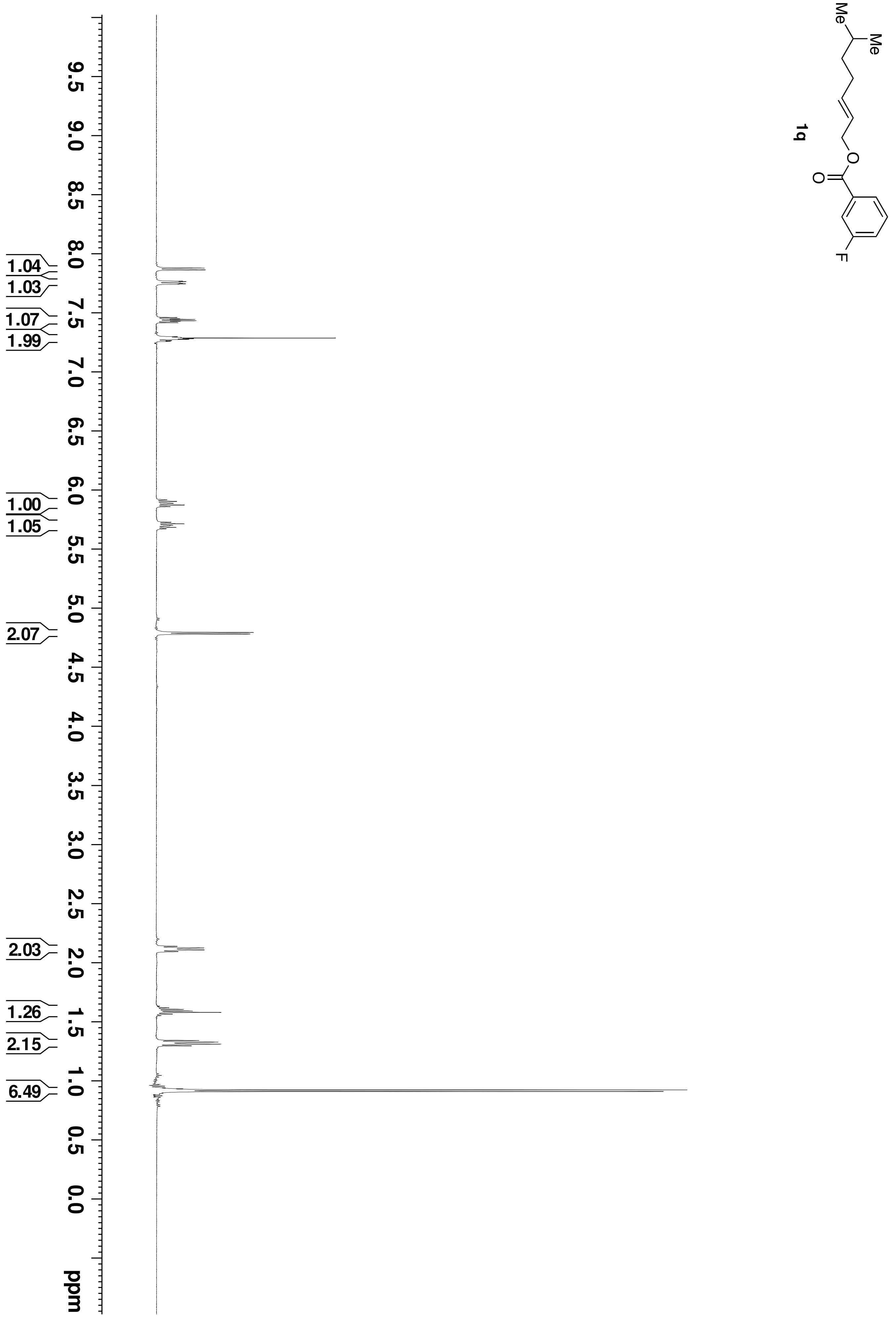
9.5
9.0
8.5
8.0
7.5
7.0
6.5
6.0
5.5
5.0
4.5
4.0
3.5
3.0
2.5
2.0
1.5
1.0
0.5
0.0
ppm
1.04
1.03
1.07
1.99
1.00
1.05
2.07
2.03
1.26
2.15
6.49
Me
Me
O
O
F

1q

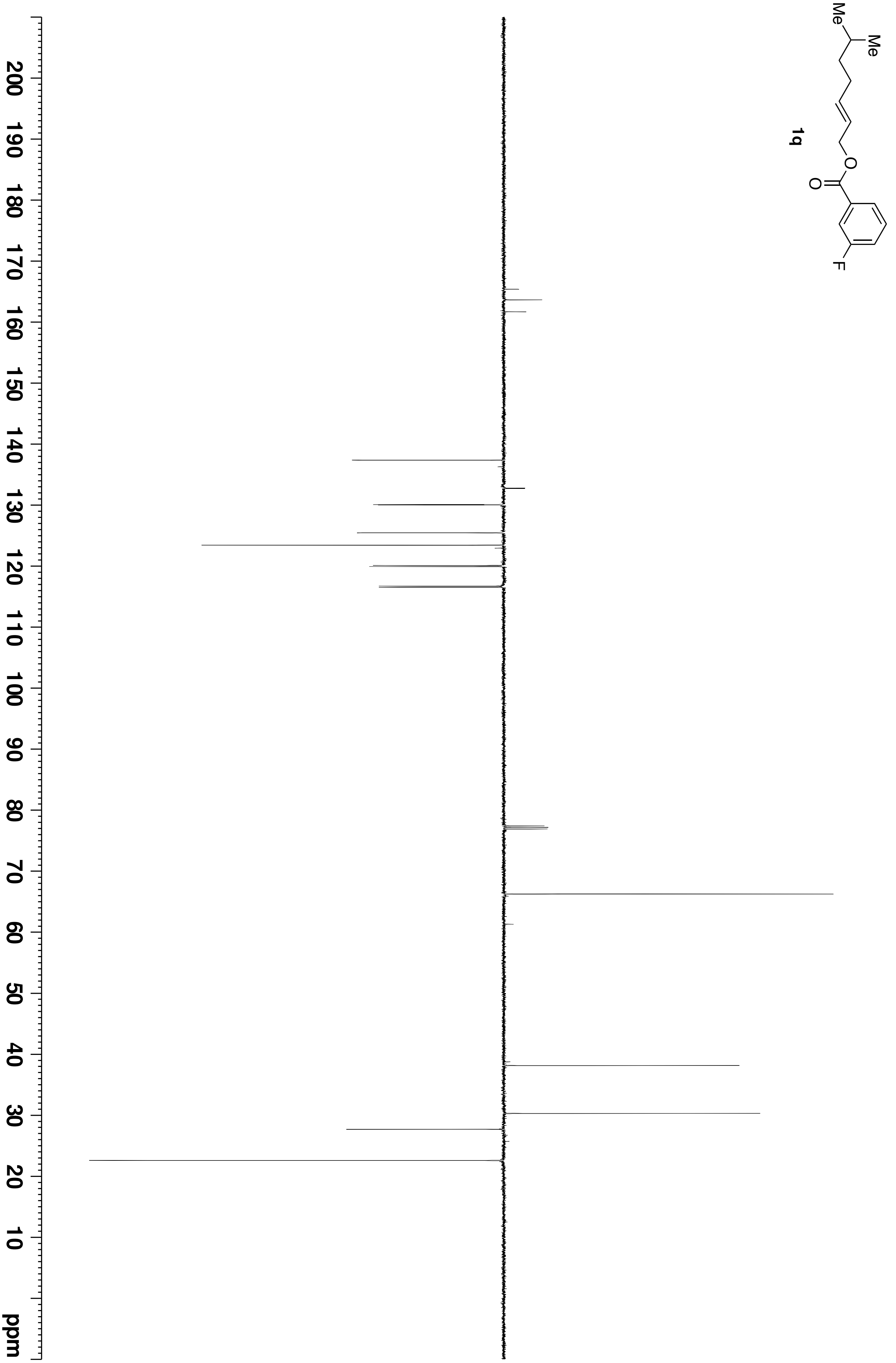
Me
Me
1q
O
O
F
200
190
180
170
160
150
140
130
120
110
100
90
80
70
60
50
40
30
20
10
ppm

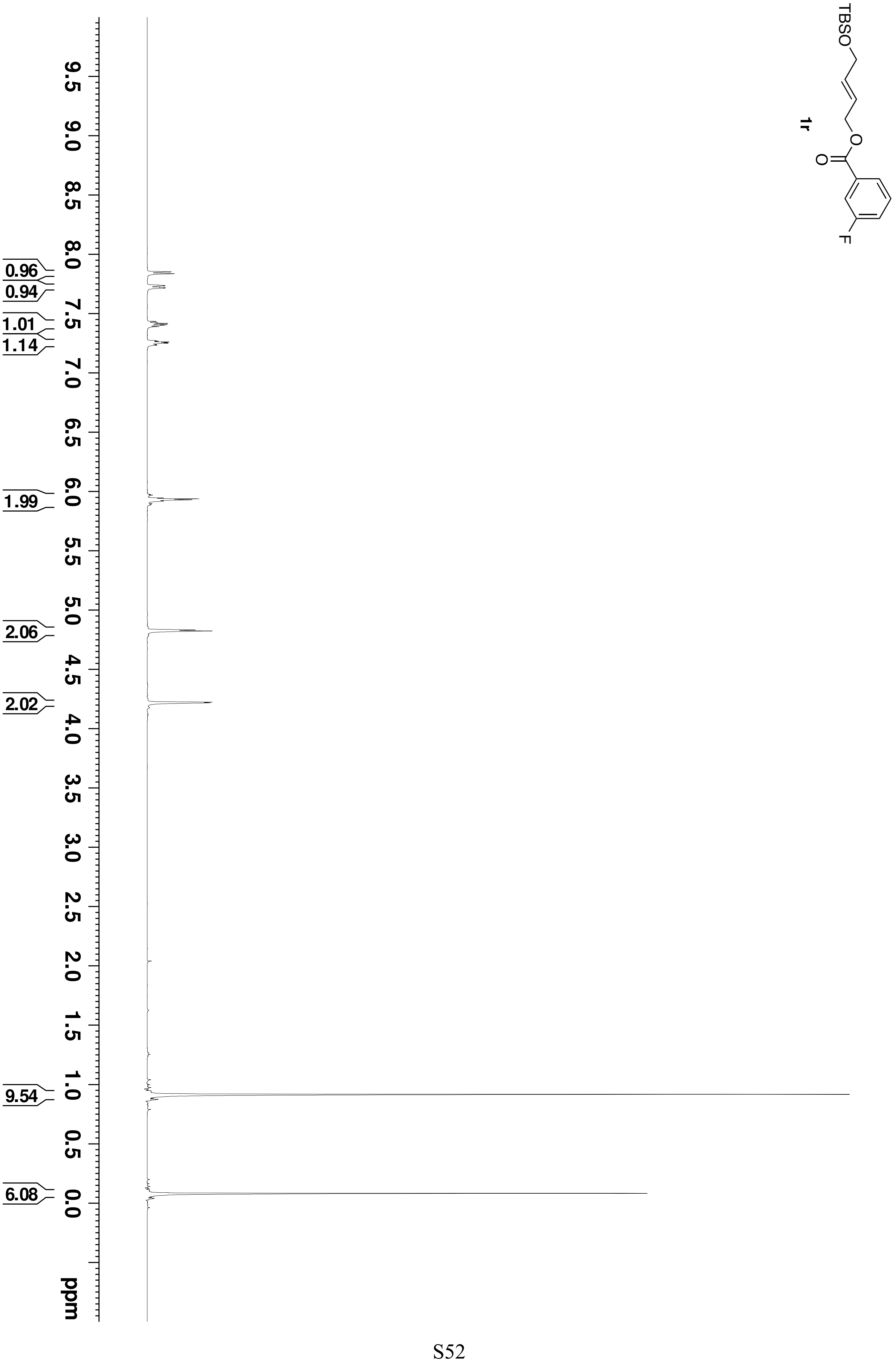
TBSO
O
O
F
1r
0.96
0.94
1.01
1.14
1.99
2.06
2.02
9.54
6.08
9.5
9.0
8.5
8.0
7.5
7.0
6.5
6.0
5.5
5.0
4.5
4.0
3.5
3.0
2.5
2.0
1.5
1.0
0.5
0.0
ppm

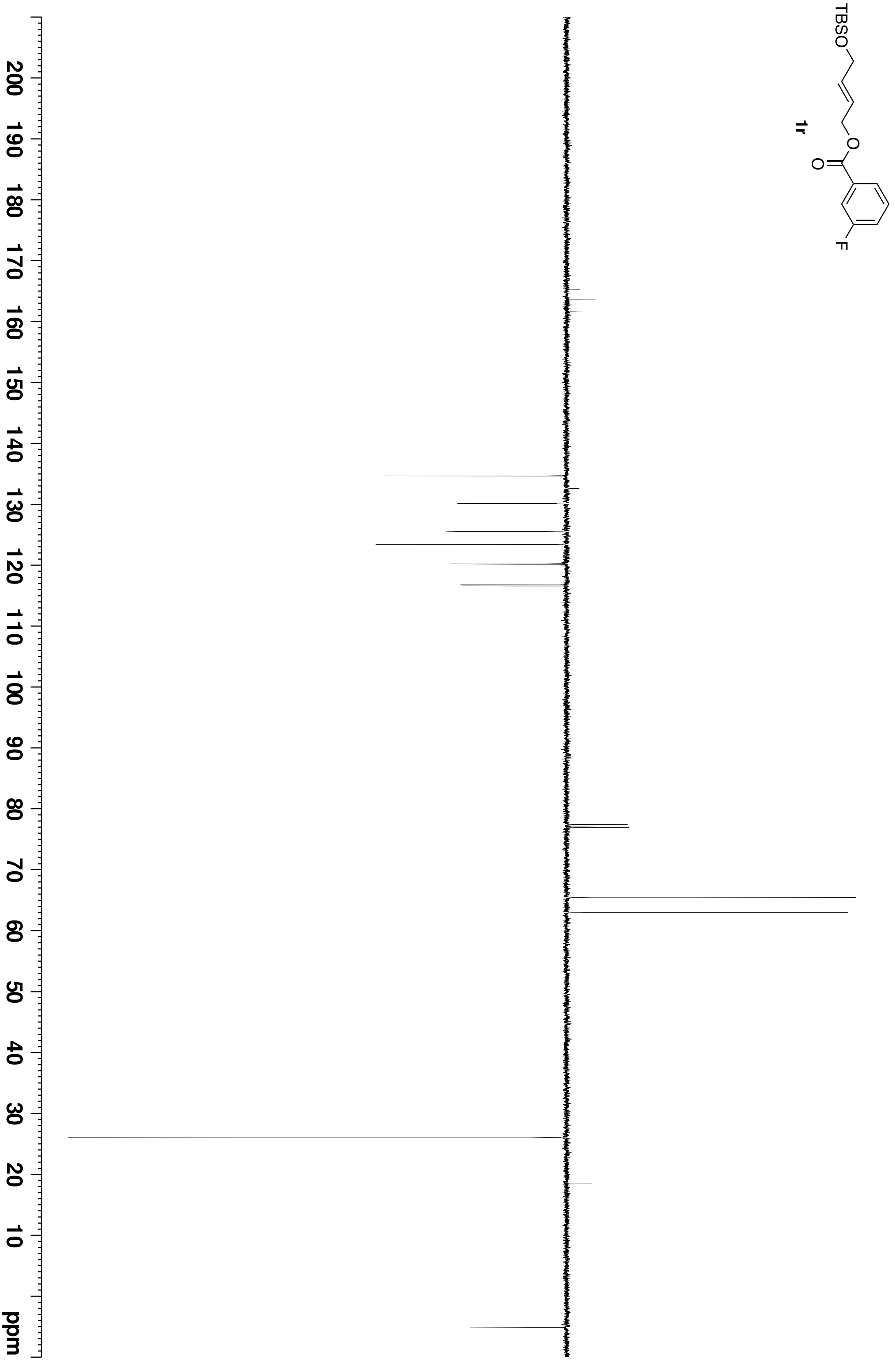

TBSO
O
F
1r
200
190
180
170
160
150
140
130
120
110
100
90
80
70
60
50
40
30
20
10
ppm

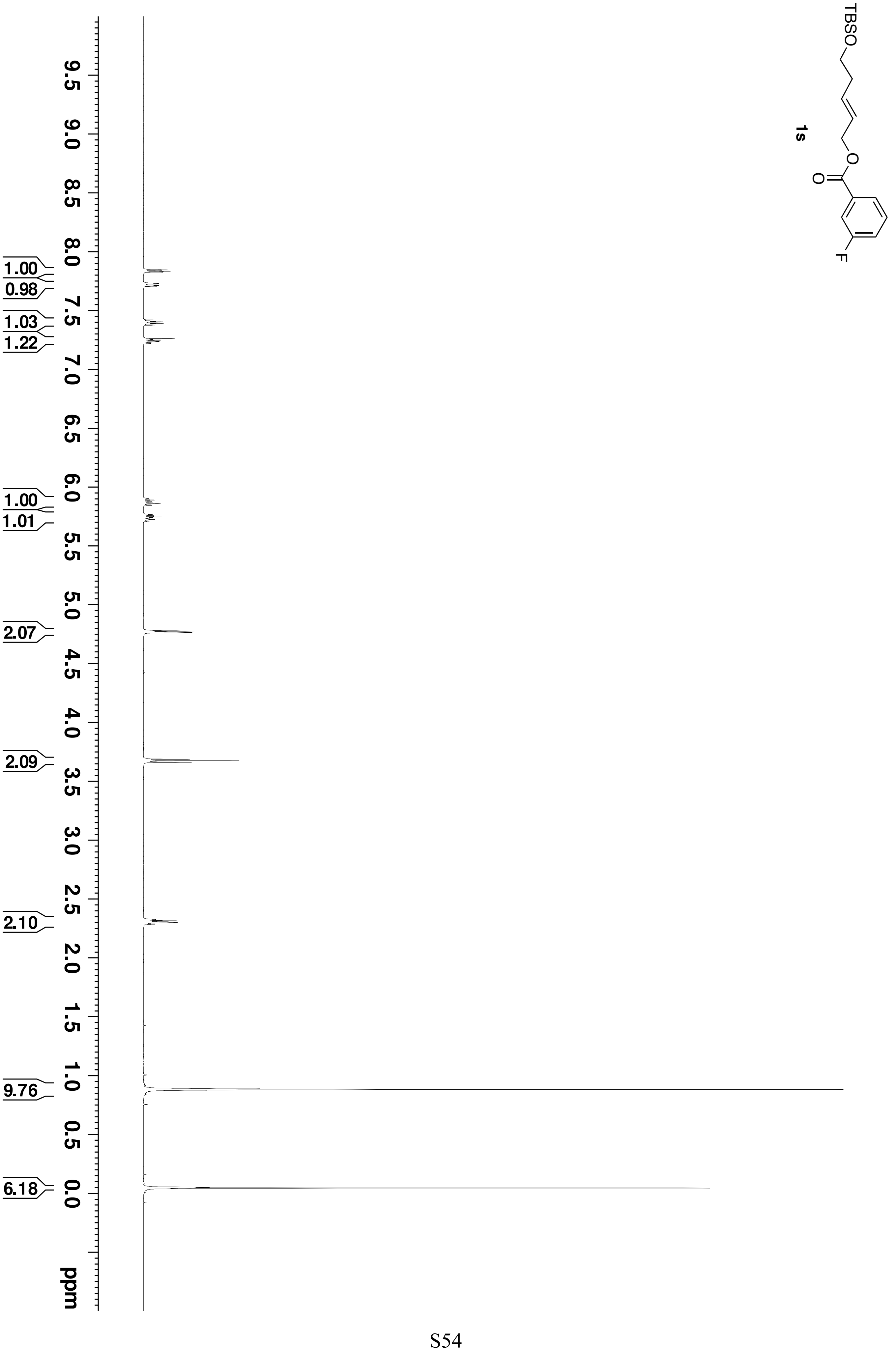
TBSO
O
F
1s
1.00
0.98
1.03
1.22
1.00
1.01
2.07
2.09
2.10
9.76
6.18
9.5
9.0
8.5
8.0
7.5
7.0
6.5
6.0
5.5
5.0
4.5
4.0
3.5
3.0
2.5
2.0
1.5
1.0
0.5
0.0
ppm

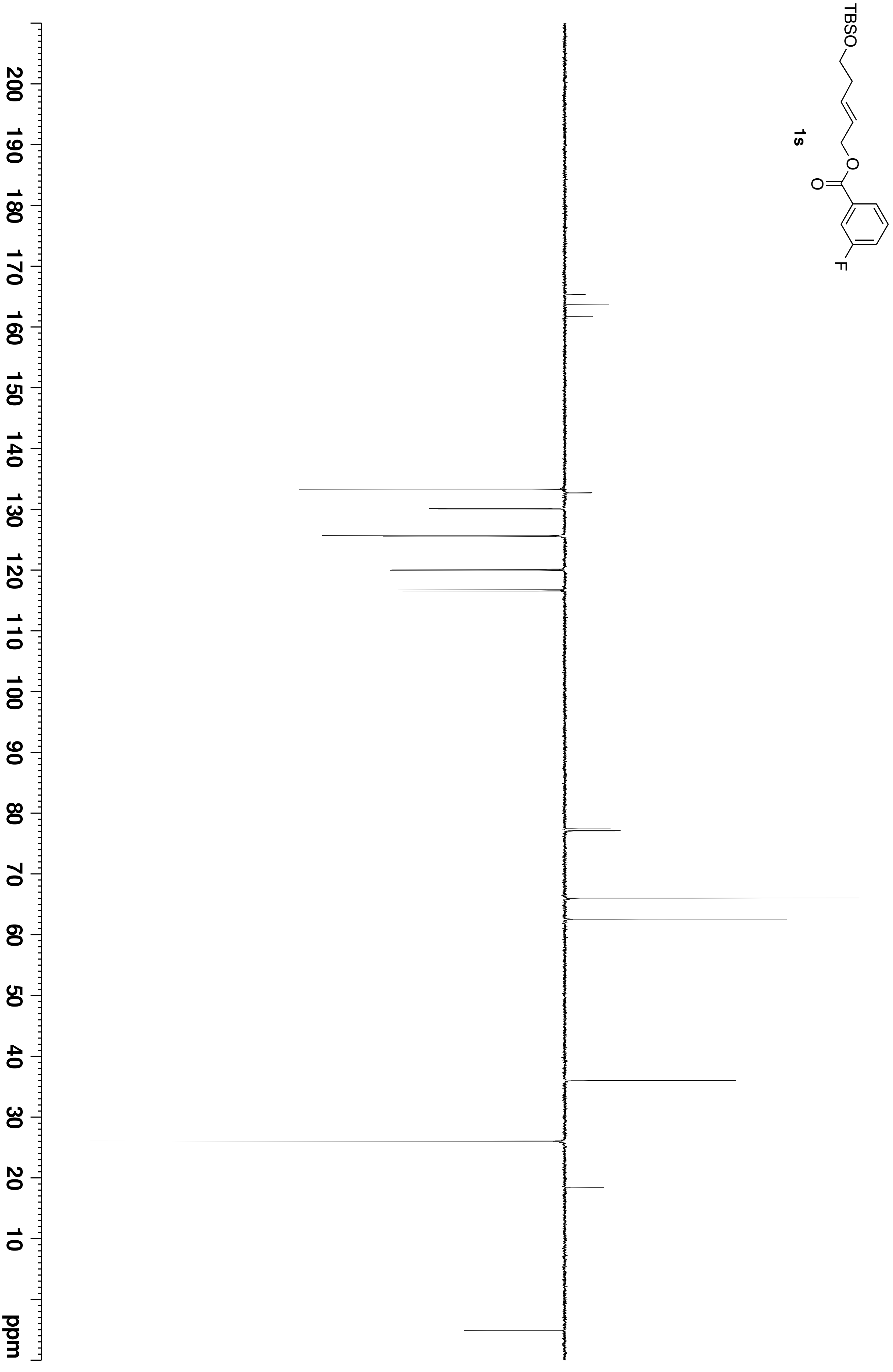
TBSO
O
O
F
1s
200
190
180
170
160
150
140
130
120
110
100
90
80
70
60
50
40
30
20
10
ppm

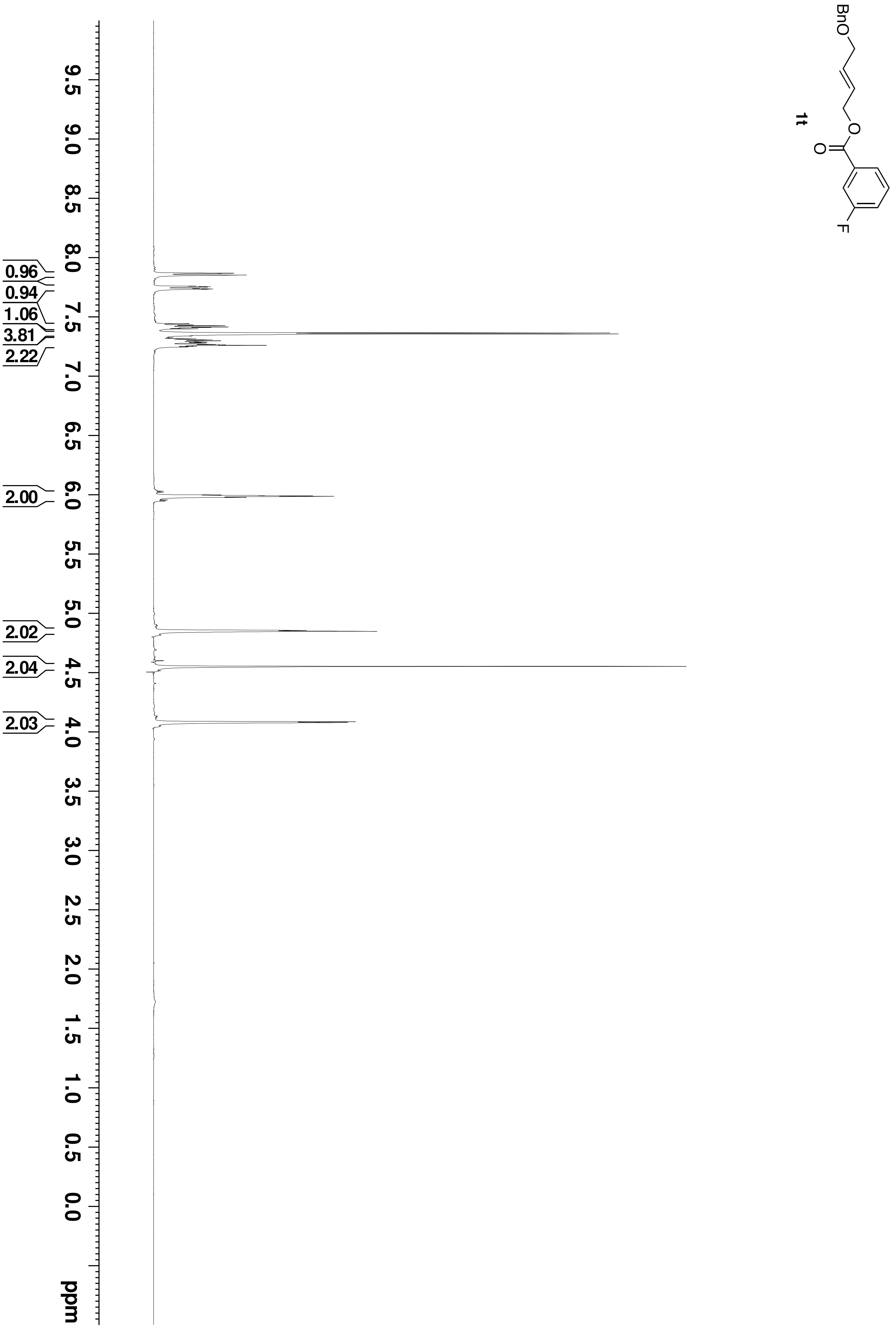
BnO
O
F
1t
0.96
0.94
1.06
3.81
2.22
2.00
2.02
2.04
2.03
ppm

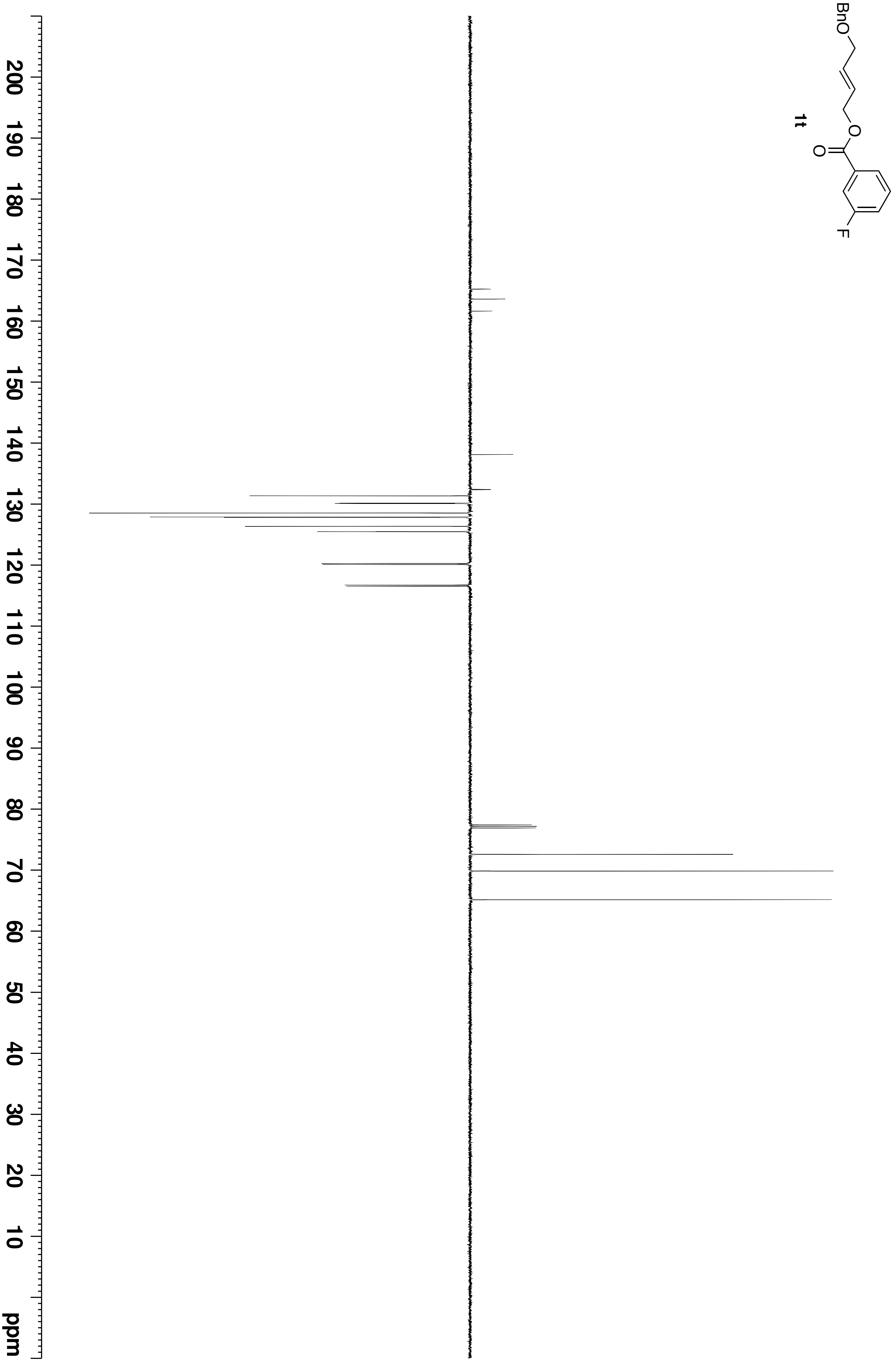

BnO
O
O
F
1t
200
190
180
170
160
150
140
130
120
110
100
90
80
70
60
50
40
30
20
10
ppm

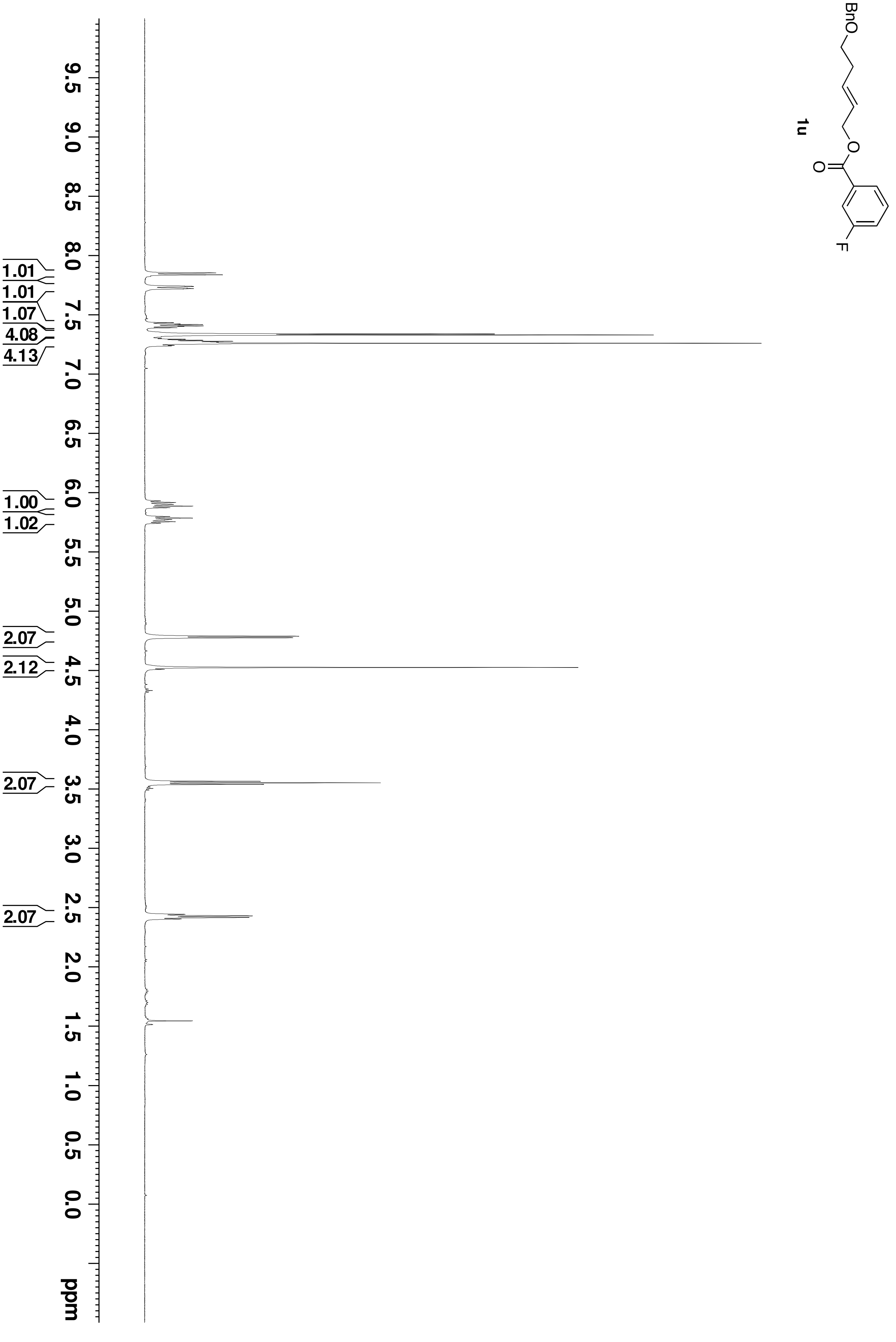
BnO
O
F
1u
1.01
1.01
1.07
4.08
4.13
1.00
1.02
2.07
2.12
2.07
2.07
9.5
9.0
8.5
8.0
7.5
7.0
6.5
6.0
5.5
5.0
4.5
4.0
3.5
3.0
2.5
2.0
1.5
1.0
0.5
0.0
ppm

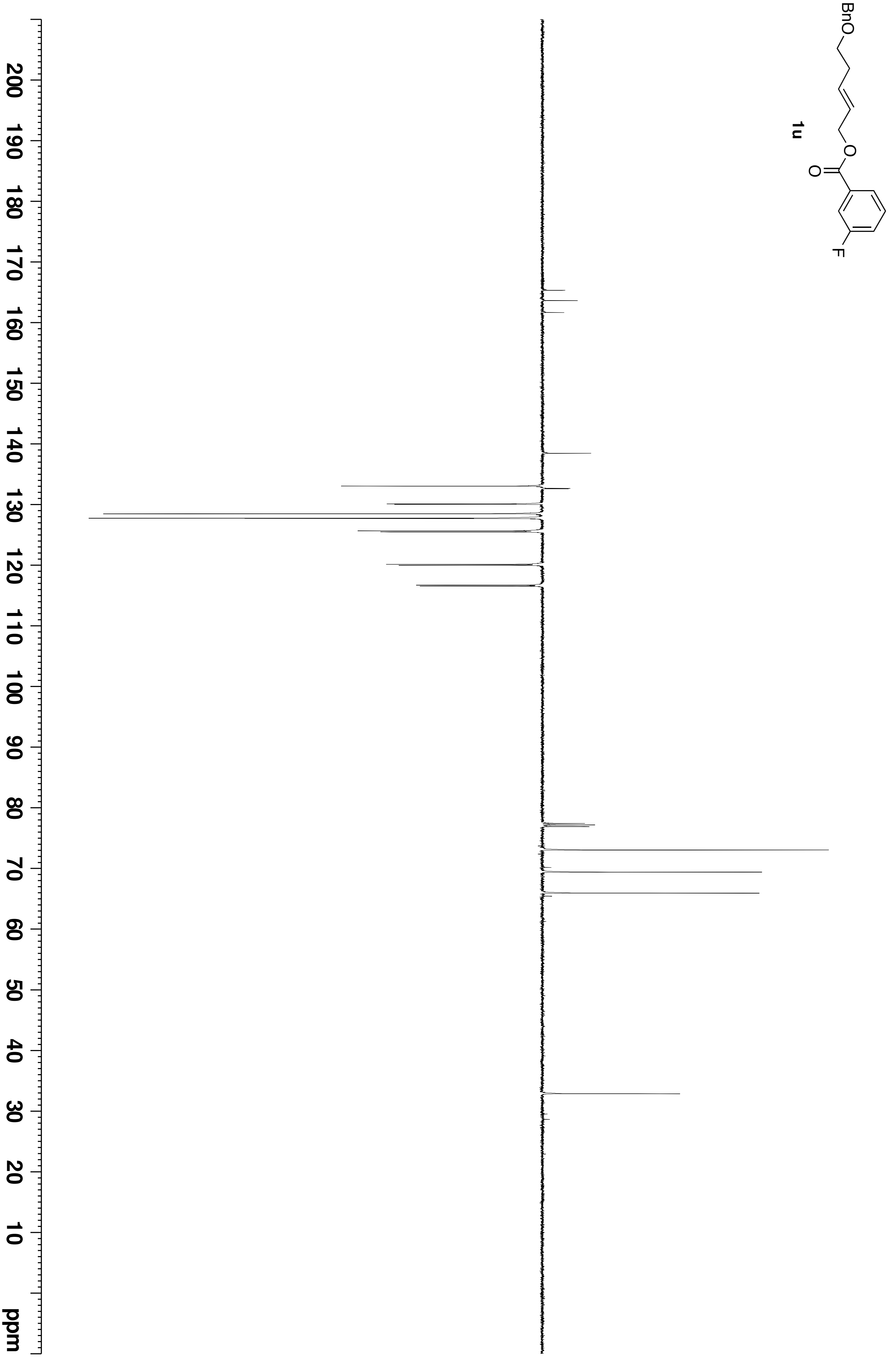
BnO
O
F
1u
200
190
180
170
160
150
140
130
120
110
100
90
80
70
60
50
40
30
20
10
ppm

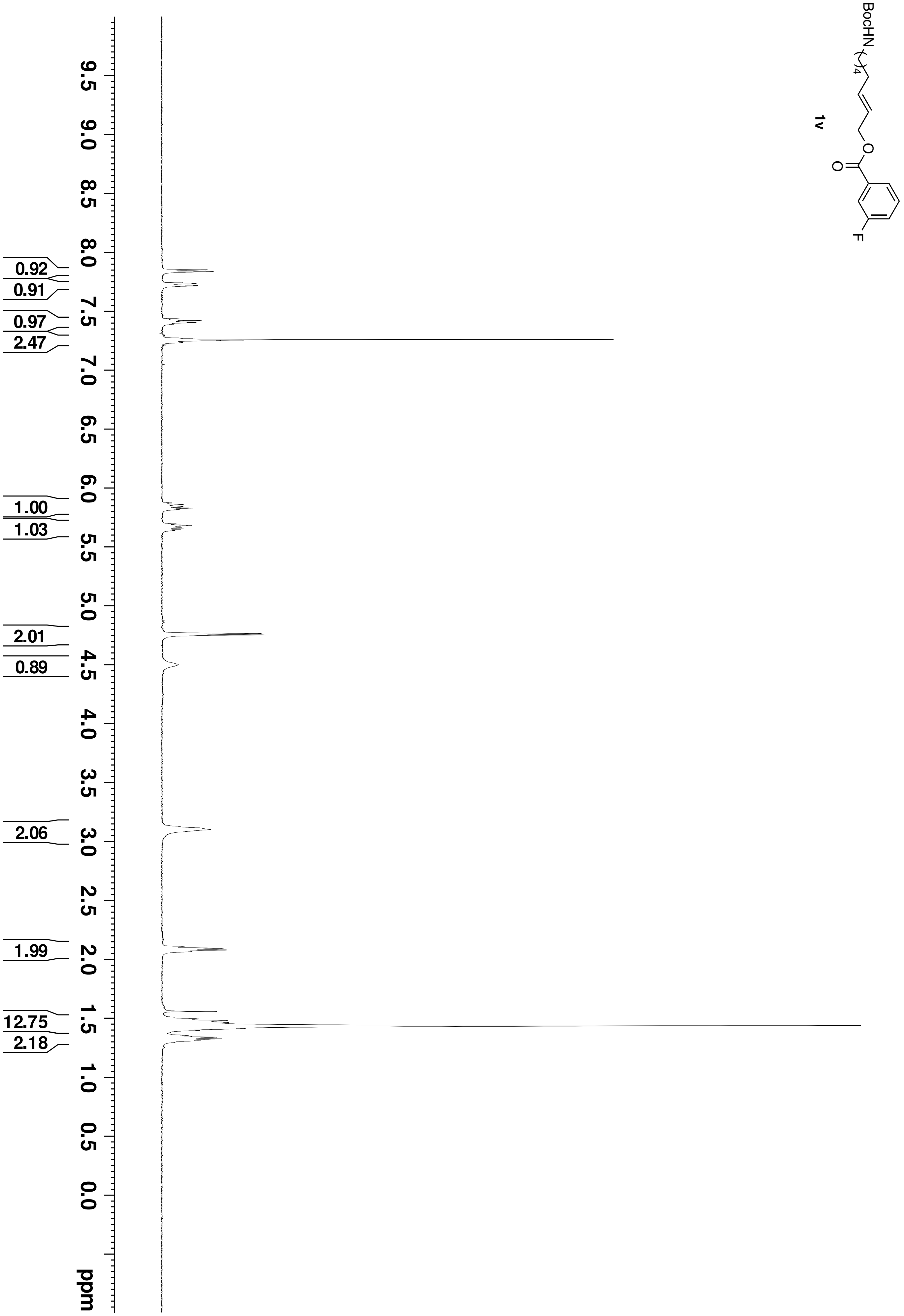
BocHN
4
O
O
F
1v
9.5 9.0 8.5 8.0 7.5 7.0 6.5 6.0 5.5 5.0 4.5 4.0 3.5 3.0 2.5 2.0 1.5 1.0 0.5 0.0 ppm
0.92
0.91
0.97
2.47
1.00
1.03
2.01
0.89
2.06
1.99
12.75
2.18

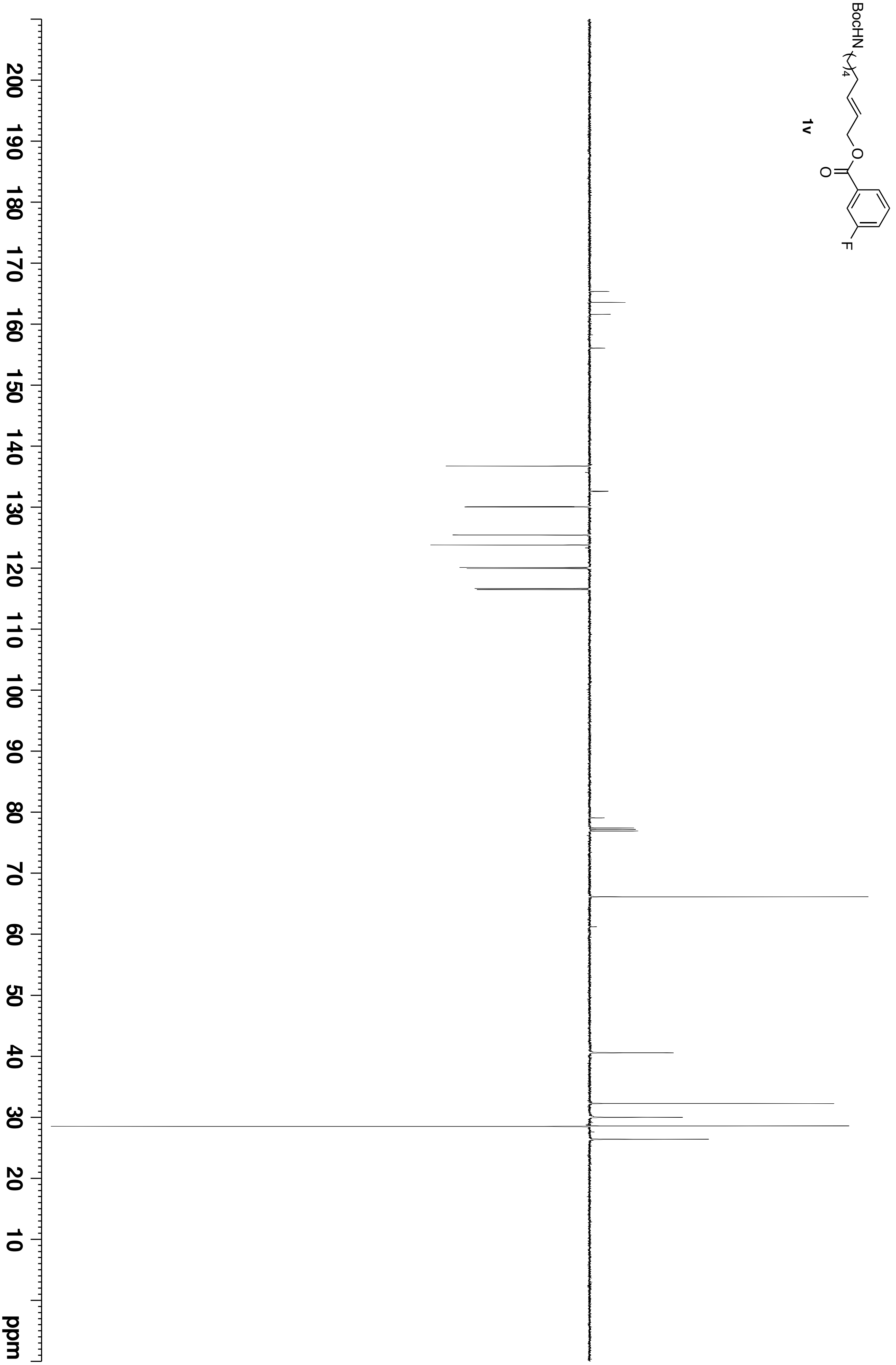
BocHN
4
O
O
F
1v
200
190
180
170
160
150
140
130
120
110
100
90
80
70
60
50
40
30
20
10
ppm

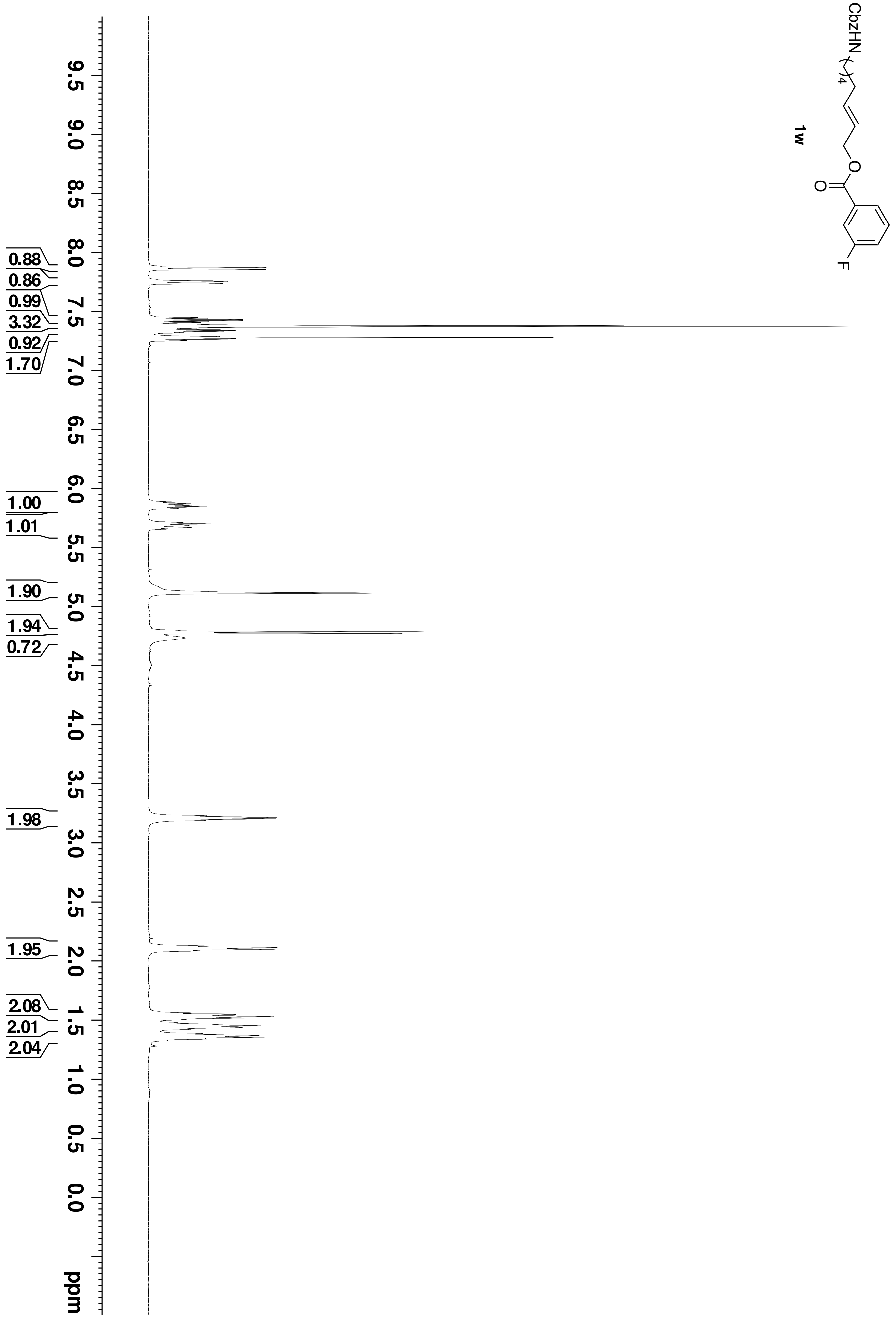
CbzHN
4
O
O
F
1w
9.5 9.0 8.5 8.0 7.5 7.0 6.5 6.0 5.5 5.0 4.5 4.0 3.5 3.0 2.5 2.0 1.5 1.0 0.5 0.0 ppm
0.88 0.86 0.99 3.32 0.92 1.70
1.00 1.01
1.90 1.94 0.72
1.98
1.95
2.08 2.01 2.04

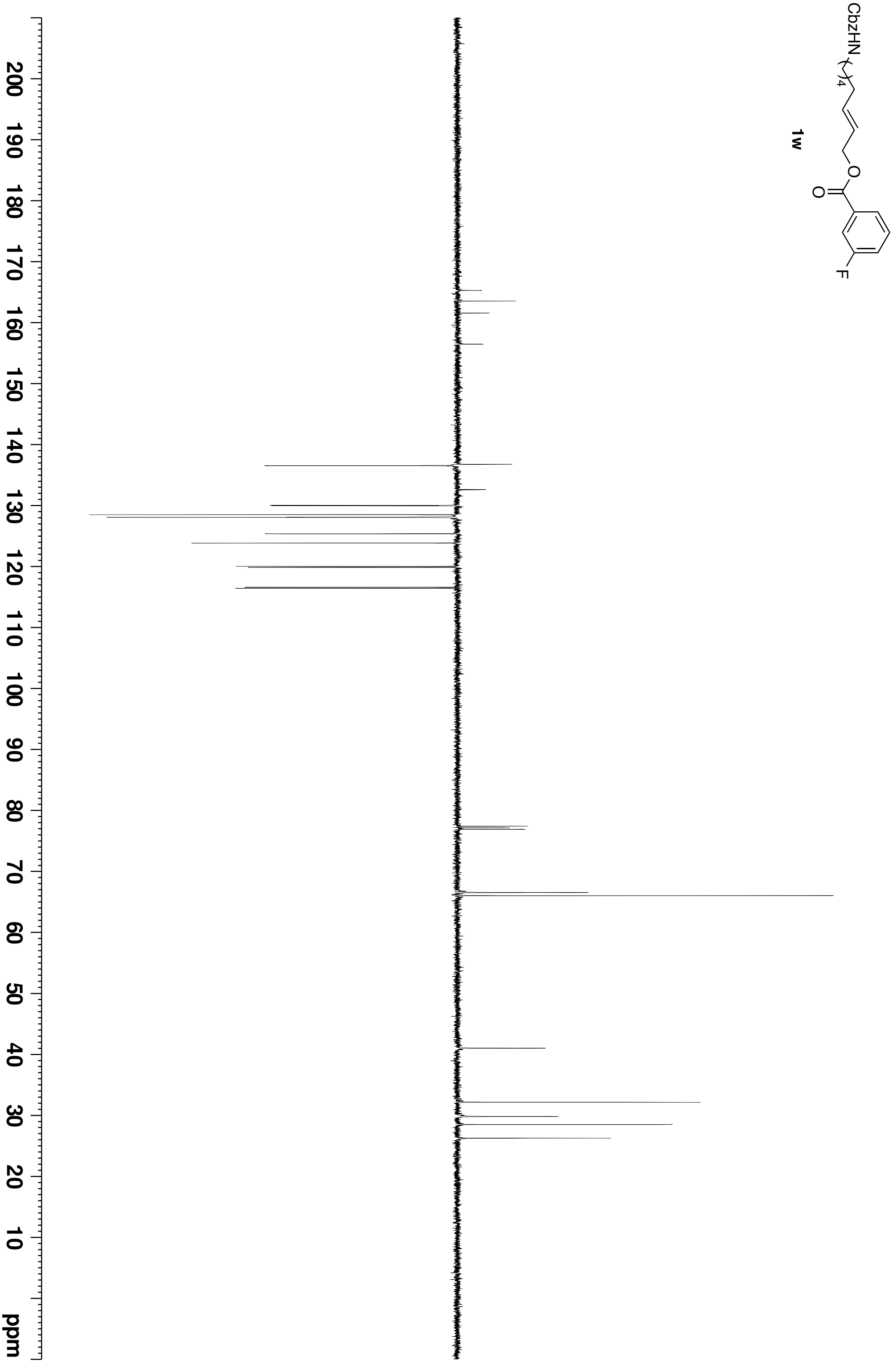
CbzHN
4
O
O
F
1w
200
190
180
170
160
150
140
130
120
110
100
90
80
70
60
50
40
30
20
10
ppm

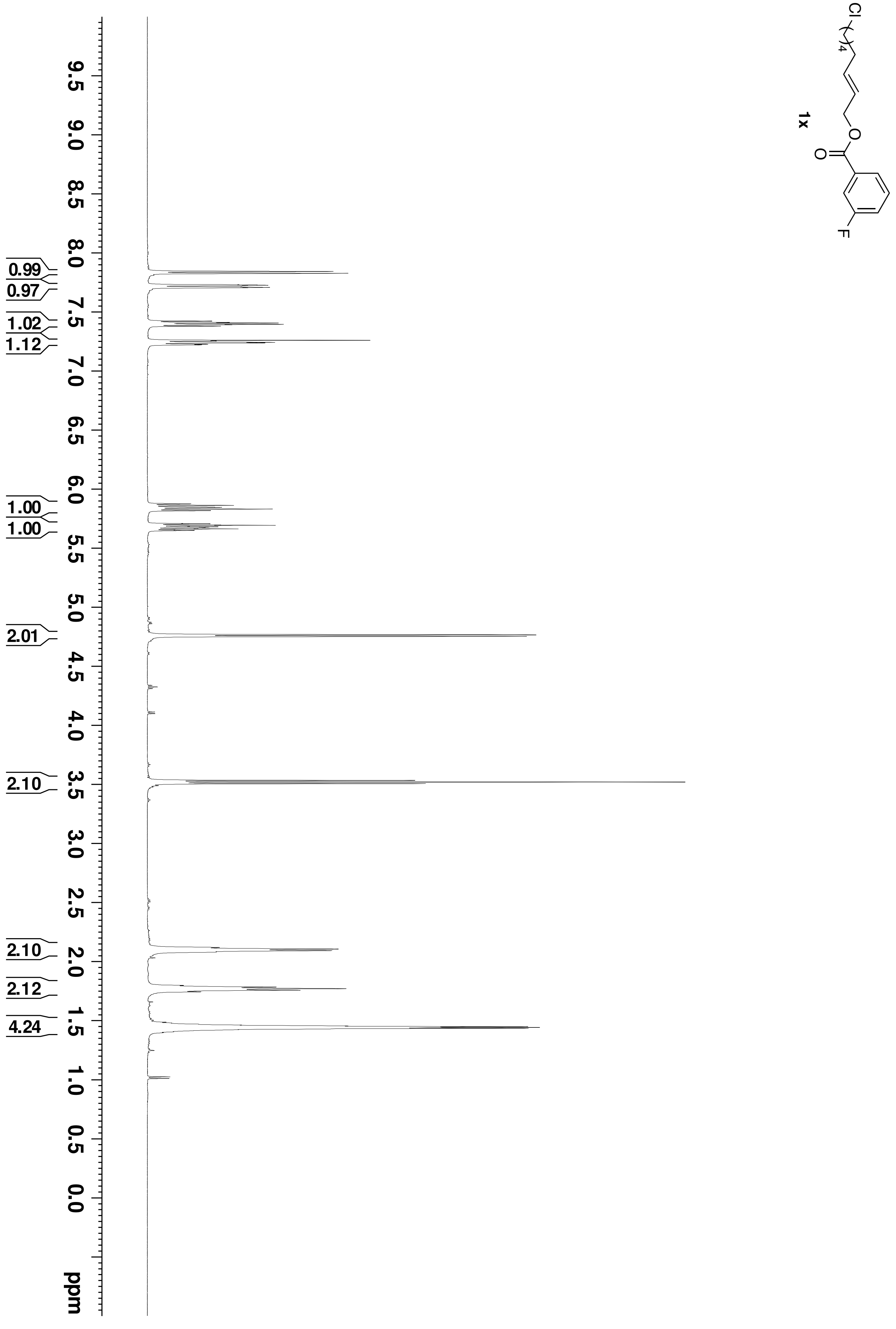
1x
ppm

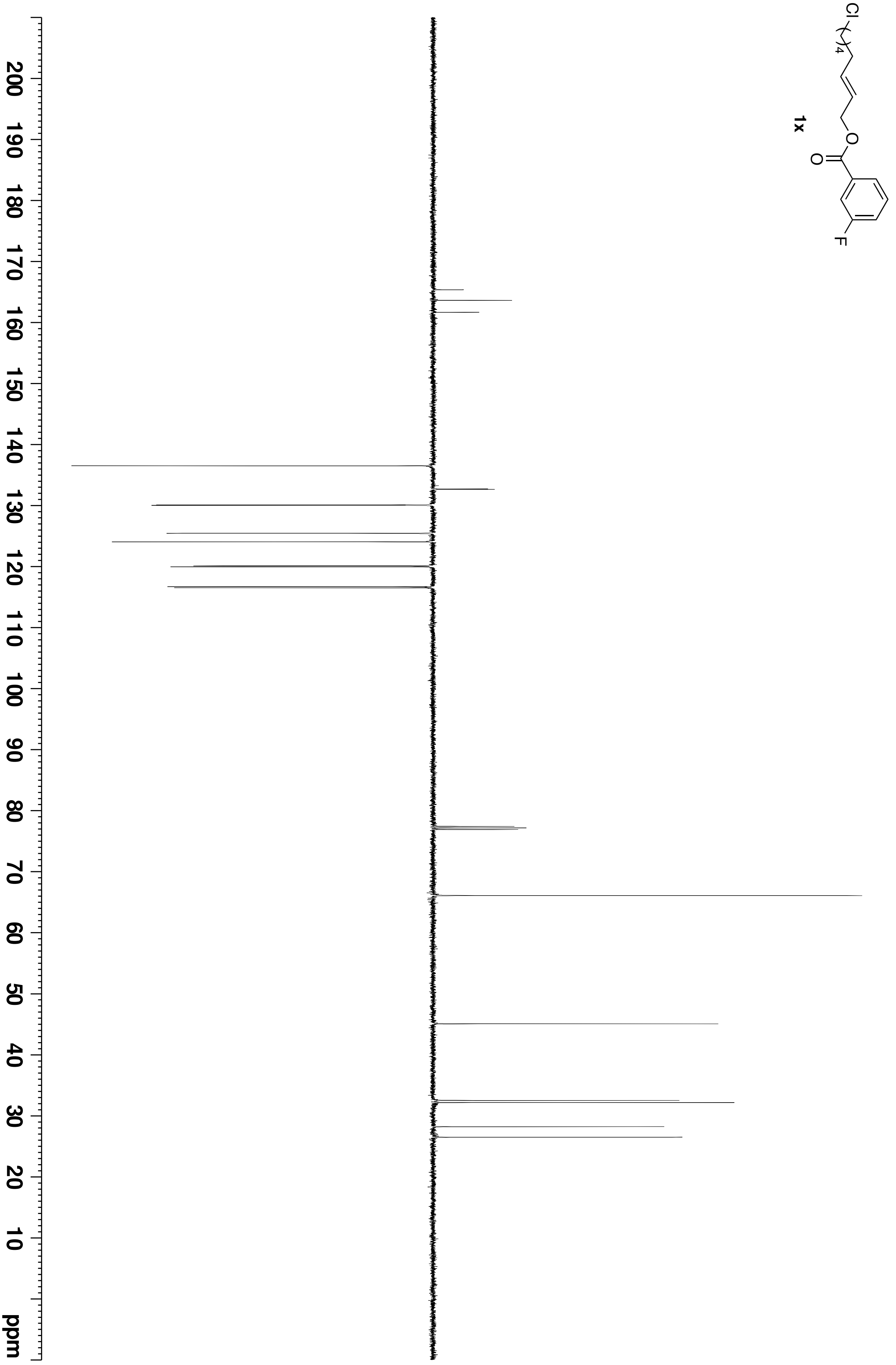

1x
Cl
O
O
F
4
200 190 180 170 160 150 140 130 120 110 100 90 80 70 60 50 40 30 20 10
ppm

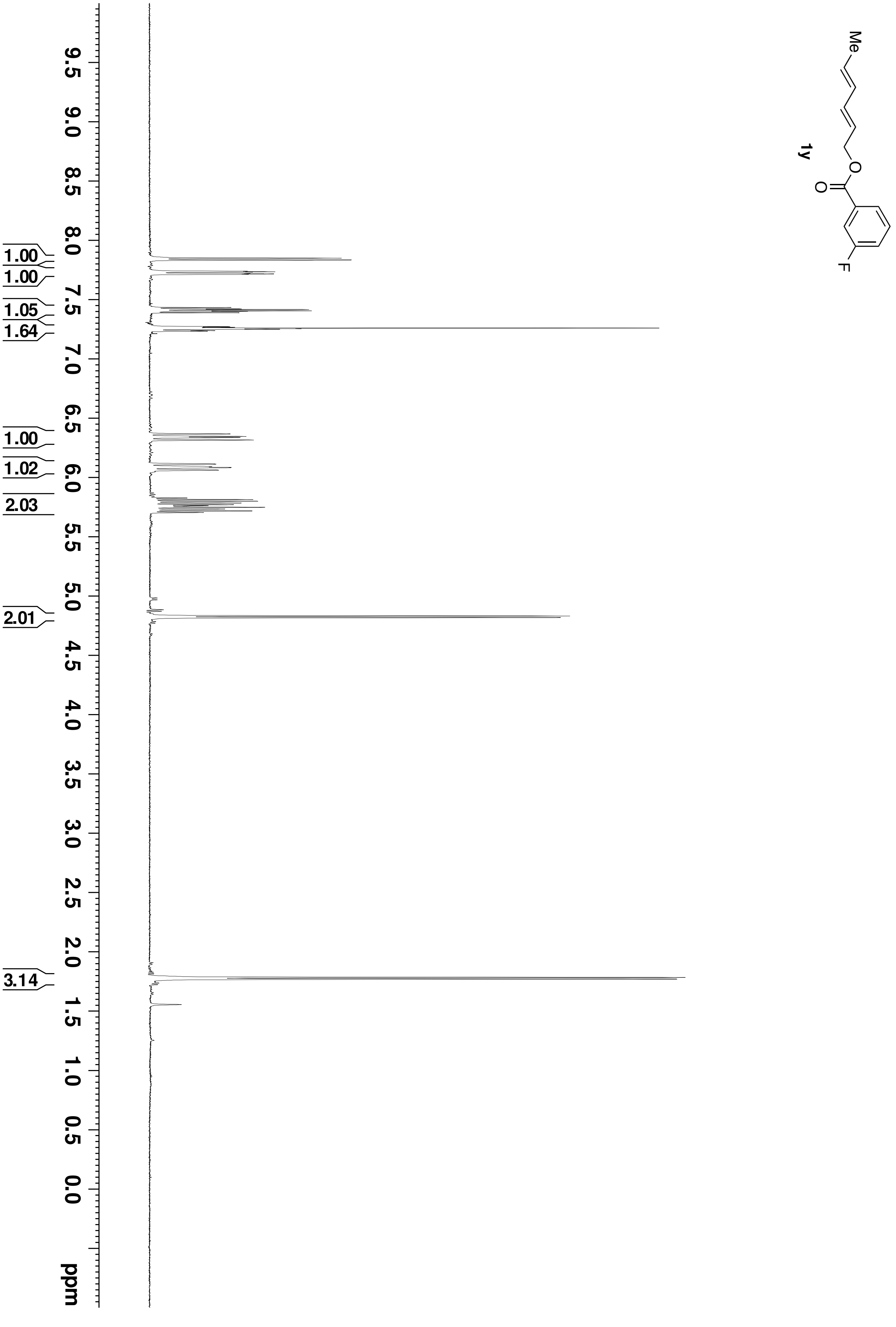

Me
O
O
F
1y
9.5
9.0
8.5
8.0
7.5
7.0
6.5
6.0
5.5
5.0
4.5
4.0
3.5
3.0
2.5
2.0
1.5
1.0
0.5
0.0
ppm
1.00
1.00
1.05
1.64
1.00
1.02
2.03
2.01
3.14

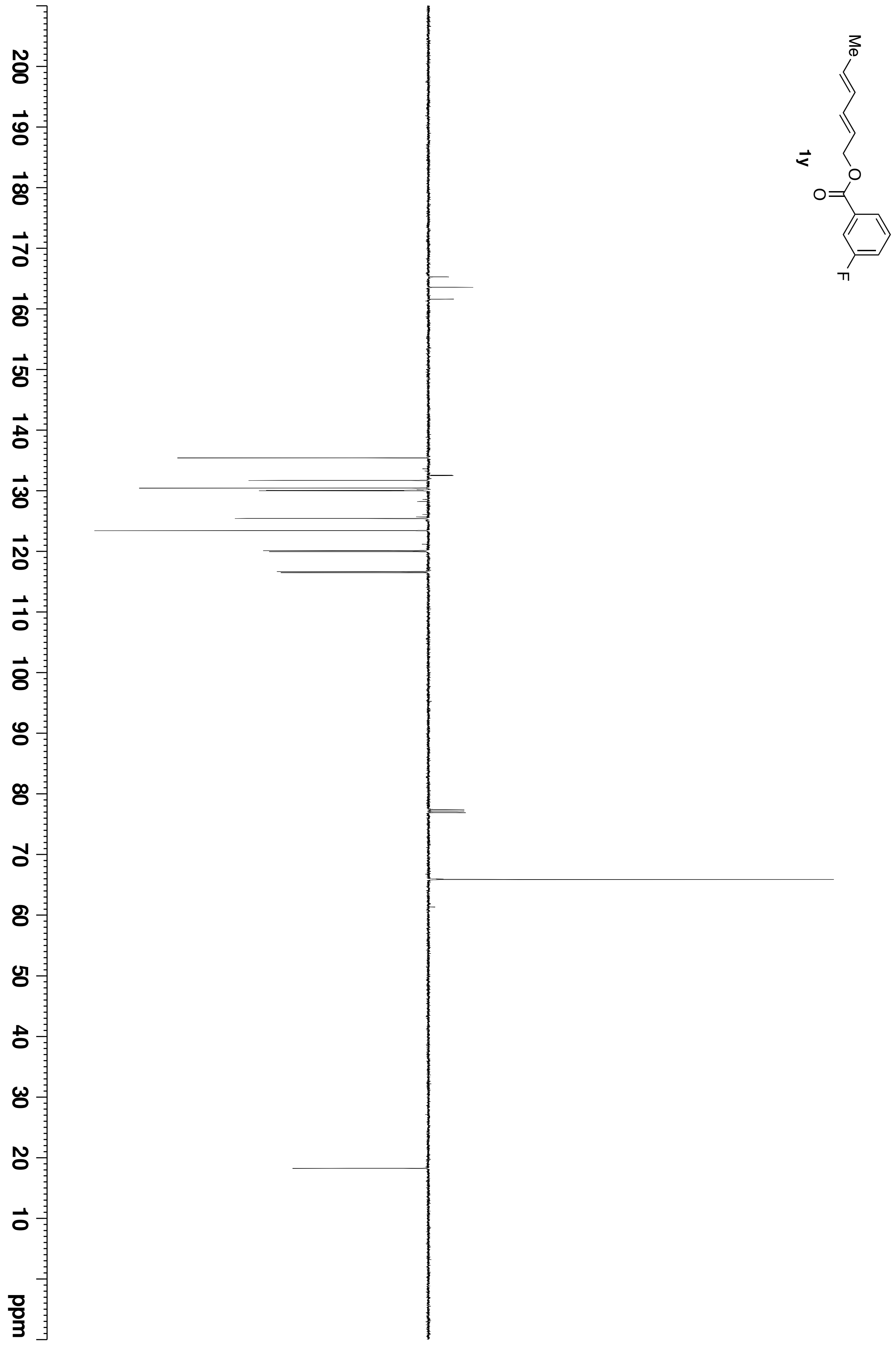

1y
ppm

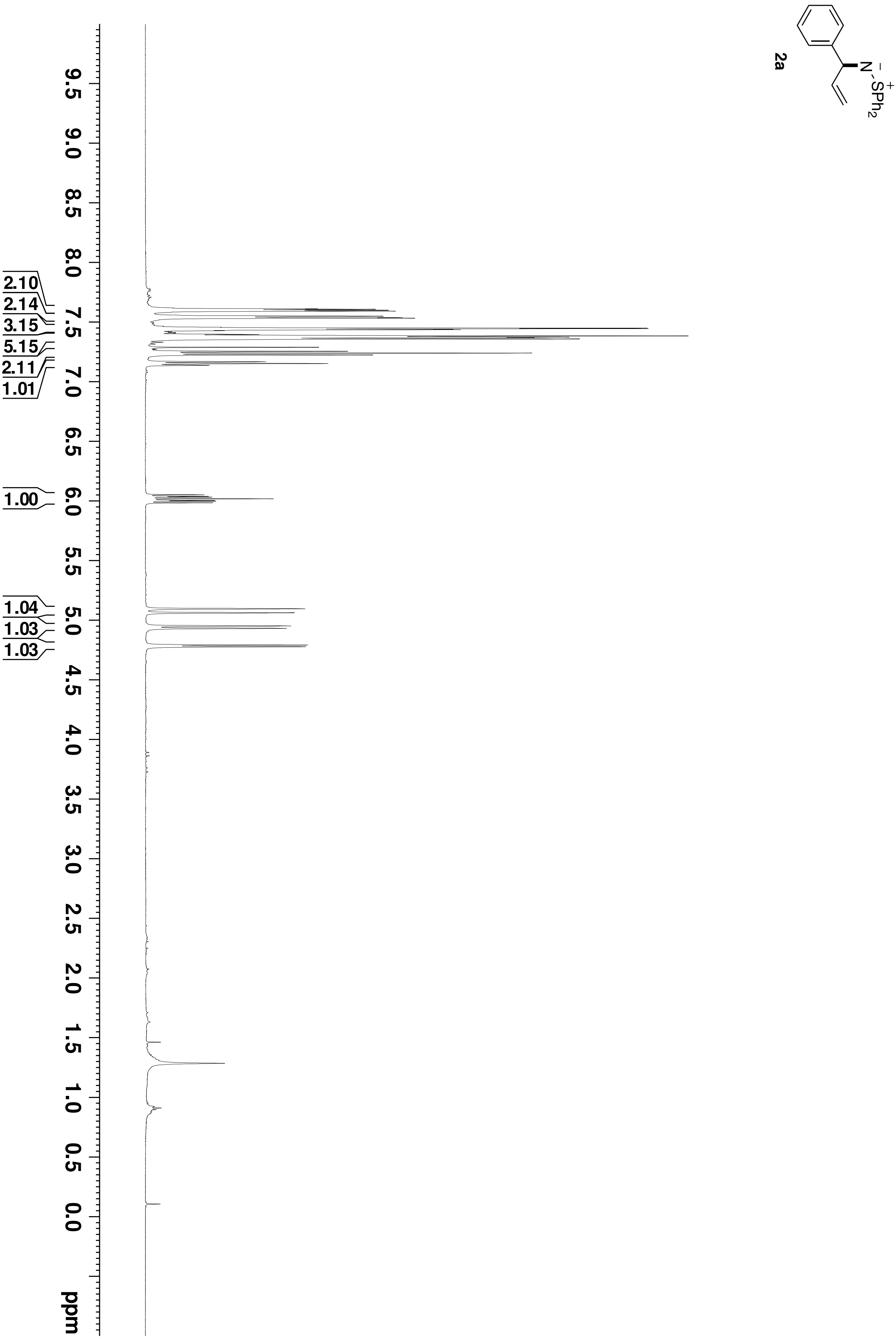
2a
N
SPh2
2.10
2.14
3.15
5.15
2.11
1.01
1.00
1.04
1.03
1.03
9.5
9.0
8.5
8.0
7.5
7.0
6.5
6.0
5.5
5.0
4.5
4.0
3.5
3.0
2.5
2.0
1.5
1.0
0.5
0.0
ppm

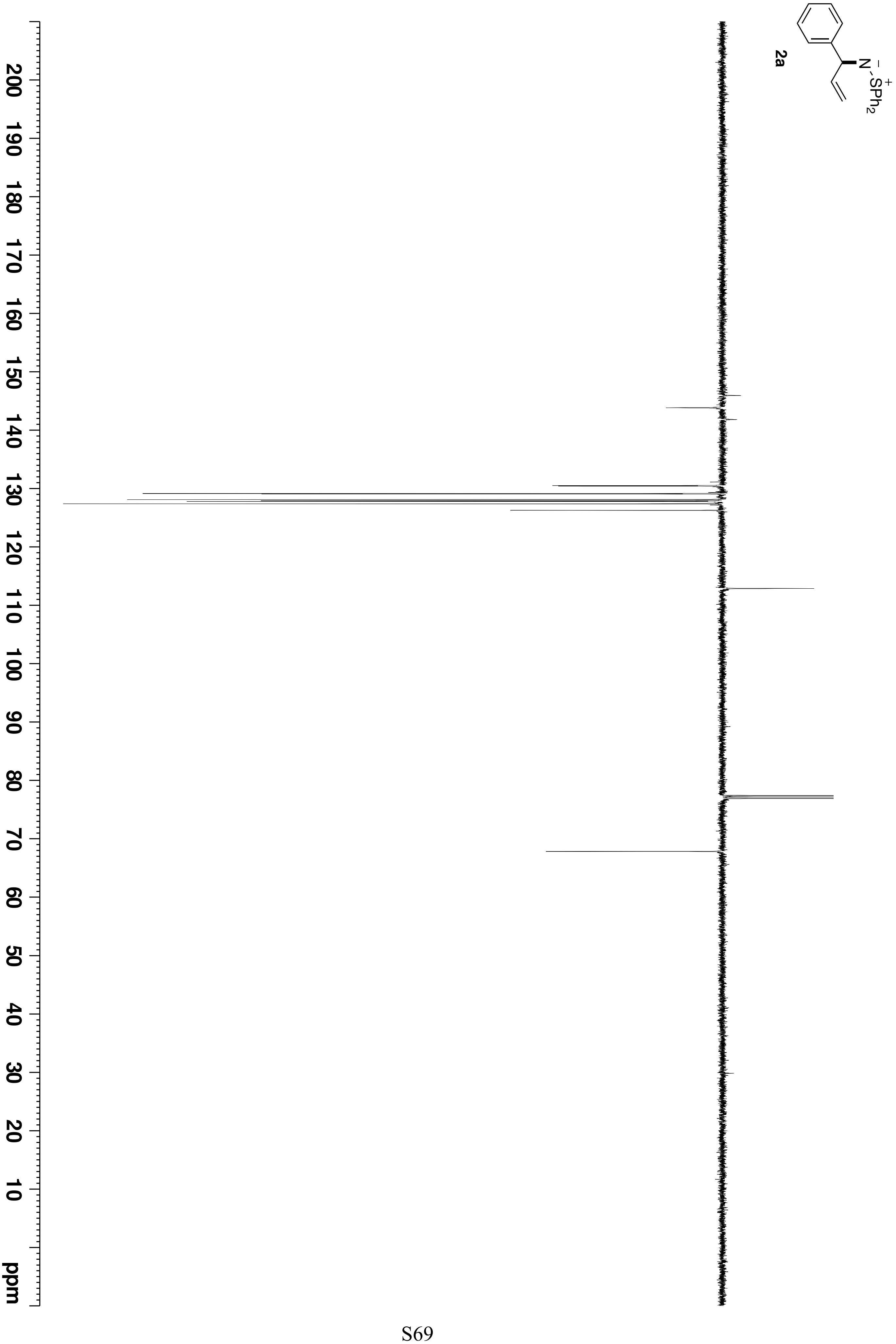
2a
N
SPh2
200
190
180
170
160
150
140
130
120
110
100
90
80
70
60
50
40
30
20
10
ppm

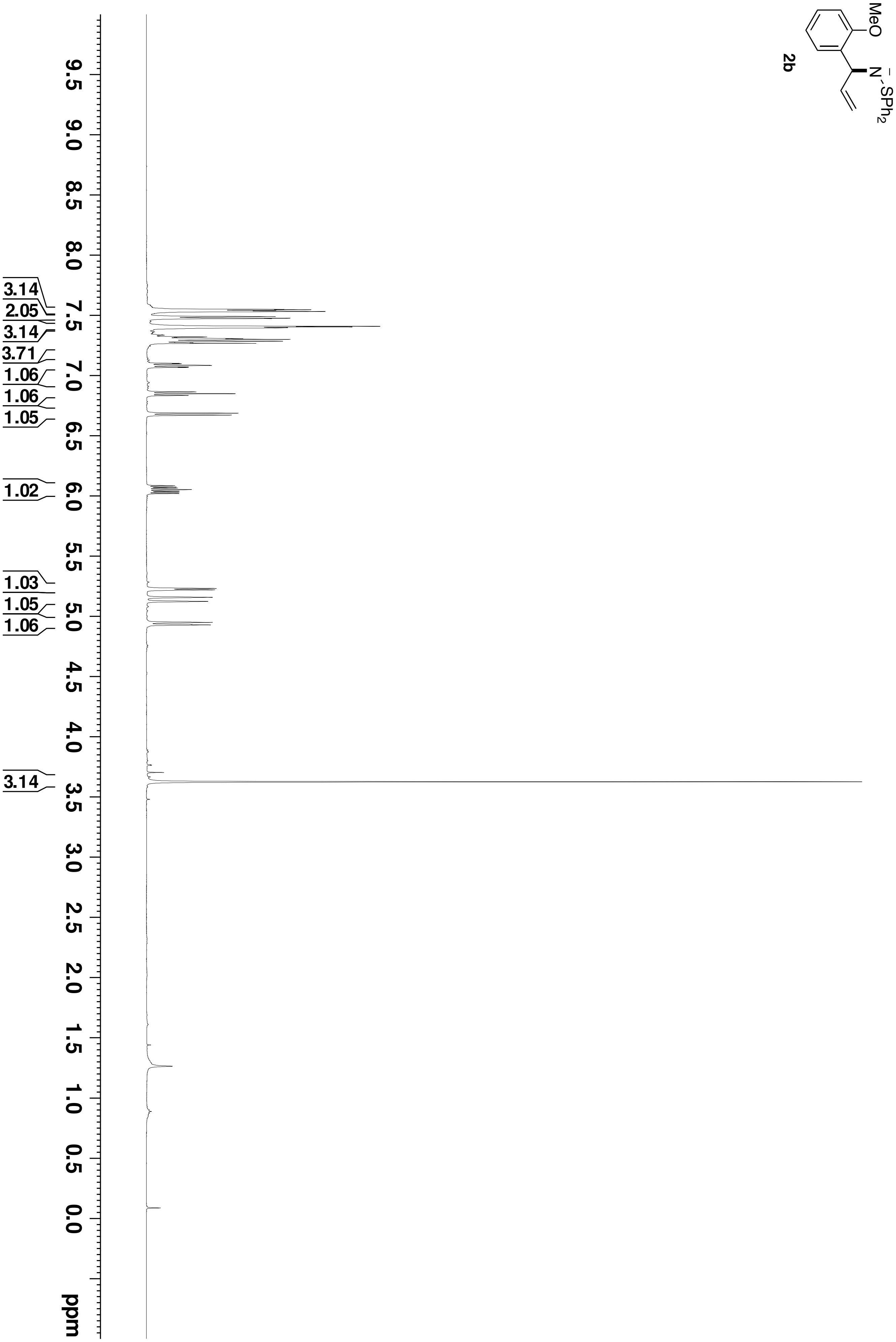
MeO
2b
ppm
3.14
2.05
3.14
3.71
1.06
1.06
1.05
1.02
1.03
1.05
1.06
3.14

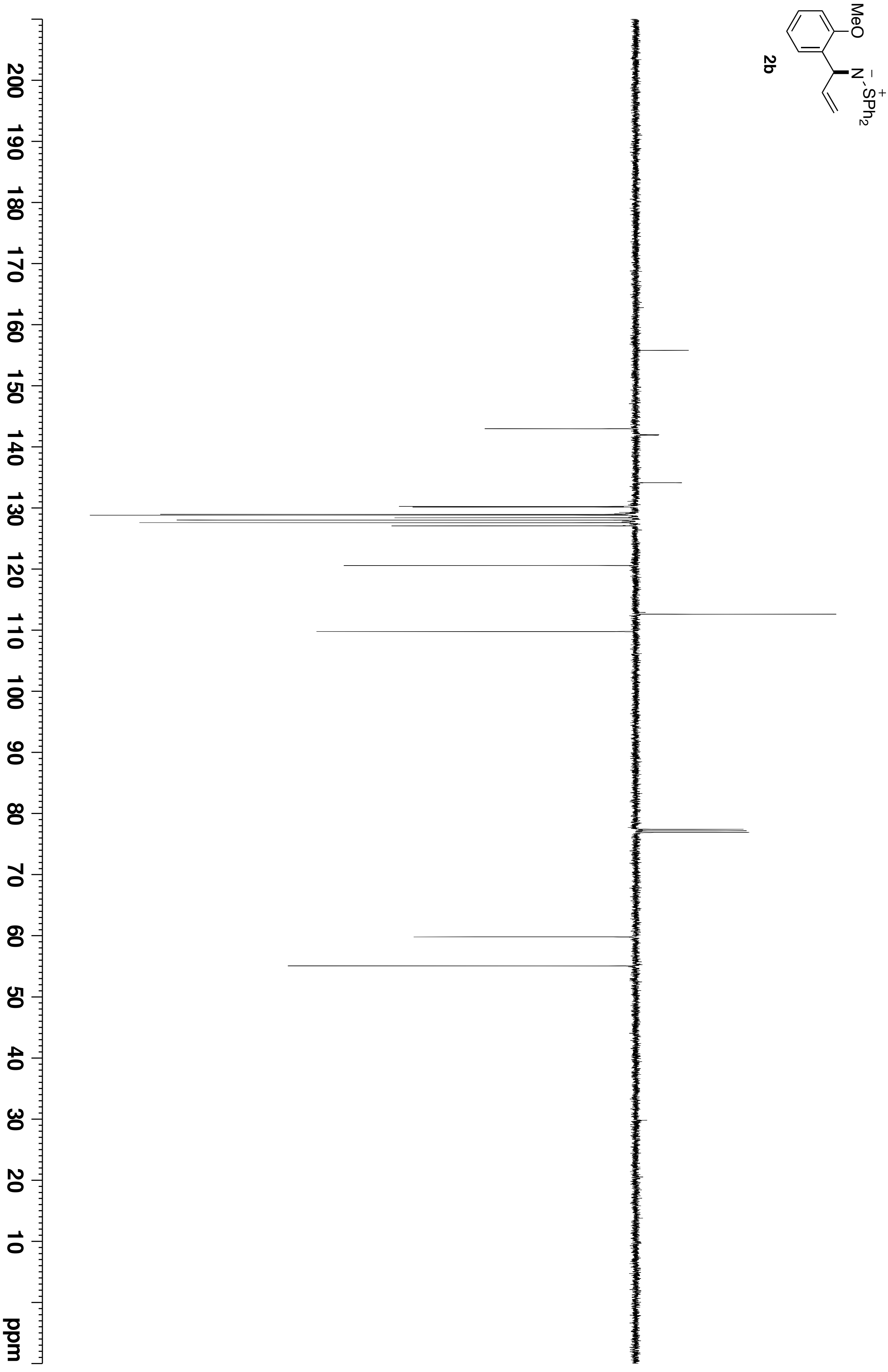
MeO
N
SPh2
2b
200
190
180
170
160
150
140
130
120
110
100
90
80
70
60
50
40
30
20
10
ppm

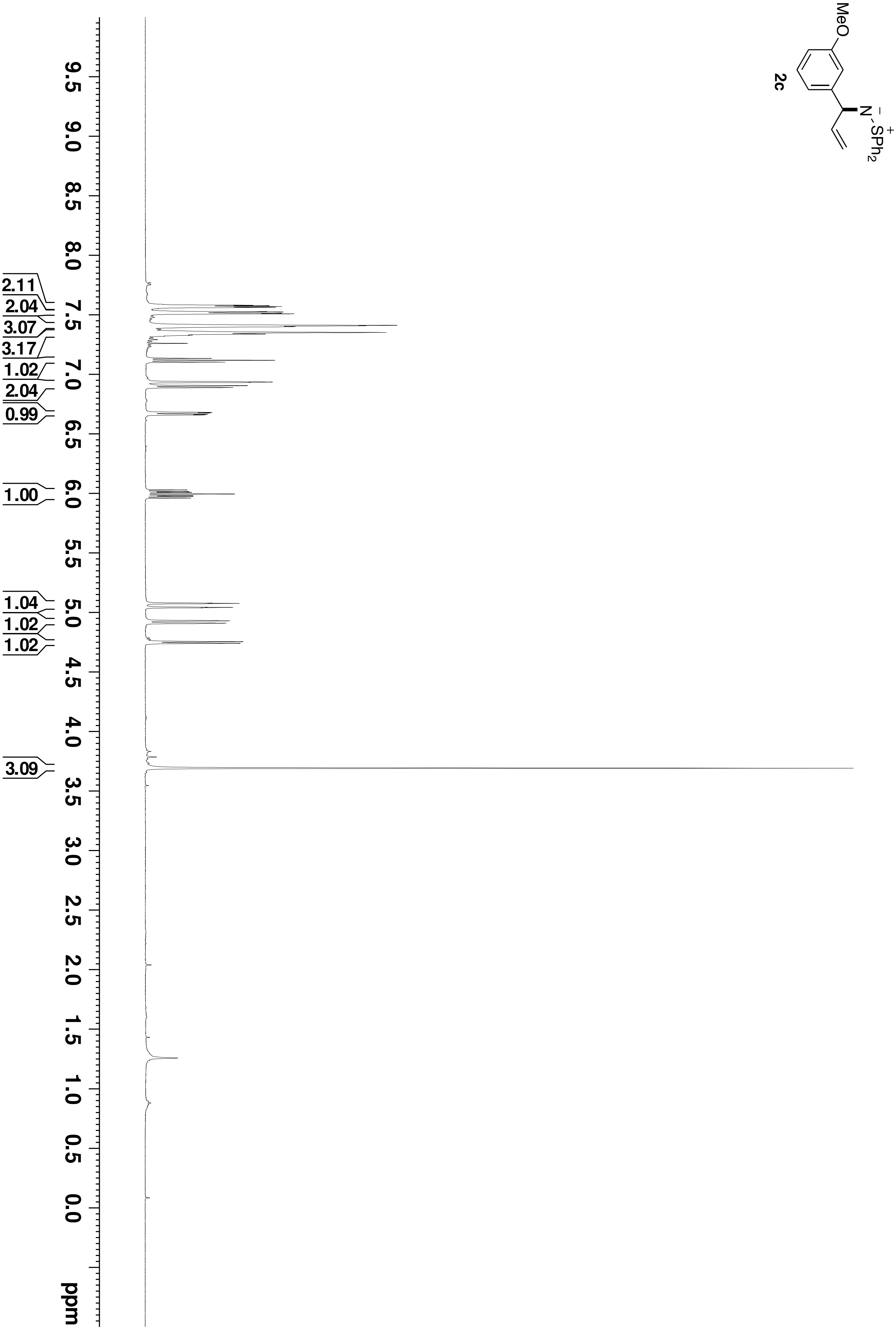
MeO
2c
ppm

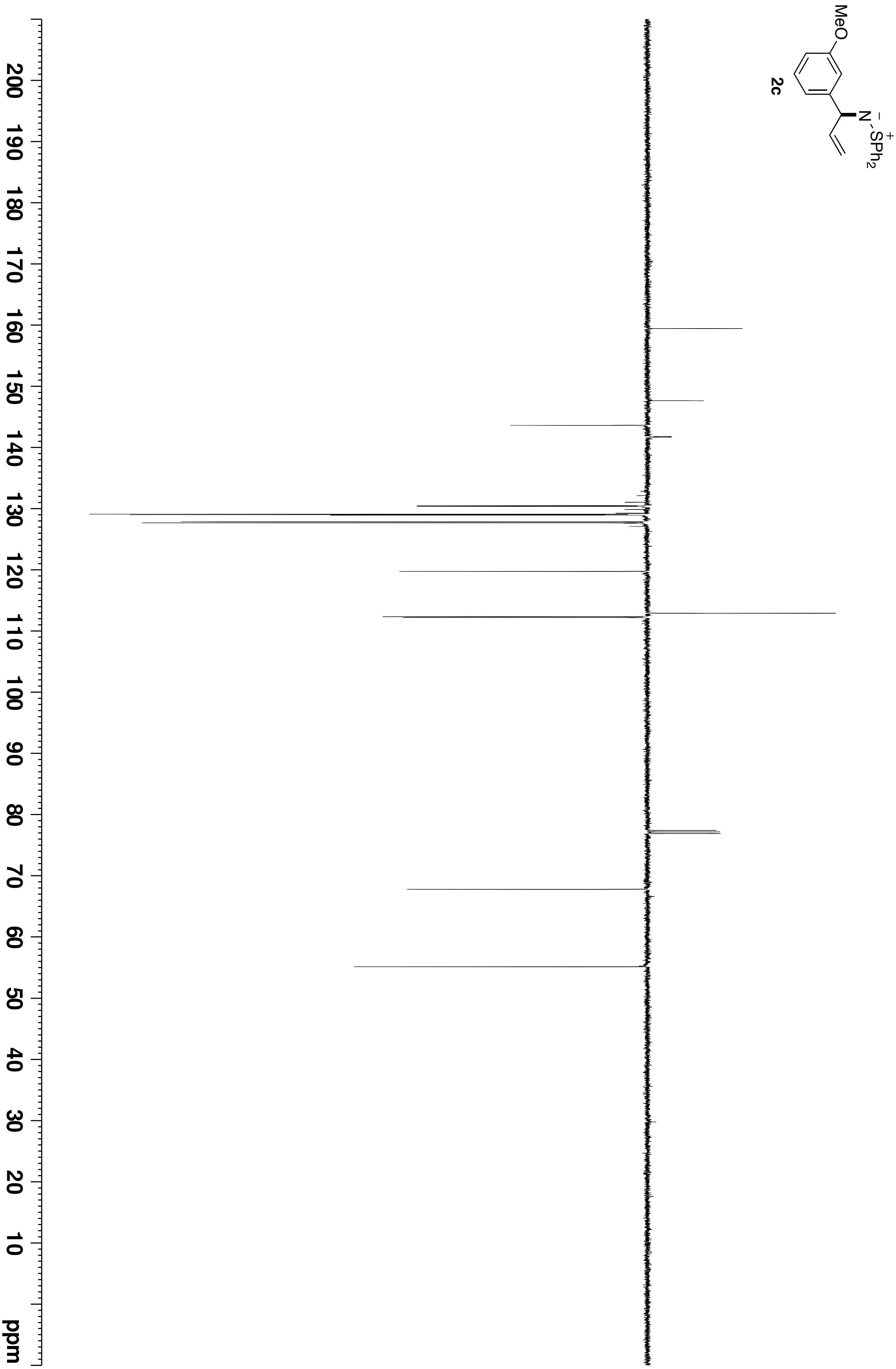

MeO
N
SPh2
2c
200 190 180 170 160 150 140 130 120 110 100 90 80 70 60 50 40 30 20 10 ppm

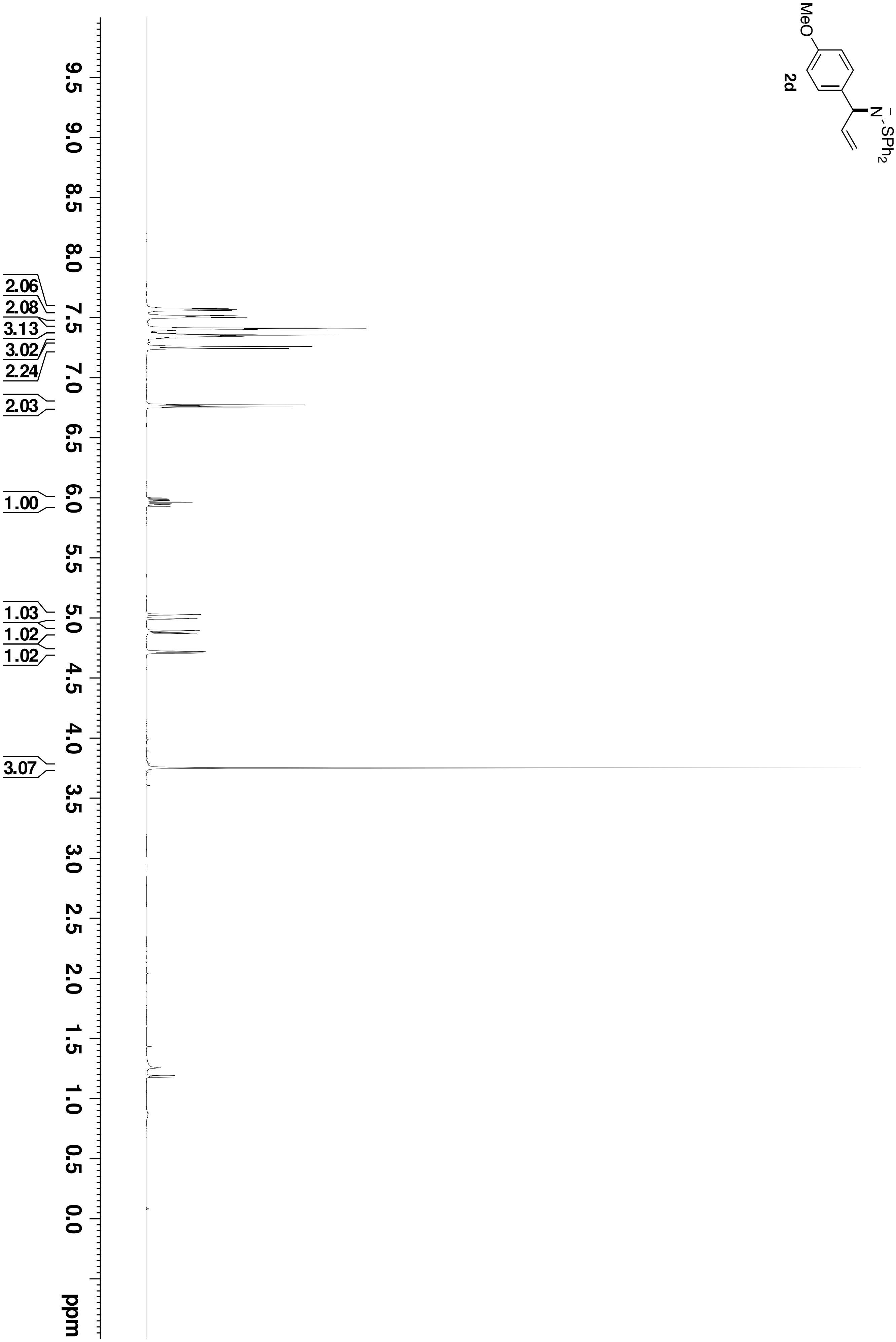

MeO
2d
N
SPh2
9.5
9.0
8.5
8.0
7.5
7.0
6.5
6.0
5.5
5.0
4.5
4.0
3.5
3.0
2.5
2.0
1.5
1.0
0.5
0.0
ppm
2.06
2.08
3.13
3.02
2.24
2.03
1.00
1.03
1.02
1.02
3.07

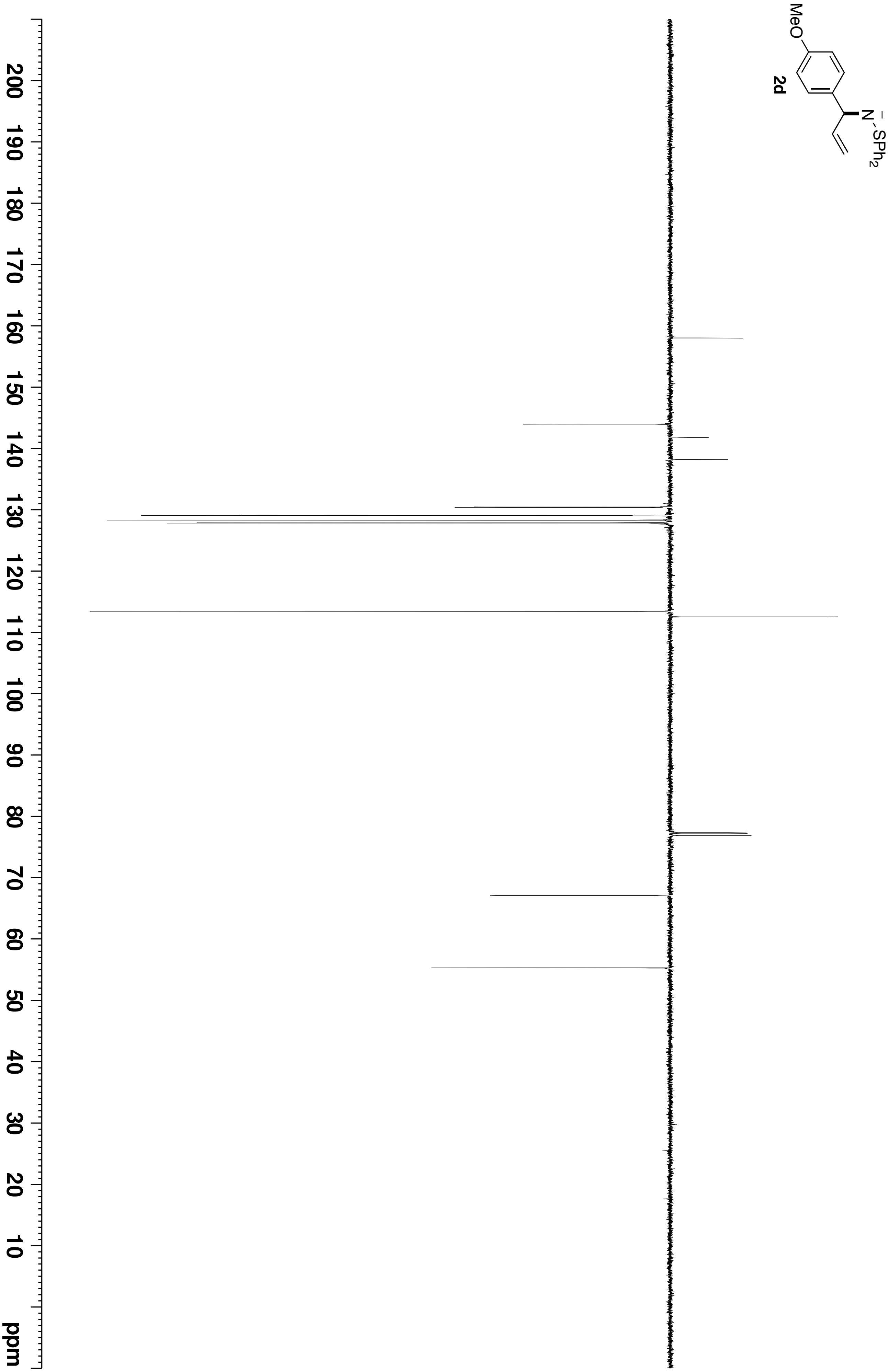
MeO
N
SPh2
2d
200
190
180
170
160
150
140
130
120
110
100
90
80
70
60
50
40
30
20
10
ppm

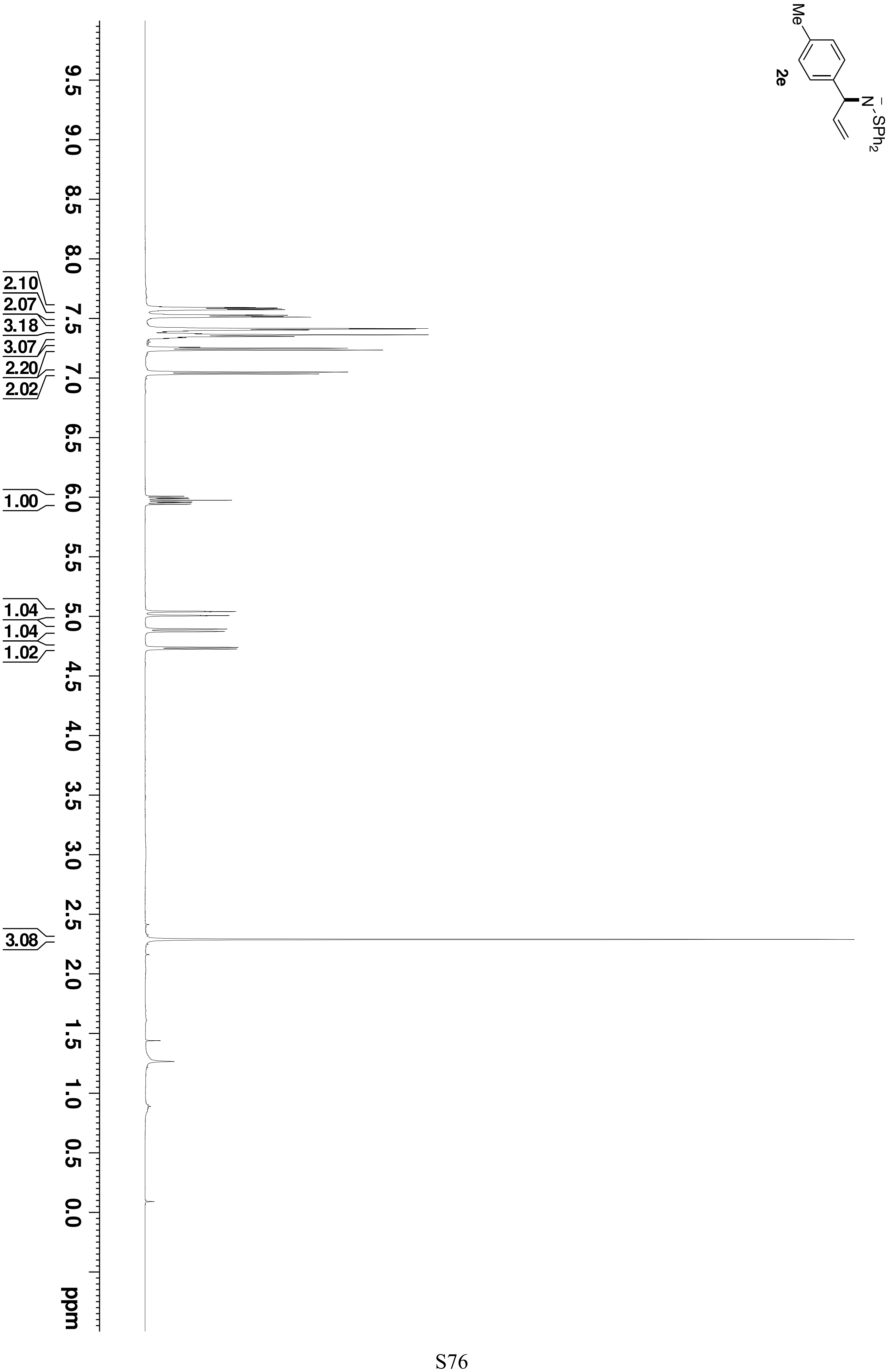
2e
Me
N
SPh2
ppm
9.5
9.0
8.5
8.0
7.5
7.0
6.5
6.0
5.5
5.0
4.5
4.0
3.5
3.0
2.5
2.0
1.5
1.0
0.5
0.0
2.10
2.07
3.18
3.07
2.20
2.02
1.00
1.04
1.04
1.02
3.08

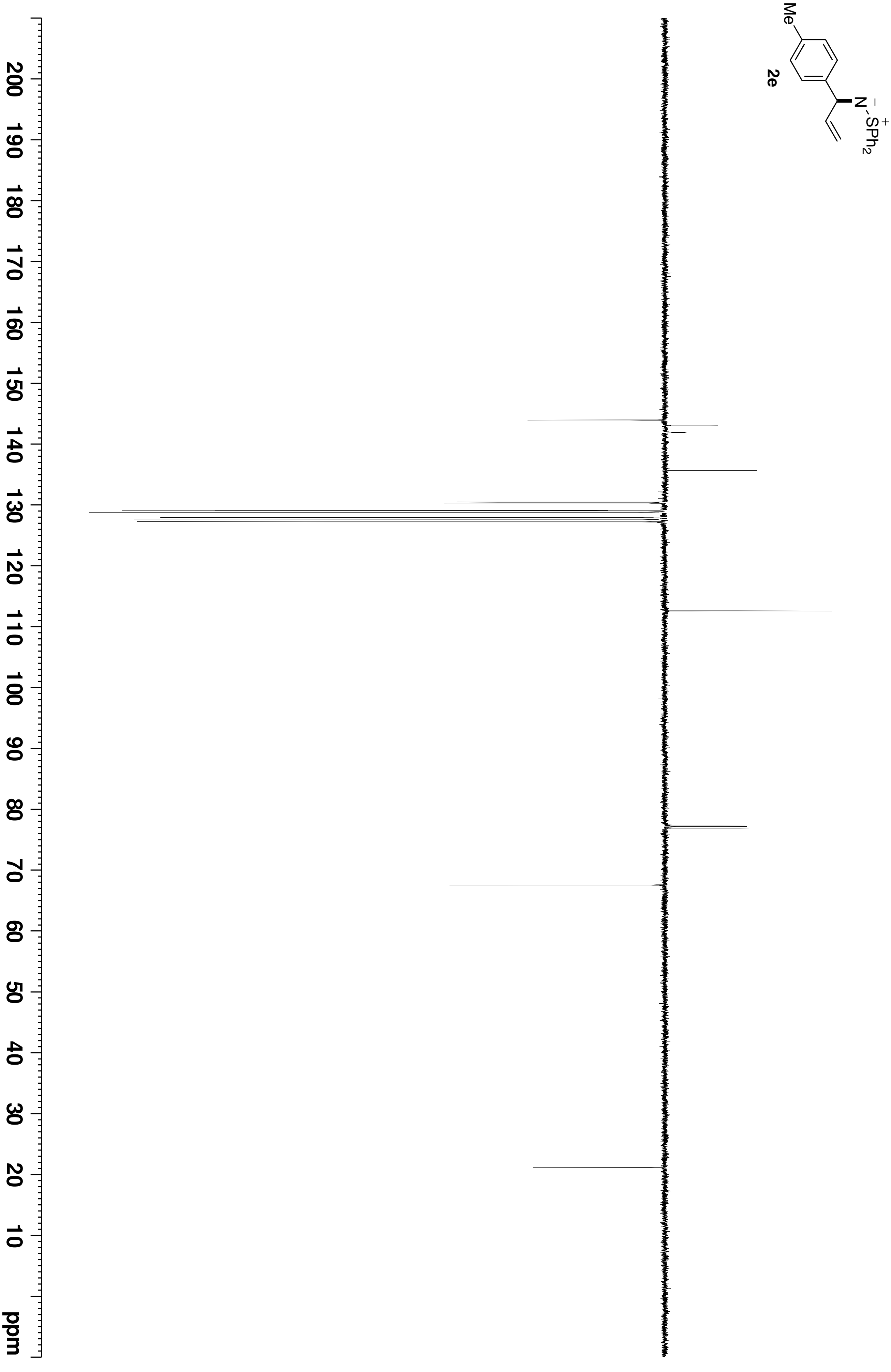

Me
2e
N
SPh2
200
190
180
170
160
150
140
130
120
110
100
90
80
70
60
50
40
30
20
10
ppm

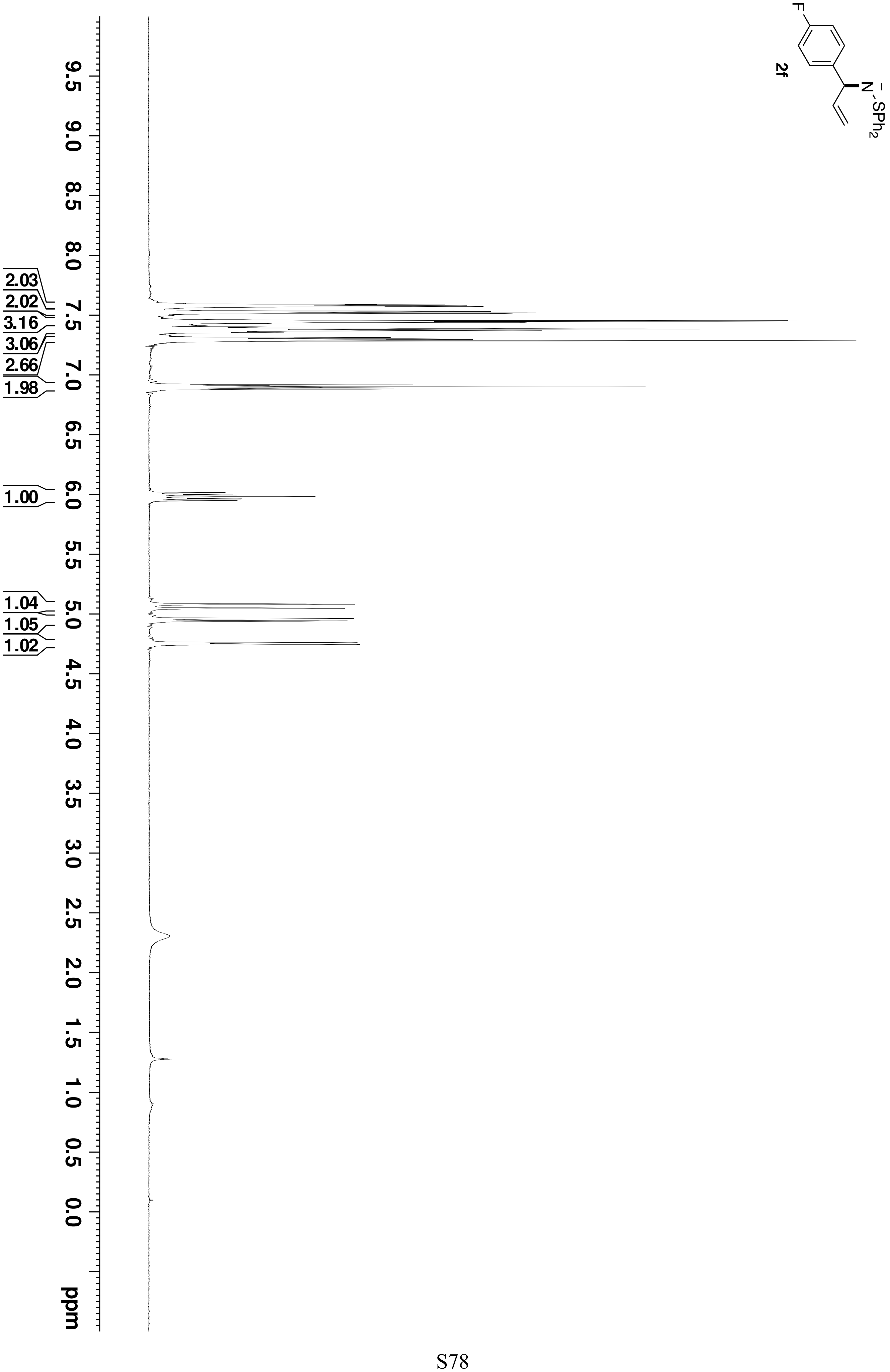

F
N
SPh2
2f
2.03
2.02
3.16
3.06
2.66
1.98
1.00
1.04
1.05
1.02
9.5
9.0
8.5
8.0
7.5
7.0
6.5
6.0
5.5
5.0
4.5
4.0
3.5
3.0
2.5
2.0
1.5
1.0
0.5
0.0
ppm

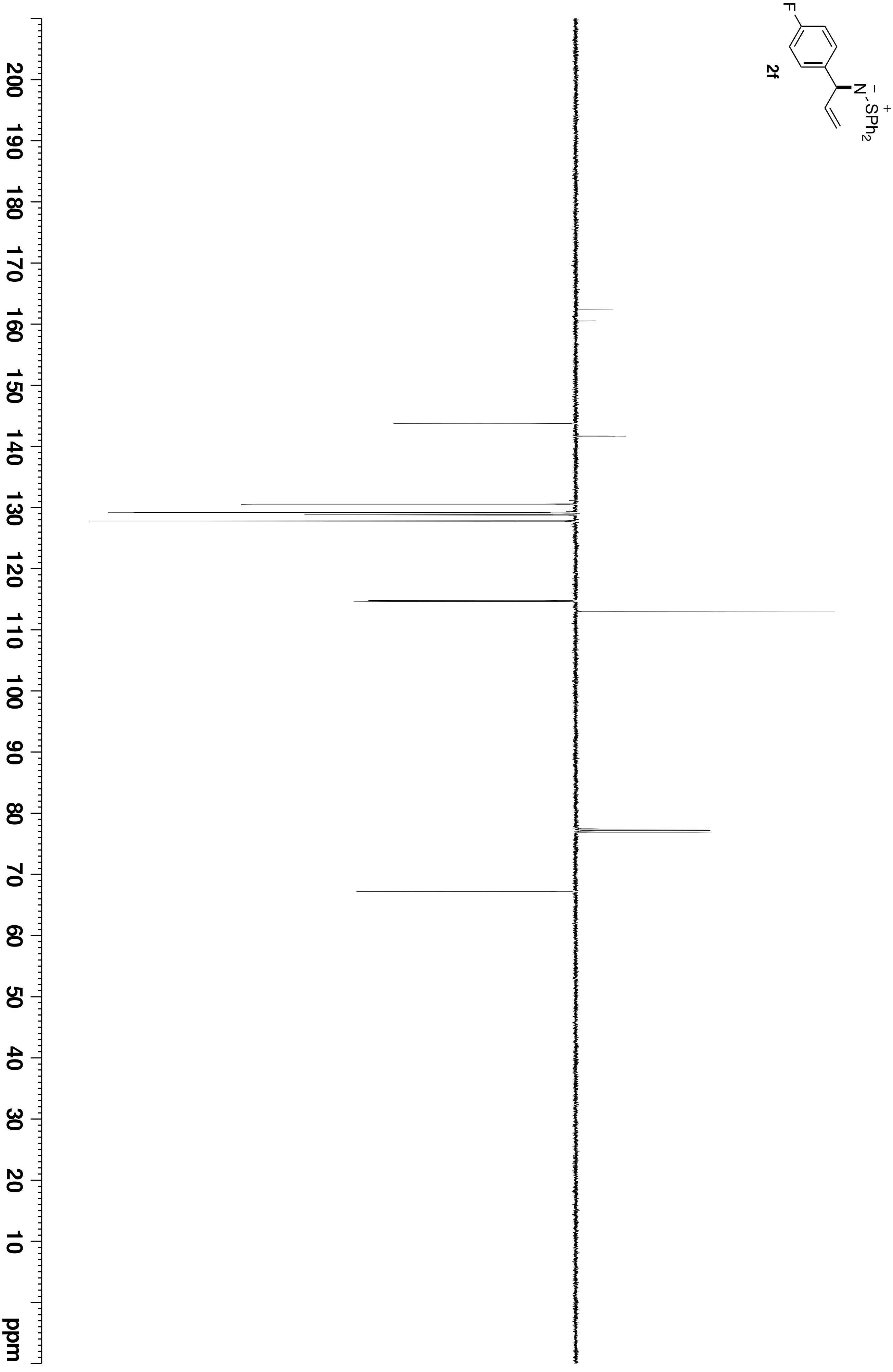
F
N
SPh2
2f
200
190
180
170
160
150
140
130
120
110
100
90
80
70
60
50
40
30
20
10
ppm

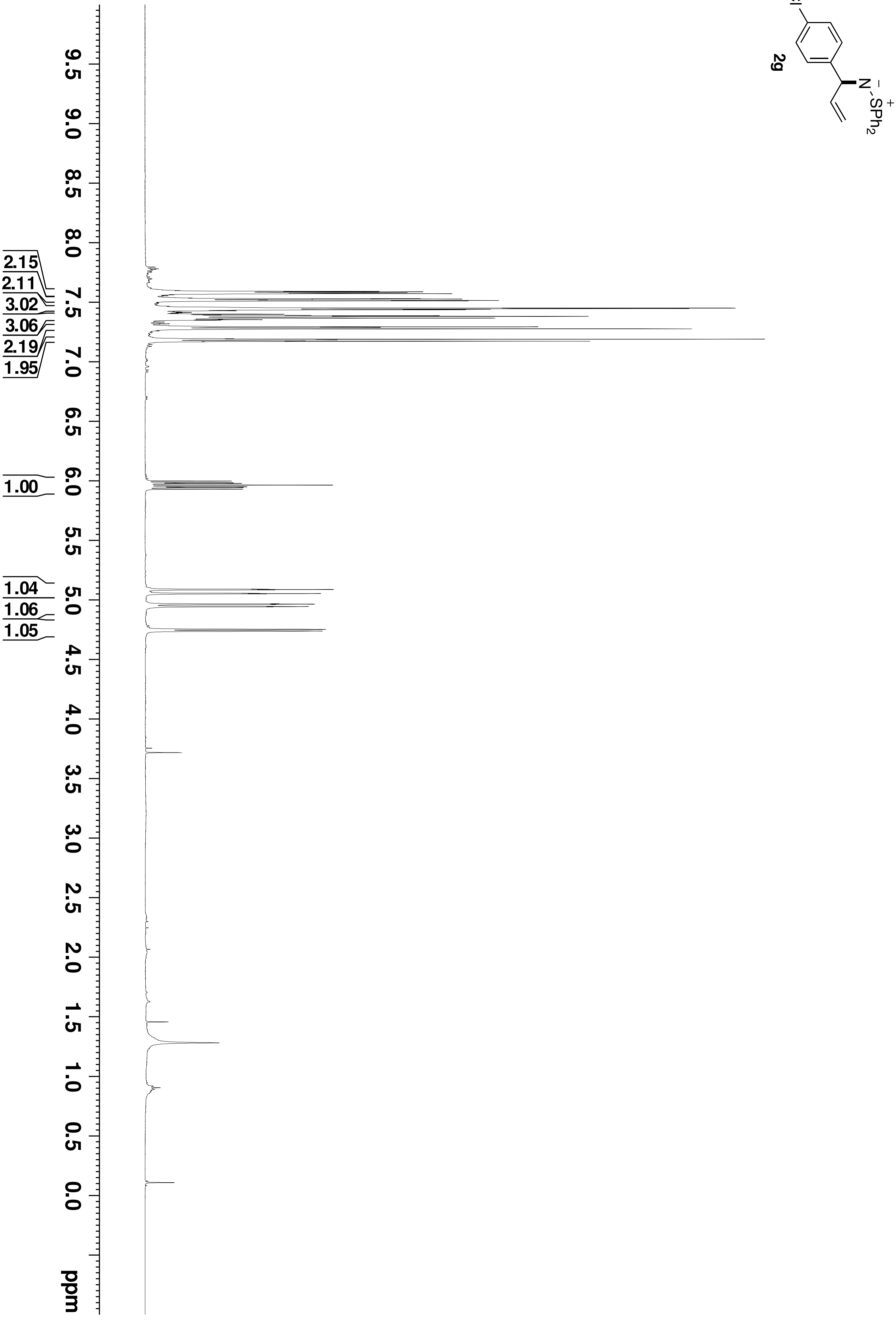
2g
ppm

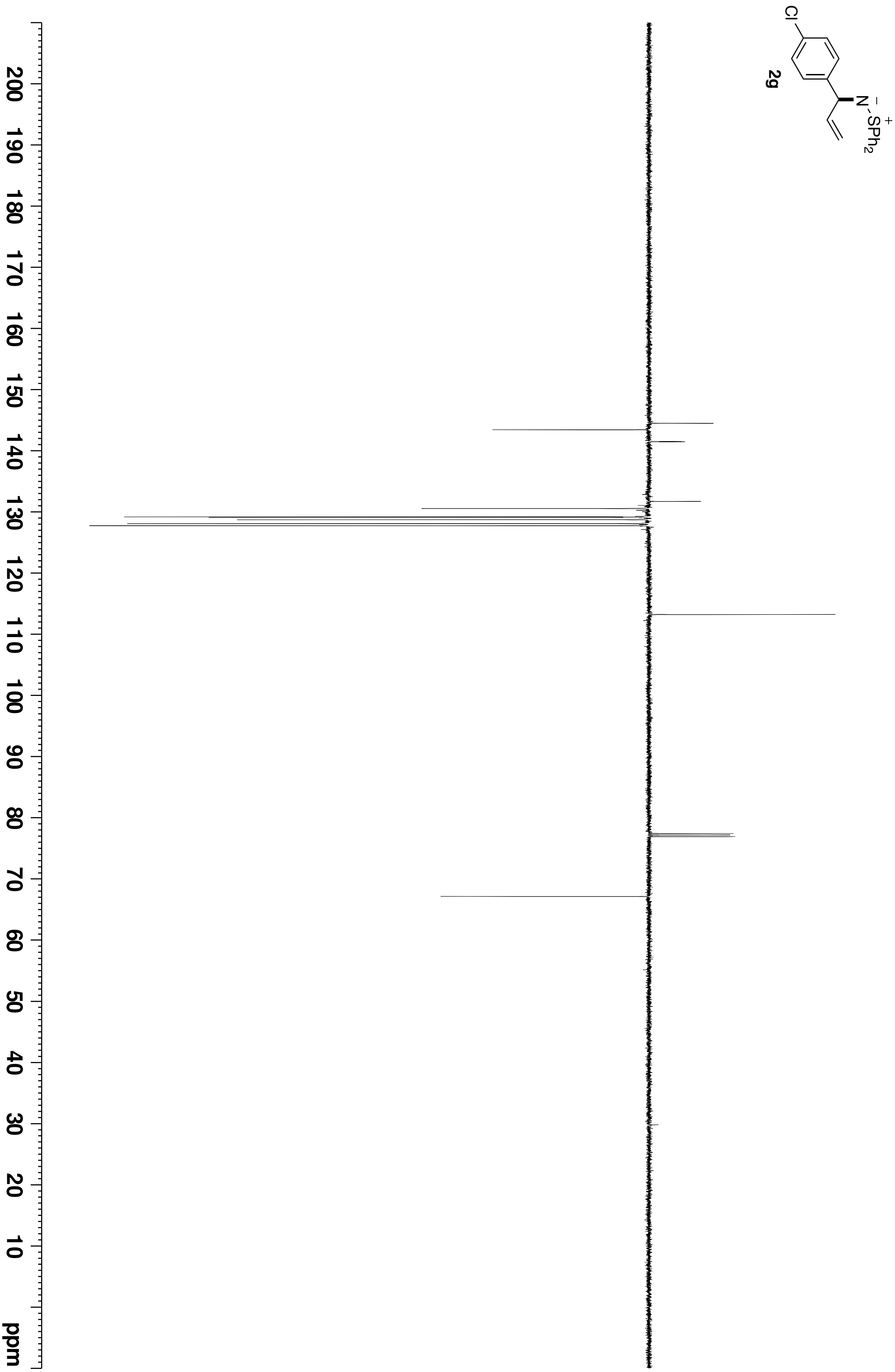
Cl
N
SPh2
2g
200
190
180
170
160
150
140
130
120
110
100
90
80
70
60
50
40
30
20
10
ppm

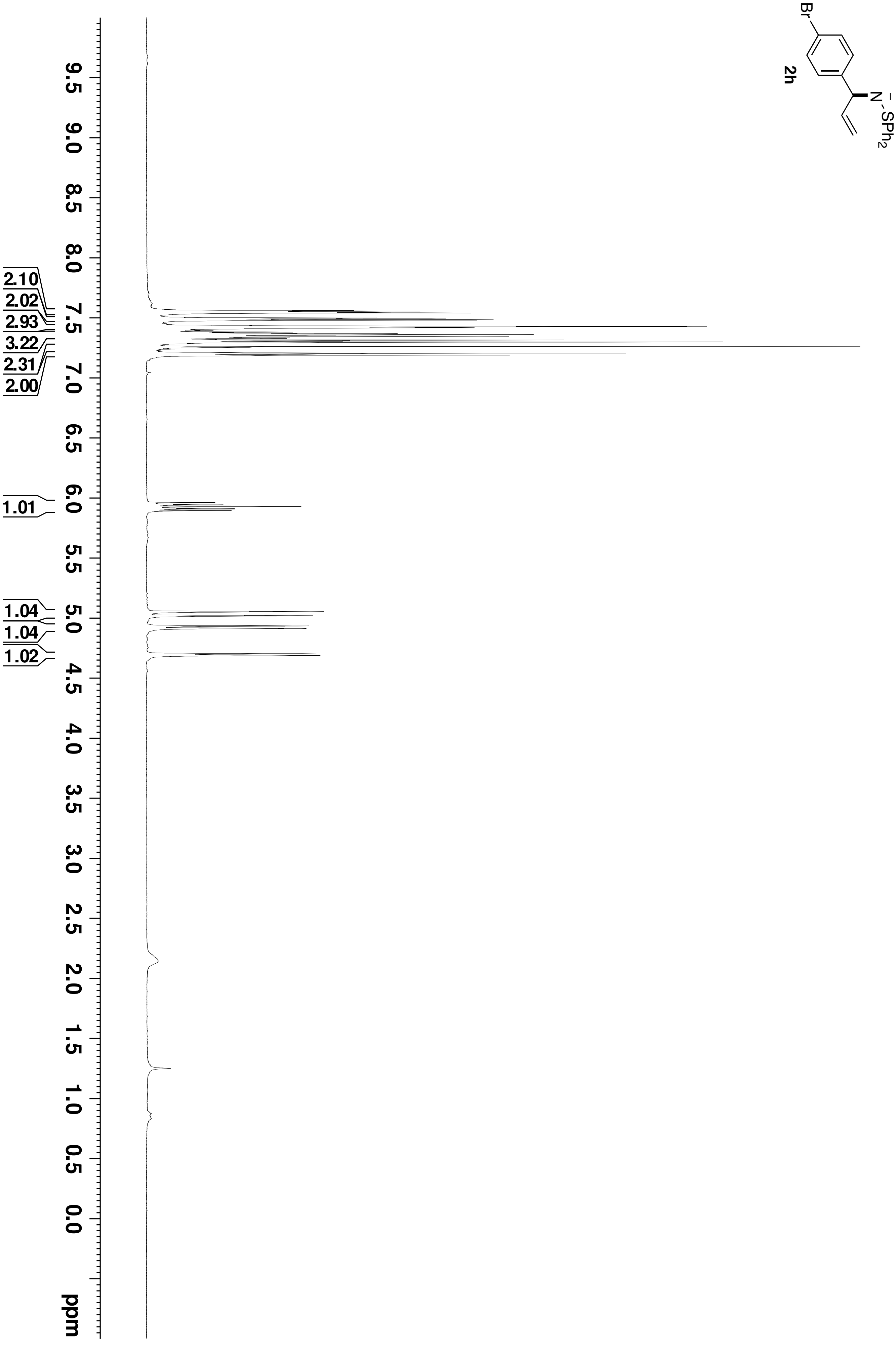

2h
Br
N
SPh2
9.5
9.0
8.5
8.0
7.5
7.0
6.5
6.0
5.5
5.0
4.5
4.0
3.5
3.0
2.5
2.0
1.5
1.0
0.5
0.0
ppm
2.10
2.02
2.93
3.22
2.31
2.00
1.01
1.04
1.04
1.02

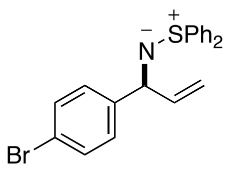
N
SPh2
Br

2h

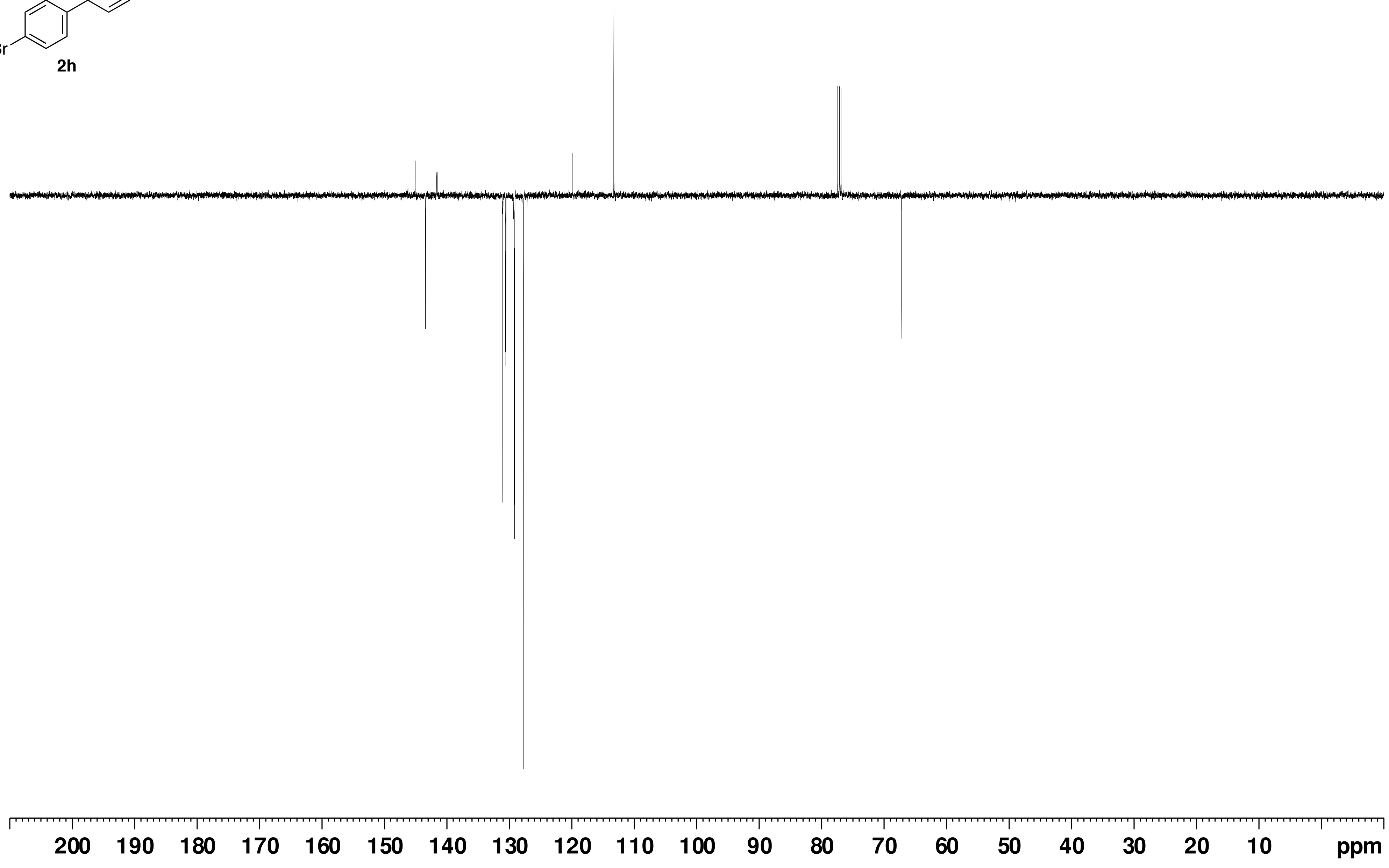
200 190 180 170 160 150 140 130 120 110 100 90 80 70 60 50 40 30 20 10 ppm

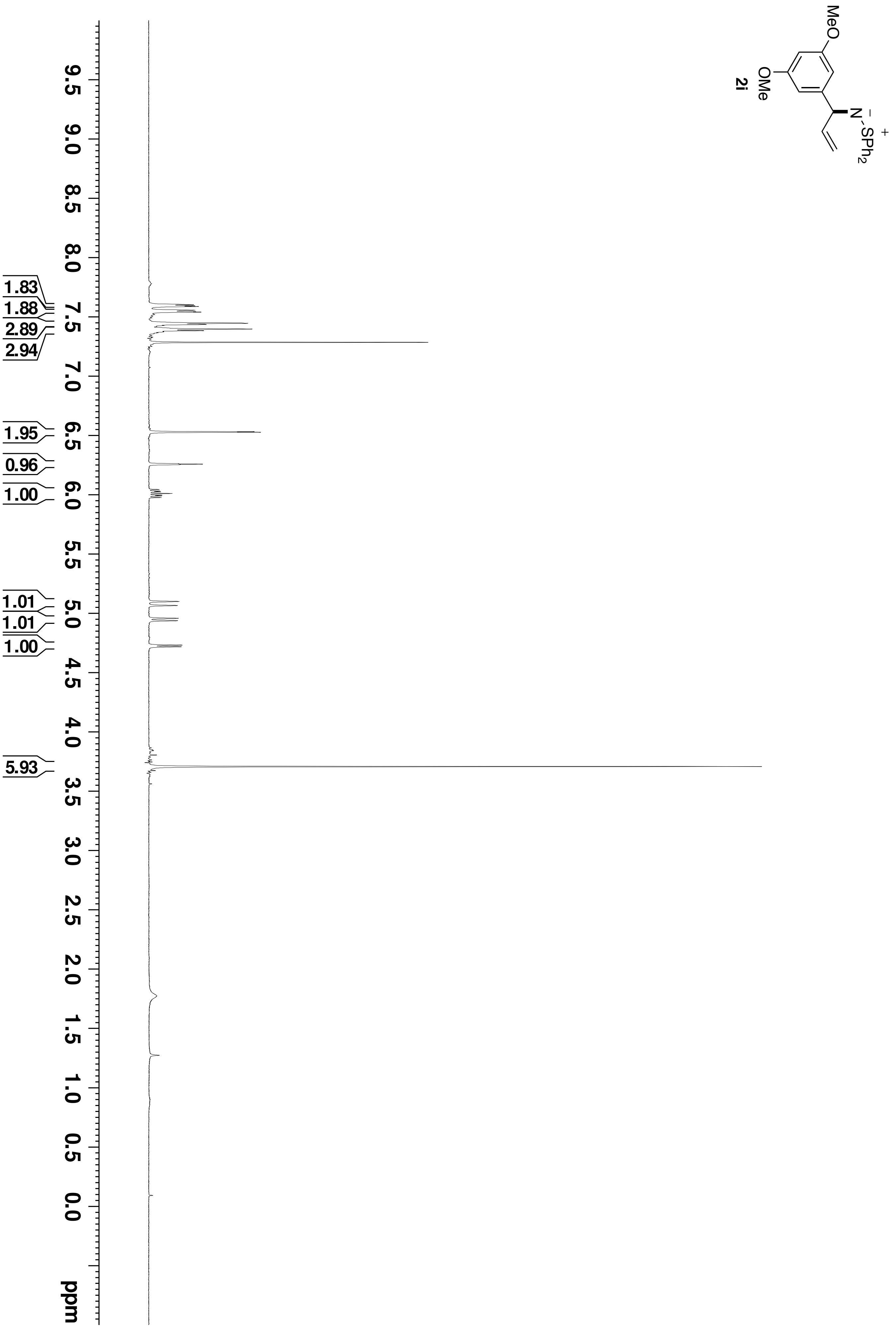
MeO
OMe
2i
N
SPh2
ppm
1.83
1.88
2.89
2.94
1.95
0.96
1.00
1.01
1.01
1.00
5.93

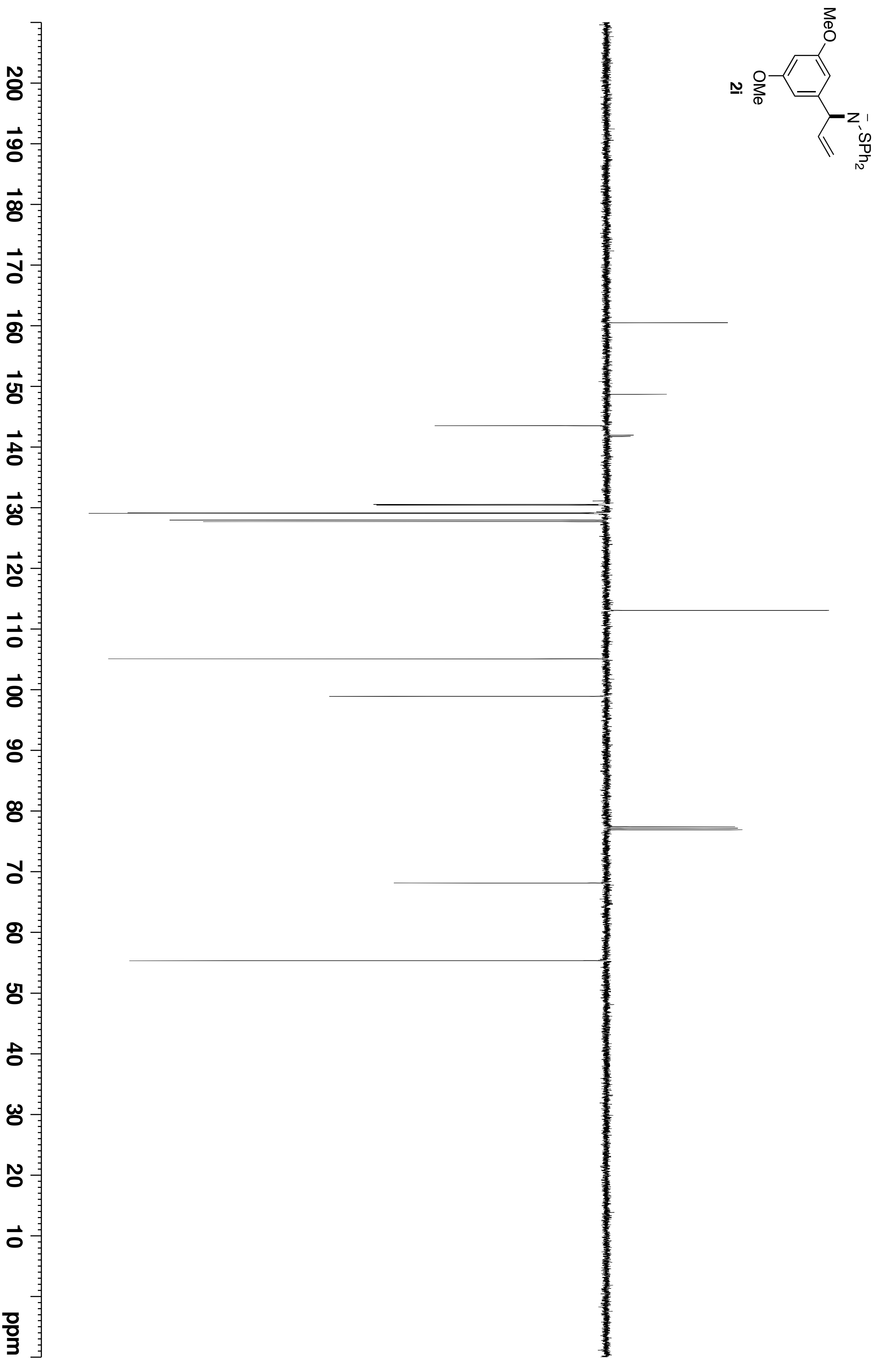

MeO
OMe
N
SPh2
2i
200 190 180 170 160 150 140 130 120 110 100 90 80 70 60 50 40 30 20 10
ppm

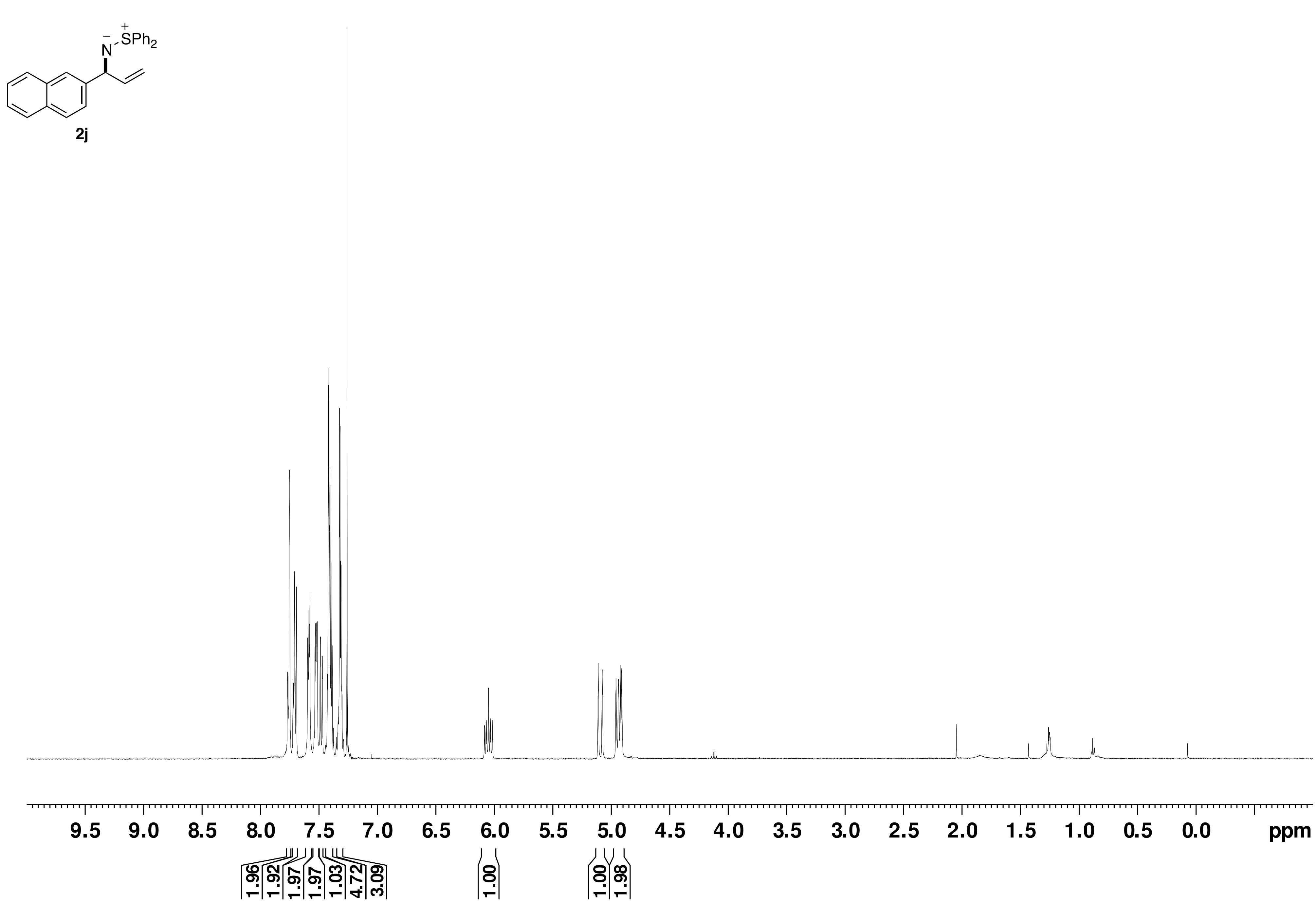

SPh2
N
2j
9.5
9.0
8.5
8.0
7.5
7.0
6.5
6.0
5.5
5.0
4.5
4.0
3.5
3.0
2.5
2.0
1.5
1.0
0.5
0.0
ppm
1.96
1.92
1.97
1.97
1.03
4.72
3.09
1.00
1.00
1.98

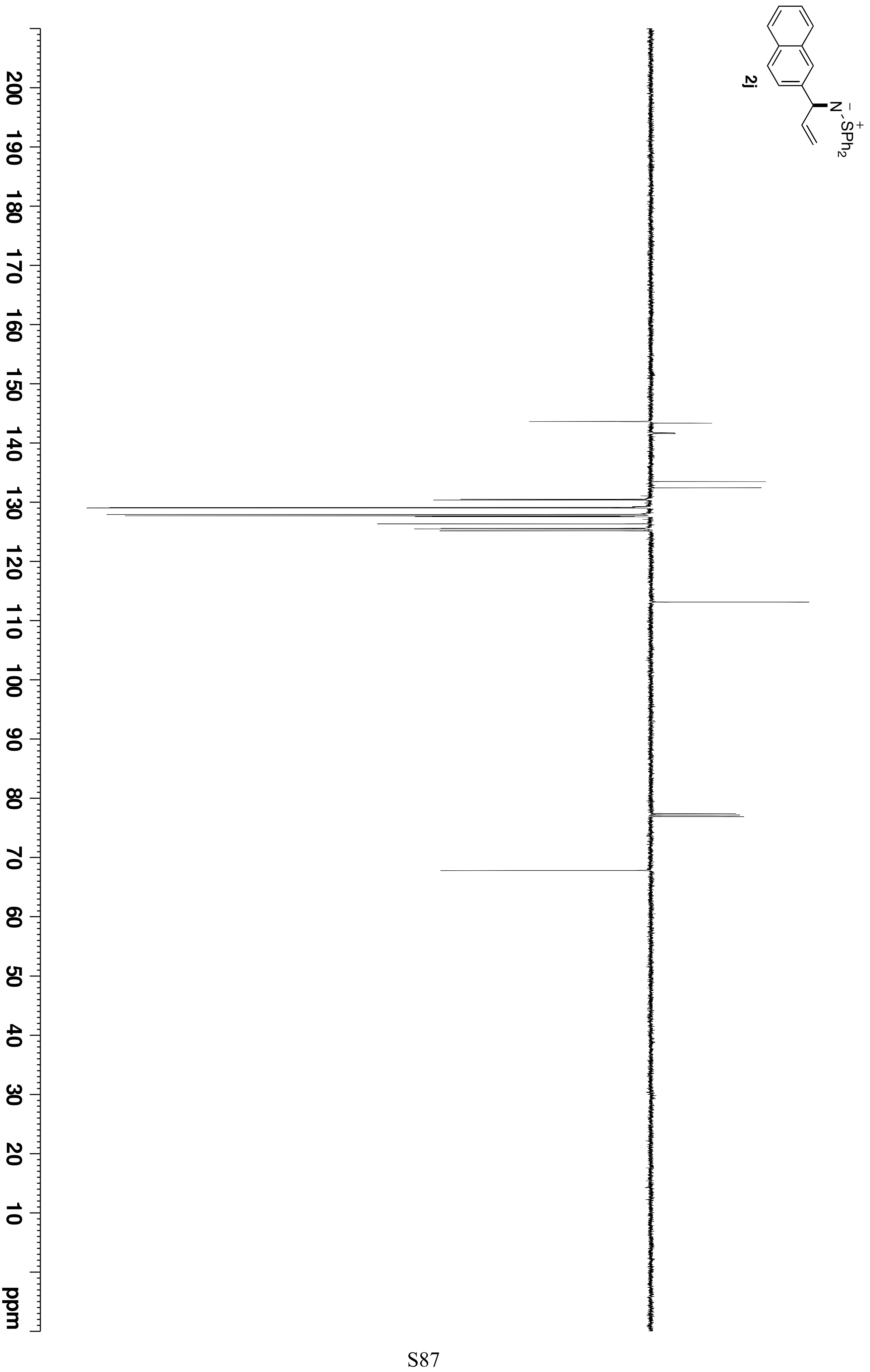
2j
N
SPh2
200
190
180
170
160
150
140
130
120
110
100
90
80
70
60
50
40
30
20
10
ppm

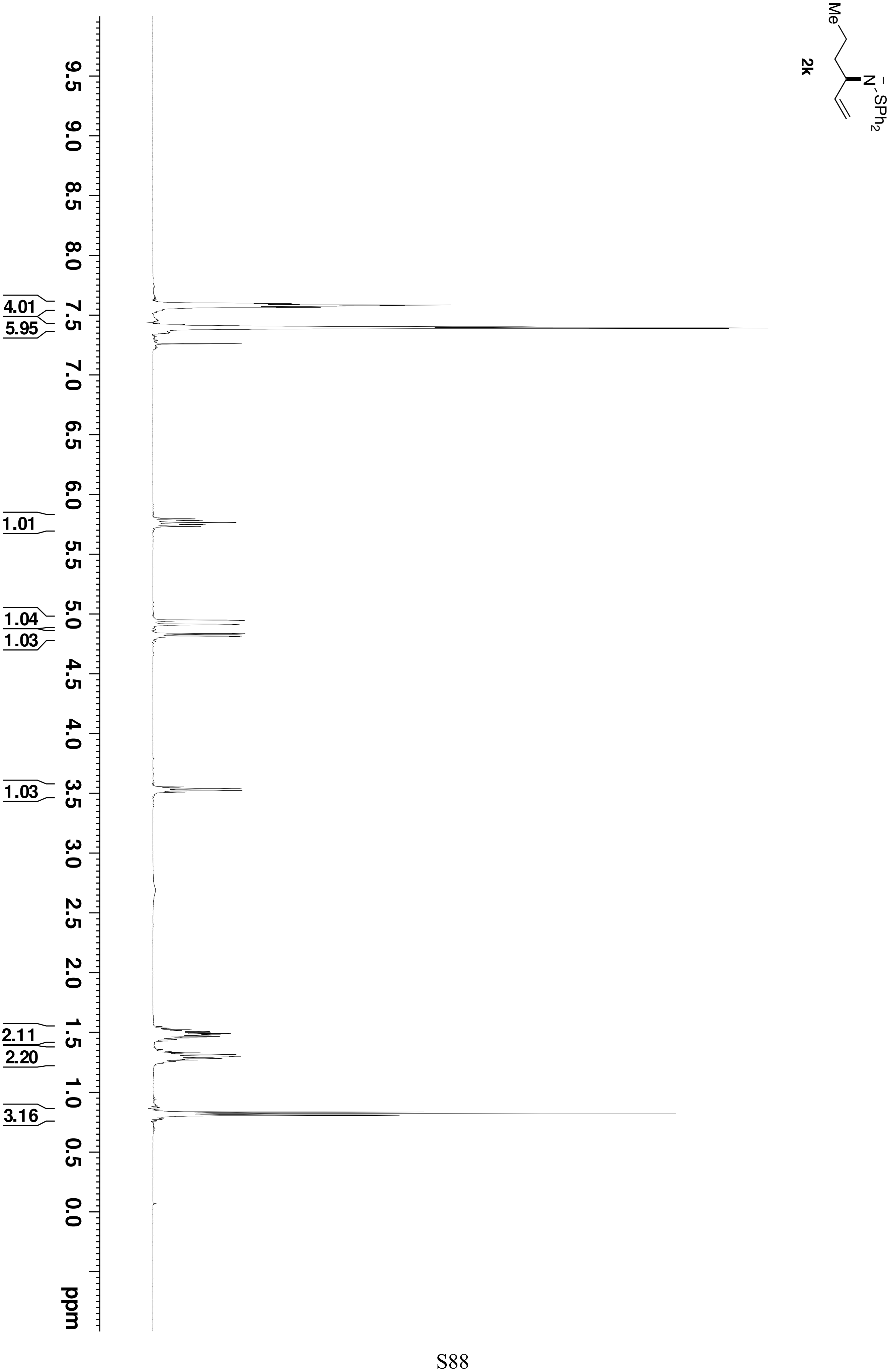

Me
N
SPh$_2$
2k
9.5
9.0
8.5
8.0
7.5
7.0
6.5
6.0
5.5
5.0
4.5
4.0
3.5
3.0
2.5
2.0
1.5
1.0
0.5
0.0
ppm
4.01
5.95
1.01
1.04
1.03
1.03
2.11
2.20
3.16

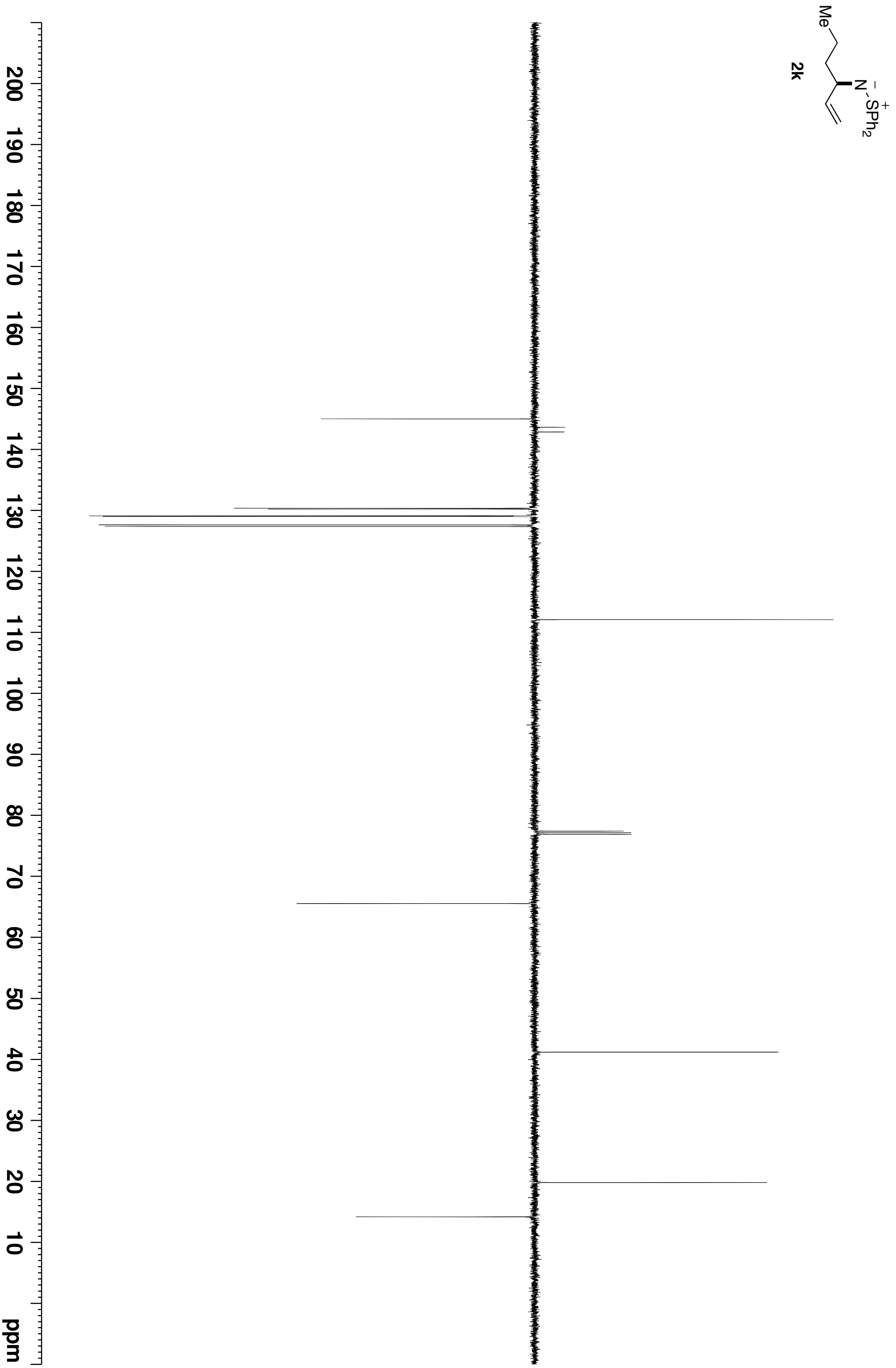
Me
N
SPh2
2k
200 190 180 170 160 150 140 130 120 110 100 90 80 70 60 50 40 30 20 10 ppm

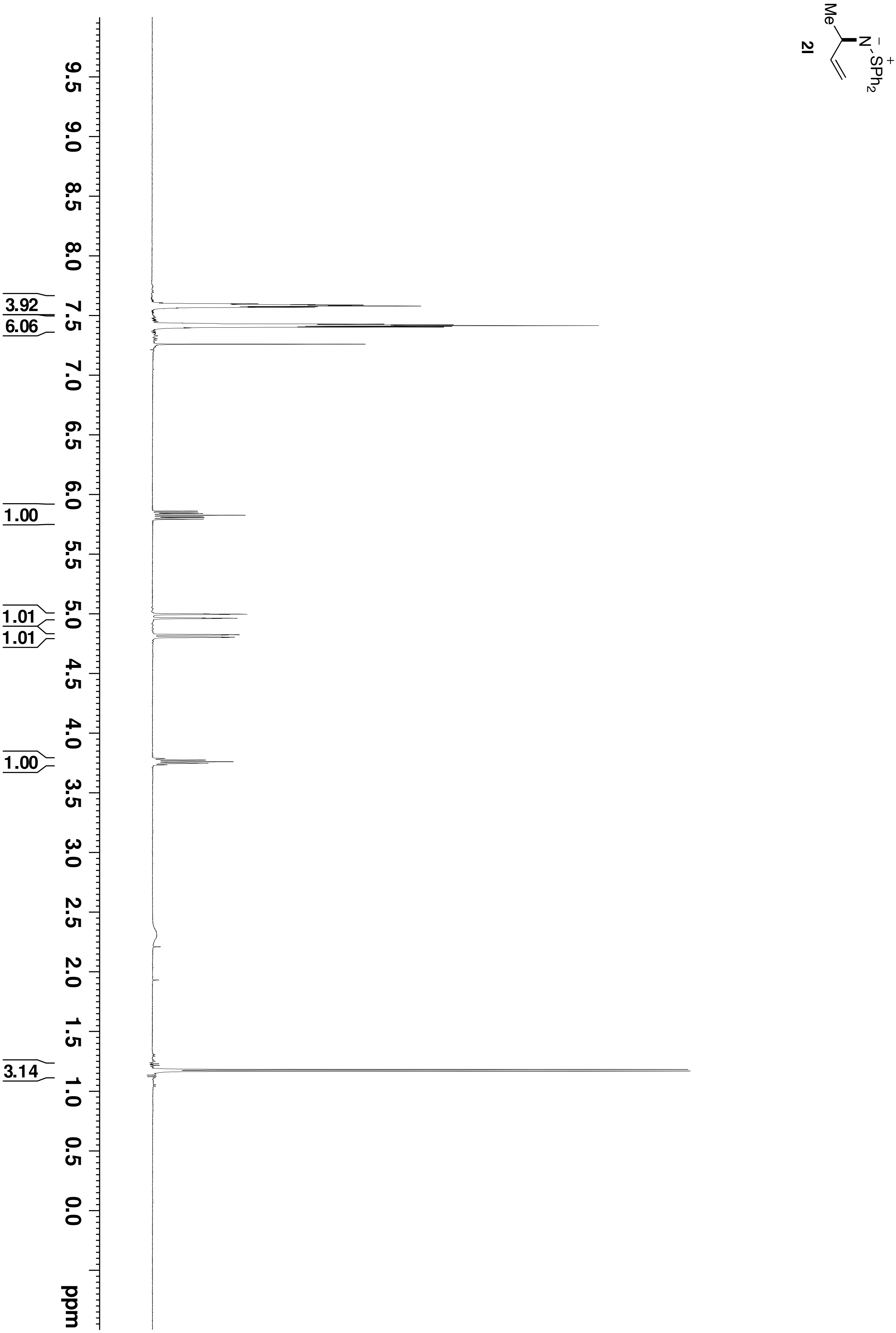

2l
ppm
3.92
6.06
1.00
1.01
1.01
1.00
3.14

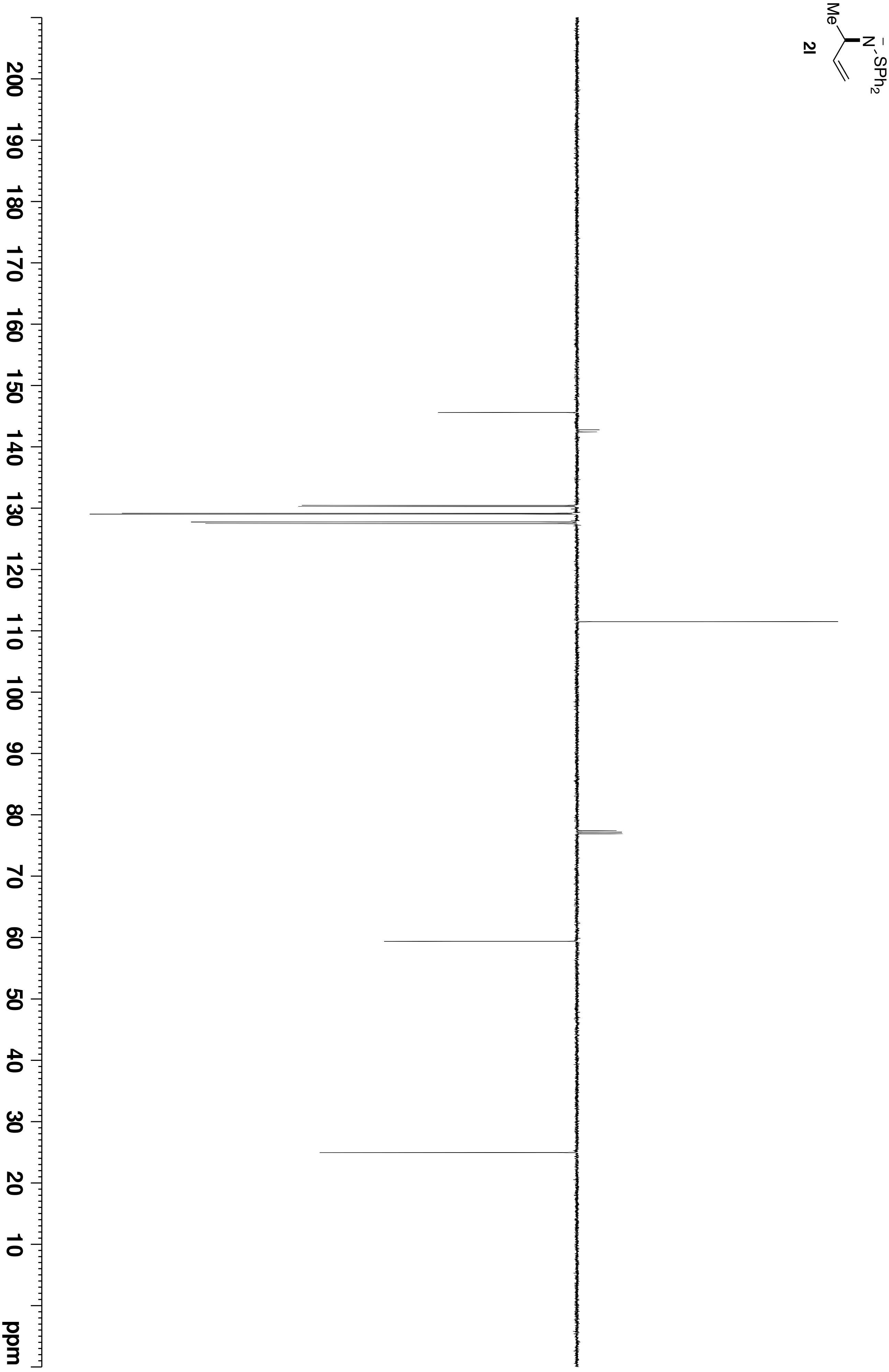
Me
N
SPh2
2l
200
190
180
170
160
150
140
130
120
110
100
90
80
70
60
50
40
30
20
10
ppm

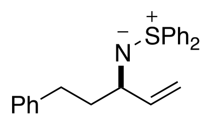
SPh2
N
Ph

2m

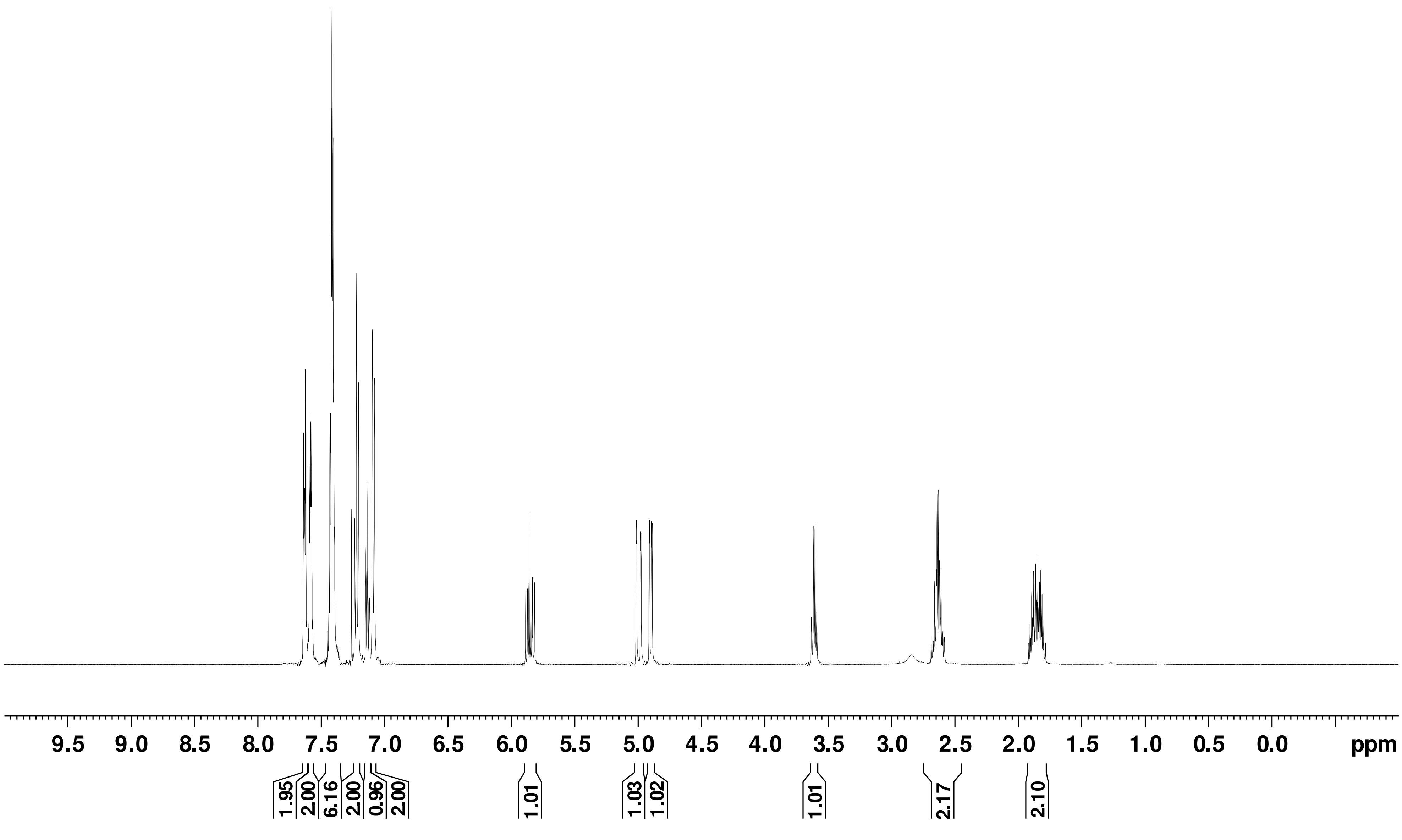
9.5 9.0 8.5 8.0 7.5 7.0 6.5 6.0 5.5 5.0 4.5 4.0 3.5 3.0 2.5 2.0 1.5 1.0 0.5 0.0 ppm
1.95
2.00
6.16
2.00
0.96
2.00
1.01
1.03
1.02
1.01
2.17
2.10

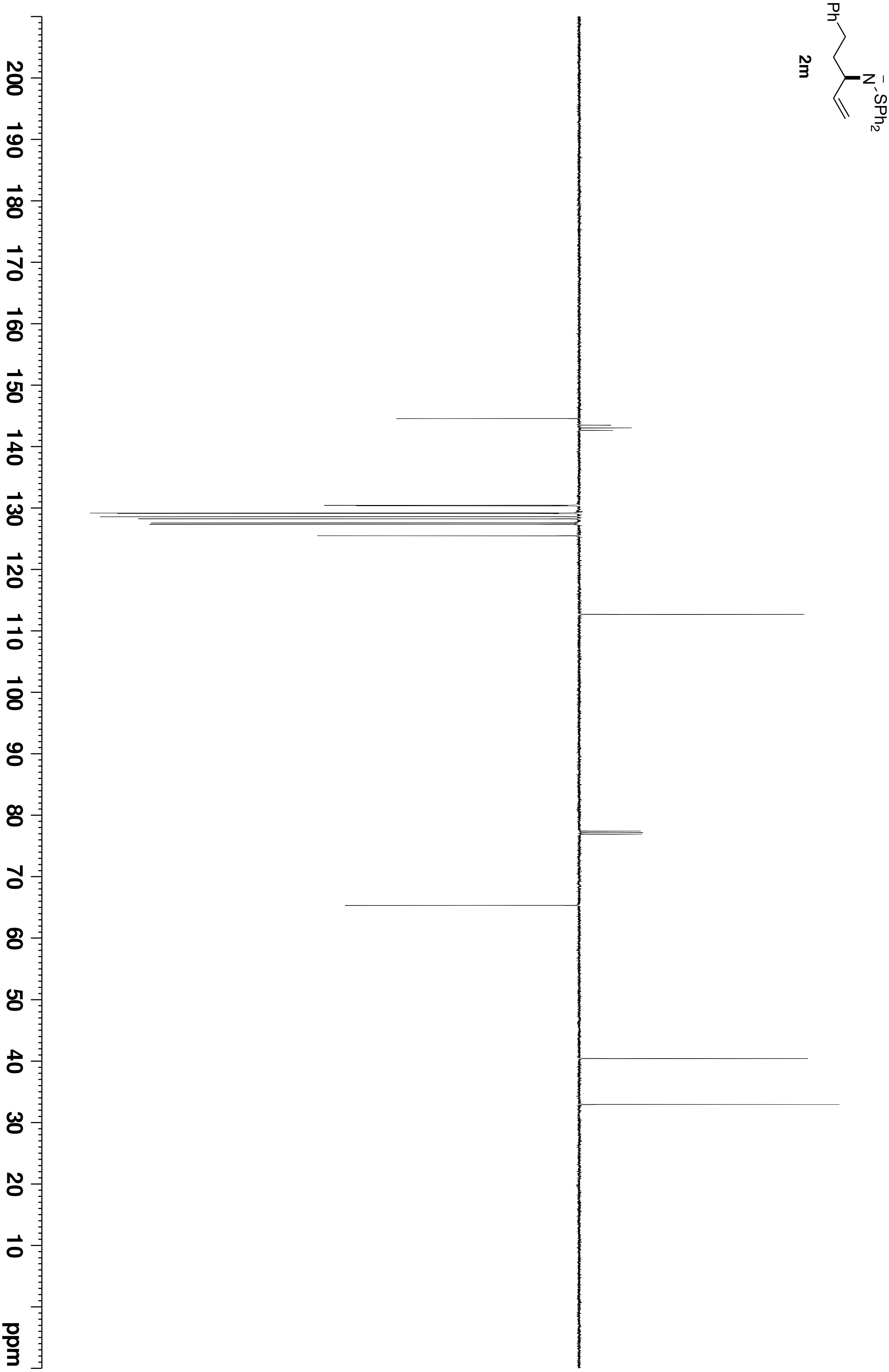

Ph
N
SPh2
2m
200
190
180
170
160
150
140
130
120
110
100
90
80
70
60
50
40
30
20
10
ppm

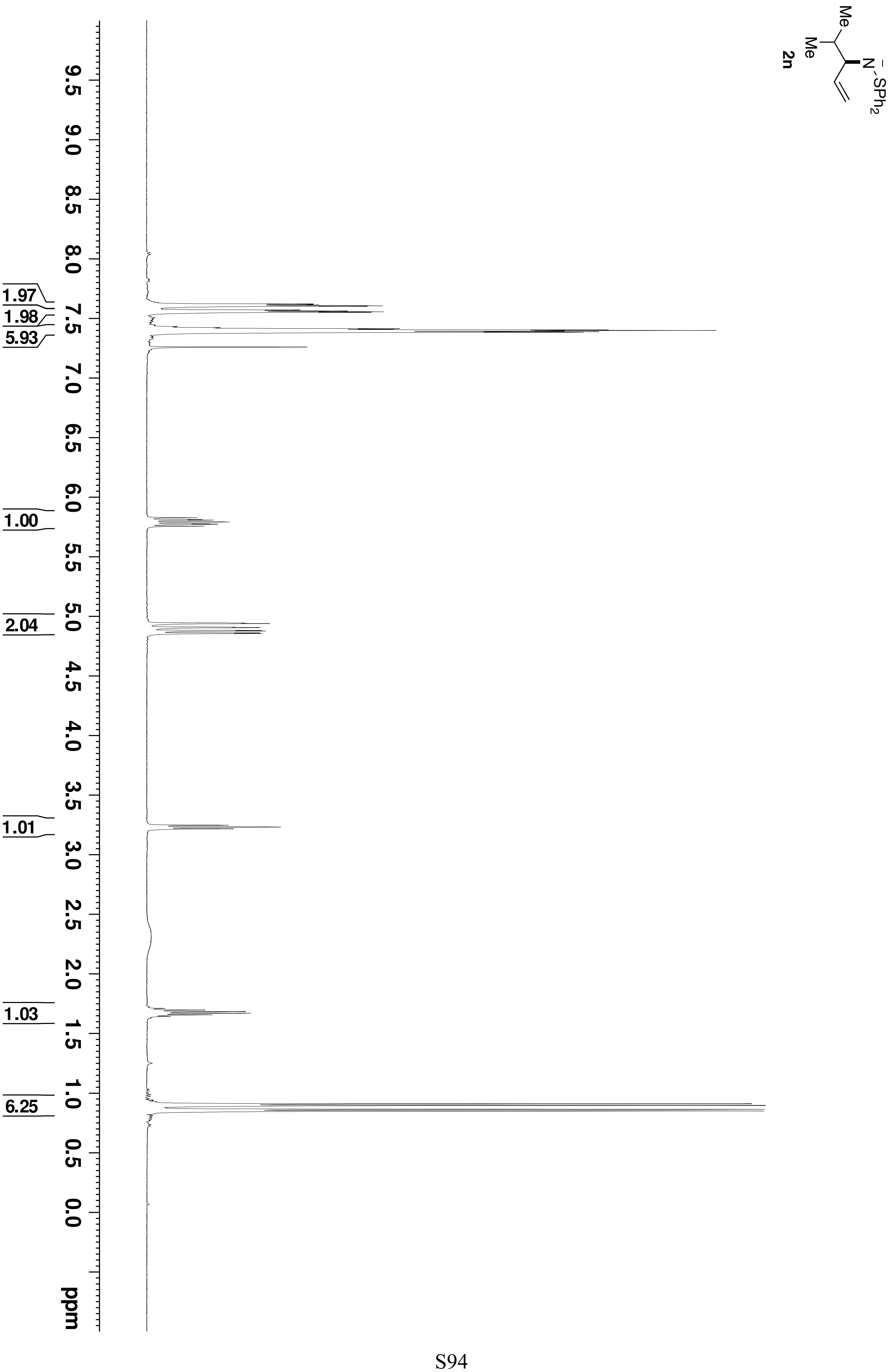
Me
Me
N
SPh2
2n
9.5
9.0
8.5
8.0
7.5
7.0
6.5
6.0
5.5
5.0
4.5
4.0
3.5
3.0
2.5
2.0
1.5
1.0
0.5
0.0
ppm
1.97
1.98
5.93
1.00
2.04
1.01
1.03
6.25

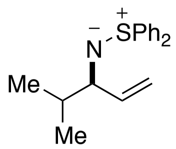

2n

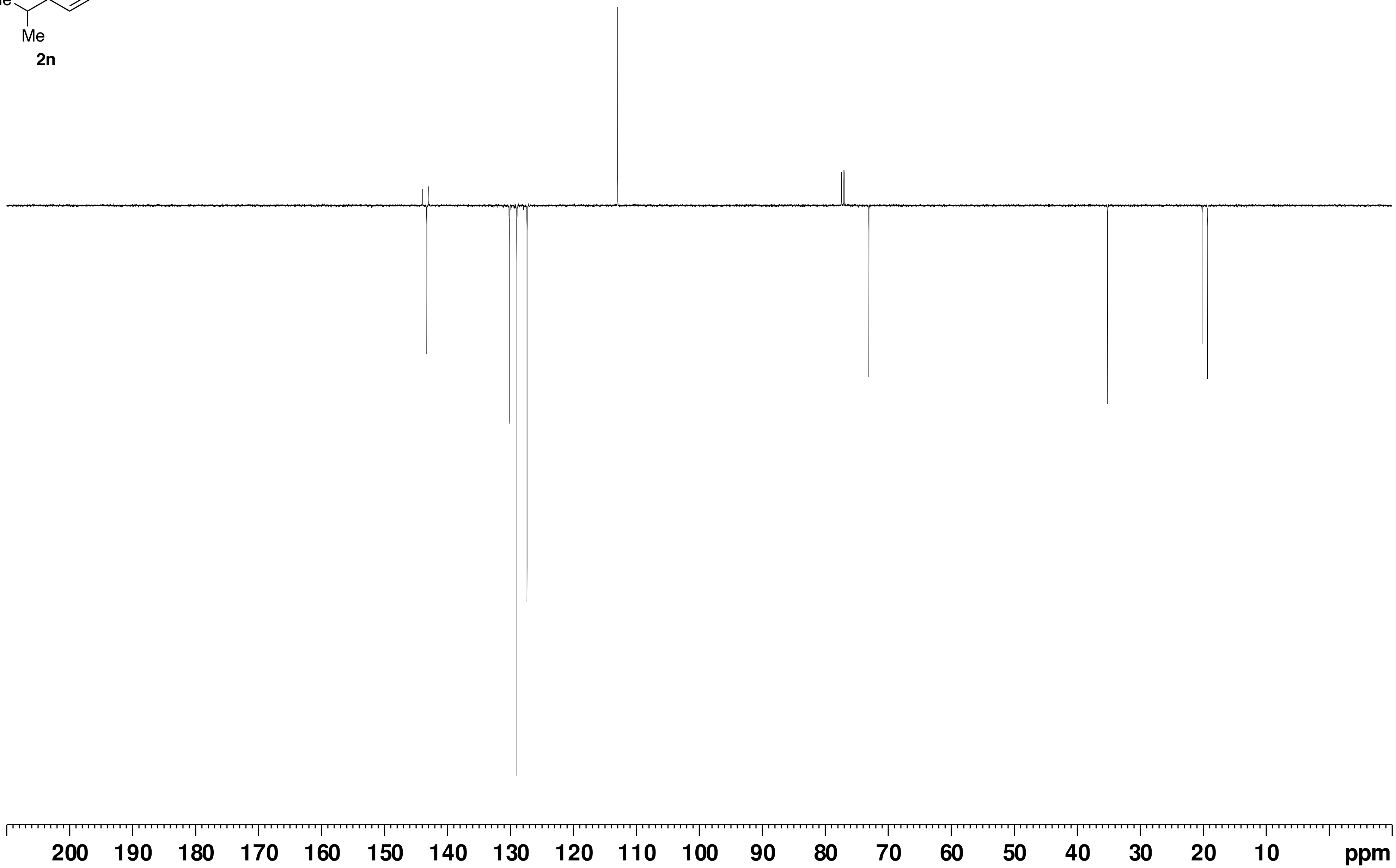
200 190 180 170 160 150 140 130 120 110 100 90 80 70 60 50 40 30 20 10 ppm

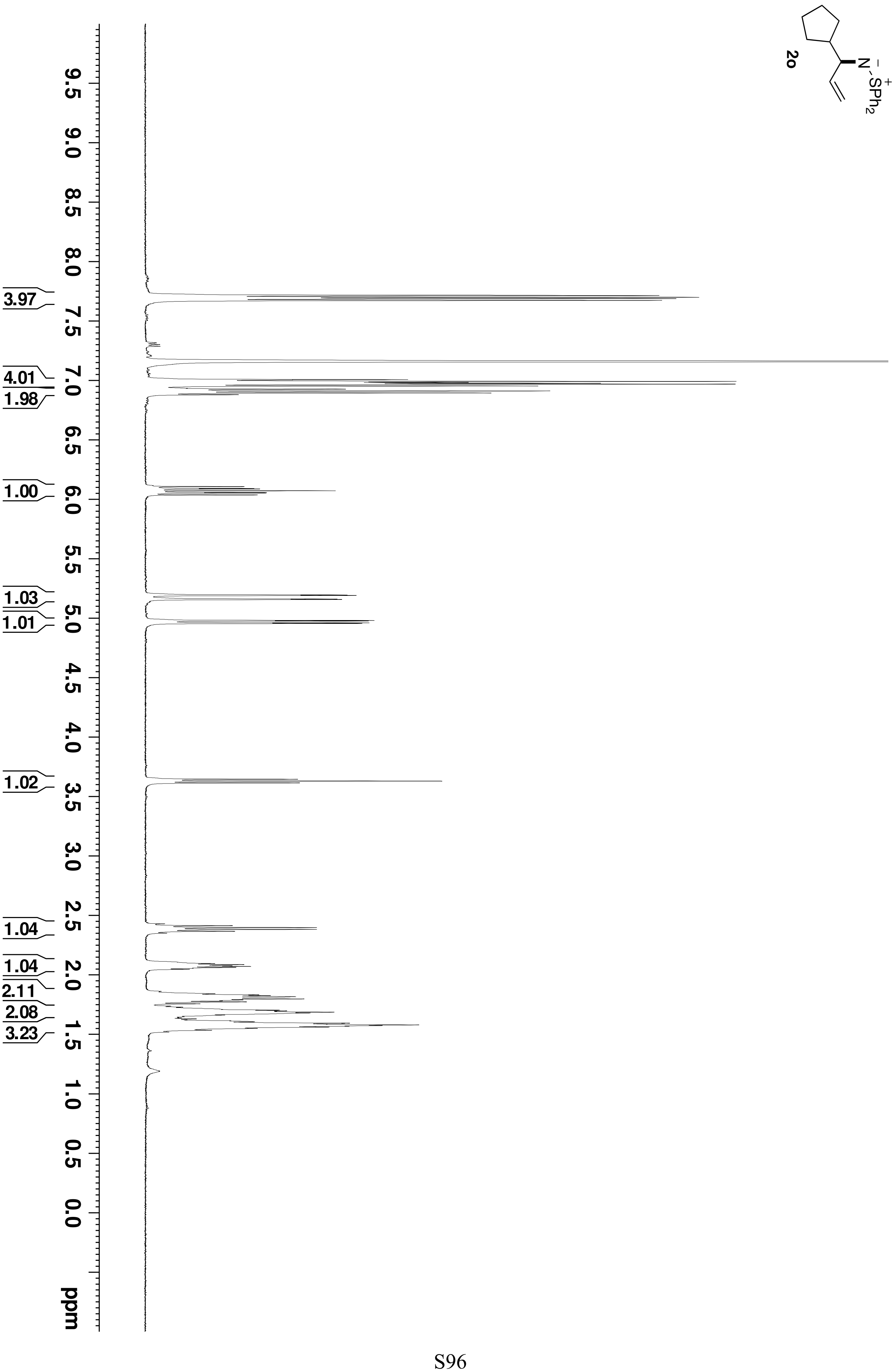
2o
N
SPh2
9.5
9.0
8.5
8.0
7.5
7.0
6.5
6.0
5.5
5.0
4.5
4.0
3.5
3.0
2.5
2.0
1.5
1.0
0.5
0.0
ppm
3.97
4.01
1.98
1.00
1.03
1.01
1.02
1.04
1.04
2.11
2.08
3.23

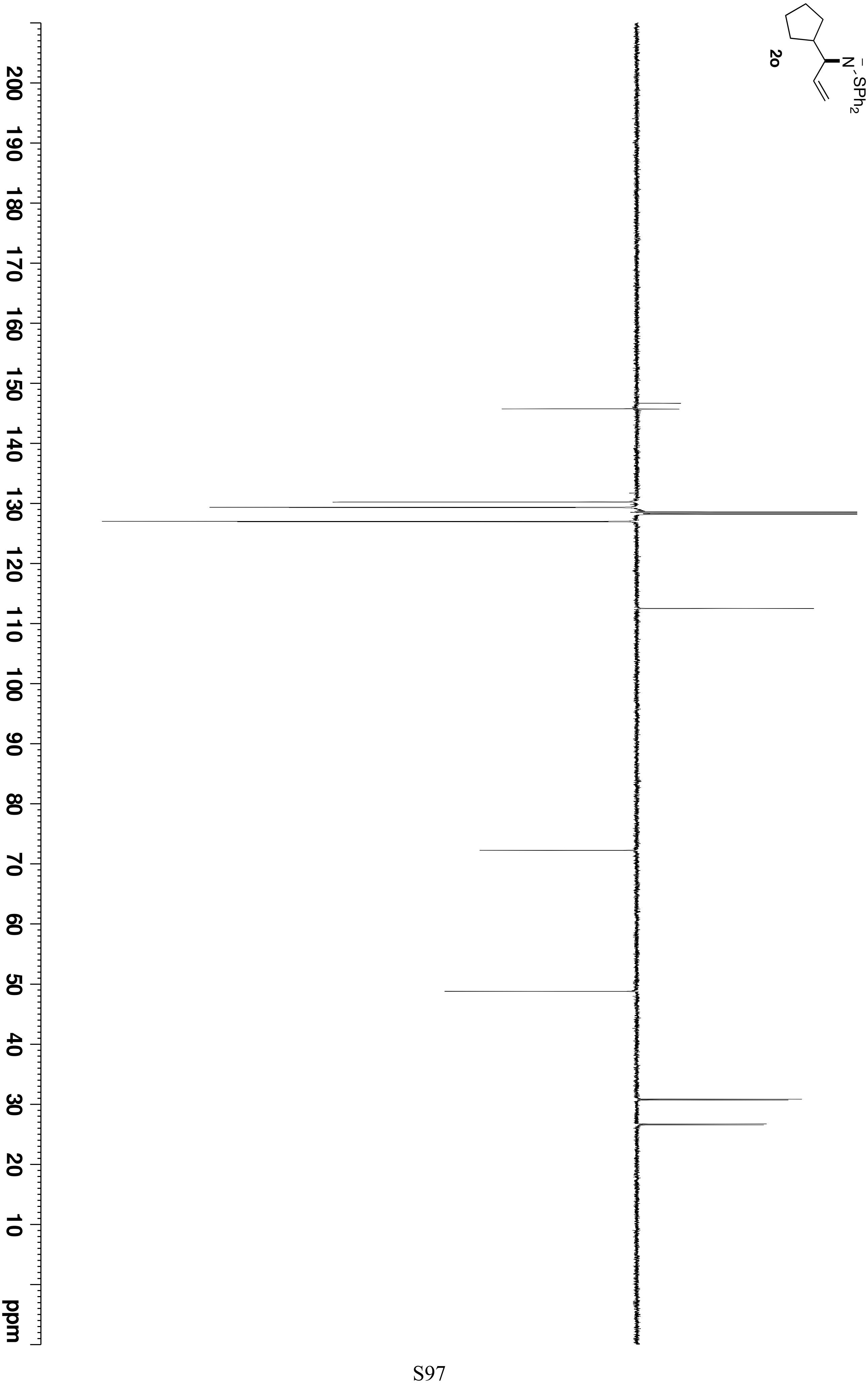
2o
200
190
180
170
160
150
140
130
120
110
100
90
80
70
60
50
40
30
20
10
ppm

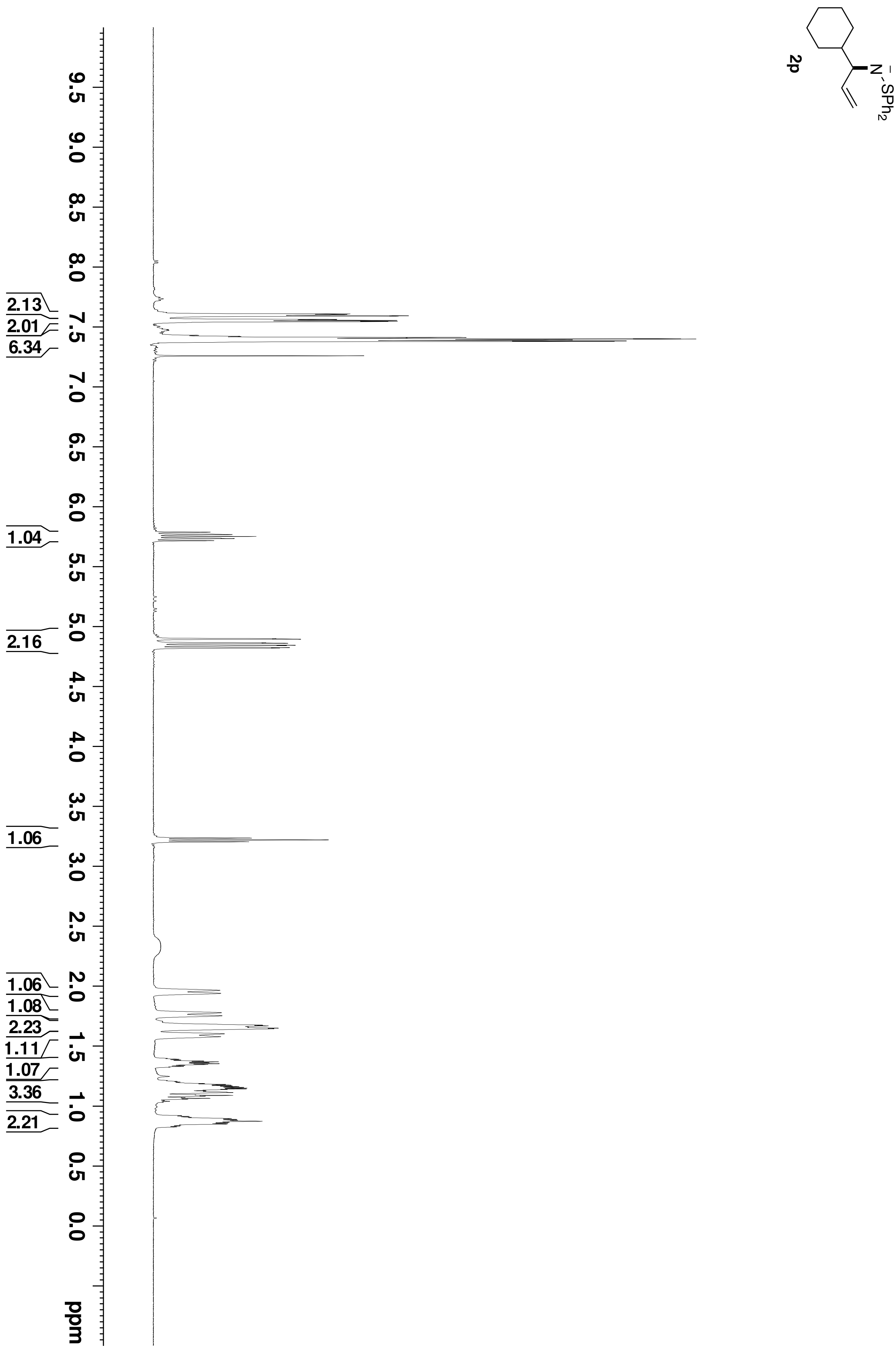
2p
N
SPh2
9.5
9.0
8.5
8.0
7.5
7.0
6.5
6.0
5.5
5.0
4.5
4.0
3.5
3.0
2.5
2.0
1.5
1.0
0.5
0.0
ppm
2.13
2.01
6.34
1.04
2.16
1.06
1.06
1.08
2.23
1.11
1.07
3.36
2.21

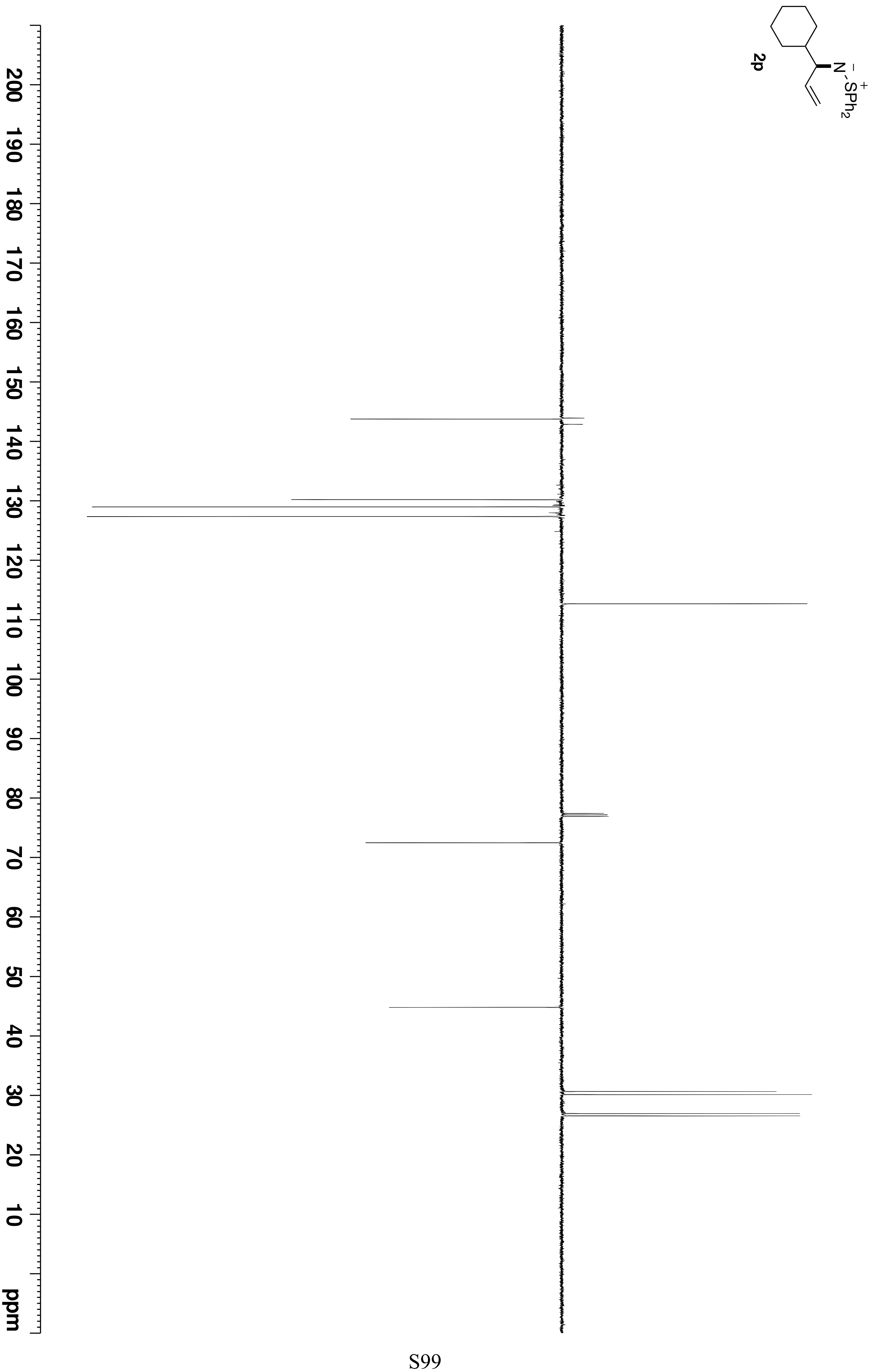

200
190
180
170
160
150
140
130
120
110
100
90
80
70
60
50
40
30
20
10
ppm
N
SPh2

2p

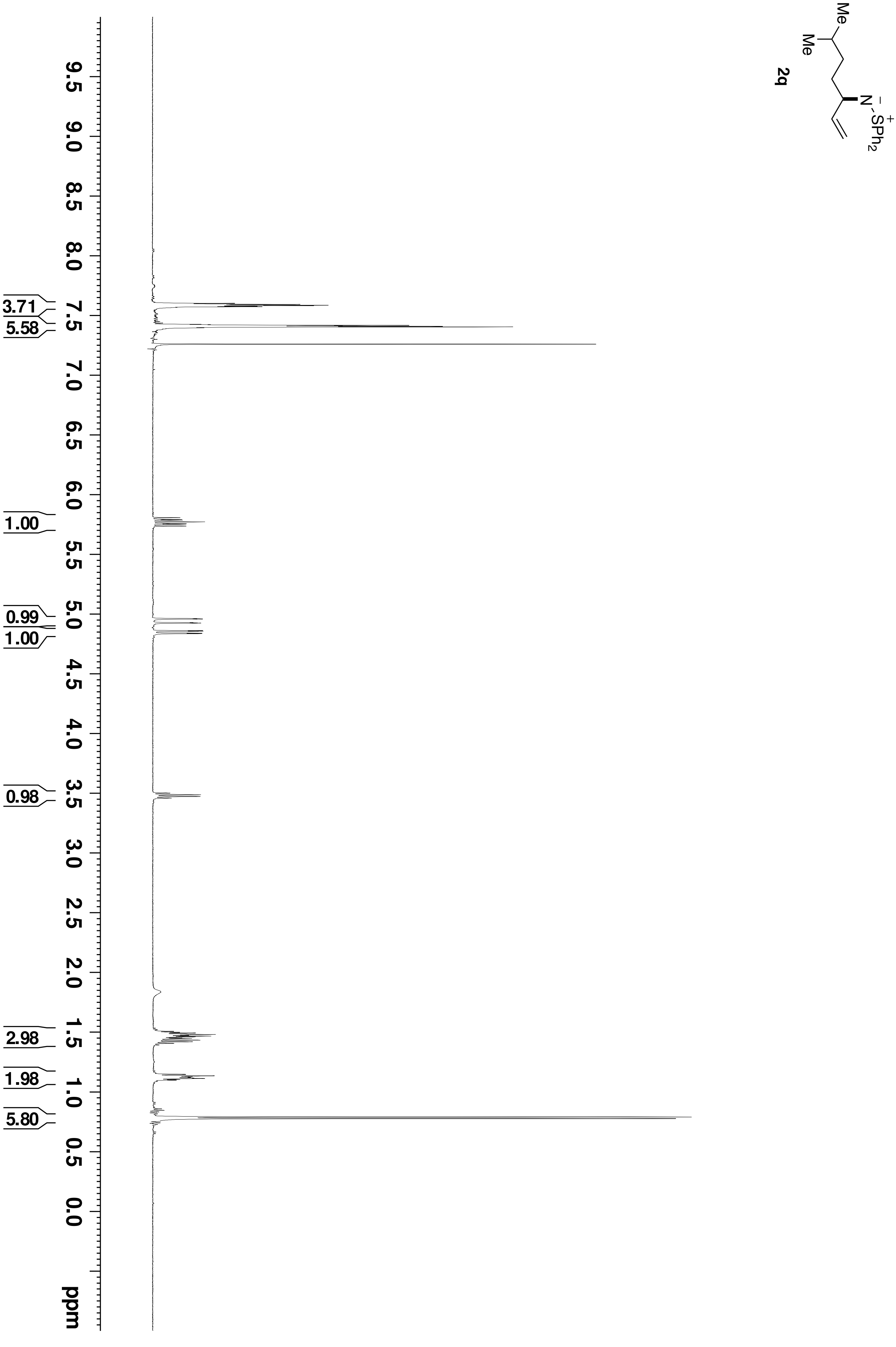
2q
Me
Me
N
SPh2
ppm
9.5
9.0
8.5
8.0
7.5
7.0
6.5
6.0
5.5
5.0
4.5
4.0
3.5
3.0
2.5
2.0
1.5
1.0
0.5
0.0
3.71
5.58
1.00
0.99
1.00
0.98
2.98
1.98
5.80

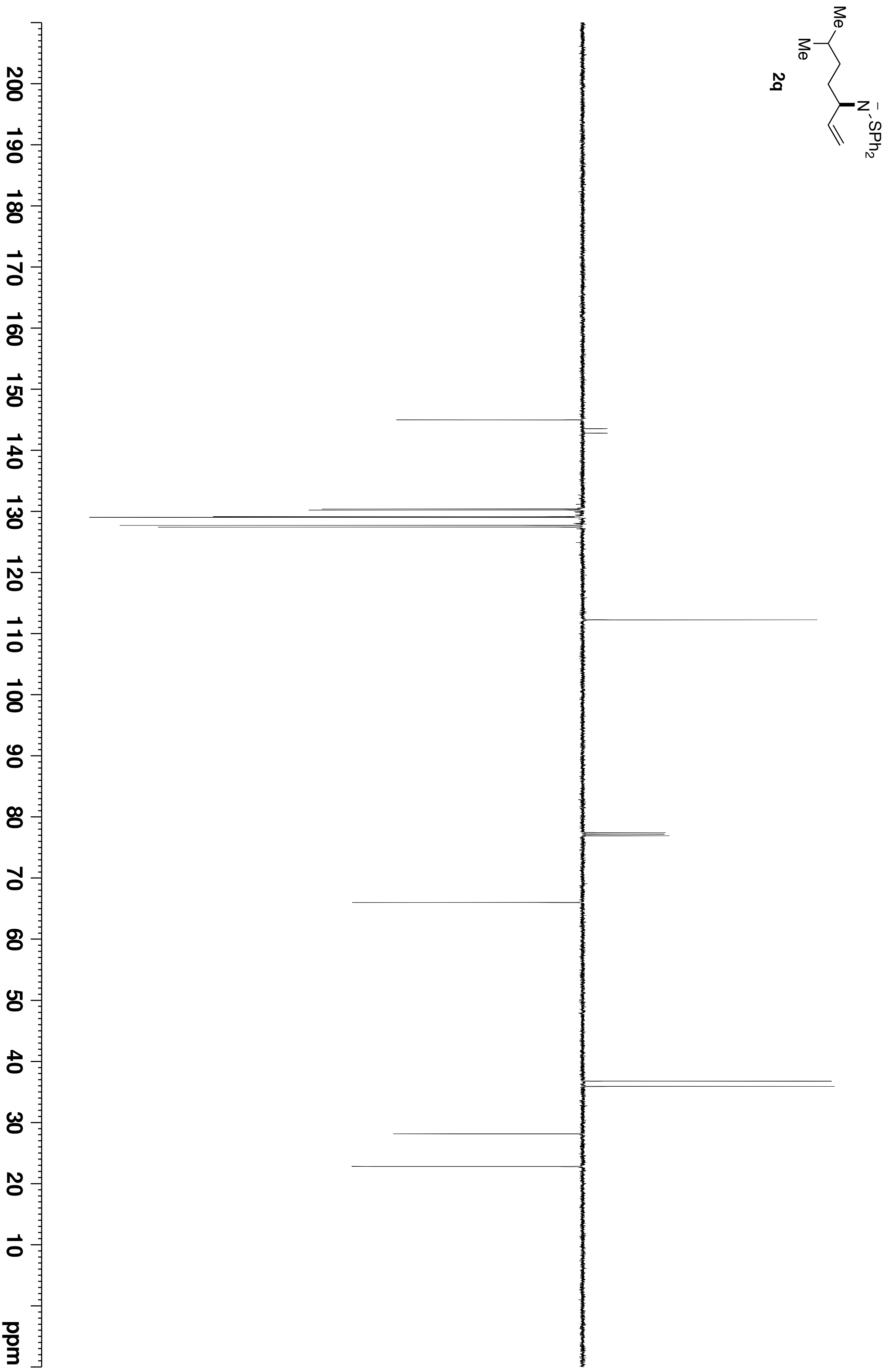

Me
Me
N
SPh2
2q
200
190
180
170
160
150
140
130
120
110
100
90
80
70
60
50
40
30
20
10
ppm

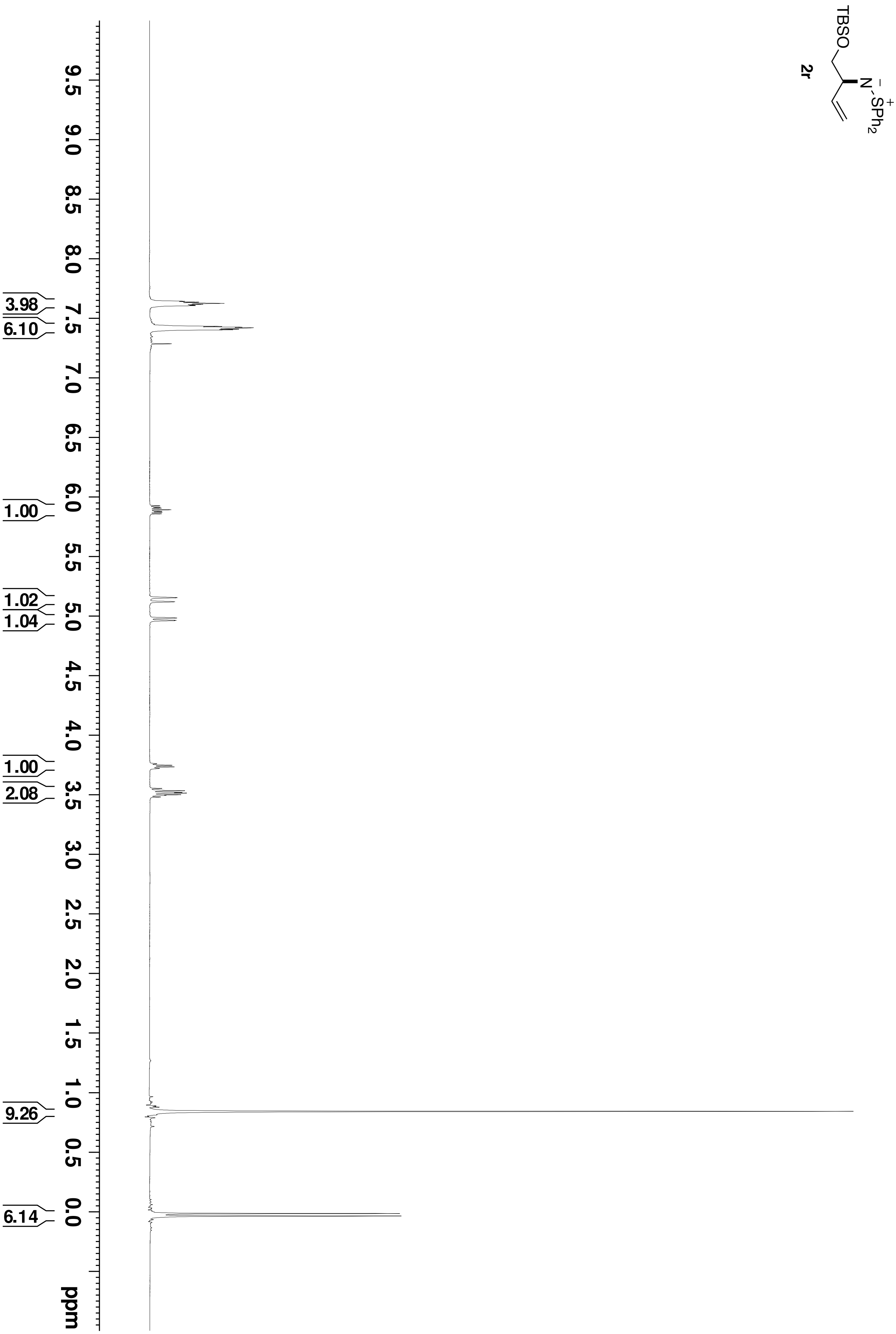
TBSO
N
SPh2
2r
9.5
9.0
8.5
8.0
7.5
7.0
6.5
6.0
5.5
5.0
4.5
4.0
3.5
3.0
2.5
2.0
1.5
1.0
0.5
0.0
ppm
3.98
6.10
1.00
1.02
1.04
1.00
2.08
9.26
6.14

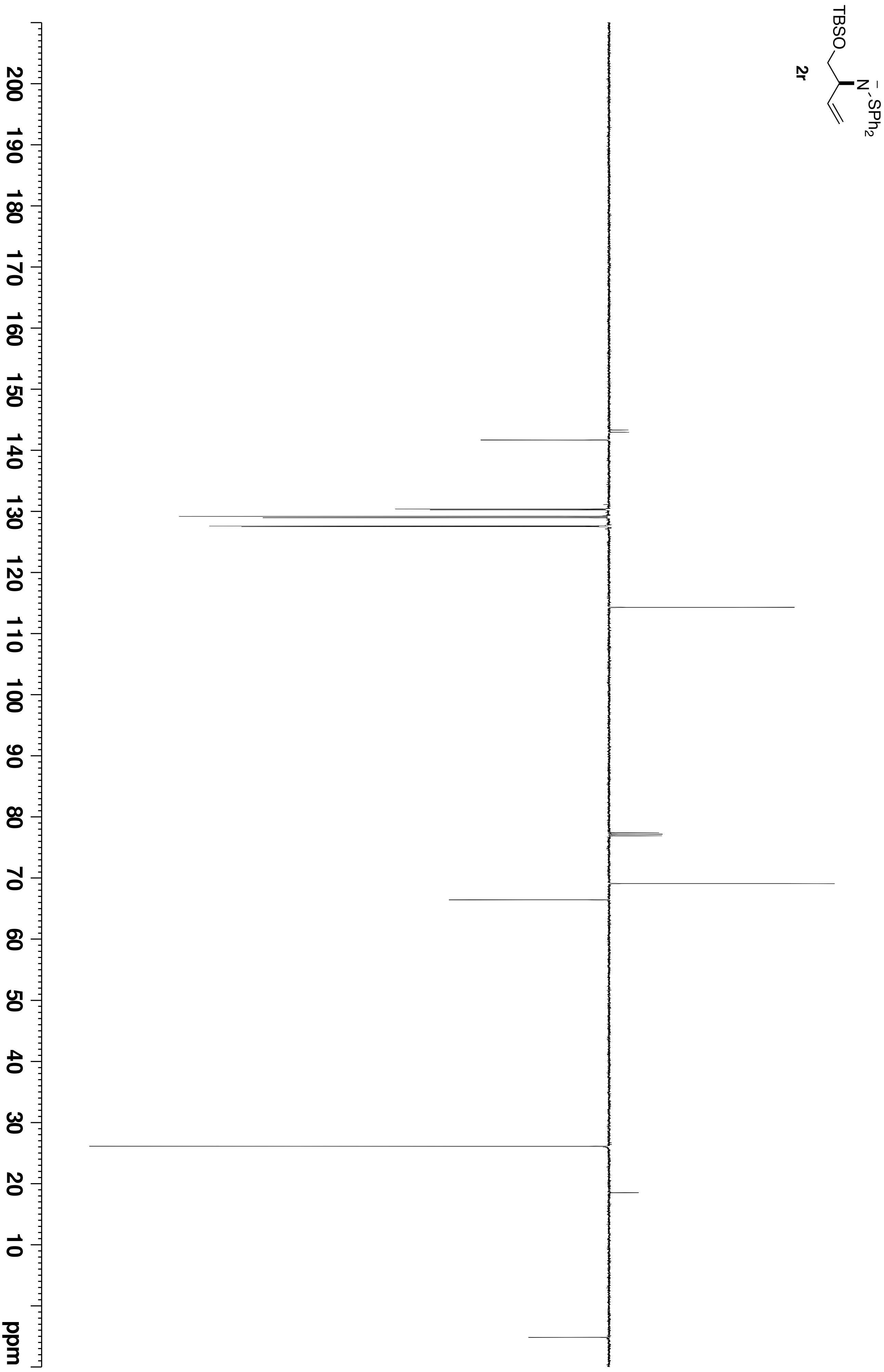
TBSO
N
SPh2
2r
200 190 180 170 160 150 140 130 120 110 100 90 80 70 60 50 40 30 20 10
ppm

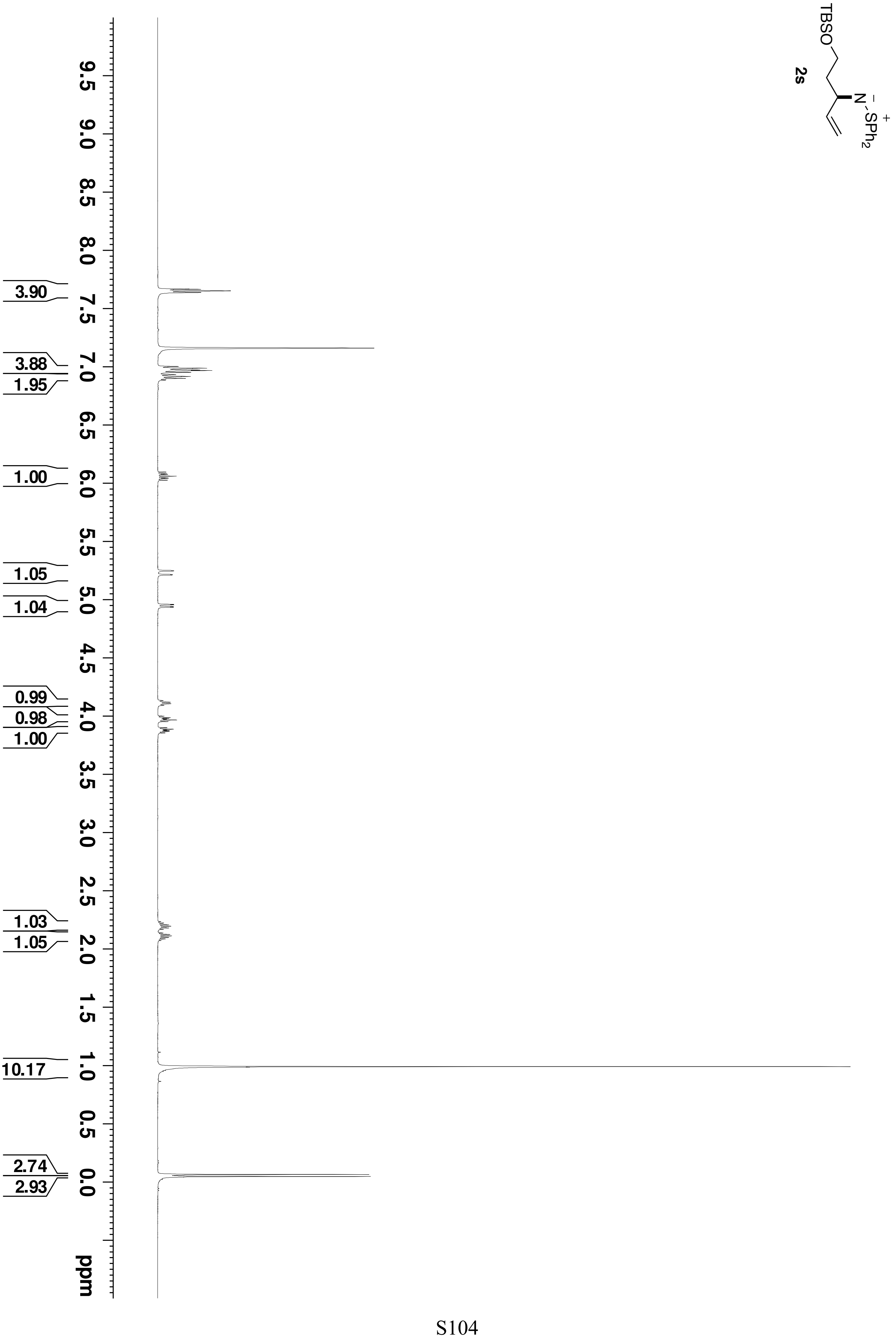
TBSO
N
SPh2
2s
9.5
9.0
8.5
8.0
7.5
7.0
6.5
6.0
5.5
5.0
4.5
4.0
3.5
3.0
2.5
2.0
1.5
1.0
0.5
0.0
ppm
3.90
3.88
1.95
1.00
1.05
1.04
0.99
0.98
1.00
1.03
1.05
10.17
2.74
2.93

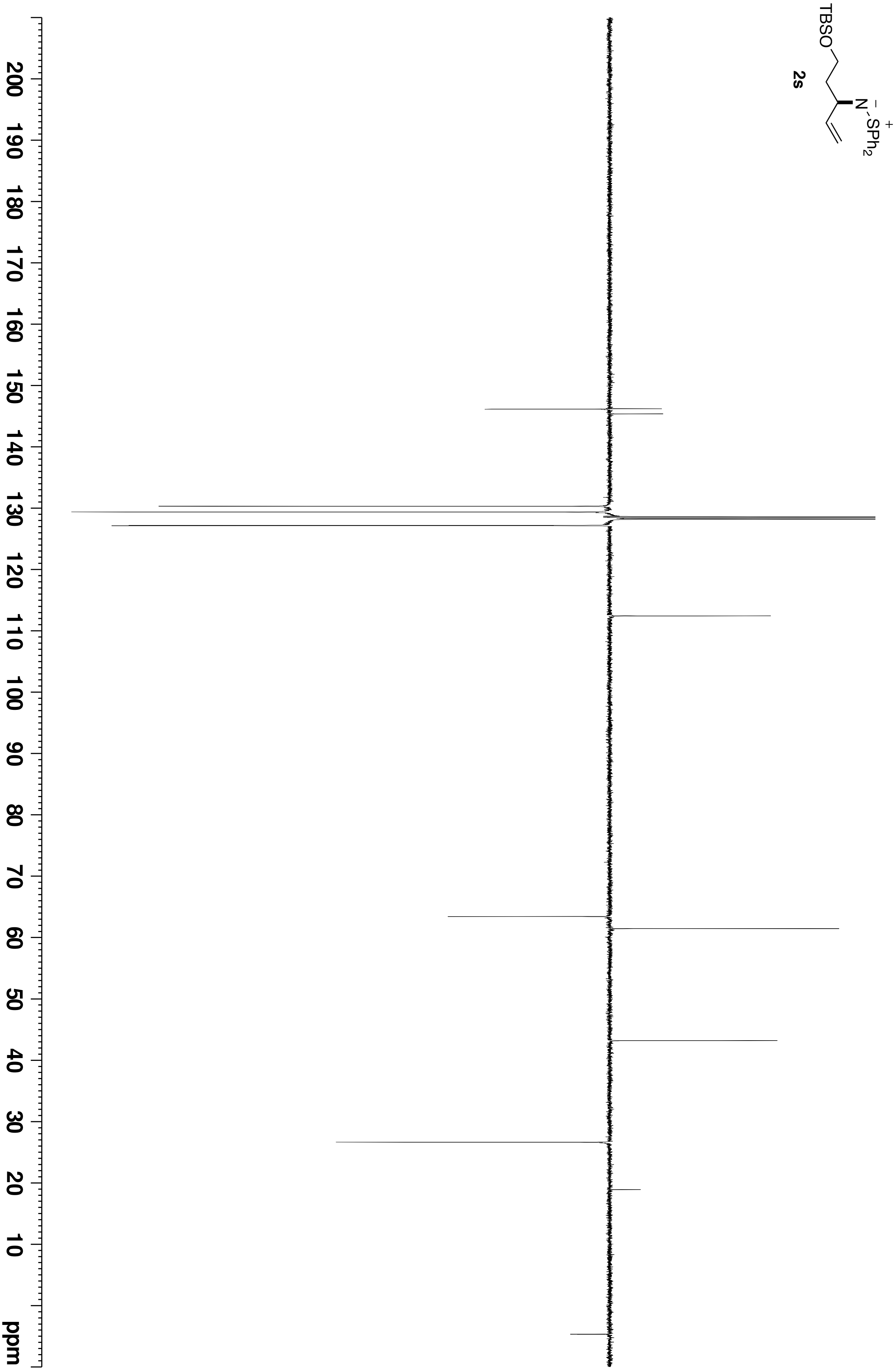
TBSO
N
SPh2
2s
200
190
180
170
160
150
140
130
120
110
100
90
80
70
60
50
40
30
20
10
ppm

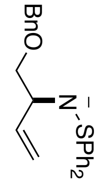
BnO
N
SPh2

2t

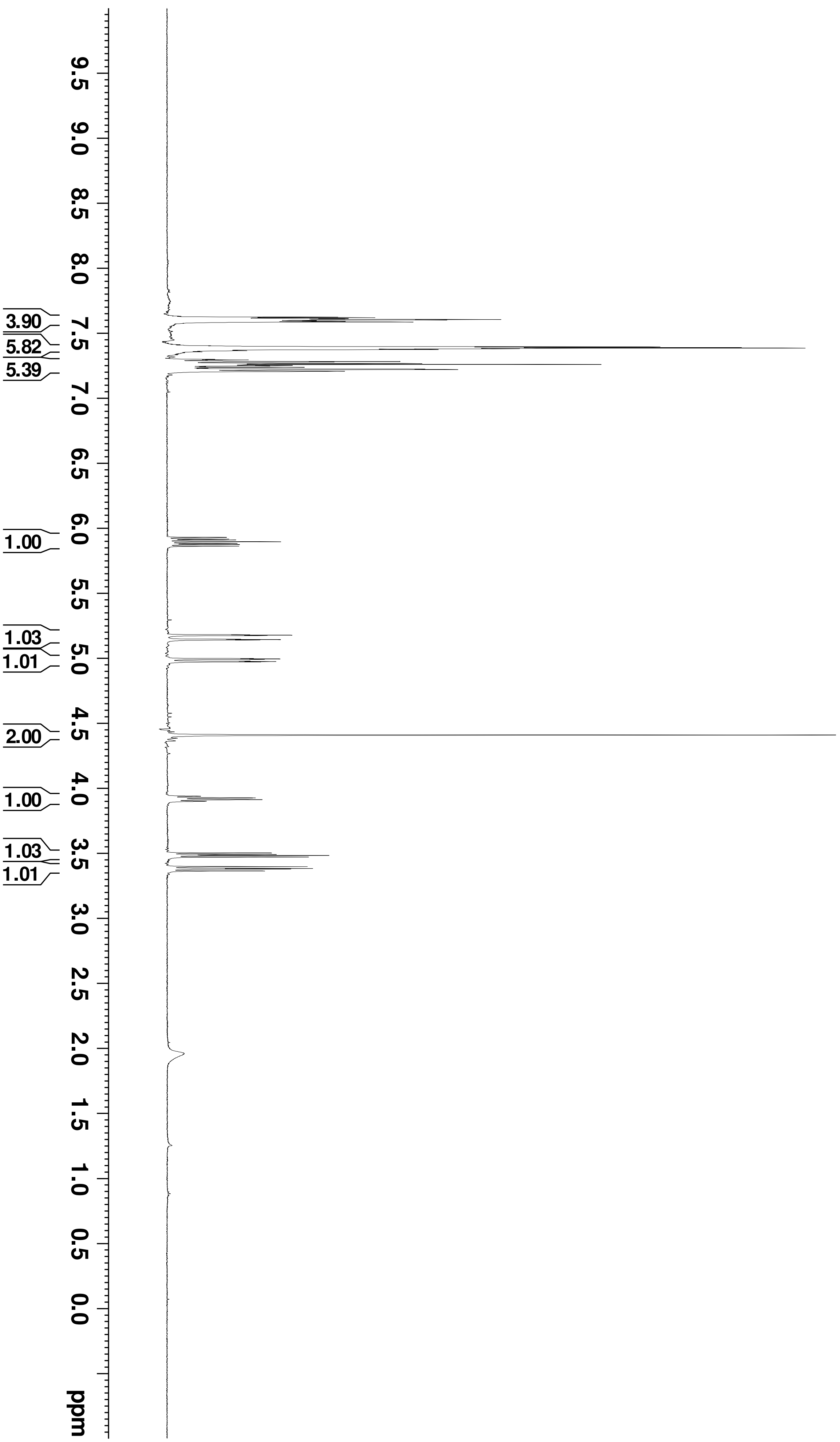
9.5
9.0
8.5
8.0
7.5
7.0
6.5
6.0
5.5
5.0
4.5
4.0
3.5
3.0
2.5
2.0
1.5
1.0
0.5
0.0
ppm
3.90
5.82
5.39
1.00
1.03
1.01
2.00
1.00
1.03
1.01

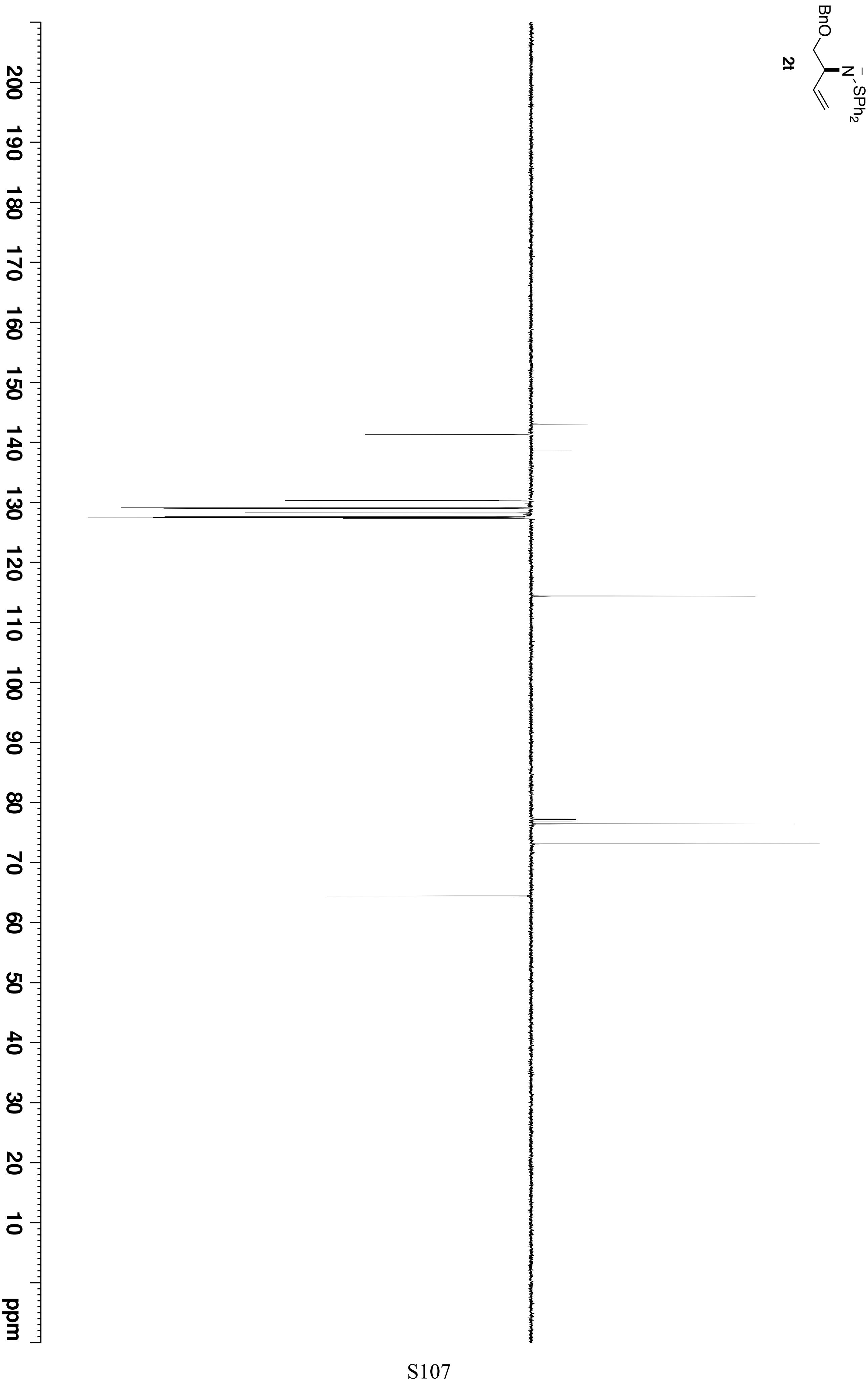
BnO
N
SPh2
2t
200
190
180
170
160
150
140
130
120
110
100
90
80
70
60
50
40
30
20
10
ppm

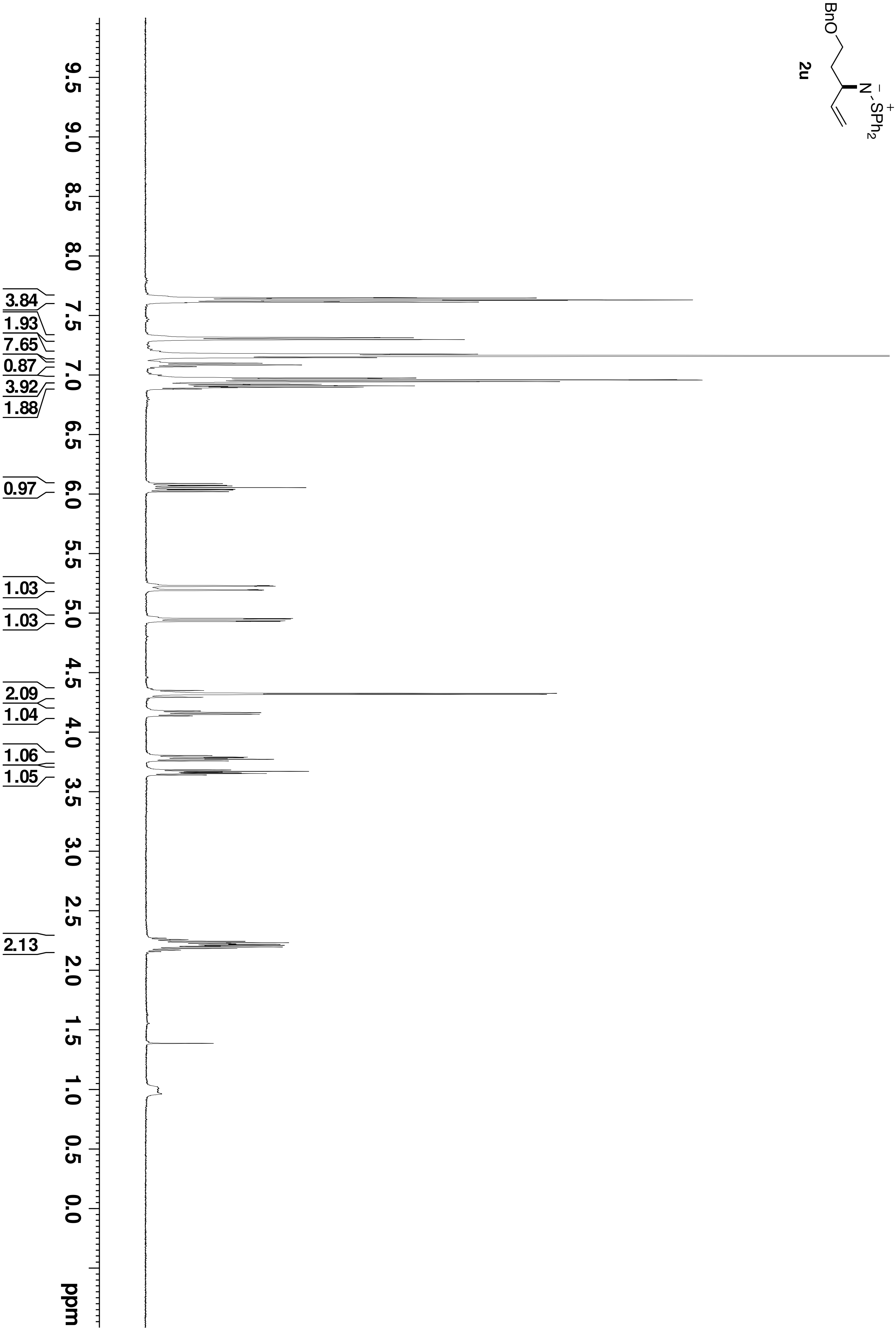

BnO
2u
N
SPh2
3.84
1.93
7.65
0.87
3.92
1.88
0.97
1.03
1.03
2.09
1.04
1.06
1.05
2.13
9.5
9.0
8.5
8.0
7.5
7.0
6.5
6.0
5.5
5.0
4.5
4.0
3.5
3.0
2.5
2.0
1.5
1.0
0.5
0.0
ppm

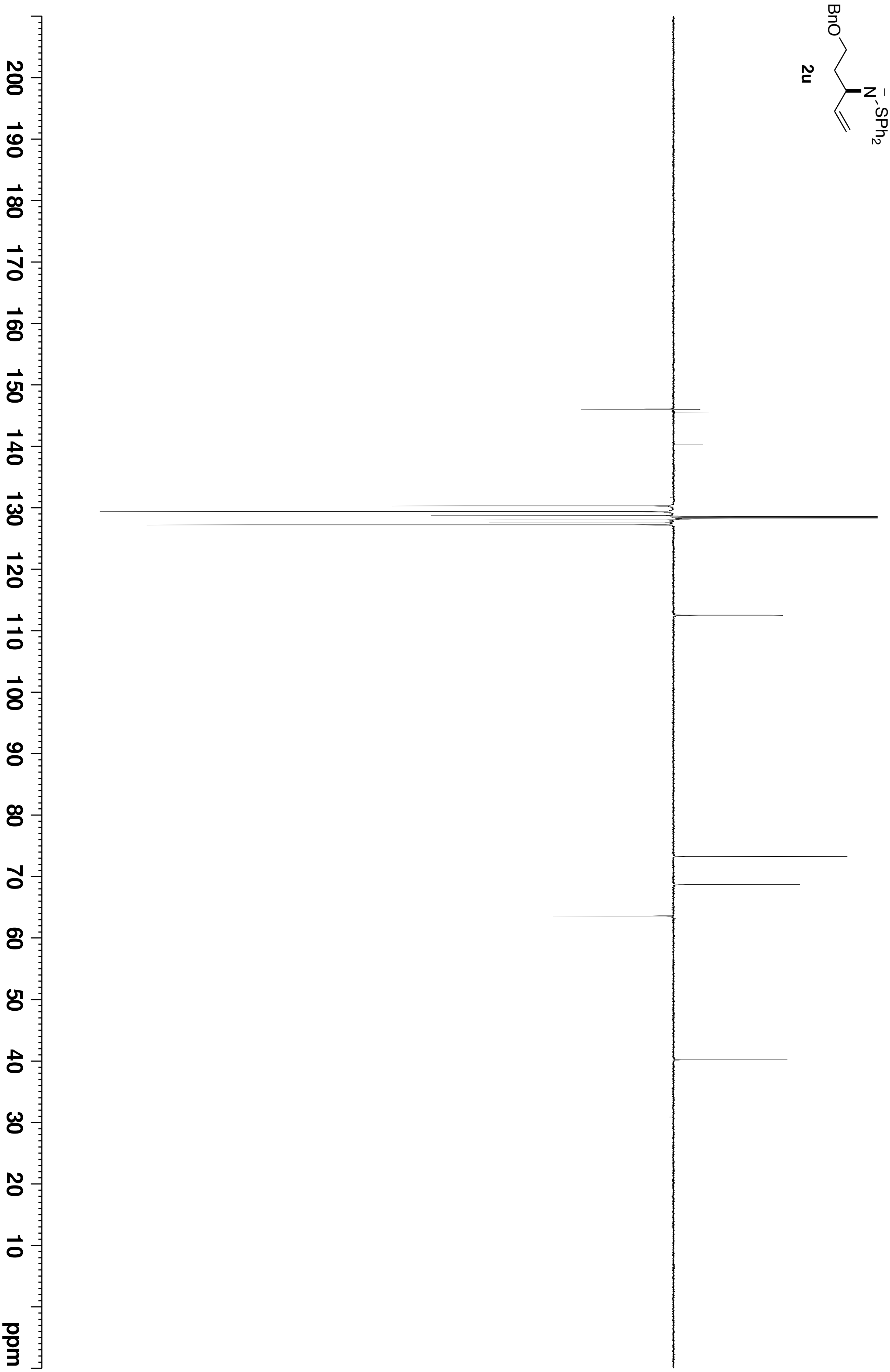

BnO
N
SPh2
2u
200
190
180
170
160
150
140
130
120
110
100
90
80
70
60
50
40
30
20
10
ppm

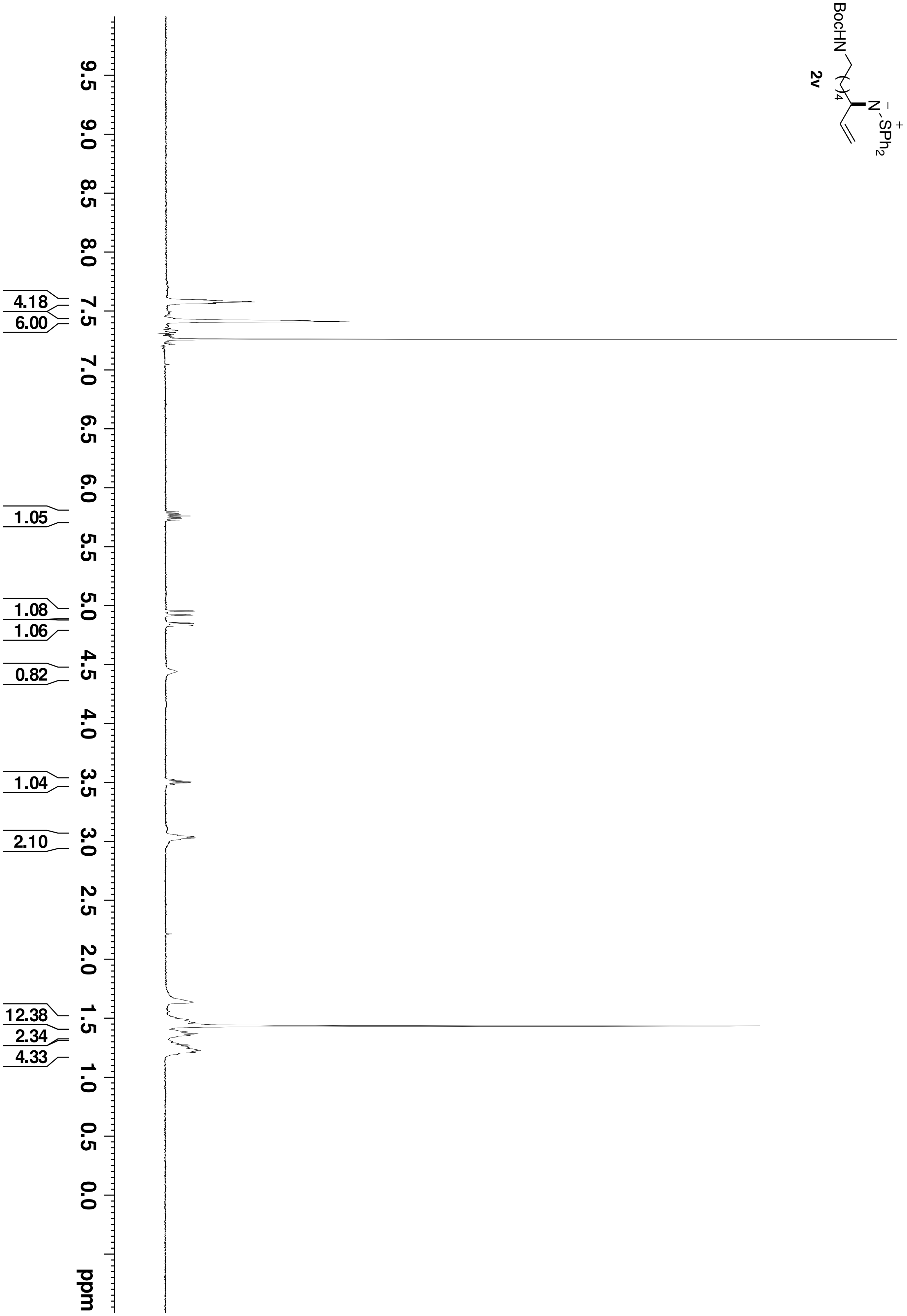
BocHN
N
SPh2
4
2v
9.5
9.0
8.5
8.0
7.5
7.0
6.5
6.0
5.5
5.0
4.5
4.0
3.5
3.0
2.5
2.0
1.5
1.0
0.5
0.0
ppm
4.18
6.00
1.05
1.08
1.06
0.82
1.04
2.10
12.38
2.34
4.33

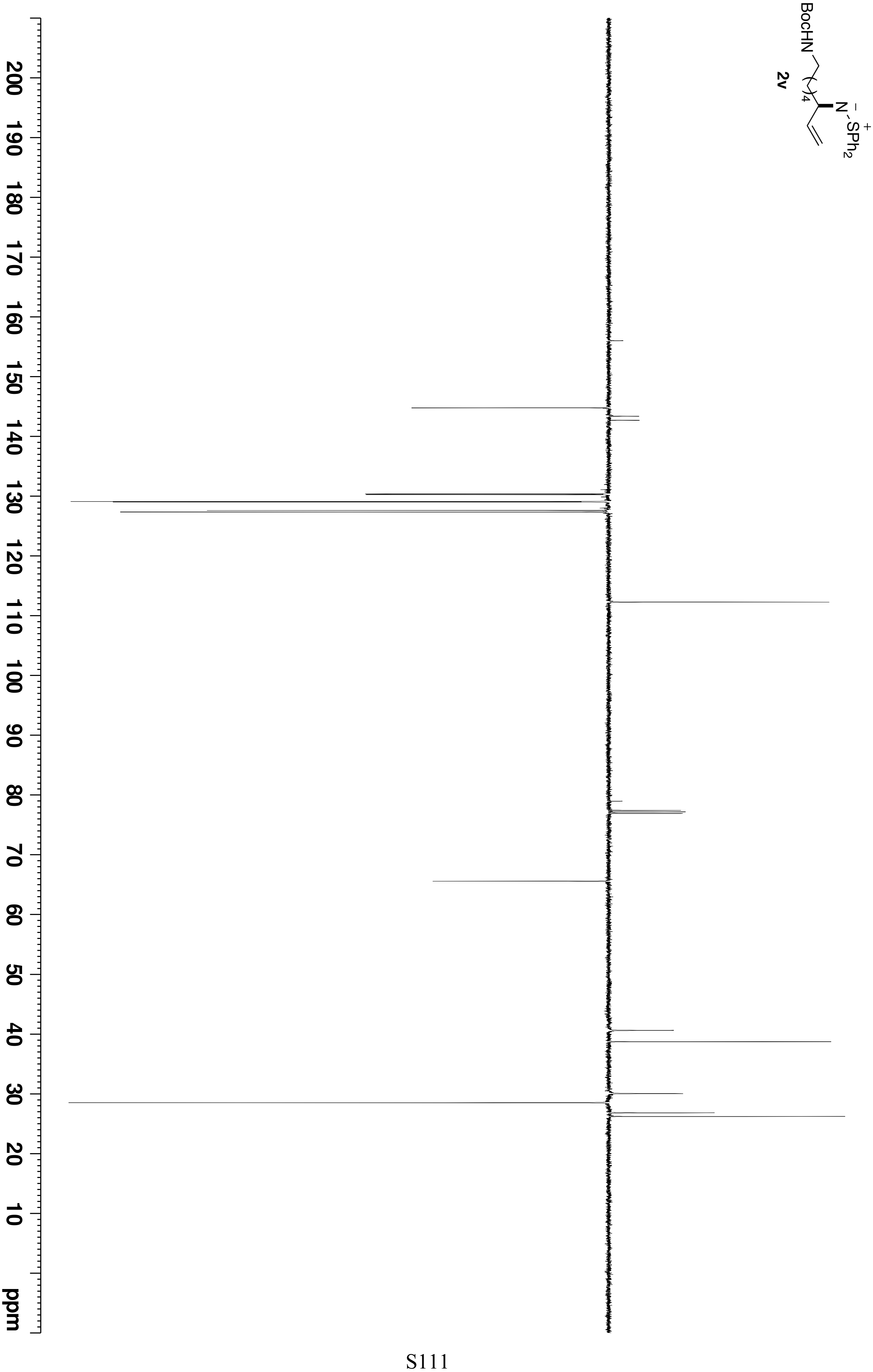
BocHN
N
SPh2
4
2v
200 190 180 170 160 150 140 130 120 110 100 90 80 70 60 50 40 30 20 10 ppm

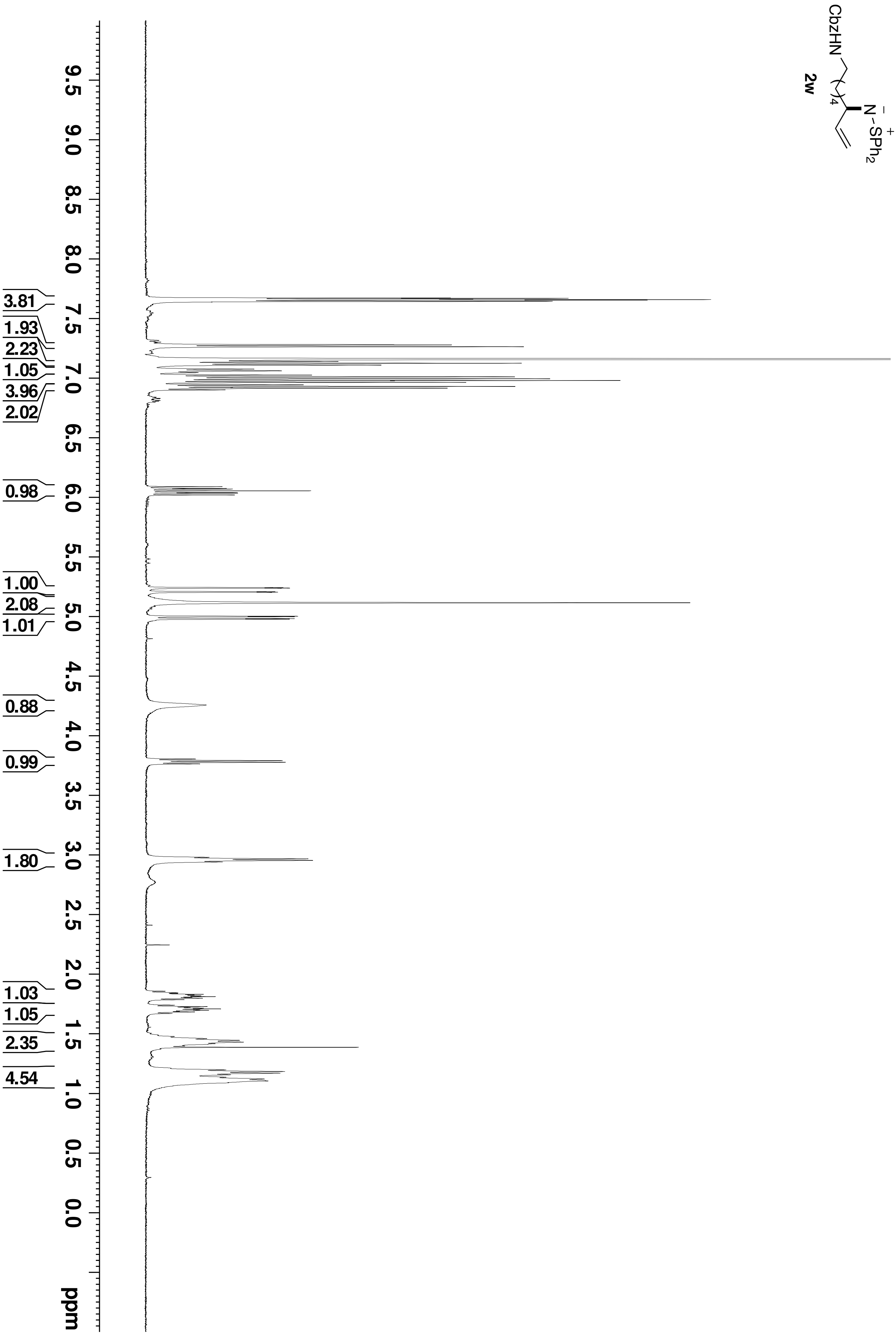

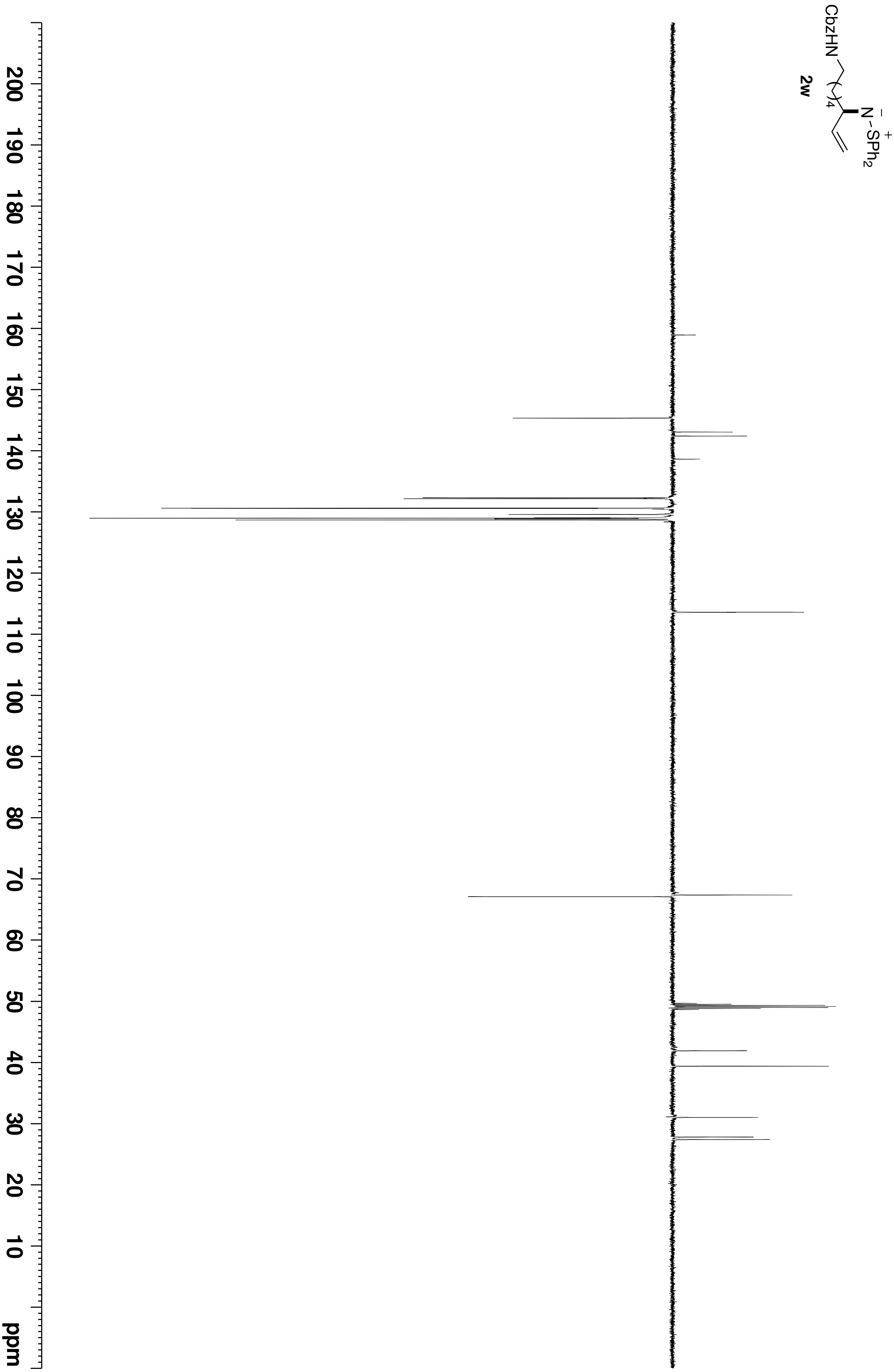

CbzHN
N-SPh2
4
2w
200
190
180
170
160
150
140
130
120
110
100
90
80
70
60
50
40
30
20
10
ppm

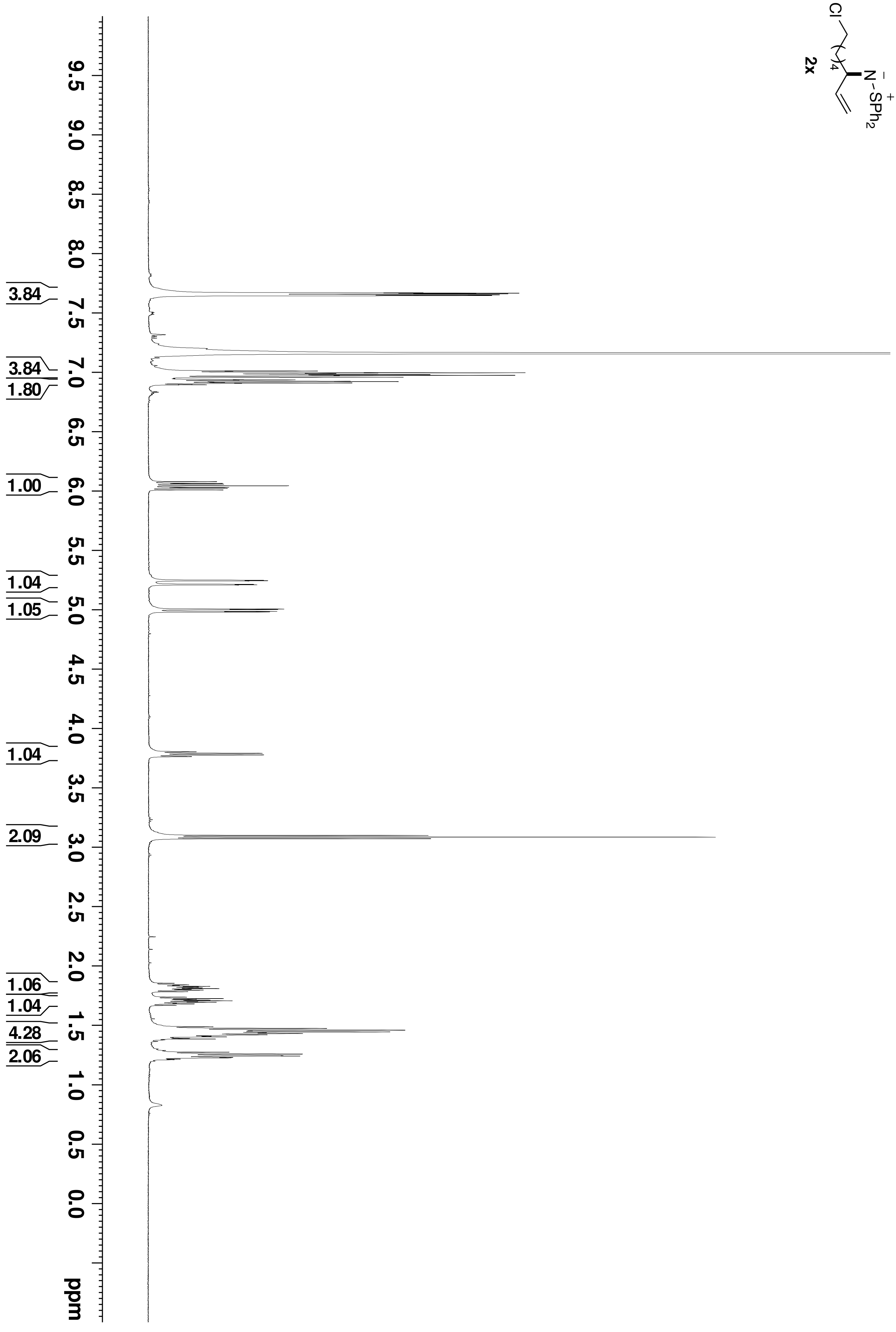

Cl
4
N
SPh2
2x
9.5
9.0
8.5
8.0
7.5
7.0
6.5
6.0
5.5
5.0
4.5
4.0
3.5
3.0
2.5
2.0
1.5
1.0
0.5
0.0
ppm
3.84
3.84
1.80
1.00
1.04
1.05
1.04
2.09
1.06
1.04
4.28
2.06

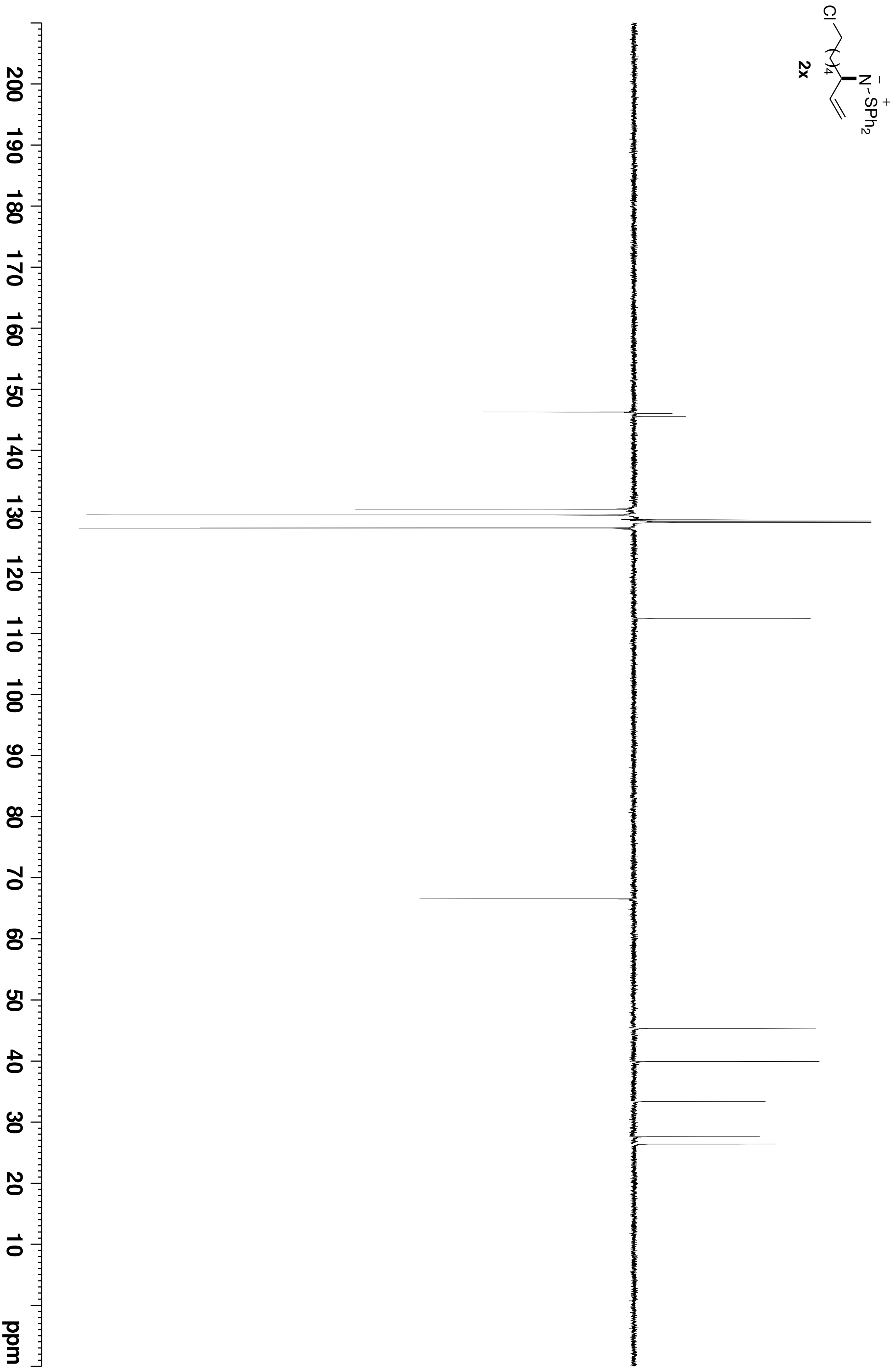
Cl
N
SPh2
4
2x
200
190
180
170
160
150
140
130
120
110
100
90
80
70
60
50
40
30
20
10
ppm

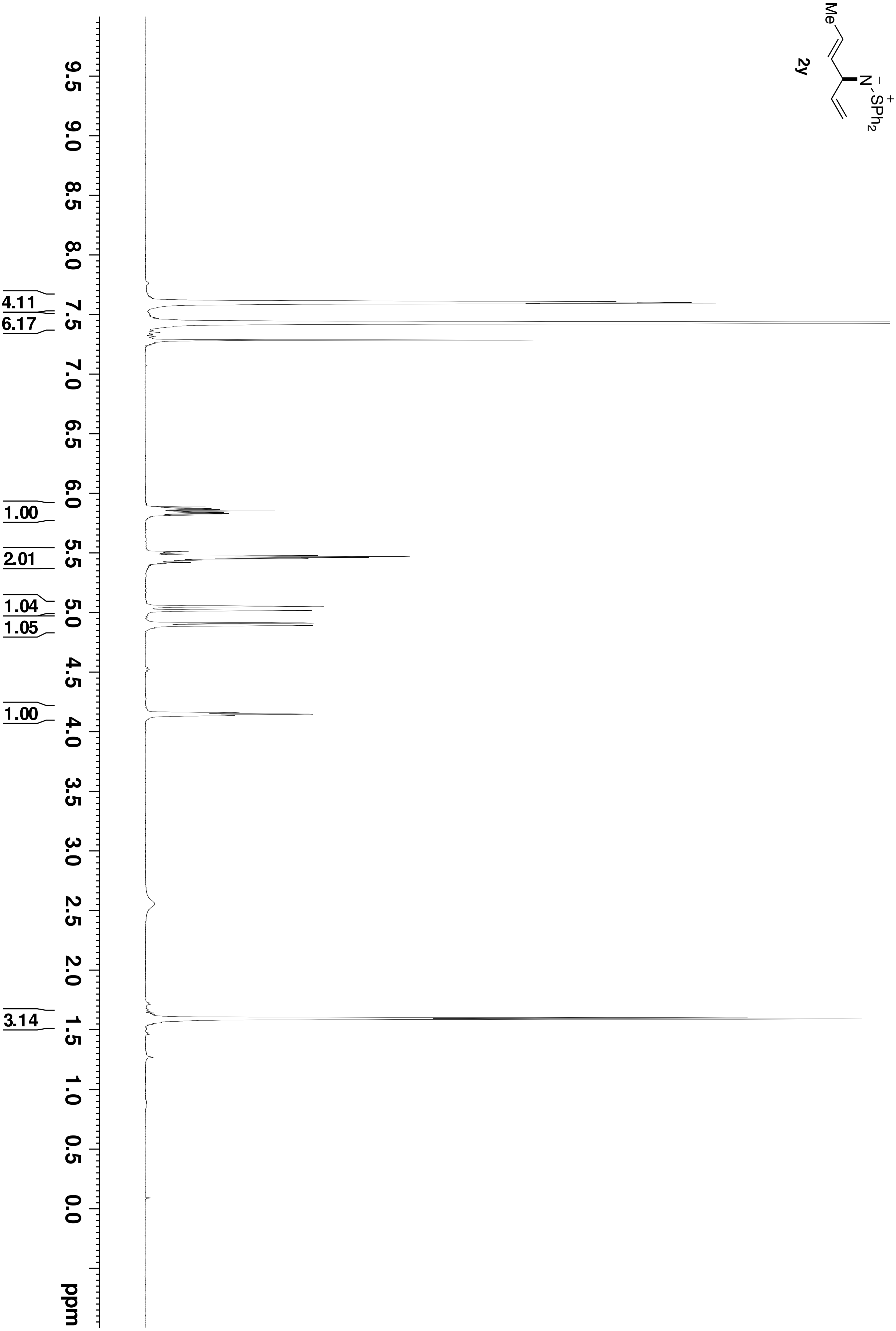
Me
N
SPh2
2y
4.11
6.17
1.00
2.01
1.04
1.05
1.00
3.14
9.5
9.0
8.5
8.0
7.5
7.0
6.5
6.0
5.5
5.0
4.5
4.0
3.5
3.0
2.5
2.0
1.5
1.0
0.5
0.0
ppm

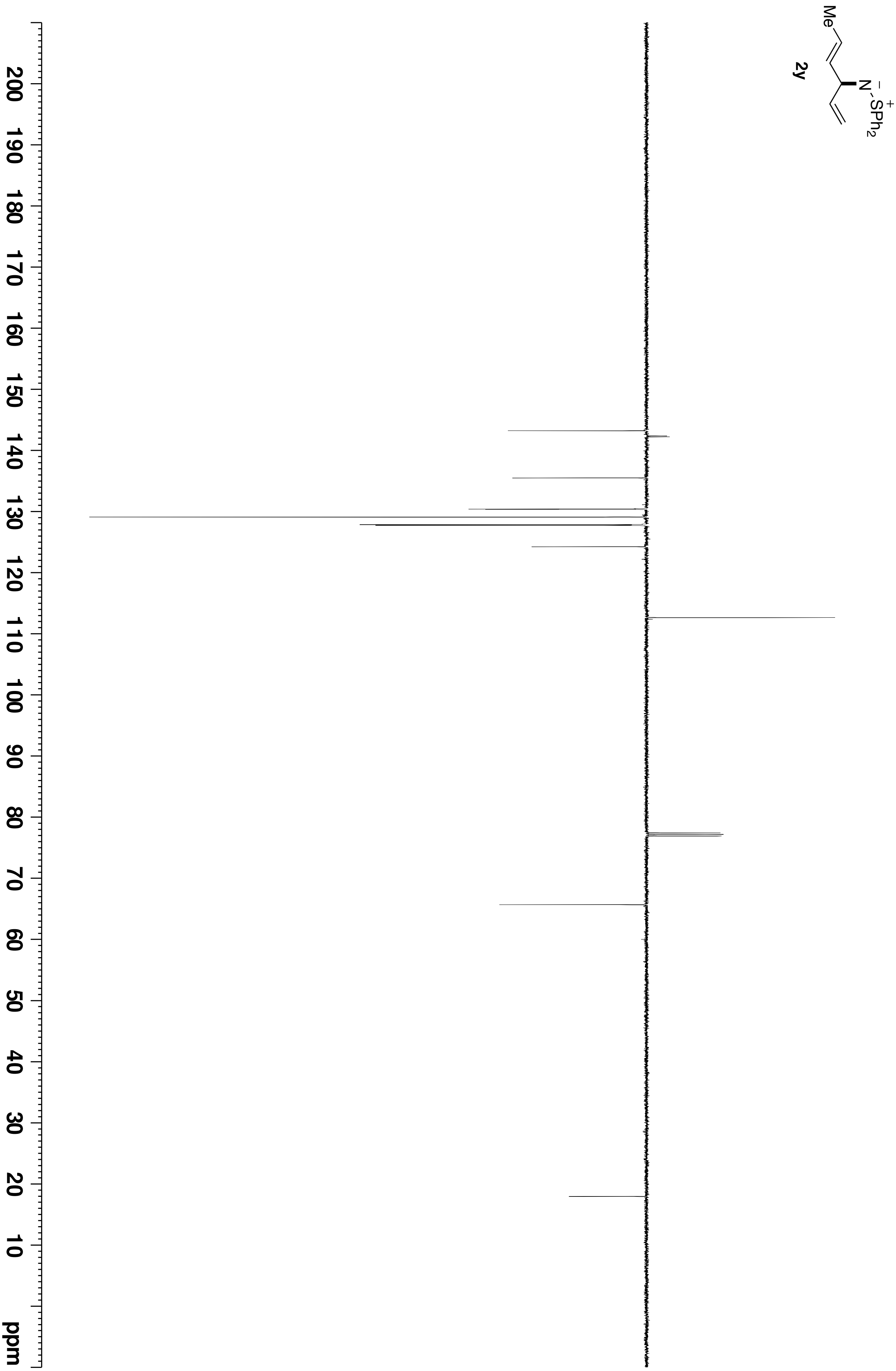
Me
N
SPh2
2y
200
190
180
170
160
150
140
130
120
110
100
90
80
70
60
50
40
30
20
10
ppm

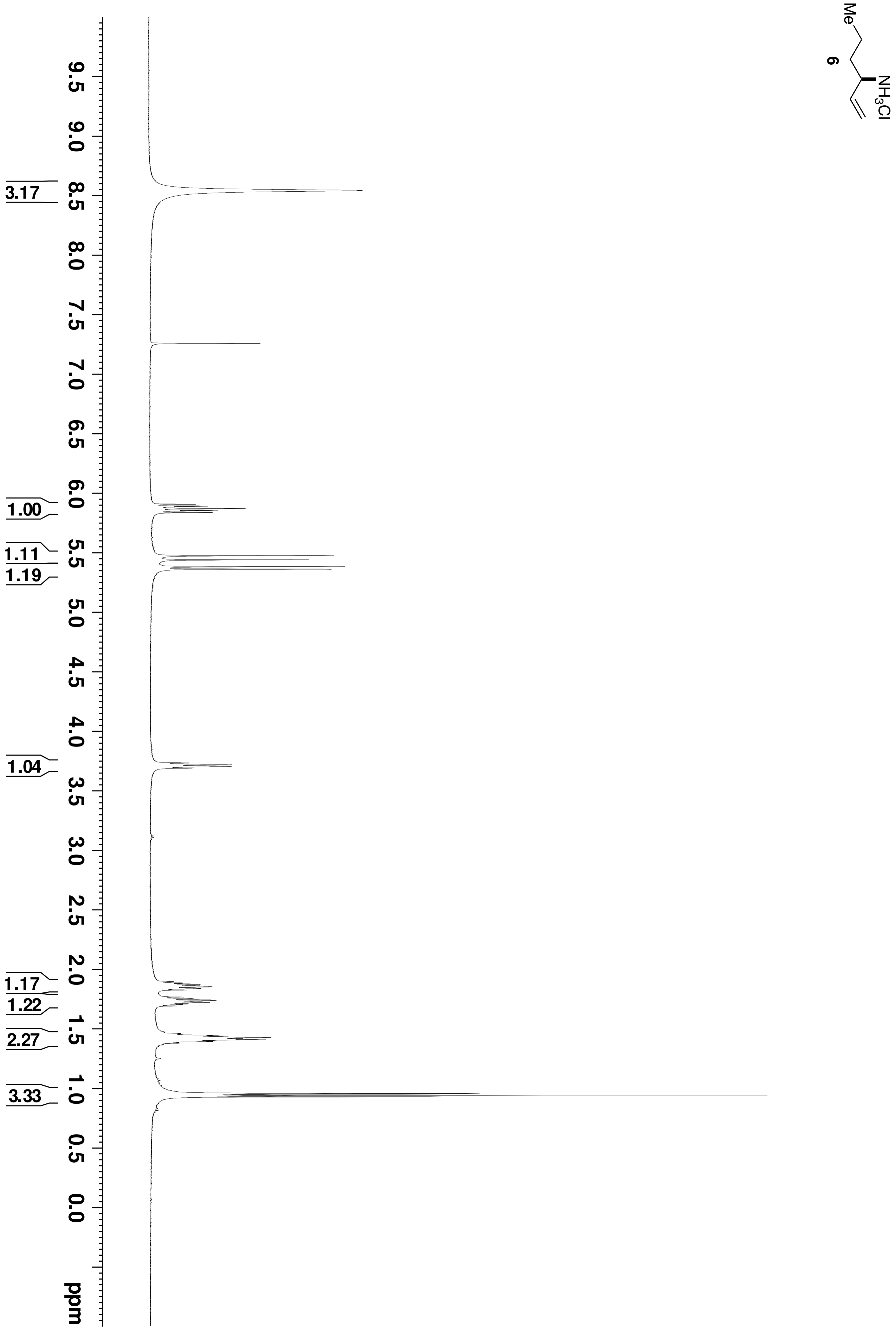

Me
NH3Cl
6
9.5
9.0
8.5
8.0
7.5
7.0
6.5
6.0
5.5
5.0
4.5
4.0
3.5
3.0
2.5
2.0
1.5
1.0
0.5
0.0
ppm
3.17
1.00
1.11
1.19
1.04
1.17
1.22
2.27
3.33

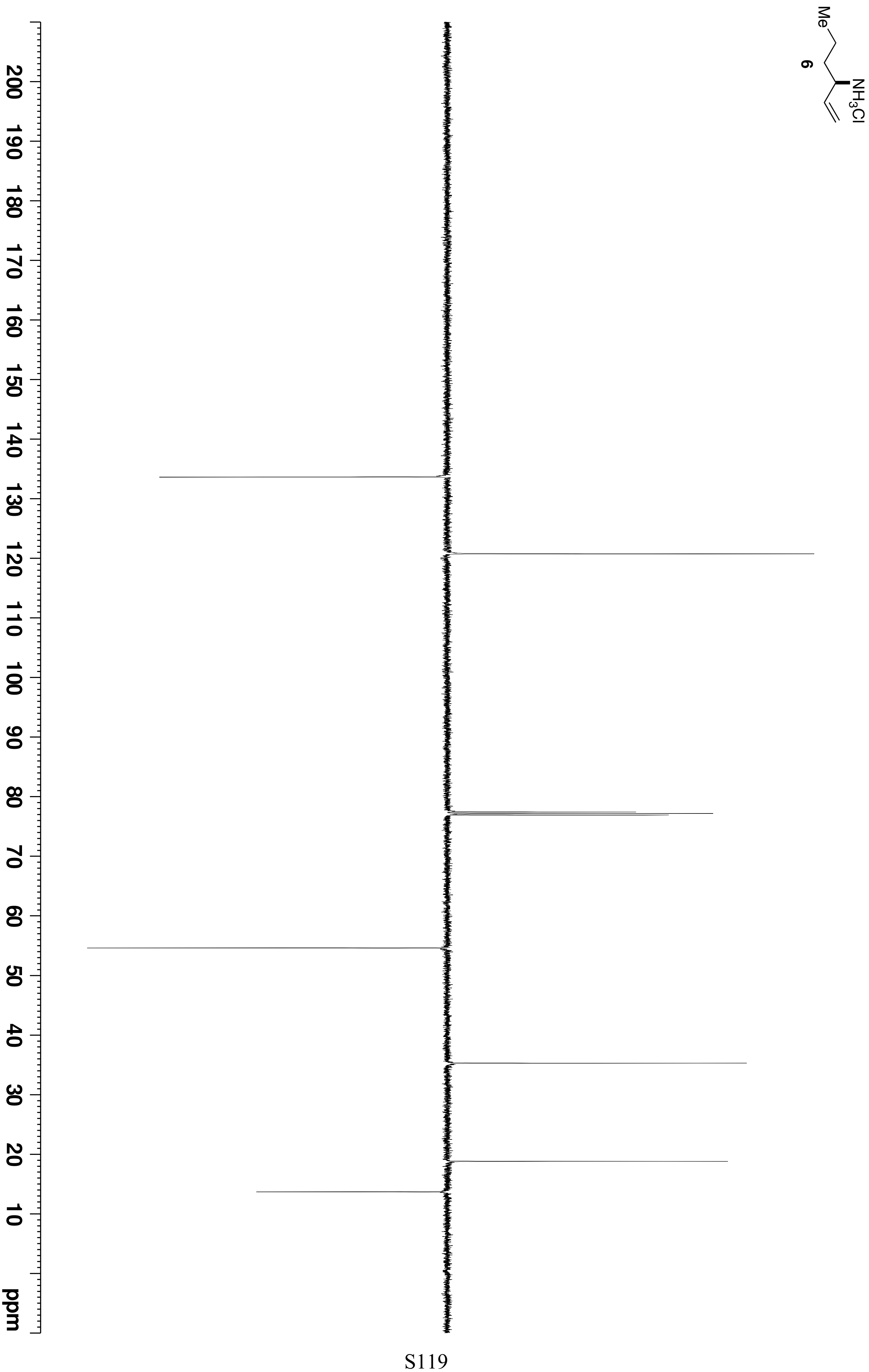

NH3Cl
Me
6
200 190 180 170 160 150 140 130 120 110 100 90 80 70 60 50 40 30 20 10 ppm

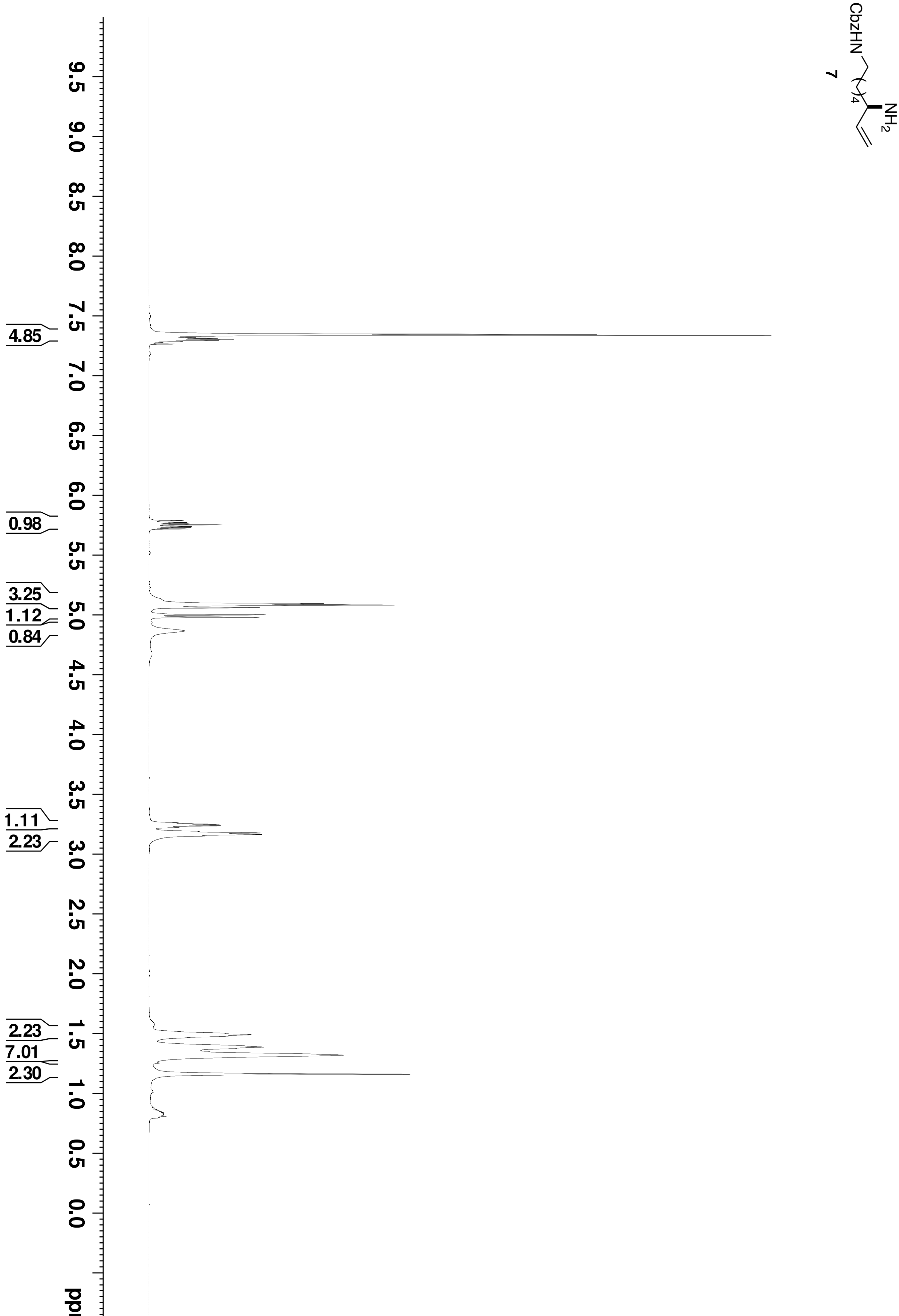
CbzHN
NH2
4
7
4.85
0.98
3.25
1.12
0.84
1.11
2.23
2.23
7.01
2.30
ppm

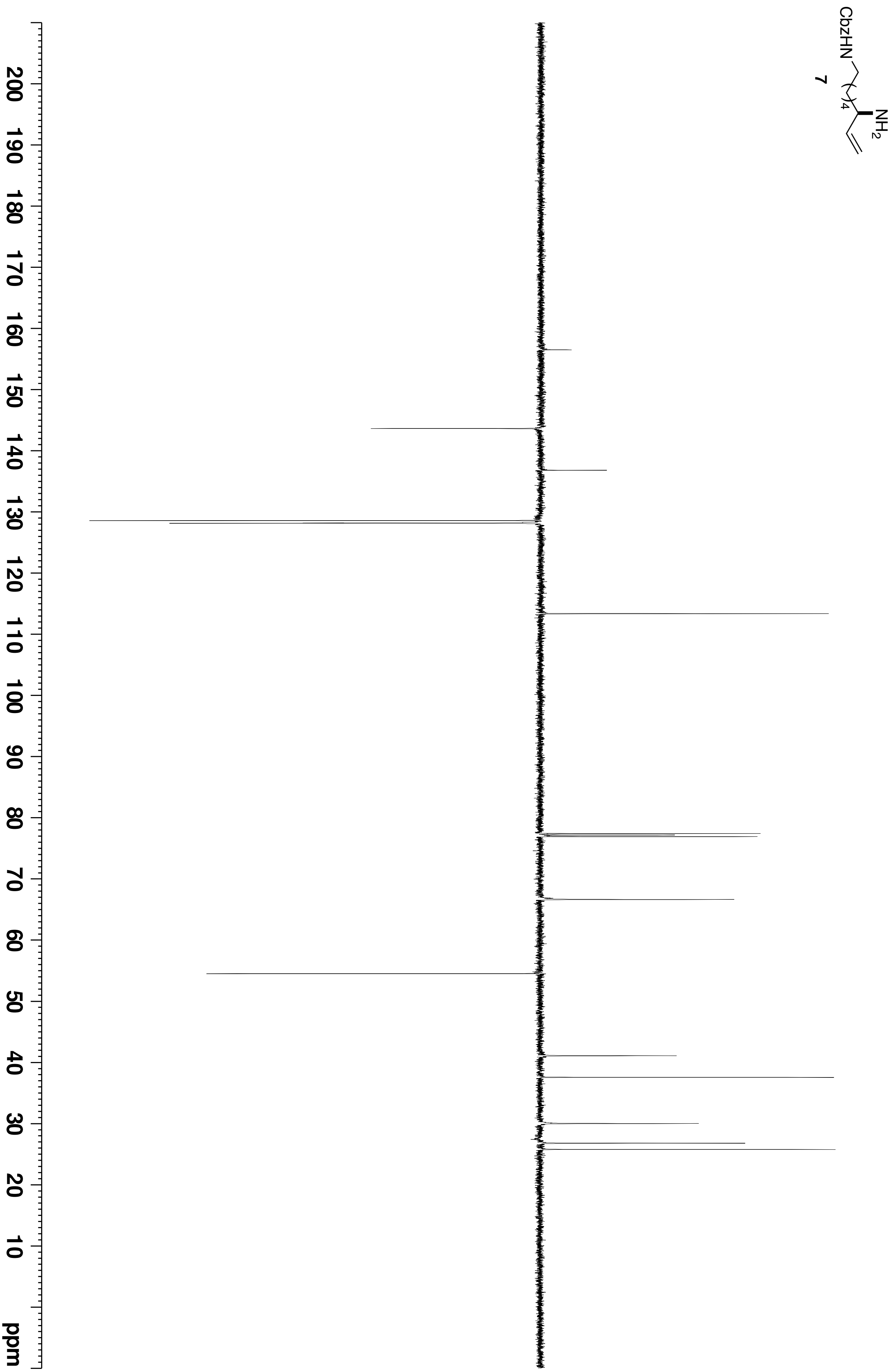
CbzHN
NH2
4
7
200
190
180
170
160
150
140
130
120
110
100
90
80
70
60
50
40
30
20
10
ppm
